# Straightforward Synthesis of Indenes by Gold-Catalyzed Intramolecular Hydroalkylation of Ynamides

**DOI:** 10.1021/acsorginorgau.1c00021

**Published:** 2021-10-14

**Authors:** Pierre Thilmany, Alejandro Guarnieri-Ibáñez, Clément Jacob, Jérôme Lacour, Gwilherm Evano

**Affiliations:** † Laboratoire de Chimie Organique, Service de Chimie et PhysicoChimie Organiques, Université libre de Bruxelles (ULB), Avenue F. D. Roosevelt 50, CP160/06, 1050 Brussels, Belgium; ‡ Department of Organic Chemistry, University of Geneva, Quai Ernest Ansermet 30, 1211 Geneva 4, Switzerland; § Organic Synthesis Division, Department of Chemistry, University of Antwerp, Groenenborgerlaan 171, 2020 Antwerp, Belgium

**Keywords:** gold catalysis, ynamides, indenes, H-shift, hydroalkylation

## Abstract

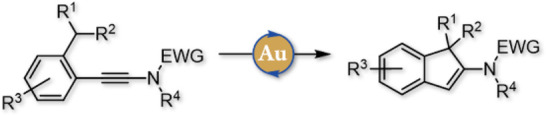

An original and straightforward entry to polysubstituted indenes from readily available ynamides is reported. Upon reaction with a *N*-heterocyclic carbene–gold complex under mild conditions, activated keteniminium ions are generated whose unique electrophilicity triggers a [1,5]-hydride shift and a subsequent cyclization. The presence of an endocyclic enamide in the densely functionalized resulting indenes was shown to be especially useful and versatile, offering a range of opportunities for their further postfunctionalization.

## Introduction

Over the last two decades, ynamides,^1−3^ readily prepared by a range of efficient methods,^4−11^ have emerged as remarkably versatile building blocks enabling the development of a variety of processes based on their unique reactivity. They have indeed been shown to display an exceptional level of reactivity and to participate in anionic,^12^ carbocationic,^13,14^ radical,^15,16^ and metal-catalyzed reactions^17,18^ as well as cycloadditions^19^ with exquisite levels of regioselectivity.^20^ They have moreover been shown to be excellent precursors of carbenoids,^3^ to provide new opportunities in asymmetric synthesis,^18,21^ and to enable the design of efficient and innovative routes to a variety of natural products.^1−3^ Among all reactions developed from these unique building blocks, the cationic ones have received much attention in recent years. In fact, they rely on the formation of highly electrophilic activated keteniminium ions^13,14,22^ whose reactivity has enabled the development of processes that would fail with other alkynes and/or less activated keteniminium ions. These activated keteniminium ions, readily generated upon reaction between an ynamide and an acid, an electrophile, or a π-acidic metal complex, are indeed among the most electrophilic intermediates known to date and readily react even with the worst nucleophiles.^23−28^ They have moreover been shown to be reactive enough to promote hydrogen and hydride shifts,^29−33^ even from relatively nonactivated positions,^34^ which have been used to develop a series of innovative and efficient processes to access a variety of building blocks and molecules, ranging from the simplest ones to remarkably complex nitrogen-containing heterocycles.

**Scheme 1:**
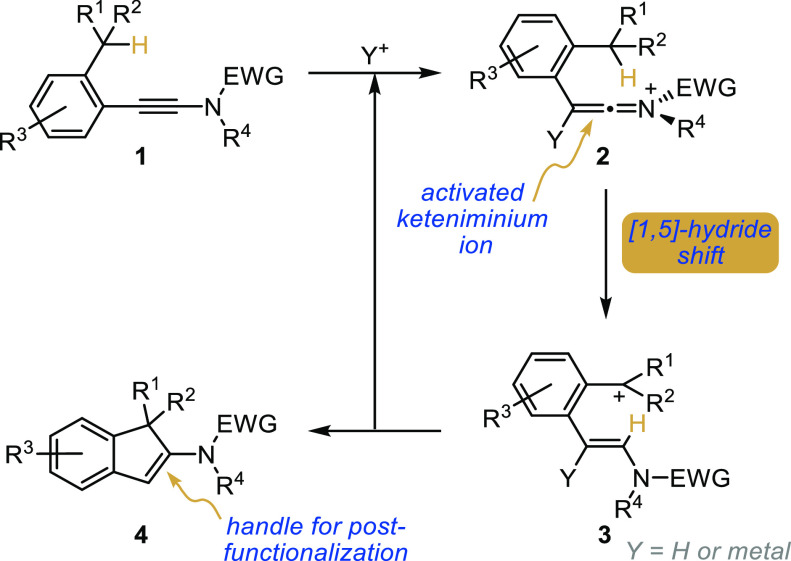
Working Hypothesis: Ynamides as Precursors of Highly Substituted Indenes

In line with our long-standing interest in the chemistry of ynamides^35−40^ as well as their use to promote hydride shifts^30,31,34^ and inspired by the remarkable studies from the Davies group,^32^ we hypothesized that ynamides such as **1** might be suitable precursors for such processes (Scheme 1). Indeed, their activation with an acid or a π-acidic metal complex should yield activated keteniminium ions **2**, which should trigger a [1,5]-hydride shift (or a related [1,5]-hydrogen shift) from the activated benzylic position generating enamides **3** whose cyclization followed by loss of a proton or protodemetalation would afford substituted indenes **4** in which the endocyclic enamide represents an especially useful handle for further diversification. Stimulated by this working hypothesis and the possibility it offers to develop a new route to polysubstituted indenes, useful building blocks found in a variety of biologically relevant products,^41−45^ we first evaluated the feasibility of this process.

## Results and Discussion

### Optimization

**Figure 1:**
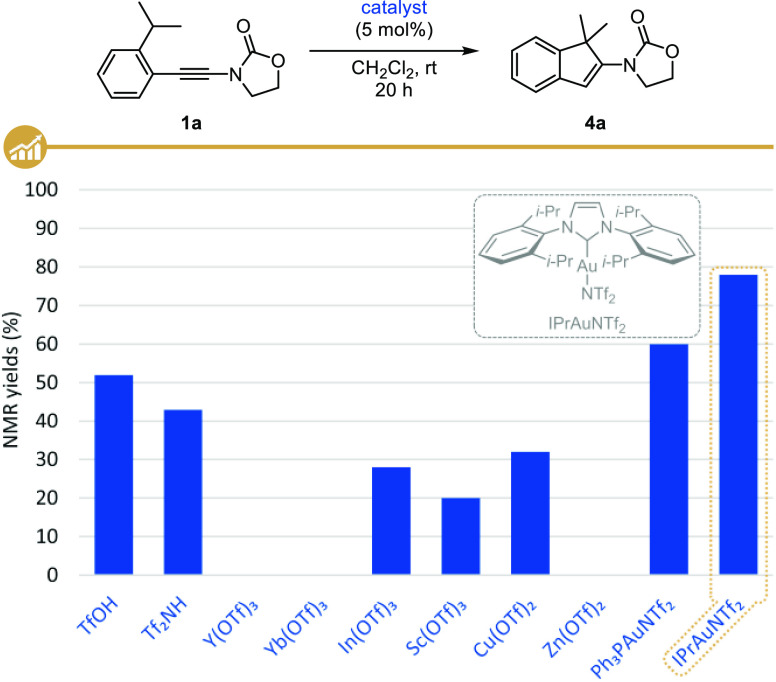
Optimization of the catalytic cyclization.

With this goal in mind, ynamide **1a**, selected as a model substrate, was reacted with catalytic amounts (5 mol %) of a set of representative Brønsted acids and π-acidic metal complexes at room temperature for 20 h in dichloromethane. These catalysts were selected based on the need for their conjugated bases or counterions to be as poorly nucleophilic as possible to avoid trapping the transient activated keteniminium ion, and dichloromethane was chosen as the solvent for the same reason. Results from this study are shown in Figure 1 and show the validity of our working hypothesis, with indene **4a** being formed in most trials. While the reaction was found to be promoted by strong acids such as triflic acid and bistriflimide, their efficiency was however shown to be in the moderate range since only low conversions and yields could be obtained. If the use of metal triflates commonly used for the activation of alkynes and/or ynamides was not met with more success, switching to gold(I) catalysts enabled better conversions and yields,^46−50^ with the *N*-heterocyclic carbene (NHC)–gold complex IPrAuNTf_2_^51^ being superior to Ph_3_PAuNTf_2_.^52^ A 78% NMR yield could be obtained with IPrAuNTf_2_ that was therefore selected as the optimal catalyst. Interestingly, the catalyst loading can be reduced to 1 mol % with only a slight erosion of the yield (72% vs 78%). For practical reasons and to ensure a full conversion, a catalytic loading of 5 mol % was however kept for the optimized reaction conditions.

### Scope and Limitation Studies

**Figure 2:**
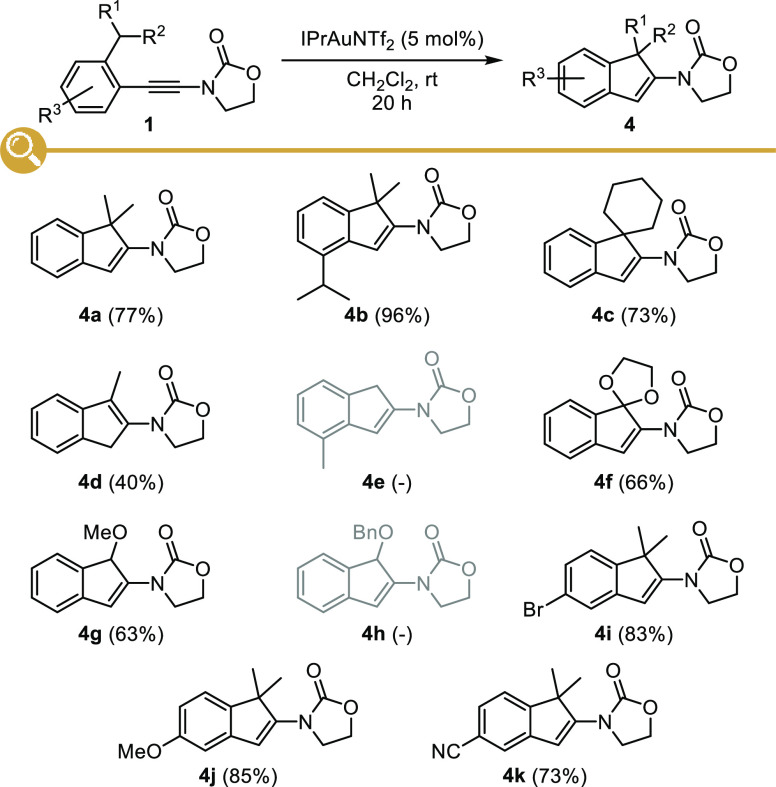
Scope of the gold-catalyzed cyclization: substitution of the aromatic ring.

With these optimized conditions in hands, we next moved to the study of the scope and limitations of this gold-catalyzed intramolecular hydroalkylation, first focusing on the influence of the nature of the group from which the hydride (or hydrogen) is transferred. As highlighted in Figure 2, tertiary positions such as isopropyl (**4a**,**b**) or cyclohexyl (**4c**) moieties are suitable for the H-shift to be operative, but secondary ones such as an ethyl group (**4d**) are also suitable, with a lower yield (40%) and an isomerization to the more stable alkene being however observed in this case. Not surprisingly, the absence of a substituent inhibited the hydroalkylation since no reaction was observed starting from *o*,*o*′-xylyl-ynamide **1e** that was fully recovered at the end of the reaction. Other groups favoring hydride or hydrogen shifts could also be utilized such as an acetal (**4f**) or a methyl ether (**4g**); a benzyl ether (**4h**) being however not tolerated and resulting in extensive degradation of the starting ynamide. Interestingly, the presence of an aromatic bromide did not interfere with the hydroalkylation, with indene **4i** being isolated in 83% yield, which offers a range of possibilities for further diversification by catalytic cross-coupling reactions. Finally, we could demonstrate that the presence of electron-donating or -withdrawing groups on the aromatic ring did not significantly impact the outcome of the reaction, as highlighted with the cyclization to **4j** and **4k** in 85% and 73% yield, respectively.

**Figure 3:**
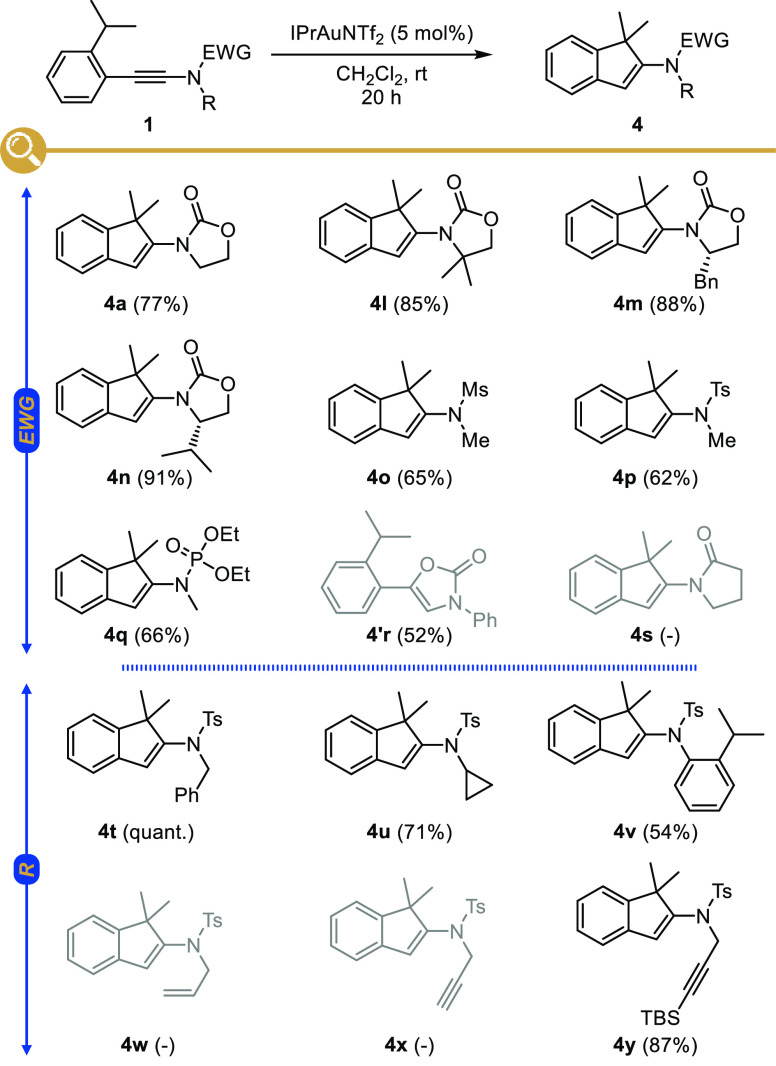
Scope of the gold-catalyzed cyclization: substitution at the nitrogen atom.

The influence of the other substituents in the starting ynamides **1**, namely, the electron-withdrawing group and the substituent on the nitrogen atom, was next investigated (Figure 3). Other oxazolidinone-derived ynamides could be smoothly converted with good to excellent yields to the corresponding indenes **4l**–**n**, regardless of the substitution of the oxazolidinone. With chiral oxazolidinone-derived ynamides being readily prepared by our previously reported copper-catalyzed alkynylation of the corresponding chiral oxazolidinones and *gem*-dibromoalkenes,^7,53,54^ their gold-catalyzed intramolecular hydroalkylation offers a straightforward access to optically enriched indenes **4m** and **4n** in which the chiral enamide represents an interesting handle for further derivatization. *N*-Alkynyl-sulfonamides and phosphoramidates were also readily cyclized to the corresponding indenes **4o**, **4p**, and **4q**, respectively, with a small decrease in efficiency, however, while starting from a *N*-Boc-substituted ynamine gave oxazolone **4′r** resulting from a faster cyclization of the carbamate to the activated keteniminium ion.^55,56^ An amide (**4s**) was not tolerated, and no conversion was observed in this case. The strong influence of the nature of the electron-withdrawing group cannot be rationalized in terms of nucleophilicity of the starting ynamides, with *N*-alkynyl-sulfonamides being more nucleophilic than *N*-alkynylamides,^30^ but rather depends on the relative electrophilicity of the activated keteniminium ions involved, species that are more reactive in the oxazolidinone series compared to the sulfonamide series.^57,58^ In the case of **4s**, the competing addition of the amide to the aurated keteneiminium ion might in addition be more favorable, thus preventing the hydrogen/hydride shift and trapping the gold catalyst.

With respect to the other substituents on the nitrogen atom of the starting ynamides, a *N*-benzyl group (**4t**), known to rapidly trap activated keteninium ions intramolecularly in a Pictet-Spengler-type cyclization,^59^ was shown to be compatible and did not interfere with the hydroalkylation, and an activated *N*-cyclopropyl group (**4u**) was left untouched. The cyclization yielding **4v**, obtained with a lower but still acceptable yield, nicely highlights the regioselectivity of the [1,5]-hydride shift which selectively involves the *ortho*-isopropyl-phenyl group at the β-position of the ynamide over the one on the nitrogen atom, most certainly due to a poor orbital overlap in the latter case. The presence of *N*-allyl (**4w**) and *N*-propargyl (**4x**) substituents however totally inhibited the reaction, which can be attributed to the formation of a two-coordinate gold π complex^60^ and preferred coordination to the terminal alkyne,^61^ respectively. Protecting the terminal alkyne by silylation however fully restored the reactivity, and the ynamide was in this case selectively activated by the gold catalyst to yield indene **4y**.

### Postfunctionalization

**Scheme 2:**
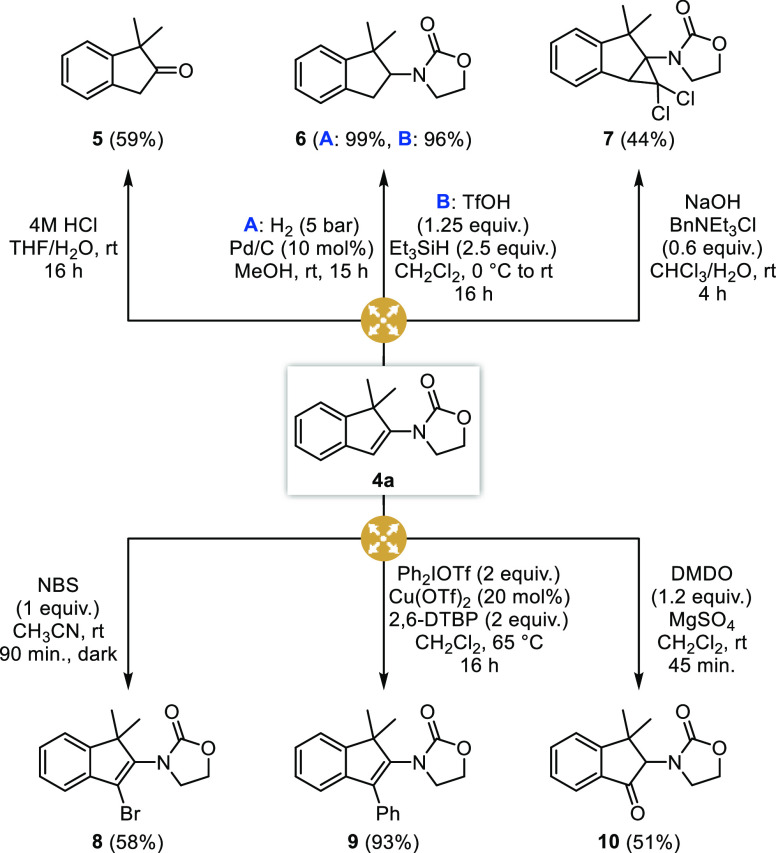
Postfunctionalization: Endocyclic Enamide as a Useful Handle for Chemical Diversification

Having studied the scope and limitations of this new intramolecular hydroalkylation of ynamides to indenes, we next focused our efforts on highlighting the synthetic potential and versatility of the indenes formed, with the endocyclic enamide moiety providing a useful handle for their postfunctionalization and diversification. In this perspective, a set of transformations was performed from indene **4a** that can be easily prepared on a large scale. As evidenced in Scheme 2, the enamide indeed turned out to be remarkably useful, with its hydrolysis under acidic conditions providing indan-2-one **5** while its reduction, either with molecular hydrogen under palladium catalysis or under ionic conditions, gave 2-amino-indane **6** in excellent yields. Cyclopropanation with chloroform under phase transfer catalysis provided fused cyclopropyl-indane **7** in a fair yield, while fully substituted indenes **8** and **9** could be smoothly obtained by an electrophilic bromination with *N*-bromo-succinimide and a copper-catalyzed arylation with diphenyliodonium triflate,^62^ respectively. Finally, a surprising result was observed upon reaction of **4a** with DMDO, 2-amino-indan-1-one **10**, resulting from a Meinwald rearrangement involving ring opening of the intermediate amino-epoxide followed by a [1,2]-hydride shift from the resulting β-hydroxy-iminium ion,^63,64^ being isolated in 51% yield. All together, these postfunctionalization reactions nicely evidence the versatility of the indenes resulting from the intramolecular hydroalkylation, with a range of polysubstituted indane and indene derivatives being readily obtained from a single precursor.

**Scheme 3:**
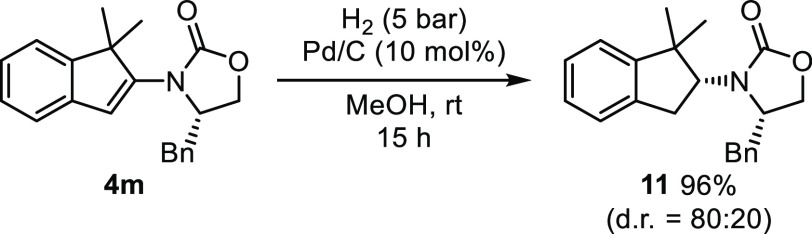
Diastereoselective Hydrogenation of a Chiral Oxazolidinone-Substituted Indene

Finally, with our process being especially convenient for the preparation of chiral oxazolodinone-derived indenes, we briefly envisioned their use for the synthesis of chiral, optically enriched indanes. Thus, enantiopure indene **4m** was subjected to catalytic hydrogenation, a reaction that proceeded smoothly to provide 2-amino-indane **11** in 96% yield and with a reasonable but still modest diastereoselectivity (Scheme 3).

### Proposed Catalytic Cycle

**Scheme 4:**
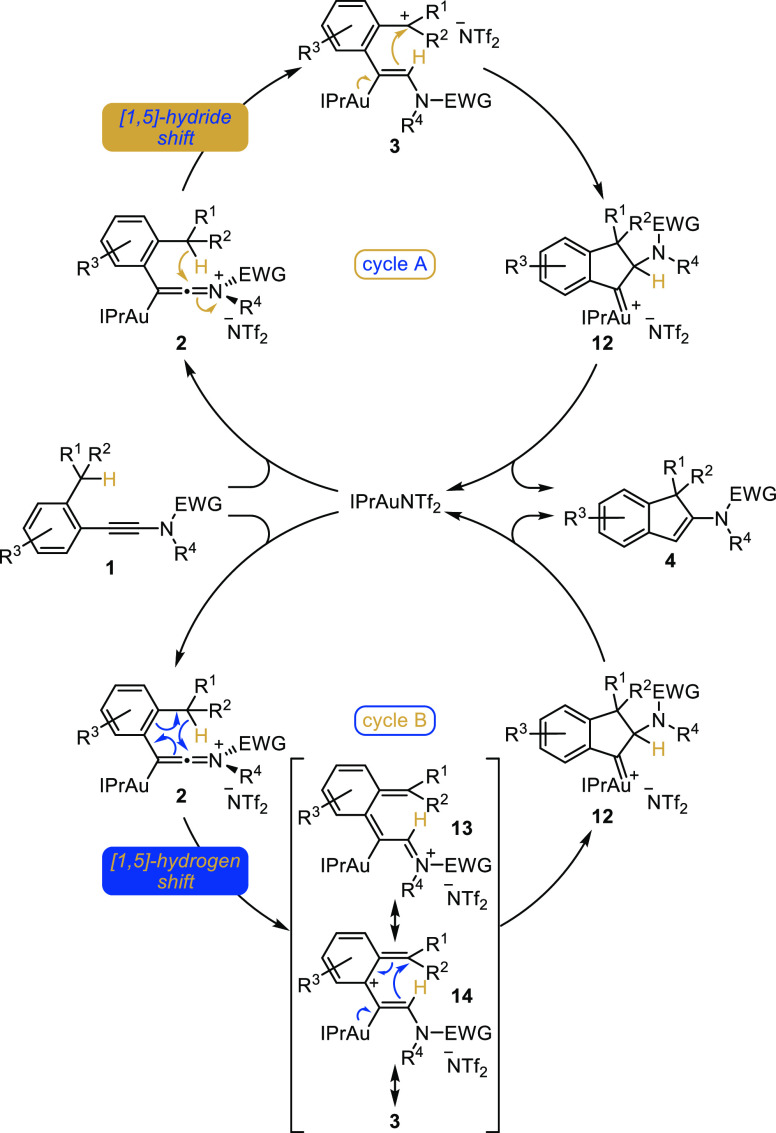
Mechanistic Proposal for the Gold-Catalyzed Cyclization

Regarding the mechanism of this gold-catalyzed intramolecular hydroalkylation, a plausible and reasonable proposal relying on a [1,5]-hydride shift is depicted in Scheme 4 (cycle A, top). Activation of the electron-rich alkyne in the starting ynamide **1** would result in the formation of a transient activated gold-keteniminium ion **2** that would trigger a [1,5]-hydride shift yielding to carbocation **3**. A subsequent cyclization involving the addition of the vinylgold in **3** to the carbocation would result in the formation of the five-membered ring in **12**. Alternatively, a concerted [1,5]-shift of hydrogen could also be operative from **2** (Scheme 4, cycle B, bottom): in this case, the cyclization could also proceed from **3** or from its resonance structure **14** by a Nazarov-type 4π-electrocyclization,^31^ a less likely pathway however due to the temporary disruption of the aromaticity in the transition state of this concerted process. In both cases, a [1,2]-hydride shift followed by elimination of the gold(I) catalyst^32^ or the loss of a proton followed by protodeauration of the resulting indenylgold complex would then account for the formation of indene **4**.

## Conclusion

In conclusion, we have developed a novel intramolecular hydroalkylation of readily available ynamides providing an original and straightforward entry to polysubstituted indenes. Upon simple reaction with a NHC–gold complex under especially mild conditions, activated keteniminium ions are generated whose unique electrophilicity triggers a [1,5]-hydride shift and a subsequent cyclization. The scope of the reaction was shown to be rather broad, and the presence of an endocyclic enamide in the densely functionalized resulting indenes was shown to be especially useful and versatile, offering a range of opportunities for their further postfunctionalization. In addition to the new entry to indenes it provides, this process further highlights the remarkable potential of the cationic chemistry of ynamides and the exceptional level of reactivity of activated keteniminium ions.

## Supporting Information Available

Experimental procedures, characterization, copies of ^1^H and ^13^C NMR spectra for all new compounds (PDF)
