# Supplementary material for: Straightforward Synthesis of Indenes by Gold-Catalyzed Intramolecular Hydroalkylation of Ynamides
Source: ACS Org Inorg Au. 2021 Oct 14;2(1):53–8. doi: 10.1021/acsorginorgau.1c00021 (PMC9954284; doi:10.1021/acsorginorgau.1c00021)
Supplement: Supplementary file 1 — gg1c00021_si_001.pdf [file gg1c00021_si_001.pdf]

Supporting Information

Straightforward Synthesis of Indenes by Gold-Catalyzed Intramolecular Hydroalkylation of Ynamides

Pierre Thilmany,[1] Alejandro Guarnieri-Ibáñez,[2] Clément Jacob,[1,3] Jérôme Lacour[2] and Gwilherm Evano[1]*

[1]Laboratoire de Chimie Organique, Service de Chimie et PhysicoChimie Organiques, Université libre de Bruxelles, Avenue F. D. Roosevelt 50, CP160/06, 1050 Brussels, Belgium.

[2]Department of Organic Chemistry, University of Geneva, Quai Ernest Ansermet 30, 1211 Geneva 4, Switzerland.

[3]Laboratory of Organic Synthesis, University of Antwerp, Groenenborgerlaan 171, 2020 Antwerp, Belgium.

General Information S2

Experimental Procedures and Characterization Data:
Unreported benzaldehydes S4

Experimental Procedures and Characterization Data:
Unreported *gem*-dibromoalkenes and bromoalkynes S7

Experimental Procedures and Characterization Data:
Unreported ynamides S11

Experimental Procedures and Characterization Data:
Intramolecular hydroalkylation of ynamides: 2-aminoindenes S22

Experimental Procedures and Characterization Data:
Post-functionalization of 2-aminoindenes S30

Copies of ^{1}H, ^{13}C and ^{31}P NMR spectra S35

General Information.

All reactions were carried out in oven-dried glassware under an argon atmosphere employing standard techniques in handling air-sensitive materials unless otherwise stated.

Acetonitrile (99.9%, Extra Dry, AcroSeal®), 1,4-dioxane (99.5%, Extra Dry over Molecular Sieve, AcroSeal®) and toluene (99.5%, Extra Dry over Molecular Sieve, AcroSeal®) were purchased from ACROS Organics and used as supplied. Dichloromethane was freshly distilled from calcium hydride under argon. All other solvents were reagent grade and used as supplied.

Copper(I) iodide (99,999% purity) and $IPrAuNTf_2$ (95% purity) were purchased from Merck/Sigma-Aldrich and used as supplied. Finely powdered anhydrous cesium carbonate was used for copper-mediated cross-coupling reactions. All other reagents were used as supplied.

Reactions were magnetically stirred and monitored by thin layer chromatography using Merck-Kieselgel 60 F_{254} plates. Flash chromatography was performed with silica gel 60 (particle size 35-70 µm) supplied by Merck. Yields refer to chromatographically and spectroscopically pure compounds unless otherwise stated.

Proton NMR spectra were recorded using an internal deuterium lock at ambient temperature on a Jeol 400 MHz spectrometer. Internal reference of δ_H 7.26 was used for $CDCl_3$. Data are presented as follows: chemical shift (in ppm on the δ scale relative to δ_{TMS} = 0), multiplicity (s = singlet, d = doublet, t = triplet, q = quartet, sept = septuplet, m = multiplet, app. = apparent), coupling constant (J/Hz) and integration. Resonances that are either partially or fully obscured are denoted obscured (obs.). Carbon-13 NMR spectra were recorded at 100 MHz using $CDCl_3$ (δ_C 77.16) as internal reference. Phosphorus-31 NMR spectra were recorded at 121 MHz using H_3PO_4 (δ_P 0.00) as external reference.

Optical rotations were recorded on an Anton Paar MCP-100 automatic polarimeter at 589 nm and reported as follows: $[\alpha]_D^{25}$, concentration (c in g/100 mL), and solvent. Melting points were recorded on a Stuart Scientific Analogue SMP11. Infrared spectra were recorded

on a Bruker Alpha (ATR). High-resolution mass-spectra (HRMS) in positive mode were recorded using a 6520 series quadrupole time-of-flight (Q-TOF) mass spectrometer (Agilent) fitted with a multimode ion source.

Experimental Procedures and Characterization Data
Unreported benzaldehydes

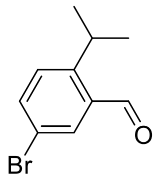

S1

5-Bromo-2-isopropyl-benzaldehyde S1. An oven-dried 250 mL round bottom flask was charged with $AlCl_3$ (5.40 g, 40.5 mmol) and fitted with a rubber septum before being evacuated under high vacuum and backfilled with argon three times. Freshly distilled dichloromethane (20 mL) was next added, and the resulting stirred solution was cooled to 0°C before a solution of 2-isopropyl-benzaldehyde (3.00 g, 20.2 mmol) in dichloromethane (20 mL) was added. A solution of Br_2 (1.35 mL, 26.3 mmol) in dichloromethane (40 mL) was next added and the resulting solution was stirred at 0 °C for 6 hours and at room temperature overnight. The solution was then carefully quenched by dropwise addition of a saturated aqueous solution of $Na_2S_2O_3$ (100 mL). The layers were separated, the aqueous layer was extracted with dichloromethane twice (2 x 100 mL) and the combined organic layers were dried over $MgSO_4$, filtered and concentrated under reduced pressure. The crude residue was finally purified by flash column chromatography over silica gel to afford 5-bromo-2-isopropyl-benzaldehyde **S1**. Yield: 28% (1.26 g, 5.56 mmol). Solvent system for flash column chromatography: petroleum ether/toluene: 80/20; Orange oil; ^{1}H NMR (400 MHz, $CDCl_3$): δ 10.31 (s, 1H), 7.93 (d, J = 2.3 Hz, 1H), 7.65 (dd, J = 8.4 and 2.3 Hz, 1H), 7.33 (d, J = 8.4 Hz, 1H), 3.88 (sept, J = 6.9 Hz, 1H), 1.29 (d, J = 6.9 Hz, 6H); ^{13}C NMR (100 MHz, $CDCl_3$): δ 190.7, 150.3, 137.0, 134.7, 133.6, 128.4, 120.2, 27.6, 23.9; IR (neat): ν_{max} 2967, 2871, 2729, 1690, 1587, 1480, 1459, 1386, 1206, 1179, 1096, 1051, 894, 876, 858, 830, 752, 731, 695, 644, 615 cm^{-1}; ESIHRMS m/z calcd. for $C_{10}H_{12}{}^{79}BrO$ $[M+H]^+$ 227.0066, found 227.0070.

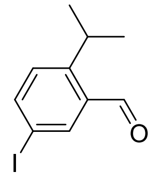

S2

5-Iodo-2-isopropyl-benzaldehyde S2. To a solution of 2-isopropyl-benzaldehyde (813 mg, 5.49 mmol) in trifluoroacetic acid (18 mL) was added *N*-iodosuccinimide (1.85 g, 8.24 mmol) portionwise at room temperature and the resulting mixture was stirred at room temperature for 24 hours. The reaction mixture was subsequently poured onto cold water (0 °C), the layers were separated and the aqueous one further extracted three times with dichloromethane. The combined organic layers were then washed with a saturated aqueous solution of sodium thiosulfate, dried over $MgSO_4$, filtered and concentrated under reduced pressure. The crude

residue was finally purified by flash column chromatography over silica gel to afford afford 5-iodo-2-isopropyl-benzaldehyde **S2**. Yield: 69% (1.04 g, 3.78 mmol). Solvent system for flash column chromatography: petroleum ether/EtOAc: 95/5 ; Yellow oil ; ^{1}H NMR (400 MHz, $CDCl_3$): δ 10.28 (s, 1H), 8.11 (d, J = 2.1 Hz, 1H), 7.85 (dd, J = 8.3 and 2.1 Hz, 1H), 7.20 (d, J = 8.3 Hz, 1H), 3.88 (sept, J = 6.9 Hz, 1H), 1.29 (d, J = 6.9 Hz, 6H); ^{13}C NMR (100 MHz, $CDCl_3$): δ 190.8, 151.0, 142.9, 139.8, 134.8, 128.5, 91.1, 27.7, 23.9; IR (neat): ν_{max} 2966, 2871, 2734, 1688, 1582 1476, 1207, 1091, 827, 749 cm^{-1}; ESIHRMS m/z calcd. for $C_{10}H_{12}IO$ [M+H]$^+$ 274.9927, found 274.9930.

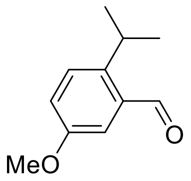

S3

2-Isopropyl-5-methoxy-benzaldehyde S3. An oven-dried 15 mL pressure tube was charged with 5-iodo-2-isopropyl-benzaldehyde **S2** (855 mg, 3.12 mmol), copper(I) iodide (30 mg, 156 µmol), 3,4,7,8-tetramethyl-1,10-phenanthroline (73 mg, 309 µmol) and cesium carbonate (1.53 g, 4.70 mmol). The tube was fitted with a rubber septum, evacuated under high vacuum and backfilled with argon three times. Dry toluene (1.6 mL) and dry methanol (253 µL, 6.24 mmol) were next added, the rubber septum was replaced by a Teflon-coated screw cap and the resulting suspension was stirred at 80 °C for 24h. The brownish suspension was cooled to room temperature, diluted with EtOAc, filtered over a plug of silica gel (washed with EtOAc) and concentrated under reduced pressure. The crude residue was finally purified by flash column chromatography over silica gel to afford 2-isopropyl-5-methoxy-benzaldehyde **S3**. Yield: 48% (267 mg, 1.50 mmol). Solvent system for flash column chromatography: petroleum ether/EtOAc: 98/2; Colorless oil; ^{1}H NMR (400 MHz, $CDCl_3$): δ 10.39 (s, 1H), 7.36 (d, J = 8.7 Hz, 1H), 7.34 (d, J = 2.3 Hz, 1H), 7.12 (dd, J = 8.6 and 2.3 Hz, 1H), 3.90-3.81 (m, 1H), 3.84 (s, 3H), 1.30 (d, J = 6.9 Hz, 6H); ^{13}C NMR (100 MHz, $CDCl_3$): δ 191.7, 158.0, 144.2, 133.8, 127.6, 121.5, 113.2, 55.6, 27.15, 24.3; IR (neat): ν_{max} 2964, 2871, 1685, 1608, 1498, 1281, 1245, 1167, 1033, 769 cm^{-1}; ESIHRMS m/z calcd. for $C_{11}H_{15}O_2$ [M+H]$^+$ 179.1067, found 179.1069.

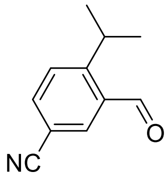

S4

5-Cyano-2-isopropyl-benzaldehyde S4. An oven-dried 60 mL pressure tube was charged with 5-bromo-2-isopropyl-benzaldehyde **S1** (1.50 g, 6.61 mmol), potassium hexacyanoferrate(II) trihydrate (1.12 g, 2.65 mmol) and tetrakis(triphenylphosphine)palladium(0) (381 mg, 330 µmol). The tube was fitted with a rubber septum, evacuated under high vacuum and backfilled with argon three times. *tert*-Butanol (10 mL), deionized water (Milli-Q®, 18 MΩ) (10 mL) and

1,8-diazabicyclo[5.4.0]undec-7-ene (250 µL, 1.65 mmol) were next added, the rubber septum was replaced by a Teflon-coated screw cap and the resulting mixture was stirred at 90 °C for 40h. The reaction mixture was cooled to room temperature, diluted with dichloromethane, filtered over a plug of Celite® (washed with dichloromethane) and concentrated under reduced pressure to a minimum volume. The layers were then separated, and the organic one was dried over $MgSO_4$, filtered and concentrated under reduced pressure. The crude residue was finally purified by flash column chromatography over silica gel to afford 2-isopropyl-5-cyanobenzaldehyde **S4**. Yield: 41% (466 mg, 2.69 mmol). Solvent system for flash column chromatography: petroleum ether/EtOAc: 95/5; White solid;Mp: 116 °C; ^{1}H NMR (400 MHz, $CDCl_3$): δ 10.37 (s, 1H), 8.11 (d, J = 1.9 Hz, 1H), 7.81 (dd, J = 8.2 and 1.9 Hz, 1H), 7.59 (d, J = 8.2 Hz, 1H), 3.99 (sept, J = 6.8 Hz, 1H), 1.33 (d, J = 6.9 Hz, 6H); ^{13}C NMR (100 MHz, $CDCl_3$): δ 190.1, 156.3, 136.7, 134.8, 133.8, 127.7, 118.0, 110.9, 28.3, 23.7; IR (neat): ν_{max} 2971, 2872, 2233, 1691, 1492, 1226, 1169, 1054, 910, 763 cm^{-1}; ESIHRMS *m/z* calcd. for $C_{11}H_{12}NO$ $[M+H]^+$ 174.0913, found 174.0916.

Experimental Procedures and Characterization Data
Unreported *gem*-dibromoalkenes and bromoalkynes

General procedure I:
To a solution of carbon tetrabromide (4.78 g, 14.4 mmol) in anhydrous dichloromethane (35 mL) was added dropwise a solution of triphenylphosphine (7.08 g, 27.0 mmol) in anhydrous dichloromethane (35 mL) at 0 °C under argon over 30 minutes. The aldehyde (6.00 mmol) was then added dropwise to the resulting orange solution and the resulting mixture was stirred at 0 °C for 30 minutes, slowly warmed to room temperature and stirred for the appropriate amount of time (90 min – 16 h). The mixture was then diluted with petroleum ether 50 mL), filtered over a plug of silica gel (washed with petroleum ether) and concentrated. When necessary, the crude residue was purified by flash column chromatography over silica gel to afford the desired 1,1-dibromo-1-alkene.

General procedure II:
An oven-dried 100 mL round bottom flask was fitted with a rubber septum before being evacuated under high vacuum and backfilled with argon three times. The alkyne (4.00 mmol), dry acetone (20 mL), *N*-bromosuccinimide (783 mg, 4.40 mmol) and $AgNO_3$ (68 mg, 400 µmol) were then successively added, and the resulting mixture was stirred at room temperature in the dark for the appropriate amount of time (2 – 18 h). The mixture was then diluted with petroleum ether (50 mL), filtrated, and concentrated under reduced pressure. The resulting residue was finally filtered over a plug of silica gel (washed with petroleum ether) to afford the desired bromoalkyne without further purification.

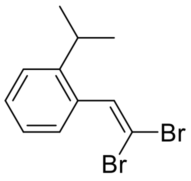

S5

(2,2-Dibromoethenyl)-2-isopropyl-benzene S5. Prepared according to general procedure I (18.6 mmol of the corresponding aldehyde, 16 h). Yield: 83% (4.70 g, 15.5 mmol). ^{1}H NMR (400 MHz, $CDCl_3$): δ 7.57 (s, 1H), 7.37-7.29 (m, 3H), 7.23-7.18 (m, 1H), 3.01 (sept, J = 6.8 Hz, 1H), 1.24 (d, J = 6.9 Hz, 6H); ^{13}C NMR (100 MHz, $CDCl_3$): δ 146.6, 137.3, 134.6, 129.2, 129.0, 125.9, 125.3, 92.2, 30.6, 23.5; IR (neat): ν_{max} 2962, 2927, 2869, 1481, 1462, 1447, 1384, 1364, 1081, 1034, 883, 856, 796, 757, 739 cm^{-1}; ESIHRMS m/z calcd. for $C_{11}H_{12}{}^{79}Br^{81}BrK$ $[M+K]^+$ 342.8917, found 342.8914.

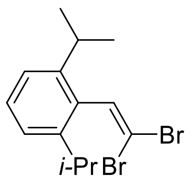

S6

2-(2,2-Dibromoethenyl)-1,3-diisopropyl-benzene S6. Prepared according to general procedure I (6.57 mmol of the corresponding aldehyde, 16 h). Yield: 86% (1.96 g, 5.66 mmol). Colorless oil; ^{1}H NMR (400 MHz, $CDCl_3$): δ 7.48 (s, 1H), 7.35 (t, J = 7.7 Hz, 1H), 7.17 (d, J = 7.8 Hz, 2H), 3.04 (sept, J = 6.9 Hz, 2H), 1.29 (d, J = 6.9 Hz, 6H), 1.17 (d, J = 6.9 Hz, 6H); ^{13}C NMR (100 MHz, $CDCl_3$): δ 146.5, 137.2, 133.5, 129.0, 123.0, 93.3, 31.0, 24.2, 23.6; IR (neat): ν_{max} 2962, 2927, 2868, 1593, 1466, 1384, 1363, 1252, 1056, 936, 873, 789, 739 cm^{-1}.

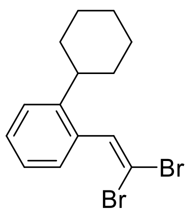

S7

2-Cyclohexyl-(2,2-dibromoethenyl)-benzene S7. Prepared according to general procedure I (3.72 mmol of the corresponding aldehyde, 16 h). Yield: 64% (821 mg, 2.39 mmol). Colorless oil; ^{1}H NMR (400 MHz, $CDCl_3$): δ 7.56 (s, 1H), 7.35-7.26 (m, 3H), 7.22-7.17 (m, 1H), 2.56 (tt, J = 11.6 and 3.2 Hz, 1H), 1.88-1.74 (m, 5H), 1.48-1.35 (m, 4H), 1.33-1.22 (m, 1H); ^{13}C NMR (100 MHz, $CDCl_3$): δ 145.8, 137.3, 134.7, 129.3, 128.9, 126.0, 125.8, 92.1, 41.3, 33.9, 27.1, 26.3; IR (neat): ν_{max} 2925, 2851, 1477, 1447, 998, 879, 855, 830, 788, 750 cm^{-1}; ESIHRMS *m/z* calcd. for $C_{14}H_{16}{}^{79}Br^{81}BrNa$ $[M+Na]^+$ 366.9491, found 366.9497.

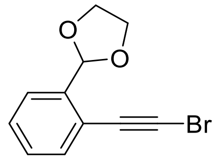

S8

(Bromoethynyl)-2-(1,3-dioxolan-2-yl)-benzene S8. Prepared according to general procedure II (4.02 mmol of the corresponding alkyne, 2 h). Yield: 97% (948 mg, 3.89 mmol). Yellow oil; ^{1}H NMR (400 MHz, $CDCl_3$): δ 7.56 (dd, J = 7.7 and 1.3 Hz, 1H), 7.48 (dd, J = 7.6 and 1.3 Hz, 1H), 7.37 (td, J = 7.6 and 1.4 Hz, 1H), 7.31 (td, J = 7.5 and 1.5 Hz, 1H), 6.14 (s, 1H), 4.22-4.03 (m, 4H); ^{13}C NMR (100 MHz, $CDCl_3$): δ 139.9, 133.3, 129.2, 129.0, 126.5, 122.1, 102.1, 77.6, 65.8, 54.2; IR (neat): ν_{max} 2973, 2957, 2889, 2196, 1394, 1229, 1196, 1115, 1073, 1026, 969, 945, 760, 620 cm^{-1}; ESIHRMS *m/z* calcd. for $C_{11}H_9{}^{79}BrO_2Na$ $[M+Na]^+$ 274.9678, found 274.9657.

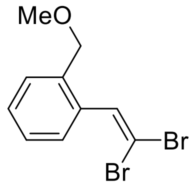

S9

(2,2-Dibromoethenyl)-2-(methoxymethyl)-benzene S9. Prepared according to general procedure I (6.00 mmol of the corresponding aldehyde, 16 h). For this compound, the plug of silica gel was washed with a 80/20 mixture of petroleum ether and ethyl acetate instead of petroleum ether. Yield: 66% (1.21 g, 3.95 mmol). Solvent system for flash column chromatography: petroleum ether/EtOAc: 98/2; Yellow oil; ^{1}H NMR (400 MHz, $CDCl_3$): δ 7.61 (s, 1H), 7.48-7.45 (m, 1H), 7.41-7.37 (m, 1H), 7.37-7.29 (m, 2H), 4.41 (s, 2H), 3.40 (s, 3H); ^{13}C NMR (100 MHz, $CDCl_3$): δ 136.0, 135.9, 135.4, 129.0, 128.9, 128.7, 128.0, 92.1, 72.7, 58.5; IR (neat): ν_{max} 2983, 2925, 2888, 2822, 1601, 1450, 1381, 1192, 1108, 1089, 946, 885, 862, 765, 749 cm^{-1}; ESIHRMS *m/z* calcd. for $C_{10}H_{10}{}^{79}Br^{81}BrONa$ $[M+Na]^+$ 328.8970, found 328.8963.

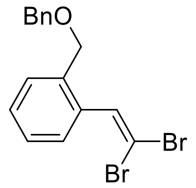

S10

2-[(Benzyloxy)methyl]-(2,2-dibromoethenyl)-benzene S10. Prepared according to general procedure I (7.00 mmol of the corresponding aldehyde, 2 h 30). Yield: 46% (1.23 g, 3.22 mmol). Solvent system for flash column chromatography: petroleum ether/EtOAc: 95/5; Yellow oil; ^{1}H NMR (400 MHz, $CDCl_3$): δ 7.62 (s, 1H), 7.51-7.47 (m, 1H), 7.45-7.41 (m, 1H), 7.40-7.38 (m, 4H), 7.37-7.31 (m, 3H), 4.58 (s, 2H), 4.51 (s, 2H); ^{13}C NMR (100 MHz, $CDCl_3$): δ 138.1, 136.1, 135.9, 135.5, 129.2, 129.0, 128.7, 128.6, 128.0 (2C), 127.9, 92.0, 72.7, 70.3.

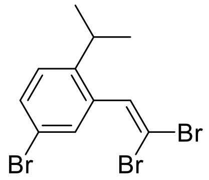

S11

4-Bromo-2-(2,2-dibromoethenyl)-1-isopropyl-benzene S11. Prepared according to general procedure I (4.50 mmol of the corresponding aldehyde, 16 h). Yield: 78% (1.34 g, 3.49 mmol). Yellow oil; ^{1}H NMR (400 MHz, $CDCl_3$): δ 7.48 (s, 1H), 7.46-7.42 (m, 2H), 7.18-7.15 (m, 1H), 2.95 (sept, J = 6.9 Hz, 1H), 1.20 (d, J = 6.9 Hz, 6H); ^{13}C NMR (100 MHz, $CDCl_3$): δ 145.7, 136.5, 135.7, 131.9, 131.8, 127.2, 119.3, 93.6, 30.3, 23.4; IR (neat): ν_{max} 2963, 2927, 2868, 2848, 1596, 1557, 1477, 1394, 1364, 1246, 1223, 1183, 1096, 1051, 907, 877, 818, 737 cm^{-1}; ESIHRMS *m/z* calcd. for $C_{11}H_{11}{}^{79}Br_2{}^{81}BrNa$ $[M+Na]^+$ 404.8283, found 404.8291.

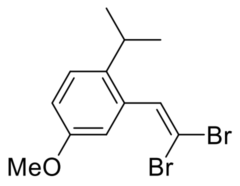

S12

2-(2,2-Dibromoethenyl)-1-isopropyl-4-methoxy-benzene S12. Prepared according to general procedure I (1.39 mmol of the corresponding aldehyde, 15 h). Yield: 86% (398 mg, 1.19 mmol). Colorless oil; ^{1}H NMR (400 MHz, $CDCl_3$): δ 7.54 (s, 1H), 7.21 (d, J = 8.5 Hz, 1H), 6.91-6.85 (m, 2H), 3.80 (s, 3H), 2.94 (sept, J = 6.9 Hz, 1H), 1.20 (d, J = 6.9 Hz, 6H); ^{13}C NMR (100 MHz, $CDCl_3$): δ 157.4, 138.8, 137.1, 135.4, 126.3, 114.8, 114.2, 92.2, 55.5, 29.9, 23.7; IR (neat): ν_{max} 3000, 2869, 2834, 1607, 1573, 1491, 1251, 1167, 1035, 820, 739 cm^{-1}; ESIHRMS *m/z* calcd. for $C_{12}H_{15}{}^{79}Br^{81}BrO$ $[M+H]^+$ 334.9464, found 334.9465.

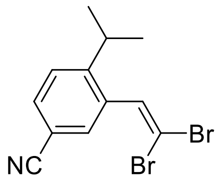

S13

4-Cyano-2-(2,2-dibromoethenyl)-1-isopropyl-benzene S13. Prepared according to general procedure I (2.56 mmol of the corresponding aldehyde, 18 h). Yield: 94% (791 mg, 2.40 mmol). White solid; Mp: 42 °C, ^{1}H NMR (400 MHz, $CDCl_3$): δ 7.62-7.58 (m, 2H), 7.49 (s, 1H), 7.40 (d, J = 8 Hz, 1H), 3.04 (sept, J = 6.9 Hz, 1H), 1.23 (d, J = 6.9 Hz, 6H); ^{13}C NMR (100 MHz, $CDCl_3$): δ 152.2, 135.8, 134.9, 133.0, 132.4, 126.5, 118.7, 110.0, 94.6, 30.9, 23.0; IR (neat): ν_{max} 2967, 2859, 2370, 2337, 2210, 1604, 1559, 1488, 1290, 1060, 910, 841, 747 cm^{-1}; ESIHRMS *m/z* calcd. for $C_{12}H_{12}{}^{79}Br^{81}BrN$ $[M+H]^+$ 329.9311, found 329.9315.

Experimental Procedures and Characterization Data
Unreported Ynamides

General procedure III: copper-catalyzed alkynylation of nitrogen nucleophiles with *gem*-dibromoalkenes[S1]

An oven-dried 15 mL pressure tube was charged with the nitrogen nucleophile (1.60 mmol), the 1,1-dibromo-1-alkene (2.40 mmol), cesium carbonate (2.09 g, 6.40 mmol) and copper(I) iodide (37 mg, 192 µmol). The tube was fitted with a rubber septum, evacuated under high vacuum and backfilled with argon three times. Dry 1,4-dioxane (3 mL) and *N*,*N*'-dimethylethylenediamine (31 µL, 288 µmol) were next added, the rubber septum was replaced by a Teflon-coated screw cap and the resulting light blue-green suspension was stirred at the appropriate temperature (70 – 80 °C) for the appropriate amount of time (16 – 84 h). The brownish suspension was cooled to room temperature, filtered over a plug of silica gel (washed with EtOAc) and concentrated. The crude residue was finally purified by flash column chromatography over silica gel to afford the desired ynamide.

General procedure IV: copper-catalyzed alkynylation of nitrogen nucleophiles with bromoalkynes[S2]

An oven-dried 15 mL pressure tube was charged with the nitrogen nucleophile (2.00 mmol), potassium carbonate or tripotassium phosphate (4.00 mmol), $CuSO_4{\cdot}5H_2O$ (50 mg, 200 µmol), 1,10-phenanthroline (72 mg, 400 µmol) and the bromoalkyne (2.20 mmol). The tube was fitted with a rubber septum, evacuated under high vacuum and backfilled with argon three times. Dry toluene (2 mL) was next added, the rubber septum was replaced by a Teflon-coated screw cap and the mixture was stirred at the appropriate temperature (75 – 95 °C) for the appropriate amount of time (36 – 48 h). The reaction mixture was then cooled to room temperature, filtered over a plug of silica (washed with EtOAc) and concentrated under reduced pressure. The crude residue was finally purified by flash column chromatography over silica gel to afford the desired ynamide.

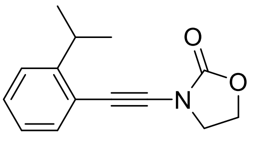

1a

3-[(2-Isopropylphenyl)ethynyl]oxazolidin-2-one 1a. Prepared according to general procedure III (2.40 mmol of the corresponding nitrogen nucleophile, 70 °C, 48 h). Yield: 74% (407 mg, 1.78 mmol). Solvent system for flash column chromatography: petroleum ether/EtOAc: 85/15; Yellow oil; ^{1}H NMR (400 MHz, $CDCl_3$): δ 7.40 (dt, J = 7.7 and 1.1 Hz, 1H), 7.28-7.25 (m, 2H), 7.14-7.09 (m, 1H), 4.50-4.45 (m, 2H), 4.02-3.97 (m, 2H), 3.42 (sept, J = 6.9 Hz, 1H), 1.26 (d, J =

[S1] Coste, A.; Karthikeyan, G.; Couty, F.; Evano, G. *Angew. Chem. Int. Ed.* **2009**, *48*, 4381-4385.
[S2] Zhang, Y.; Hsung, R. P.; Tracey, M. R.; Kurtz, K. C. M.; Vera, E. L. *Org. Lett.* **2004**, *6*, 1151-1154.

6.9 Hz, 6H); ^{13}C NMR (100 MHz, CDCl$_3$): δ 156.0, 150.4, 132.3, 128.6, 125.6, 125.0, 121.0, 82.5, 70.0, 63.1, 47.2, 31.6, 23.2; IR (neat): ν_{max} 2963, 2926, 2870, 2257, 1771, 1479, 1411, 1200, 1169, 1091, 1034, 750, 711, 627 cm^{-1}; ESIHRMS *m/z* calcd. for $C_{14}H_{16}NO_2$ [M+H]$^+$ 230.1176, found 230.1186.

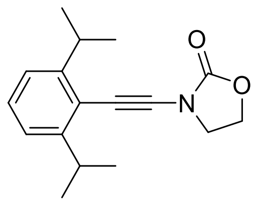

1b

3-[(2,6-Diisopropylphenyl)ethynyl]oxazolidin-2-one 1b. Prepared according to general procedure III (1.64 mmol of the corresponding nitrogen nucleophile, 70 °C, 48 h). Yield: 79% (351 mg, 1.29 mmol). Solvent system for flash column chromatography: petroleum ether/EtOAc: 80/20; White solid, Mp: 118 °C; ^{1}H NMR (400 MHz, CDCl$_3$): δ 7.28-7.23 (m, 1H), 7.12 (d, *J* = 7.7 Hz, 2H), 4.54-4.48 (m, 2H), 4.06-4.01 (m, 2H), 3.47 (sept, *J* = 6.9 Hz, 2H), 1.26 (d, *J* = 6.9 Hz, 12H); ^{13}C NMR (100 MHz, CDCl$_3$): δ 155.8, 150.6, 128.4, 122.3, 120.0, 86.7, 68.4, 63.0, 47.2, 31.9, 23.3; IR (neat): ν_{max} 2964, 2870, 2255, 1755, 1478, 1415, 1216, 1162, 1108, 1030, 801, 752 cm^{-1}; ESIHRMS *m/z* calcd.. for $C_{17}H_{22}NO_2$ [M+H]$^+$ 272.1645, found 272.1659.

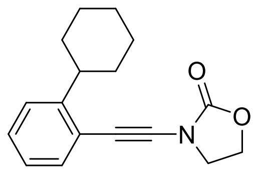

1c

3-[(2-Cyclohexylphenyl)ethynyl]oxazolidin-2-one 1c. Prepared according to general procedure III (1.33 mmol of the corresponding nitrogen nucleophile, 75 °C, 60 h). Yield: 32% (115 mg, 427 µmol). Solvent system for flash column chromatography: petroleum ether/EtOAc: 80/20; Yellow oil; ^{1}H NMR (400 MHz, CDCl$_3$): δ 7.40 (d, *J* = 7.7 Hz, 1H), 7.29-7.21 (m, 2H), 7.14-7.09 (m, 1H), 4.53-4.47 (m, 2H), 4.05-3.99 (m, 2H), 3.03 (tt, *J* = 11.2 and 3.0 Hz, 1H), 1.94-1.82 (m, 4H), 1.80-1.72 (m, 1H), 1.53-1.33 (m, 4H), 1.32-1.20 (m, 1H); ^{13}C NMR (100 MHz, CDCl$_3$): δ 155.9, 149.4, 132.0, 128.5, 125.6 (2C), 121.3, 82.6, 70.3, 63.1, 47.2, 42.2, 33.6, 27.1, 26.4; IR (neat): ν_{max} 2923, 2853, 2259, 1776, 1759, 1479, 1449, 1412, 1205, 1170, 1033, 757, 747, 630 cm^{-1}; ESIHRMS *m/z* calcd.. for $C_{17}H_{20}NO_2$ [M+H]$^+$ 270.1489, found 270.1490.

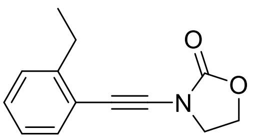

1d

3-[(2-Ethylphenyl)ethynyl]oxazolidin-2-one 1d. Prepared according to general procedure III (3.68 mmol of the corresponding nitrogen nucleophile, 75 °C, 40 h). Yield: 60% (474 mg, 2.20 mmol). Solvent system for flash column chromatography: petroleum ether/EtOAc: gradient from 85/15 to 70/30; Yellow oil; ^{1}H NMR (400 MHz, $CDCl_3$): δ 7.40 (dd, J = 7.6 and 1.2 Hz, 1H), 7.27-7.19 (m, 2H), 7.13 (td, J = 7.2 and 1.9 Hz, 1H), 4.52-4.46 (m, 2H), 4.04-3.99 (m, 2H), 2.81 (q, J = 7.6 Hz, 2H), 1.25 (t, J = 7.6 Hz, 3H); ^{13}C NMR (100 MHz, $CDCl_3$): δ 156.0, 146.1, 132.1, 128.5, 128.0, 125.7, 121.4, 82.4, 70.1, 63.1, 47.2, 27.8, 14.8; IR (neat): ν_{max} 2967, 2931, 2873, 2258, 1770, 1478, 1411, 1205, 1119, 1079, 1034, 971, 749, 628 cm^{-1}; ESIHRMS *m/z* calcd.. for $C_{13}H_{14}NO_2$ $[M+H]^+$ 216.1019, found 216.1022.

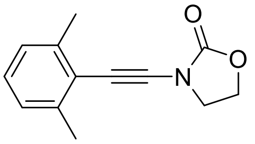

1e

3-[(2,6-Dimethylphenyl)ethynyl]oxazolidin-2-one 1e. Prepared according to general procedure III (5.00 mmol of the corresponding nitrogen nucleophile, 70 °C, 48 h). Yield: 76% (820 mg, 3.81 mmol). Solvent system for flash column chromatography: petroleum ether/EtOAc: 80/20; Off-white solid; ^{1}H NMR (400 MHz, $CDCl_3$): δ 7.12-7.07 (m, 1H), 7.05-7.01 (m, 2H), 4.53-4.47 (m, 2H), 4.05-4.00 (m, 2H), 2.43 (s, 6H); ^{13}C NMR (100 MHz, $CDCl_3$): δ 155.9, 139.9, 127.6, 126.8, 122.1, 87.2, 69.1, 63.1, 47.4, 21.2.

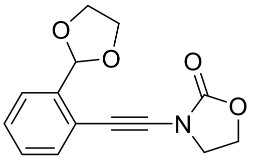

1f

3-{[2-(1,3-Dioxolan-2-yl)phenyl]ethynyl}oxazolidin-2-one 1f. Prepared according to general procedure IV (1.70 mmol of the corresponding nitrogen nucleophile, K_2CO_3, 75 °C, 48 h). Yield: 80% (351 mg, 1.35 mmol). Solvent system for flash column chromatography: petroleum ether/EtOAc: 40/60; Orange oil; ^{1}H NMR (400 MHz, $CDCl_3$): δ 7.58-7.54 (m, 1H), 7.46-7.42 (m, 1H), 7.36-7.27 (m, 2H), 6.15 (s, 1H), 4.49-4.43 (m, 2H), 4.22-4.02 (m, 4H), 4.02-3.97 (m, 2H); ^{13}C NMR (100 MHz, $CDCl_3$): δ 155.8, 138.6, 132.2, 129.1, 128.3, 126.4, 121.7, 102.2, 83.6, 69.0, 65.7, 63.2, 47.0; IR (neat): ν_{max} 2972, 2891, 2257, 1769, 1478, 1415, 1396, 1203, 1169, 1116, 1069, 1032, 971, 945, 762, 748 cm^{-1}; ESIHRMS *m/z* calcd.. for $C_{14}H_{14}NO_4$ $[M+H]^+$ 260.0917, found 260.0916.

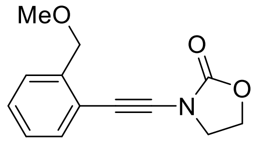

1g

3-{[2-(Methoxymethyl)phenyl]ethynyl}oxazolidin-2-one 1g. Prepared according to general procedure III (2.00 mmol of the corresponding nitrogen nucleophile, 80 °C, 84 h). Yield: 36% (164 mg, 709 µmol). Solvent system for flash column chromatography: petroleum ether/EtOAc: 60/40; Orange oil; ^{1}H NMR (400 MHz, $CDCl_3$): *δ* 7.46-7.41 (m, 2H), 7.31 (td, *J* = 7.6 and 1.3 Hz, 1H), 7.23 (td, *J* = 7.6 and 1.2 Hz, 1H), 4.62 (s, 2H), 4.52-4.47 (m, 2H), 4.05-3.99 (m, 2H), 3.45 (s, 3H); ^{13}C NMR (100 MHz, $CDCl_3$): *δ* 155.9, 139.8, 131.9, 128.4, 127.8, 127.5, 121.1, 83.4, 72.8, 69.2, 63.2, 58.7, 47.1; IR (neat): ν_{max} 2982, 2922, 2824, 2255, 1770, 1478, 1411, 1200, 1168, 1114, 1094, 1033, 759, 748, 628 cm^{-1}; ESIHRMS *m/z* calcd.. for $C_{13}H_{17}N_2O_3$ $[M+NH_4]^+$ 249.1234, found 249.1245.

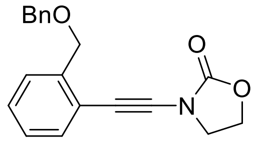

1h

3-{{2-[(Benzyloxy)methyl]phenyl}ethynyl}oxazolidin-2-one 1h. Prepared according to general procedure III (1.60 mmol of the corresponding nitrogen nucleophile, 80 °C, 16 h). Yield: 45% (219 mg, 713 µmol). Solvent system for flash column chromatography: petroleum ether/EtOAc: 70/30; Orange oil; ^{1}H NMR (400 MHz, $CDCl_3$): *δ* 7.51 (d, *J* = 7.6 Hz, 1H), 7.45-7.38 (m, 3H), 7.38-7.32 (m, 3H), 7.32-7.27 (m, 1H), 7.27-7.21 (m, 1H), 4.73 (s, 2H), 4.63 (s, 2H), 4.50-4.44 (m, 2H), 3.94-3.88 (m, 2H); ^{13}C NMR (100 MHz, $CDCl_3$): *δ* 155.8, 140.0, 138.6, 132.0, 128.5 (2C), 128.1, 128.0, 127.7, 127.5, 121.2, 83.4, 72.7, 70.5, 69.3, 63.1, 47.1.

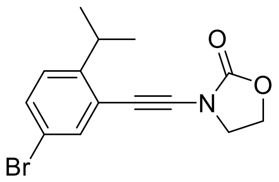

1i

3-[(5-Bromo-2-isopropylphenyl)ethynyl]oxazolidin-2-one 1i. Prepared according to general procedure III (2.00 mmol of the corresponding nitrogen nucleophile, 80 °C, 48 h). Yield: 50% (309 mg, 999 µmol). Solvent system for flash column chromatography: petroleum ether/EtOAc: 80/20; Orange oil; ^{1}H NMR (400 MHz, $CDCl_3$): *δ* 7.53 (d, *J* = 2.2 Hz, 1H), 7.37 (dd, *J* = 8.4 and 2.2 Hz, 1H), 7.12 (d, *J* = 8.4 Hz, 1H), 4.53-4.48 (m, 2H), 4.04-3.99 (m, 2H), 3.36 (sept, *J* = 6.9 Hz, 1H), 1.23 (d, *J* = 6.9 Hz, 6H); ^{13}C NMR (100 MHz, $CDCl_3$): *δ* 155.8, 149.3, 134.5, 131.6, 126.8, 123.2, 118.8, 83.7, 69.0, 63.2, 47.1, 31.4, 23.1; IR (neat): ν_{max} 2960, 2925, 2871, 2254, 1756, 1471, 1426, 1391, 1323, 1216, 1090, 1030, 870, 813, 745, 643 cm^{-1}; ESIHRMS *m/z* calcd.. for $C_{14}H_{15}{}^{81}BrNO_2$ $[M+H]^+$ 310.0260, found 310.0248.

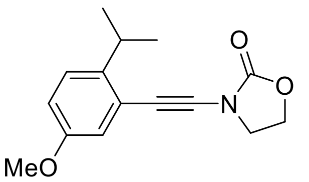

1j

3-[(2-Isopropyl-5-methoxyphenyl)ethynyl]oxazolidin-2-one 1j. Prepared according to general procedure III (701 µmol of the corresponding nitrogen nucleophile, 80 °C, 60 h). Yield: 32% (58 mg, 224 µmol). Solvent system for flash column chromatography: petroleum ether/EtOAc: 80/20; Yellow solid; Mp: 48 °C; ^{1}H NMR (400 MHz, $CDCl_3$): δ 7.16 (d, J = 8.6 Hz, 1H), 6.94 (d, J = 2.8 Hz, 1H), 6.85 (dd, J = 8.6 and 2.8 Hz, 1H), 4.52-4.48 (m, 2H), 4.05-4.00 (m, 2H), 3.77 (s, 3H), 3.35 (sept, J = 6.9 Hz, 1H), 1.23 (d, J = 6.9 Hz, 6H); ^{13}C NMR (100 MHz, $CDCl_3$): δ 157.2, 155.9, 142.8, 126.1, 121.7, 116.5, 115.5, 82.3, 70.2, 63.1, 55.5, 47.2, 31.0, 23.4; IR (neat): ν_{max} 2965, 2932, 2362, 2258, 1761, 1600, 1429, 1395, 1208, 1192, 1097, 1026, 752 cm^{-1}; ESIHRMS *m/z* calcd.. for $C_{15}H_{18}NO_3$ $[M+H]^+$ 260.1281, found 260.1284.

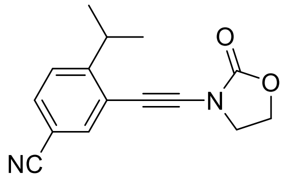

1k

3-[(5-Cyano-2-isopropylphenyl)ethynyl]oxazolidin-2-one 1k. Prepared according to general procedure III (1.33 mmol of the corresponding nitrogen nucleophile, 80 °C, 60 h). Yield: 28% (95 mg, 374 µmol). Solvent system for flash column chromatography: petroleum ether/EtOAc: 70/30; Orange solid; Mp: 89 °C; ^{1}H NMR (400 MHz, $CDCl_3$): δ 7.66 (d, J = 1.8 Hz, 1H), 7.53 (dd, J = 8.2 and 1.8 Hz, 1H), 7.36 (d, J = 8.2 Hz, 1H), 4.55-4.50 (m, 2H), 4.07-4.01 (m, 2H), 3.46 (sept, J = 6.9 Hz, 1H), 1.26 (d, J = 6.9 Hz, 6H); ^{13}C NMR (100 MHz, $CDCl_3$): δ 155.7, 155.4, 135.3, 131.7, 126.2, 122.9, 118.5, 109.9, 84.7, 68.4, 63.3, 46.9, 32.1, 22.8; IR (neat): ν_{max} 2964, 2926, 2259, 2233, 1775, 1750, 1471, 1437, 1394, 1222, 1031, 740 cm^{-1}; ESIHRMS *m/z* calcd.. for $C_{15}H_{15}N_2O_2$ $[M+H]^+$ 255.1128, found 255.1132.

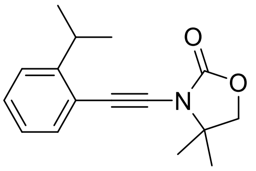

1l

3-[(2-Isopropylphenyl)ethynyl]-4,4-dimethyloxazolidin-2-one 1l. Prepared according to general procedure III (1.50 mmol of the corresponding nitrogen nucleophile, 80 °C, 48 h). Yield: 90% (349 mg, 1.36 mmol). Solvent system for flash column chromatography: petroleum ether/EtOAc: 85/15; Yellow oil; ^{1}H NMR (400 MHz, $CDCl_3$): δ 7.44 (dt, J = 7.7 and 1.0 Hz, 1H), 7.29-7.26 (m, 2H), 7.16-7.11 (m, 1H), 4.20 (s, 2H), 3.43 (sept, J = 6.9 Hz, 1H), 1.51 (s, 6H), 1.28 (d, J = 6.9 Hz, 6H); ^{13}C NMR (100 MHz, $CDCl_3$): δ 155.1, 150.1, 132.5, 128.6, 125.7, 125.0, 121.4, 80.0, 75.7, 72.3, 60.5, 31.8, 25.1, 23.2; IR (neat): ν_{max} 2967, 2931, 2870, 2246, 1771, 1464,

1448, 1403, 1283, 1271, 1180, 1072, 1023, 753 cm^{-1}; ESIHRMS *m/z* calcd. for $C_{16}H_{23}N_2O_2$ $[M+NH_4]^+$ 275.1754, found 275.1762.

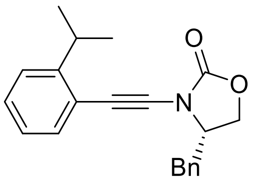

1m

(*S*)-4-Benzyl-3-[(2-isopropylphenyl)ethynyl]oxazolidin-2-one 1m. Prepared according to general procedure III (3.00 mmol of the corresponding nitrogen nucleophile, 80 °C, 48 h). Yield: 84% (808 mg, 2.53 mmol). Solvent system for flash column chromatography: petroleum ether/EtOAc: 80/20; White solid, Mp: 82 °C; $[\alpha]_D^{25}$ + 71 (*c* 2.0, $CHCl_3$); ^1H NMR (400 MHz, $CDCl_3$): δ 7.47-7.43 (m, 1H), 7.39-7.33 (m, 2H), 7.33-7.28 (m, 3H), 7.27-7.23 (m, 2H), 7.19-7.12 (m, 1H), 4.41-4.33 (m, 2H), 4.22-4.14 (m, 1H), 3.49 (sept, *J* = 6.9 Hz, 1H), 3.34-3.27 (m, 1H), 3.07-3.00 (m, 1H), 1.31 (d, *J* = 6.9 Hz, 3H), 1.30 (d, *J* = 6.9 Hz, 3H); ^{13}C NMR (100 MHz, $CDCl_3$): δ 155.5, 150.3, 134.3, 132.5, 129.5, 129.2, 128.7, 127.7, 125.7, 125.0, 121.1, 81.6, 72.2, 67.5, 58.7, 38.1, 31.8, 23.3, 23.2; IR (neat): ν_{max} 2962, 2934, 2248, 1756, 1415, 1217, 1191, 1100, 1066, 996, 764, 754, 705 cm^{-1}; ESIHRMS *m/z* calcd. for $C_{21}H_{22}NO_2$ $[M+H]^+$ 320.1645, found 320.1655.

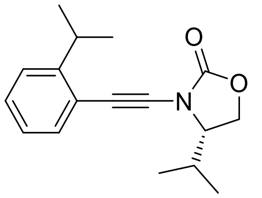

1n

(*S*)-4-Isopropyl-3-[(2-isopropylphenyl)ethynyl]oxazolidin-2-one 1n. Prepared according to general procedure III (3.00 mmol of the corresponding nitrogen nucleophile, 80 °C, 48 h). Yield: 73% (597 mg, 2.20 mmol). Solvent system for flash column chromatography: petroleum ether/EtOAc: 80/20; Yellow oil; $[\alpha]_D^{25}$ + 39 (*c* 1.0, $CHCl_3$); ^1H NMR (400 MHz, $CDCl_3$): δ 7.41 (d, *J* = 7.6 Hz, 1H), 7.30-7.24 (m, 2H), 7.16-7.08 (m, 1H), 4.43 (t, *J* = 8.9 Hz, 1H), 4.20 (dd, *J* = 9.0 and 5.9 Hz, 1H), 4.06 (ddd, *J* = 8.8, 5.8 and 4.1 Hz, 1H), 3.42 (sept, *J* = 6.9 Hz, 1H), 2.29 (septd, *J* = 7.0 and 4.1 Hz, 1H), 1.26 (d, *J* = 6.9 Hz, 6H), 1.04 (d, *J* = 6.8 Hz, 3H), 1.03 (d, *J* = 7.0 Hz, 3H); ^{13}C NMR (100 MHz, $CDCl_3$): δ 156.1, 150.3, 132.4, 128.6, 125.6, 125.0, 121.3, 82.0, 71.2, 64.9, 62.2, 31.7, 29.5, 23.2, 23.2, 17.4, 15.3; IR (neat): ν_{max} 2963, 2933, 2874, 2249, 1774, 1465, 1409, 1196, 1092, 1072, 980, 758, 715, 642 cm^{-1}; ESIHRMS *m/z* calcd. for $C_{17}H_{22}NO_2$ $[M+H]^+$ 272.1645, found 272.1650.

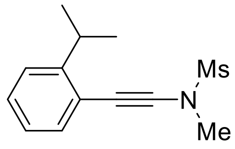

1o

***N*-Methyl-*N*-methanesulfonyl-(2-isopropylphenyl)ethynylamine 1o.** Prepared according to general procedure III (1.20 mmol of the corresponding nitrogen nucleophile, 80 °C, 24 h). Yield: 76% (234 mg, 931 µmol). Solvent system for flash column chromatography: petroleum ether/EtOAc: 85/15; Yellow oil; ^{1}H NMR (400 MHz, $CDCl_3$): *δ* 7.37 (dt, *J* = 7.7 and 1.0 Hz, 1H), 7.28-7.25 (m, 2H), 7.16-7.09 (m, 1H), 3.37 (sept, *J* = 6.9 Hz, 1H), 3.32 (s, 3H), 3.12 (s, 3H), 1.27 (d, *J* = 6.9 Hz, 6H); ^{13}C NMR (100 MHz, $CDCl_3$): *δ* 150.2, 132.1, 128.5, 125.7, 125.1, 121.3, 86.6, 68.5, 39.4, 36.9, 31.8, 23.2; IR (neat): ν_{max} 2962, 2933, 2872, 2235, 1462, 1447 1360, 1325, 1185, 1162, 959, 788, 760, 713, 641 cm^{-1}; ESIHRMS *m/z* calcd. for $C_{13}H_{17}KNO_2S$ $[M+K]^+$ 290.0612, found 290.0608.

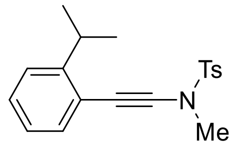

1p

***N*-Methyl-*N*-4-toluenesulfonyl-(2-isopropylphenyl)ethynylamine 1p.** Prepared according to general procedure III (1.20 mmol of the corresponding nitrogen nucleophile, 80 °C, 24 h). Yield: 94% (371 mg, 1.13 mmol). Solvent system for flash column chromatography: petroleum ether/EtOAc: 90/10; Orange oil; ^{1}H NMR (400 MHz, $CDCl_3$): *δ* 7.85 (d, *J* = 8.3 Hz, 2H), 7.37 (d, *J* = 8.0 Hz, 2H), 7.32 (dt, *J* = 7.6 and 0.9 Hz, 1H), 7.27-7.23 (m, 2H), 7.15-7.07 (m, 1H), 3.33 (sept, *J* = 6.9 Hz, 1H), 3.19 (s, 3H), 2.46 (s, 3H), 1.24 (d, *J* = 6.9 Hz, 6H); ^{13}C NMR (100 MHz, $CDCl_3$): *δ* 150.1, 144.9, 133.6, 132.1, 129.9, 128.3, 128.0, 125.6, 125.0, 121.6, 87.4, 67.9, 39.5, 31.6, 23.3, 21.8; IR (neat): ν_{max} 2961, 2931, 2871, 2233, 1598, 1486, 1447, 1367, 1187, 1168, 1089, 964, 913, 778, 758, 722, 675, 637 cm^{-1}; ESIHRMS *m/z* calcd. for $C_{19}H_{25}N_2O_2S$ $[M+NH_4]^+$ 345.1631, found 345.1646.

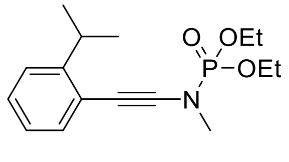

1q

***N*-Methyl-*N*-diethoxyphosphoryl-(2-isopropylphenyl)ethynylamine 1q.** Prepared according to general procedure IV (1.50 mmol of the corresponding nitrogen nucleophile, K_3PO_4, 95 °C, 48 h). Yield: 26% (122 mg, 394 µmol). Solvent system for flash column chromatography: petroleum ether/EtOAc: 70/30; Red oil; ^{1}H NMR (400 MHz, $CDCl_3$): *δ* 7.33-7.30 (m, 1H), 7.25-7.18 (m, 2H), 7.12-7.06 (m, 1H), 4.28-4.13 (m, 4H), 3.38 (sept, *J* = 6.9 Hz, 1H), 3.18 (d, *J* = 7.9 Hz, 3H), 1.39 (td, *J* = 7.1 and 1.0 Hz, 6H), 1.25 (d, *J* = 6.9 Hz, 6H); ^{13}C NMR (100 MHz, $CDCl_3$): *δ*

149.5, 131.6, 127.4, 125.6, 124.9, 122.7, 90.8 (d, $J_{C\text{-}P}$ = 4.6 Hz), 63.9 (d, $J_{C\text{-}P}$ = 5.8 Hz), 62.7 (d, $J_{C\text{-}P}$ = 5.1 Hz), 39.2 (d, $J_{C\text{-}P}$ = 5.3 Hz), 31.6, 23.2, 16.3 (d, $J_{C\text{-}P}$ = 7.1 Hz); ^{31}P NMR (121 MHz, $CDCl_3$): δ 4.5; IR (neat): ν_{max} 2962, 2237, 1464, 1446, 1356, 1338, 1269, 1057, 1022, 994, 802, 757, 722 cm^{-1}; ESIHRMS *m/z* calcd. for $C_{16}H_{25}NO_3P$ [M+H]$^+$ 310.1567, found 310.1573.

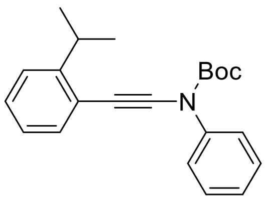

1r

***N*-(*tert*-Butoxycarbonyl)-*N*-phenyl-(2-isopropylphenyl)ethynylamine 1r.** Prepared according to general procedure IV (2.00 mmol of the corresponding nitrogen nucleophile, K_3PO_4, 80 °C, 48 h). Yield: 70% (472 mg, 1.41 mmol). Solvent system for flash column chromatography: petroleum ether/EtOAc: 98/2; Orange oil; ^{1}H NMR (400 MHz, $CDCl_3$): δ 7.60-7.55 (m, 2H), 7.44-7.38 (m, 2H), 7.38-7.35 (m, 1H), 7.30-7.22 (m, 3H), 7.15-7.09 (m, 1H), 3.45 (sept, *J* = 6.9 Hz, 1H), 1.60 (s, 9H), 1.26 (d, *J* = 6.9 Hz, 6H); ^{13}C NMR (100 MHz, $CDCl_3$): δ 153.0, 149.5, 140.0, 131.6, 128.9, 127.8, 126.7, 125.6, 125.0, 124.7, 122.3, 87.2, 83.6, 69.3, 31.6, 28.2, 23.3.

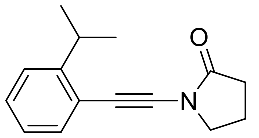

1s

1-[(2-Isopropylphenyl)ethynyl]pyrrolidin-2-one 1s. Prepared according to general procedure III (1.20 mmol of the corresponding nitrogen nucleophile, 80 °C, 48 h). Yield: 28% (75 mg, 330 µmol). Solvent system for flash column chromatography: petroleum ether/EtOAc: 75/25; Orange oil; ^{1}H NMR (400 MHz, $CDCl_3$): δ 7.41 (dt, *J* = 7.6 and 1.1 Hz, 1H), 7.27-7.24 (m, 2H), 7.15-7.07 (m, 1H), 3.80 (t, *J* = 7.2 Hz, 2H), 3.45 (sept, *J* = 6.9 Hz, 1H), 2.52-2.46 (m, 2H), 2.24-2.15 (m, 2H), 1.26 (d, *J* = 6.9 Hz, 6H); ^{13}C NMR (100 MHz, $CDCl_3$): δ 175.8, 150.3, 132.3, 128.4, 125.6, 125.0, 121.6, 84.1, 71.6, 50.2, 31.7, 29.8, 23.2, 19.0.

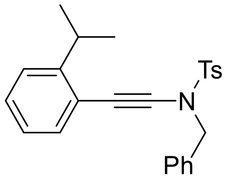

1t

***N*-Benzyl-*N*-4-toluenesulfonyl-(2-isopropylphenyl)ethynylamine 1t.** Prepared according to general procedure III (1.20 mmol of the corresponding nitrogen nucleophile, 80 °C, 48 h). Yield: 71% (343 mg, 850 µmol). Solvent system for flash column chromatography: petroleum ether/EtOAc: 90/10; Off-white solid, Mp: 98 °C; ^{1}H NMR (400 MHz, $CDCl_3$): δ 7.82 (d, *J* = 8.3 Hz, 2H), 7.37-7.30 (m, 7H), 7.23-7.16 (m, 3H), 7.09-7.03 (m, 1H), 4.61 (s, 2H), 3.02 (sept, *J* = 6.9

Hz, 1H), 2.45 (s, 3H), 1.09 (d, *J* = 6.9 Hz, 6H); ^{13}C NMR (100 MHz, $CDCl_3$): *δ* 149.9, 144.8, 134.9, 134.6, 131.9, 129.9, 129.0, 128.7, 128.5, 128.1, 127.9, 125.5, 124.9, 121.7, 85.9, 70.3, 55.8, 31.5, 23.2, 21.8; IR (neat): ν_{max} 2971, 2238, 1764, 1612, 1412, 1365, 1174, 946, 824, 787, 761, 732, 681, 656 cm^{-1}; ESIHRMS *m/z* calcd. for $C_{25}H_{25}NNaO_2S$ $[M+Na]^+$ 426.1498, found 426.1499.

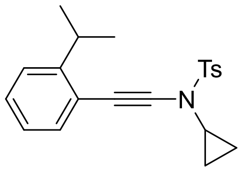

1u

***N*-Cyclopropyl-*N*-4-toluenesulfonyl-(2-isopropylphenyl)ethynylamine 1u.** Prepared according to general procedure III (1.20 mmol of the corresponding nitrogen nucleophile, 80 °C, 24 h). Yield: 82% (347 mg, 982 µmol). Solvent system for flash column chromatography: petroleum ether/EtOAc: 95/5; Yellow oil; ^{1}H NMR (400 MHz, $CDCl_3$): *δ* 7.88 (d, *J* = 8.3 Hz, 2H), 7.36 (d, *J* = 8.0 Hz, 2H), 7.33 (dt, *J* = 7.7 and 1.0 Hz, 1H), 7.26-7.23 (m, 2H), 7.15-7.08 (m, 1H), 3.32 (sept, *J* = 6.9 Hz, 1H), 2.88 (tt, *J* = 7.0 and 3.6 Hz, 1H), 2.46 (s, 3H), 1.24 (d, *J* = 6.9 Hz, 6H), 0.96-0.90 (m, 2H), 0.83-0.77 (m, 2H); ^{13}C NMR (100 MHz, $CDCl_3$): *δ* 150.0, 144.9, 134.2, 132.1, 129.8, 128.2, 128.2, 125.6, 125.0, 121.8, 85.3, 69.4, 33.2, 31.7, 23.3, 21.8, 6.5; IR (neat): ν_{max} 2962, 2931, 2871, 2233, 1598, 1486, 1447, 1370, 1174, 1091, 1031, 862, 814, 758, 720, 671, 628 cm^{-1}; ESIHRMS *m/z* calcd. for $C_{21}H_{24}NO_2S$ $[M+H]^+$ 354.1522, found 354.1499.

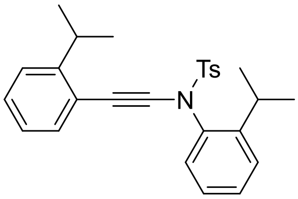

1v

***N*-(2-Isopropylphenyl)-*N*-4-toluenesulfonyl-(2-isopropylphenyl)ethynylamine 1v.** Prepared according to general procedure IV (2.00 mmol of the corresponding nitrogen nucleophile, K_2CO_3, 80 °C, 48 h). Yield: 23% (198 mg, 458 µmol). Solvent system for flash column chromatography: petroleum ether/EtOAc: 95/5; Orange oil; ^{1}H NMR (400 MHz, $CDCl_3$): *δ* 7.79 (d, *J* = 8.3 Hz, 2H), 7.42-7.33 (m, 4H), 7.29 (dt, *J* = 7.7 and 1.0 Hz, 1H), 7.25-7.22 (m, 2H), 7.14-7.06 (m, 2H), 6.88 (dd, *J* = 8.0 and 1.3 Hz, 1H), 3.52 (sept, *J* = 6.9 Hz, 1H), 3.32 (sept, *J* = 6.9 Hz, 1H); 2.48 (s, 3H), 1.25 (d, *J* = 6.9 Hz, 6H), 1.20 (d, *J* = 6.9 Hz, 6H); ^{13}C NMR (100 MHz, $CDCl_3$): *δ* 150.3, 148.6, 145.0, 136.7, 134.6, 132.2, 129.9, 129.8, 128.6, 128.3, 128.0, 127.4, 126.6, 125.6, 125.0, 121.8, 87.5, 67.9, 31.7, 28.4, 23.3 (2C), 21.9; IR (neat): ν_{max} 2965, 2928, 2870, 2235, 1597, 1486, 1447, 1372, 1187, 1172, 1090, 1058, 1031, 923, 802, 757, 712, 675, 651 cm^{-1}; ESIHRMS *m/z* calcd. for $C_{27}H_{33}N_2O_2S$ $[M+NH_4]^+$ 449.2257, found 449.2255.

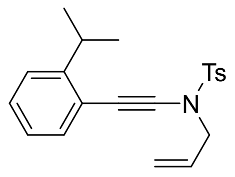

1w

***N*-Allyl-*N*-4-toluenesulfonyl-(2-isopropylphenyl)ethynylamine 1w.** Prepared according to general procedure III (1.50 mmol of the corresponding nitrogen nucleophile, 80 °C, 48 h). Yield: 46% (245 mg, 693 µmol). Solvent system for flash column chromatography: petroleum ether/EtOAc: 95/5; Yellow oil; ^{1}H NMR (400 MHz, $CDCl_3$): δ 7.85 (d, J = 8.3 Hz, 2H), 7.35 (d, J = 8.2 Hz, 2H), 7.31 (app. d, J = 7.6 Hz, 1H), 7.25-7.22 (m, 2H), 7.13-7.07 (m, 1H), 5.81 (ddt, J = 16.6, 10.1 and 6.4 Hz, 1H), 5.33-5.22 (m, 2H), 4.09 (dt, J = 6.4 and 1.3 Hz, 2H), 3.30 (sept, J = 6.9 Hz, 1H), 2.45 (s, 3H), 1.21 (d, J = 6.9 Hz, 6H); ^{13}C NMR (100 MHz, $CDCl_3$): δ 150.0, 144.8, 135.0, 132.1, 131.2, 129.9, 128.2, 127.9, 125.6, 124.9, 121.7, 120.2, 85.8, 69.8, 54.6, 31.6, 23.3, 21.8; IR (neat): ν_{max} 2963, 2928, 2869, 2233, 1597, 1366, 1170, 1090, 1032, 924, 813, 783, 758, 663, 610 cm^{-1}; ESIHRMS *m/z* calcd. for $C_{21}H_{27}N_2O_2S$ $[M+NH_4]^+$ 371.1788, found 371.1790.

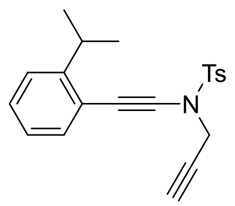

1x

***N*-Propargyl-*N*-4-toluenesulfonyl-(2-isopropylphenyl)ethynylamine 1x.** An oven-dried 25 mL round bottom flask was charged with **1w** (400 mg, 859 µmol) and fitted with a rubber septum before being evacuated under high vacuum and backfilled with argon three times. Freshly distilled tetrahydrofuran (5 mL) was next added and the resulting solution was cooled to 0 °C before a 1 M solution of tetrabutylammonium fluoride in tetrahydrofuran (1.29 mL, 1.29 mmol) was added dropwise. The reaction mixture was stirred at 0 °C for 1 hour before being quenched by addition of a saturated aqueous solution of NH_4Cl (10 mL). The layers were separated and the aqueous layer was extracted with diethyl ether thrice (3x10 mL). The combined organic layers were then washed with brine (30 mL), dried over $MgSO_4$, filtered and concentrated under reduced pressure. The resulting crude residue was finally purified by flash column chromatography over silica gel to afford the desired unprotected ynamide. Yield: 49% (149 mg, 424 µmol). Solvent system for flash column chromatography: petroleum ether/EtOAc: 90/10; Beige solid; ^{1}H NMR (400 MHz, $CDCl_3$): δ 7.88 (d, J = 8.4 Hz, 2H), 7.38-7.32 (m, 3H), 7.29-7.23 (m, 2H), 7.14-7.09 (m, 1H), 4.37 (d, J = 2.5 Hz, 2H), 3.33 (sept, J = 6.9 Hz, 1H), 2.45 (s, 3H), 2.21 (t, J = 2.5 Hz, 1H), 1.20 (d, J = 6.9 Hz, 6H); ^{13}C NMR (100 MHz, $CDCl_3$): δ 150.4, 145.1, 134.4, 132.4, 129.8, 128.5, 128.4, 125.6, 125.0, 121.4, 84.9, 76.1, 74.7, 70.2, 42.0, 31.6, 23.3, 21.8.

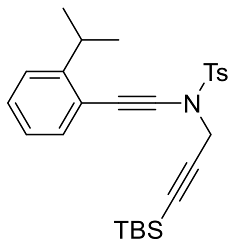

1y

***N*-[3-(*tert*-Butyldimethylsilyl)prop-2-yn-1-yl]-*N*-4-toluenesulfonyl-(2-isopropylphenyl)ethynylamine 1y.** Prepared according to general procedure III (1.50 mmol of the corresponding nitrogen nucleophile, 80 °C, 16 h). Yield: 64% (447 mg, 960 µmol). Solvent system for flash column chromatography: petroleum ether/EtOAc: 95/5; Yellow oil; ^{1}H NMR (400 MHz, $CDCl_3$): δ 7.88 (d, J = 8.4 Hz, 2H), 7.36-7.30 (m, 3H), 7.27-7.23 (m, 2H), 7.10 (ddd, J = 7.6, 5.9 and 2.8 Hz, 1H), 4.41 (s, 2H), 3.34 (sept, J = 6.9 Hz, 1H), 2.44 (s, 3H), 1.22 (d, J = 6.9 Hz, 6H), 0.80 (s, 9H), -0.03 (s, 6H); ^{13}C NMR (100 MHz, $CDCl_3$): δ 150.4; 144.8; 134.7; 132.6; 129.8; 128.4; 128.3; 125.5; 124.9; 121.6; 98.0; 90.2; 85.1; 70.0, 42.8, 31.6, 26.0, 23.3, 21.8, 16.4, -4.8; IR (neat): ν_{max} 2958, 2929, 2857, 2233, 1598, 1470, 1371, 1251, 1171, 1091, 1046, 1003, 923, 839, 826, 812, 778, 732, 662, 604 cm^{-1}; ESIHRMS m/z calcd. for $C_{27}H_{36}NO_2SSi$ $[M+H]^+$ 466.2231, found 466.2226.

Experimental Procedures and Characterization Data
Intramolecular hydroalkylation of ynamides: 2-aminoindenes

General procedure V: gold-catalyzed intramolecular hydroalkylation of ynamides
An oven-dried 5 mL round bottom flask was charged with the ynamide (500 µmol) and $IPrAuNTf_2$ (22 mg, 25 µmol) and fitted with a rubber septum before being evacuated under high vacuum and backfilled with argon three times. Freshly distilled dichloromethane (3 mL) was next added and the resulting mixture was stirred at room temperature for 20 h. The reaction mixture was then concentrated under reduced pressure and the resulting crude residue was finally purified by flash column chromatography over silica gel to afford the desired 2-aminoindene.

General procedure VI: gold-catalyzed intramolecular hydroalkylation of ynamides
An oven-dried 5 mL round bottom flask was charged with the ynamide (500 µmol) and fitted with a rubber septum before being evacuated under high vacuum and backfilled with argon three times. Freshly distilled dichloromethane (3 mL) and $IPrAuNTf_2$ (22 mg, 25 µmol) were next added and the resulting mixture was stirred at room temperature for 20 h. The reaction mixture was then concentrated under reduced pressure and the resulting crude residue was finally purified by flash column chromatography over silica gel to afford the desired 2-aminoindene.

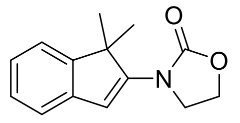

4a

2-(2-Oxooxazolidin-3-yl)-1,1-dimethyl-1*H*-indene 4a. Prepared according to general procedure V (500 µmol of the corresponding ynamide). Yield: 77% (88 mg, 384 µmol). Solvent system for flash column chromatography: petroleum ether/EtOAc: 80/20; Pale yellow solid, Mp: 104 °C; ^{1}H NMR (400 MHz, $CDCl_3$): δ 7.25-7.12 (m, 4H), 6.34 (s, 1H), 4.50-4.45 (m, 2H), 4.07-4.01 (m, 2H), 1.56 (s, 6H); ^{13}C NMR (100 MHz, $CDCl_3$): δ 154.9, 151.3, 151.0, 140.0, 126.9, 124.8, 120.9, 120.5, 114.2, 61.8, 49.8, 46.8, 24.0; IR (neat): ν_{max} 2962, 2926, 2869, 1747, 1564, 1470, 1403, 1343, 1263, 1212, 1114, 1052, 745, 644 cm^{-1}; ESIHRMS *m/z* calcd. for $C_{14}H_{16}NO_2$ $[M+H]^+$ 230.1176, found 230.1177.

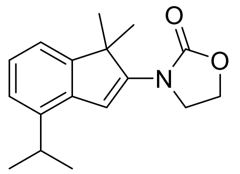

4b

2-(2-Oxooxazolidin-3-yl)-4-isopropyl-1,1-dimethyl-1*H*-indene 4b. Prepared according to general procedure V (500 µmol of the corresponding ynamide). Yield: 96% (130 mg, 479 µmol). Solvent system for flash column chromatography: petroleum ether/EtOAc: 80/20; Pink oil; ^{1}H NMR (400 MHz, $CDCl_3$): δ 7.16-7.05 (m, 3H), 6.54 (s, 1H), 4.51-4.45 (m, 2H), 4.09-4.04 (m, 2H), 3.16 (sept, *J* = 7.0 Hz, 1H), 1.54 (s, 6H), 1.28 (d, *J* = 6.9 Hz, 6H); ^{13}C NMR (100 MHz, $CDCl_3$): δ 155.1, 151.0, 150.6, 140.6, 137.5, 125.2, 123.2, 118.4, 112.7, 61.8, 49.9, 46.8, 30.6, 24.2, 23.5; IR (neat): ν_{max} 2964, 2927, 2870, 1749, 1564, 1479, 1406, 1250, 1209, 1100, 1055, 793, 749 cm^{-1}; ESIHRMS *m/z* calcd. for $C_{17}H_{22}NO_2$ $[M+H]^+$ 272.1645, found 272.1645.

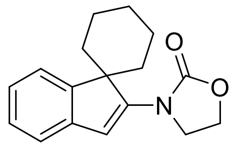

4c

2'-(2-Oxooxazolidin-3-yl)spiro(cyclohexane)-1,1'-indene 4c. Prepared according to general procedure V (223 µmol of the corresponding ynamide). Yield: 73% (44 mg, 163 µmol). Solvent system for flash column chromatography: petroleum ether/EtOAc: 75/25; Off-white solid, Mp: 135 °C; ^{1}H NMR (400 MHz, $CDCl_3$): δ 7.73 (d, *J* = 7.5 Hz, 1H), 7.29-7.21 (m, 2H), 7.14 (td, *J* = 7.3 and 1.7 Hz, 1H), 6.52 (s, 1H), 4.50-4.44 (m, 2H), 4.04-3.98 (m, 2H), 2.20 (td, *J* = 13.1 and 4.5 Hz, 2H), 2.04-1.87 (m, 3H), 1.83-1.74 (m, 2H), 1.58-1.35 (m, 3H); ^{13}C NMR (100 MHz, $CDCl_3$): δ 156.3, 151.8, 150.3, 140.3, 126.8, 124.5, 124.2, 121.4, 120.9, 62.0, 53.7, 48.6, 30.7, 24.9, 22.3; IR (neat): ν_{max} 2927, 2907, 2854, 1749, 1565, 1478, 1454, 1402, 1265, 1220, 1181, 1120, 1046, 812, 742 cm^{-1}; ESIHRMS *m/z* calcd. for $C_{17}H_{20}NO_2$ $[M+H]^+$ 270.1489, found 270.1489.

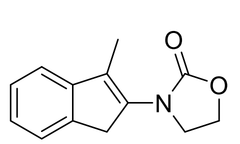

4d

2-(2-Oxooxazolidin-3-yl)-1-methyl-3*H*-indene 4d. Prepared according to general procedure V (500 µmol of the corresponding ynamide). Yield: 40% (43 mg, 200 µmol). Solvent system for flash column chromatography: petroleum ether/EtOAc: 80/20; Pale green solid, Mp: 117 °C; ^{1}H NMR (400 MHz, $CDCl_3$): δ 7.37 (d, *J* = 7.3 Hz, 1H), 7.33-7.27 (m, 2H), 7.23-7.16 (m, 1H), 4.52-4.46 (m, 2H), 4.05-3.99 (m, 2H), 3.63 (app. q, *J* = 1.9 Hz, 2H), 2.13 (t, *J* = 2.0 Hz, 3H); ^{13}C NMR (100 MHz, $CDCl_3$): δ 156.1, 145.3, 139.4, 135.7, 128.8, 126.6, 125.0, 123.4, 118.9, 62.4, 46.9, 37.4, 10.9; IR (neat): ν_{max} 2925, 2868, 1740, 1471, 1403, 1359, 1225, 1205, 1115, 1086, 1035, 752, 717 cm^{-1}; ESIHRMS *m/z* calcd. for $C_{13}H_{17}N_2O_2$ $[M+NH_4]^+$ 233.1285, found 233.1286.

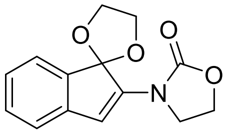

4f

2'-(2-Oxooxazolidin-3-yl)spiro(1,3-dioxolane)-2,1'-indene 4f. Prepared according to general procedure V (500 µmol of the corresponding ynamide). Yield: 66% (85 mg, 328 µmol). Solvent system for flash column chromatography: petroleum ether/EtOAc: 20/80; White solid, Mp: 176 °C; ^{1}H NMR (400 MHz, $CDCl_3$): δ 7.21-7.16 (m, 1H), 7.16-7.12 (m, 1H), 7.06-7.00 (m, 2H), 6.96 (s, 1H), 4.51-4.44 (m, 2H), 4.43-4.37 (m, 2H), 4.36-4.26 (m, 2H), 4.13-4.07 (m, 2H); ^{13}C NMR (100 MHz, $CDCl_3$): δ 155.8, 140.8, 140.4, 137.7, 130.1, 125.6, 121.4, 121.1, 117.0, 113.2, 64.8, 62.6, 43.2; IR (neat): ν_{max} 2973, 2928, 2914, 1747, 1610, 1484, 1410, 1216, 1181, 1112, 1012, 991, 851, 742 cm^{-1}; ESIHRMS *m/z* calcd. for $C_{14}H_{14}NO_4$ $[M+H]^+$ 260.0917, found 260.0922.

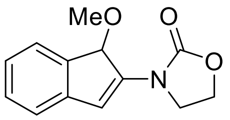

4g

2-(2-Oxooxazolidin-3-yl)-1-methoxy-1*H*-indene 4g. Prepared according to general procedure V (500 µmol of the corresponding ynamide). Yield: 63% (73 mg, 316 µmol). Solvent system for flash column chromatography: petroleum ether/EtOAc: 60/40; Pale yellow solid, Mp: 86 °C; ^{1}H NMR (400 MHz, $CDCl_3$): δ 7.38 (app. d, *J* = 7.3 Hz, 1H), 7.25 (td, *J* = 7.5 and 1.0 Hz, 1H), 7.15 (app. d, *J* = 7.4 Hz, 1H), 7.11 (td, *J* = 7.4 and 1.1 Hz, 1H), 6.66 (s, 1H), 5.38 (s, 1H), 4.51-4.45 (m, 2H), 4.28 (td, *J* = 7.2 and 7.4 Hz, 1H), 3.95 (td, *J* = 8.8 and 6.8 Hz, 1H), 3.15 (s, 3H); ^{13}C NMR (100 MHz, $CDCl_3$): δ 155.0, 143.0, 141.0, 137.6, 129.2, 124.8, 123.8, 120.8, 114.0, 81.4, 62.4, 52.6, 44.8; IR (neat): ν_{max} 3000, 2932, 2823, 1741, 1604, 1412, 1256, 1218, 1197, 1119, 1100, 1073, 749 cm^{-1}; ESIHRMS *m/z* calcd. for $C_{13}H_{14}NO_3$ $[M+H]^+$ 232.0968, found 232.0972.

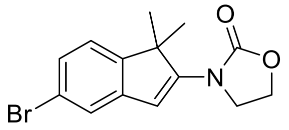

4i

2-(2-Oxooxazolidin-3-yl)-5-bromo-1,1-dimethyl-1*H*-indene 4i. Prepared according to general procedure VI (500 µmol of the corresponding ynamide). Yield: 83% (128 mg, 415 µmol). Solvent system for flash column chromatography: petroleum ether/EtOAc: 80/20; Off-white solid, Mp: 138 °C; ^{1}H NMR (400 MHz, $CDCl_3$): δ 7.30 (d, *J* = 1.8 Hz, 1H), 7.25 (dd, *J* = 8.0 and 1.9 Hz, 1H), 7.07 (d, *J* = 7.9 Hz, 1H), 6.23 (s, 1H), 4.51-4.45 (m, 2H), 4.05-4.00 (m, 2H), 1.54 (s, 6H); ^{13}C NMR (100 MHz, $CDCl_3$): δ 154.6, 152.7, 149.7, 142.1, 127.3, 123.3, 122.4, 120.6, 112.3, 61.8, 49.7, 46.6, 23.7; IR (neat): ν_{max} 2962, 2905, 2859, 1784, 1751, 1699, 1582, 1557, 1471, 1421, 1396, 1322, 1231, 1210, 1123, 1109, 1050, 996, 879, 806, 744, 704, 629 cm^{-1}; ESIHRMS *m/z* calcd. for $C_{14}H_{15}{}^{79}BrNO_2$ $[M+H]^+$ 308.0281, found 308.0278.

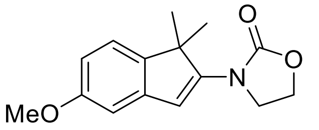

4j

2-(2-Oxooxazolidin-3-yl)-5-methoxy-1,1-dimethyl-1*H*-indene 4j. Prepared according to general procedure VI (200 µmol of the corresponding ynamide). Yield: 85% (44 mg, 170 µmol). Solvent system for flash column chromatography: petroleum ether/EtOAc: 80/20; Off-white solid, Mp: 155 °C; ^{1}H NMR (400 MHz, $CDCl_3$): δ 7.11 (d, J = 8.2 Hz, 1H), 6.77 (d, J = 2.4 Hz, 1H), 6.68 (dd, J = 8.2 and 2.4 Hz, 1H), 6.32 (s, 1H), 4.49-4.4 (m, 2H), 4.06-4.01 (m, 2H), 3.80 (s, 3H), 1.52 (s, 6H); ^{13}C NMR (100 MHz, $CDCl_3$): δ 159.2, 154.9, 152.2, 143.2, 141.3, 121.3, 114.0, 110.0, 106.5, 61.8, 55.6, 49.2, 46.6, 24.2; IR (neat): ν_{max} 2987, 2920, 2328, 1739, 1569, 1472, 1408, 1300, 1215, 1150, 1111, 857, 798, 623 cm^{-1}; ESIHRMS *m/z* calcd. for $C_{15}H_{18}NO_3$ $[M+H]^+$ 260.1281, found 260.1284.

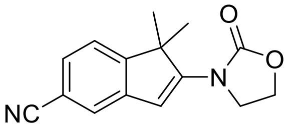

4k

2-(2-Oxooxazolidin-3-yl)-5-cyano-1,1-dimethyl-1*H*-indene 4k. Prepared according to general procedure VI (300 µmol of the corresponding ynamide). Yield: 73% (56 mg, 220 µmol). Solvent system for flash column chromatography: petroleum ether/EtOAc: 70/30; White solid, Mp: 134 °C; ^{1}H NMR (400 MHz, $CDCl_3$): δ 7.41-7.38 (m, 2H), 7.27 (d, J = 7.6 Hz, 1H), 6.22 (s, 1H), 4.52-4.47 (m, 2H), 4.07-4.02 (m, 2H), 1.56 (s, 6H); ^{13}C NMR (100 MHz, $CDCl_3$): δ 155.7, 154.4, 153.4, 141.0, 128.8, 123.2, 121.5, 119.6, 111.2, 110.5, 61.9, 50.3, 46.5, 23.2; IR (neat): ν_{max} 2970, 2924, 2373, 2229, 1758, 1561, 1474, 1398, 1245, 1110, 832, 743, 625 cm^{-1}; ESIHRMS *m/z* calcd. for $C_{15}H_{15}N_2O_2$ $[M+H]^+$ 255.1128, found 255.1130.

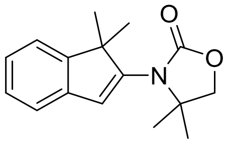

4l

2-(2-Oxo-4,4-dimethyloxazolidin-3-yl)-1,1-dimethyl-1*H*-indene 4l. Prepared according to general procedure VI (500 µmol of the corresponding ynamide). Yield: 85% (109 mg, 424 µmol). Solvent system for flash column chromatography: petroleum ether/EtOAc: 90/10; Pale yellow oil; ^{1}H NMR (400 MHz, $CDCl_3$): δ 7.34-7.28 (m, 2H), 7.26-7.22 (m, 2H), 6.62 (s, 1H), 4.15 (s, 2H), 1.42 (s, 6H), 1.38 (s, 6H); ^{13}C NMR (100 MHz, $CDCl_3$): δ 156.5, 152.1, 149.6, 139.2, 126.8 (2C), 126.0, 121.7, 121.4, 76.0, 61.8, 51.1, 26.1, 25.2; IR (neat): ν_{max} 2971, 2930, 1750, 1600, 1574, 1468, 1397, 1378, 1335, 1292, 1261, 1182, 1149, 1065, 1028, 916, 755, 663 cm^{-1}; ESIHRMS *m/z* calcd. for $C_{16}H_{23}N_2O_2$ $[M+NH_4]^+$ 275.1754, found 275.1760.

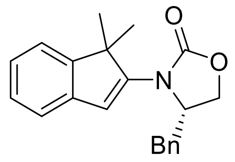

4m

(*S*)-2-(2-Oxo-4-benzyloxazolidin-3-yl)-1,1-dimethyl-1*H*-indene 4m. Prepared according to general procedure VI (500 µmol of the corresponding ynamide). Yield: 88% (140 mg, 438 µmol). Solvent system for flash column chromatography: petroleum ether/EtOAc: 80/20; Yellow oil; $[\alpha]_D^{25}$ - 79 (*c* 1.5, $CHCl_3$); ^1H NMR (400 MHz, $CDCl_3$): *δ* 7.37-7.26 (m, 5H), 7.26-7.21 (m, 2H), 7.21-7.16 (m, 2H), 6.66 (s, 1H), 4.46 (ddt, *J* = 10.1, 7.7 and 3.4 Hz, 1H), 4.31 (t, *J* = 8.2 Hz, 1H), 4.23 (dd, *J* = 8.9 and 3.4 Hz, 1H), 3.21 (dd, *J* = 13.7 and 3.3 Hz, 1H), 2.78 (dd, *J* = 13.7 and 10.2 Hz, 1H), 1.63 (s, 3H), 1.47 (s, 3H); ^{13}C NMR (100 MHz, $CDCl_3$): *δ* 155.5, 151.3, 149.0, 139.7, 135.5, 129.3, 129.2, 127.5, 127.0, 125.4, 121.3, 121.2, 121.1, 66.3, 58.4, 50.3, 38.1, 24.8, 24.2; IR (neat): ν_{max} 2964, 2926, 2868, 2250, 1756, 1603, 1565, 1470, 1399, 1231, 1209, 1105, 1017, 910, 731, 702, 647 cm^{-1}; ESIHRMS *m/z* calcd. for $C_{21}H_{22}NO_2$ $[M+H]^+$ 320.1645, found 320.1650.

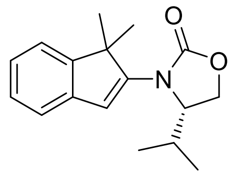

4n

(*S*)-2-(2-Oxo-4-isopropyloxazolidin-3-yl)-1,1-dimethyl-1*H*-indene 4n. Prepared according to general procedure VI (500 µmol of the corresponding ynamide). Yield: 91% (124 mg, 457 µmol). Solvent system for flash column chromatography: petroleum ether/EtOAc: 80/20; White solid, Mp: 149 °C; $[\alpha]_D^{25}$ - 148 (*c* 2.0, $CHCl_3$); ^1H NMR (400 MHz, $CDCl_3$): *δ* 7.28-7.24 (m, 2H), 7.24-7.16 (m, 2H), 6.59 (s, 1H), 4.39 (t, *J* = 8.8 Hz, 1H), 4.26 (dd, *J* = 9.0 and 3.1 Hz, 1H), 4.21 (dt, *J* = 8.7 and 3.1 Hz, 1H), 2.19 (septd, *J* = 7.0 and 3.1 Hz, 1H), 1.51 (s, 3H), 1.41 (s, 3H), 0.95 (d, *J* = 6.8 Hz, 3H), 0.92 (d, *J* = 7.2 Hz, 3H); ^{13}C NMR (100 MHz, $CDCl_3$): *δ* 156.0, 151.2, 148.8, 139.8, 127.0, 125.3, 121.9, 121.2, 121.0, 62.6, 61.0, 50.3, 28.3, 25.0, 24.2, 18.1, 14.0; IR (neat): ν_{max} 2959, 1754, 1598, 1574, 1471, 1405, 1385, 1205, 1105, 1058, 853, 759, 664 cm^{-1}; ESIHRMS *m/z* calcd. for $C_{17}H_{22}NO_2$ $[M+H]^+$ 272.1645, found 272.1646.

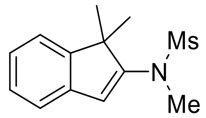

4o

2-(*N*-Methyl-*N*-methylsulfonylamino)-1,1-dimethyl-1*H*-indene 4o. Prepared according to general procedure VI (500 µmol of the corresponding ynamide). Yield: 65% (81 mg, 322 µmol). Solvent system for flash column chromatography: petroleum ether/EtOAc: 80/20; White solid, Mp: 92 °C; ^1H NMR (400 MHz, $CDCl_3$): *δ* 7.33-7.27 (m, 2H), 7.26-7.22 (m, 2H), 6.73 (s, 1H), 3.24

(s, 3H), 3.05 (s, 3H), 1.39 (s, 6H); ^{13}C NMR (100 MHz, CDCl$_3$): δ 155.3, 151.4, 139.2, 127.0, 126.2, 125.0, 121.9, 121.5, 50.9, 39.4, 37.1, 24.6; IR (neat): ν_{max} 2968, 2933, 2867, 1463, 1337, 1162, 1148, 1123, 1029, 1015, 955, 896, 838, 772, 735, 669, 610 cm^{-1}; ESIHRMS *m/z* calcd. for $C_{13}H_{21}N_2O_2S$ [M+NH$_4$]$^+$ 269.1318, found 269.1312.

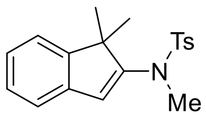

4p

2-(*N*-Methyl-*N*-4-toluenesulfonylamino)-1,1-dimethyl-1*H*-indene 4p. Prepared according to general procedure V (500 µmol of the corresponding ynamide). Yield: 62% (101 mg, 309 µmol). Solvent system for flash column chromatography: petroleum ether/EtOAc: 90/10; Pale yellow oil; ^{1}H NMR (400 MHz, CDCl$_3$): δ 7.77 (d, J = 8.3 Hz, 2H), 7.34 (d, J = 8.1 Hz, 2H); 7.29-7.19 (m, 4H), 6.23 (s, 1H), 3.14 (s, 3H), 2.47 (s, 3H), 1.35 (s, 6H); ^{13}C NMR (100 MHz, CDCl$_3$): δ 155.9, 151.6, 143.9, 139.4, 135.2, 129.6, 128.6, 126.8, 126.1, 125.6, 121.8, 121.5, 50.9, 40.5, 24.4, 21.7; IR (neat): ν_{max} 2966, 2927, 2865, 1598, 1468, 1456, 1348, 1164, 1088, 1029, 1014, 885, 871, 838, 815, 749, 700, 662, 616 cm^{-1}; ESIHRMS *m/z* calcd. for $C_{19}H_{25}N_2O_2S$ [M+NH$_4$]$^+$ 345.1631, found 345.1625.

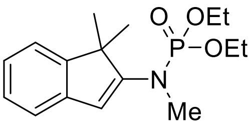

4q

2-(*N*-Methyl-*N*-diethoxyphosphorylamino)-1,1-dimethyl-1*H*-indene 4q. Prepared according to general procedure V (250 µmol of the corresponding ynamide). For this compound, the reaction was performed in 3.0 mL of dichloromethane instead of 1.5 mL. Yield: 66% (51 mg, 165 µmol). Solvent system for flash column chromatography: petroleum ether/EtOAc: 50/50; Orange oil; ^{1}H NMR (400 MHz, CDCl$_3$): δ 7.24-7.11 (m, 4H), 6.44 (d, J = 1.7 Hz, 1H), 4.20-4.02 (m, 4H), 3.12 (d, J = 9.6 Hz, 3H), 1.41 (s, 6H), 1.33 (td, J = 7.1 and 0.8 Hz, 6H); ^{13}C NMR (100 MHz, CDCl$_3$): δ 156.8, 151.3, 140.6, 126.7, 124.7, 121.0, 120.7, 119.7, 63.0 (d, J_{C-P} = 5.7 Hz), 50.4 (d, J_{C-P} = 5.2 Hz), 37.4 (d, J_{C-P} = 4.4 Hz), 25.0, 16.4 (d, J_{C-P} = 7.1 Hz); ^{31}P NMR (121 MHz, CDCl$_3$): δ 6.7; IR (neat): ν_{max} 2979, 2930, 1598, 1572, 1470, 1357, 1311, 1261, 1193, 1063, 1027, 965, 885, 851, 749, 671, 622 cm^{-1}; ESIHRMS *m/z* calcd. for $C_{16}H_{28}N_2O_3P$ [M+NH$_4$]$^+$ 327.1832, found 237.1839.

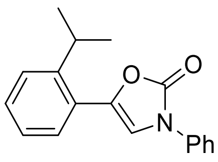

4'r

5-(2-Isopropylphenyl)-3-phenyloxazol-2(3*H*)-one 4'r. Prepared according to general procedure V (500 µmol of the corresponding ynamide). Yield: 52% (72 mg, 258 µmol). Solvent

system for flash column chromatography: petroleum ether/EtOAc: 95/5; Yellow oil; ^{1}H NMR (400 MHz, $CDCl_3$): δ 7.66-7.62 (m, 2H), 7.54-7.46 (m, 3H), 7.44-7.36 (m, 2H), 7.35-7.29 (m, 1H), 7.29-7.23 (m, 1H), 6.96 (s, 1H), 3.34 (sept, J = 6.8 Hz, 1H), 1.30 (d, J = 6.9 Hz, 6H); ^{13}C NMR (100 MHz, $CDCl_3$): δ 152.9, 147.1, 139.8, 135.7, 129.8, 129.7, 128.5, 126.8, 126.2, 126.1, 125.4, 121.2, 111.4, 30.1, 24.0.

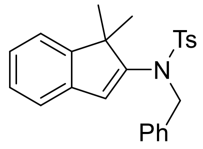

4t

2-(*N*-Benzyl-*N*-4-toluenesulfonylamino)-1,1-dimethyl-1*H*-indene 4t. Prepared according to general procedure V (500 µmol of the corresponding ynamide). Yield: quantitative (202 mg, 500 µmol). Solvent system for flash column chromatography: petroleum ether/EtOAc: 90/10; Pale yellow oil; ^{1}H NMR (400 MHz, $CDCl_3$): δ 7.74 (d, J = 8.3 Hz, 2H), 7.31-7.14 (m, 11H), 6.45 (s, 1H), 4.78 (s, 2H), 2.43 (s, 3H), 1.05 (s, 6H); ^{13}C NMR (100 MHz, $CDCl_3$): δ 152.8, 152.0, 143.9, 139.1, 136.7, 136.4, 129.5, 129.0, 128.6, 128.5, 128.0, 127.8, 126.6, 126.0, 121.6, 121.2, 55.3, 50.6, 24.7, 21.7; IR (neat): ν_{max} 3067, 2967, 2924, 2862, 1598, 1468, 1455, 1348, 1161, 1090, 1018, 814, 752, 704, 661, 620 cm^{-1}; ESIHRMS m/z calcd. for $C_{25}H_{26}NO_2S$ [M+H]$^+$ 404.1679, found 404.1683.

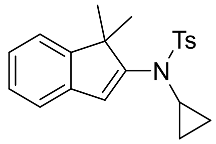

4u

2-(*N*-Cyclopropyl-*N*-4-toluenesulfonylamino)-1,1-dimethyl-1*H*-indene 4u. Prepared according to general procedure V (500 µmol of the corresponding ynamide). Yield: 71% (125 mg, 354 µmol). Solvent system for flash column chromatography: petroleum ether/EtOAc: 95/5; White solid, Mp: 130 °C; ^{1}H NMR (400 MHz, $CDCl_3$): δ 7.82 (d, J = 8.3 Hz, 2H), 7.32 (d, J = 8.0 Hz, 2H), 7.28-7.20 (m, 4H), 6.33 (s, 1H), 2.78-2.72 (m, 1H), 2.45 (s, 3H), 1.34 (s, 6H), 0.76-0.71 (m, 4H); ^{13}C NMR (100 MHz, $CDCl_3$): δ 153.6, 152.1, 143.8, 139.3, 136.5, 129.5, 128.8, 126.9, 126.7, 126.0, 121.7, 121.4, 50.3, 32.2, 25.0, 21.7, 8.6; IR (neat): ν_{max} 2968, 2932, 2876, 1596, 1467, 1363, 1346, 1165, 1091, 1031, 889, 817, 759, 738, 699, 658, 616 cm^{-1}; ESIHRMS m/z calcd. for $C_{21}H_{24}NO_2S$ [M+H]$^+$ 354.1522, found 354.1524.

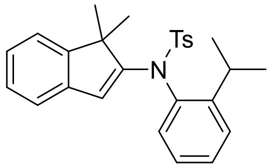

4v

2-(*N*-2-Isopropylphenyl-*N*-4-toluenesulfonylamino)-1,1-dimethyl-1*H*-indene 4v. Prepared according to general procedure VI (300 µmol of the corresponding ynamide). Yield: 54% (70

mg, 162 µmol). Solvent system for flash column chromatography: petroleum ether/EtOAc: 85/15; Orange oil; ^{1}H NMR (400 MHz, $CDCl_3$): δ 7.80 (d, J = 8.3 Hz, 2H), 7.40-7.36 (m, 3H), 7.31 (app. d, J = 7.6 Hz, 1H), 7.25 (obs. d, J = 7.9 Hz, 2H), 7.23-7.17 (m, 2H), 7.13 (td, J = 7.4 and 1.3 Hz, 1H), 7.09-7.05 (m, 2H), 3.61 (sept, J = 6.8 Hz, 1H), 2.39 (s, 3H), 1.34 (d, J = 6.8 Hz, 3H), 1.16 (d, J = 6.9 Hz, 3H), 0.80 (s, 3H), 0.70 (s, 3H); ^{13}C NMR (100 MHz, $CDCl_3$): δ 152.2, 151.3, 150.3, 144.1, 139.7, 136.9, 135.7, 130.7, 129.5, 129.4, 128.8, 127.7, 126.8, 126.1, 125.4, 122.8, 121.5, 120.9, 50.7, 27.9, 24.7, 24.1, 24.0, 23.7, 21.7; IR (neat): ν_{max} 2967, 2936, 2870, 1597, 1567, 1487, 1468, 1450, 1356, 1341, 1325, 1289, 1161, 1138, 1089, 936, 818, 752, 672, 654 cm^{-1}; ESIHRMS *m/z* calcd. for $C_{27}H_{30}NO_2S$ $[M+H]^+$ 432.1992, found 432.2010.

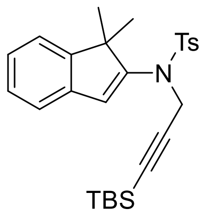

4y

2-[*N*-3-(*tert*-Butyldimethylsilyl)prop-2-yn-1-yl-*N*-4-toluenesulfonylamino]-1,1-dimethyl-1*H*-indene 4y. Prepared according to general procedure VI (500 µmol of the corresponding ynamide). Yield: 87% (202 mg, 434 µmol). Solvent system for flash column chromatography: petroleum ether/EtOAc: 95/5; Orange oil; ^{1}H NMR (400 MHz, $CDCl_3$): δ 7.83 (d, J = 8.3 Hz, 2H), 7.31-7.19 (m, 6H), 6.48 (s, 1H), 4.48 (s, 2H), 2.44 (s, 3H), 1.35 (s, 6H), 0.80 (s, 9H), -0.03 (s, 6H); ^{13}C NMR (100 MHz, $CDCl_3$): δ 151.8, 151.7, 143.8, 139.2, 136.8, 129.5, 128.9, 128.6, 126.7, 126.2, 121.9, 121.4, 100.8, 89.6, 50.9, 42.1, 26.1, 24.4, 21.7, 16.5, -4.7; IR (neat): ν_{max} 2957, 2928, 2857, 2178, 1599, 1469, 1353, 1251, 1163, 1091, 1060, 1001, 838, 825, 812, 753, 677, 663, 606 cm^{-1}; ESIHRMS *m/z* calcd. for $C_{27}H_{36}NO_2SSi$ $[M+H]^+$ 466.2231, found 466.2237.

Experimental Procedures and Characterization Data
Post-functionalization of 2-aminoindenes

General procedure VII:
An oven-dried glass vial was charged with the 2-aminoindene (250 µmol), palladium on carbon 10% wt (27 mg, 25 µmol) and methanol (2.5 mL). The vial was placed in an autoclave, purged with N_2, pressurized with H_2 (5 bars) and stirred at room temperature for 15 h. After the reactor was depressurized and purged with N_2, the reaction mixture was filtered over a plug of Celite® (washed with methanol) and concentrated under reduced pressure. The crude residue was finally purified by flash column chromatography over silica gel to afford the desired product.

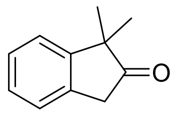

5

1,1-Dimethyl-2,3-dihydro-1*H*-inden-2-one 5. This compound was prepared according to a previously reported procedure.[S3] A 100 mL round bottom flask was charged with 2-(2-oxooxazolidin-3-yl)-1,1-dimethyl-1*H*-indene **4a** (80 mg, 350 µmol) before adding tetrahydrofuran (25 mL) and a 4 M aqueous solution of HCl (25 mL) successively. The resulting heterogeneous solution was stirred vigorously overnight. The reaction mixture was then quenched by addition of a saturated aqueous solution of $NaHCO_3$ (50 mL). The layers were separated, the aqueous layer was extracted with ethyl acetate thrice (3 x 50 mL) and the combined organic layers were dried over $MgSO_4$, filtered and concentrated under high vacuum. The crude residue was finally purified by flash column chromatography over silica gel to afford 1,1-dimethyl-1,3-dihydro-2*H*-inden-2-one. Yield: 59% (33 mg, 206 µmol). Solvent system for flash column chromatography: petroleum ether/EtOAc: 98/2; Yellow oil; ^{1}H NMR (400 MHz, $CDCl_3$): δ 7.34-7.28 (m, 2H), 7.28-7.23 (m, 2H), 3.60 (s, 2H), 1.33 (s, 6H); ^{13}C NMR (100 MHz, $CDCl_3$): δ 220.6, 148.5, 135.0, 127.9, 127.4, 125.0, 123.3, 50.4, 41.8, 25.5; IR (neat): ν_{max} 2967, 2927, 2867, 1749, 1480, 1456, 1396, 1379, 1194, 1102, 1060, 1023, 761, 728 cm^{-1}.

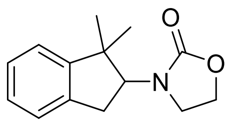

6

2-(2-Oxooxazolidin-3-yl)-1,1-dimethyl-2,3-dihydro-1*H*-indene 6. Prepared according to general procedure VII (350 µmol of the corresponding 2-aminoindene). Yield: 99% (80 mg, 346 µmol). Solvent system for flash column chromatography: petroleum ether/EtOAc: 80/20; Colorless oil; ^{1}H NMR (400 MHz, $CDCl_3$): δ 7.25-7.12 (m, 4H), 4.41 (dd, *J* = 7.1 and 3.6 Hz, 1H), 4.25-4.15 (m, 2H), 3.36-3.26 (m, 2H), 3.02 (td, *J* = 8.9 and 6.2 Hz, 1H), 2.89 (dd, *J* = 16.5 and 3.6

[S3] Fang, L.-C.; Hsung, R. P. *Org. Lett.* **2014**, *16*, 1826-1829.

Hz, 1H), 1.28 (s, 6H); ^{13}C NMR (100 MHz, $CDCl_3$): δ 158.8, 150.1, 138.9, 127.5, 127.2, 124.4, 121.8, 63.7, 62.0, 47.7, 42.7, 33.7, 28.6, 21.9; IR (neat): ν_{max} 2960, 2926, 2877, 1740, 1476, 1424, 1361, 1273, 1216, 1082, 1042, 981, 866, 758, 734 cm^{-1}; ESIHRMS *m/z* calcd. for $C_{14}H_{18}NO_2$ $[M+H]^+$ 232.1332, found 232.1334.

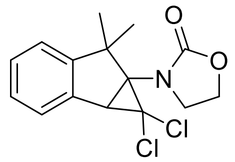

7

1,1-Dichloro-6,6-dimethyl-6a-(2-oxooxazolidin-3-yl)-1,1a,6,6a-tetrahydrocyclopropa[*a*]indene 7. This compound was prepared according to a previously reported procedure.[S4] To a solution of 2-(2-oxooxazolidin-3-yl)-1,1-dimethyl-1*H*-indene **4a** (80 mg, 350 µmol) and $BnNEt_3Cl$ (50 mg, 220 µmol) in chloroform (2 mL) was added dropwise a 10 M solution of NaOH in water (2 mL). The resulting mixture was vigorously stirred at room temperature for two hours. The layers were then separated and the organic phase was washed with water (2 mL) and brine (2 mL) before being dried over $MgSO_4$, filtered and concentrated under reduced pressure. The crude residue was finally purified by flash column chromatography over silica gel to afford the desired product. Yield: 44% (48 mg, 154 µmol). Solvent system for flash column chromatography: petroleum ether/EtOAc: 80/20; White solid, Mp: 121 °C; ^{1}H NMR (400 MHz, $CDCl_3$): δ 7.35 (d, *J* = 7.4 Hz, 1H), 7.28 (td, *J* = 7.5 and 1.4 Hz, 1H), 7.22 (td, *J* = 7.4 and 1.3 Hz, 1H), 7.06 (d, *J* = 7.6 Hz, 1H), 4.49-4.35 (m, 2H), 4.12 (s, 1H), 4.10-3.97 (m, 2H), 1.72 (s, 3H), 1.35 (s, 3H); ^{13}C NMR (100 MHz, $CDCl_3$): δ 157.2, 150.6, 136.8, 128.5, 127.3, 125.6, 122.1, 69.9, 62.2, 56.9, 51.7, 45.7, 45.0, 30.9, 22.6; IR (neat): ν_{max} 2974, 2916, 1750, 1480, 1417, 1269, 1232, 1189, 1133, 1117, 1046, 976, 899, 879, 838, 775, 757, 613 cm^{-1}; ESIHRMS *m/z* calcd. for $C_{15}H_{16}Cl_2NO_2$ $[M+H]^+$ 312.0553, found 312.0563.

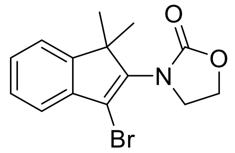

8

2-(2-Oxooxazolidin-3-yl)-3-bromo-1,1-dimethyl-1*H*-indene 8. An oven-dried 5 mL round bottom flask was charged with 2-(2-oxooxazolidin-3-yl)-1,1-dimethyl-1*H*-indene **4a** (80 mg, 350 µmol) and fitted with a rubber septum before being evacuated under high vacuum and backfilled with argon three times. Dry acetonitrile (2.0 mL) was next added before adding *N*-bromo succinimide (62 mg, 350 µmol) in one portion. The reaction mixture was stirred in the dark for 1.5 hours. The reaction mixture was then diluted in dichloromethane (10 mL) and quenched by addition of a 10% wt aqueous solution of $Na_2S_2O_3$ (15 mL). The layers were separated, the aqueous layer was extracted with dichloromethane twice (2 x 15 mL) and the

[S4] Chen, C.; Kattanguru, P.; Tomashenko, O. A.; Karpowicz, R.; Siemiaszko, G.; Bhattacharya, A.; Calasans, V.; Six, Y. *Org. Biomol. Chem.* **2017**, *15*, 5364-5372.

combined organic layers were dried over $MgSO_4$, filtered and concentrated under reduced pressure. The crude residue was finally purified by flash column chromatography over silica gel to afford 3-bromo-2-(2-oxooxazolidin-3-yl)-1,1-dimethyl-1*H*-indene. Yield: 58% (63 mg, 204 µmol). Solvent system for flash column chromatography: petroleum ether/EtOAc: 80/20; White solid, Mp: 167 °C; ^1H NMR (400 MHz, $CDCl_3$): δ 7.42-7.37 (m, 1H), 7.37-7.28 (m, 3H), 4.58-4.52 (m, 2H), 4.01-3.95 (m, 2H), 1.41 (s, 6H); ^{13}C NMR (100 MHz, $CDCl_3$): δ 156.1, 149.8, 147.3, 138.4, 127.6, 127.4, 121.2, 121.2, 118.4, 63.0, 51.0, 46.8, 23.9; IR (neat): ν_{max} 2966, 2927, 2869, 1740, 1623, 1601, 1465, 1412, 1262, 1206, 1124, 1037, 946, 891, 795, 756, 717, 682 cm^{-1}; ESIHRMS *m/z* calcd. for $C_{14}H_{15}{}^{81}BrNO_2$ $[M+H]^+$ 310.0260, found 310.0272.

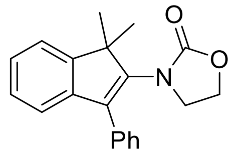

9

2-(2-Oxooxazolidin-3-yl)-3-phenyl-1,1-dimethyl-1*H*-indene 9. This compound was prepared according to a previously reported procedure.[S5] A 10 mL pressure tube was charged with 2-(2-oxooxazolidin-3-yl)-1,1-dimethyl-1*H*-indene **4a** (80 mg, 350 µmol), diphenyliodonium trifluoromethanesulfonate (301 mg, 700 µmol) and copper(II) trifluoromethanesulfonate (25 mg, 70 µmol). The tube was fitted with a rubber septum, evacuated under high vacuum and backfilled with argon three times. Freshly distilled dichloromethane (800 µL) and 2,6-di-*tert*-butylpyridine (157 µL, 700 µmol) were then added, the rubber septum was replaced by a Teflon-coated screw cap and the resulting mixture was stirred at 65 °C for 16 hours. The reaction mixture was cooled to room temperature before being quenched by addition of a saturated aqueous solution of $NaHCO_3$ and extracted with dichloromethane thrice. The combined organic layers were then washed with brine, dried over $MgSO_4$, filtered and concentrated under reduced pressure. The crude residue was finally purified by flash column chromatography over silica gel to afford the desired arylated product. Yield: 93% (99 mg, 324 µmol). Solvent system for flash column chromatography: petroleum ether/EtOAc: 80/20; Yellow oil; ^1H NMR (400 MHz, $CDCl_3$): δ 7.52-7.45 (m, 4H), 7.43-7.36 (m, 2H), 7.34-7.28 (m, 2H), 7.28-7.23 (m, 1H), 4.31-7.26 (m, 2H), 3.55-3.49 (m, 2H), 1.48 (s, 6H); ^{13}C NMR (100 MHz, $CDCl_3$): δ 157.5, 151.5, 145.6, 140.3, 138.4, 133.1, 129.1, 128.5, 128.4, 126.9, 126.6, 121.6, 121.1, 62.7, 50.6, 47.4, 24.5; IR (neat): ν_{max} 3015, 2967, 2922, 2867, 1750, 1469, 1409, 1238, 1219, 1098, 1039, 754, 727, 703, 666 cm^{-1}; ESIHRMS *m/z* calcd. for $C_{20}H_{20}NO_2$ $[M+H]^+$ 306.1489, found 306.1479.

[S5] Gigant, N.; Chausset-Boissarie, L.; Belhomme, M. C.; Poisson, T.; Pannecoucke, X.; Gillaizeau, I. *Org. Lett.* **2013**, *15*, 278-281.

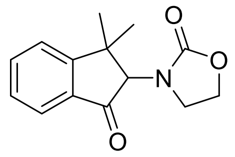

10

2-(2-Oxooxazolidin-3-yl)-1,1-dimethyl-3-oxo-2,3-dihydro-1*H*-indene 10. An oven-dried 25 mL round bottom flask was charged with 2-(2-oxooxazolidin-3-yl)-1,1-dimethyl-1*H*-indene **4a** (80 mg, 350 µmol) and $MgSO_4$ (500 mg) and was fitted with a rubber septum before being evacuated under high vacuum and backfilled with argon three times. Freshly distilled dichloromethane (5 mL) and a 65 mM solution of dimethyldioxirane in acetone (8.08 mL, 525 µmol) were next added and the resulting solution was stirred at room temperature for 45 min. The reaction mixture was next filtered over a plug of Celite® (washed with dichloromethane) and concentrated under reduced pressure. The crude residue was finally purified by flash column chromatography over silica gel to afford the desired product. Yield: 51% (44 mg, 179 µmol). Solvent system for flash column chromatography: petroleum ether/EtOAc: 65/35; White solid; ^{1}H NMR (400 MHz, $CDCl_3$): δ 7.76 (dt, J = 7.7 and 0.9 Hz, 1H), 7.69 (td, J = 7.5 and 1.3 Hz, 1H), 7.52 (dt, J = 7.8 and 0.8 Hz, 1H), 7.43 (td, J = 7.5 and 1.0 Hz, 1H), 4.63 (s, 1H), 4.51-4.35 (m, 2H), 4.12 (dt, J = 9.4 and 8.2 Hz, 1H), 3.48 (td, J = 8.7 and 5.8 Hz, 1H), 1.62 (s, 3H), 1.27 (s, 3H); ^{13}C NMR (100 MHz, $CDCl_3$): δ 199.8, 161.0, 159.6, 136.2, 132.8, 128.2, 124.1, 123.9, 69.4, 62.8, 44.3, 43.1, 27.6, 27.2; IR (neat): ν_{max} 2929, 2860, 1747, 1715, 1605, 1477, 1418, 1379, 1248, 1227, 1208, 1076, 1039, 886, 783, 762, 667 cm^{-1}; ESIHRMS *m/z* calcd. for $C_{14}H_{16}NO_3$ $[M+H]^+$ 246.1125, found 246.1128.

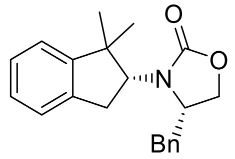

11

(*R*,*S*)-2-(2-Oxo-4-benzyloxazolidin-3-yl)-1,1-dimethyl-2,3-dihydro-1*H*-indene 11. Prepared according to general procedure VII (250 µmol of the corresponding 2-aminoindene). Obtained as a 80:20 mixture of diastereoisomers in the crude reaction mixture and 80:20 after purification (determined by ^{1}H NMR analysis). Yield: 96% (77 mg, 240 µmol). Solvent system for flash column chromatography: petroleum ether/EtOAc: 80/20; Colorless oil; $[\alpha]_D^{25}$ + 22 (*c* 1.0, $CHCl_3$); ^{1}H NMR (400 MHz, $CDCl_3$): δ 7.36-7.15 (m, 7H), 6.86-6.81 (m, 1.60H, major diastereoisomer), 6.74-6.69 (m, 0.40H, minor diastereoisomer), 4.57 (dd, J = 7.1 and 4.3 Hz, 0.20H, minor diastereoisomer), 4.22 (dd, J = 7.5 and 4.9 Hz, 0.80H, major diastereoisomer), 4.09-4.01 (m, 1.60H, major diastereoisomer), 3.98 (dd, J = 8.9 and 2.5 Hz, 0.20H, minor diastereoisomer), 3.94-3.89 (m, 0.20H, minor diastereoisomer), 3.82 (app. dq, J = 7.2 and 4.1 Hz, 0.80H, major diastereoisomer), 3.55 (dd, J = 16.6 and 4.9 Hz, 0.80H, major diastereoisomer), 3.51-3.41 (m, 0.40H, minor diastereoisomer), 3.30 (dd, J = 16.6 and 7.5 Hz, 0.80H, major diastereoisomer), 3.16 (dd, J = 16.3 and 4.4 Hz, 0.20H, minor diastereoisomer), 3.00 (dd, J = 13.1 and 3.3 Hz, 0.20H, minor diastereoisomer), 2.58 (dd, J = 13.1 and 10.8 Hz,

0.20H, minor diastereoisomer), 2.46 (d, *J* = 7.2 Hz, 1.60H, major diastereoisomer), 1.38 (s, 4.80H, major diastereoisomer), 1.33 (s, 0.60H, minor diastereoisomer), 1.28 (s, 0.60H, minor diastereoisomer); ^{13}C NMR (100 MHz, $CDCl_3$): δ 158.9 (minor diastereoisomer), 158.6 (major diastereoisomer), 150.5 (minor diastereoisomer), 150.4 (major diastereoisomer), 139.6 (major diastereoisomer), 139.0 (minor diastereoisomer), 136.1 (minor diastereoisomer), 136.1 (major diastereoisomer), 129.3 (minor diastereoisomer), 129.2 (major diastereoisomer), 129.0 (major diastereoisomer), 128.9 (minor diastereoisomer), 127.7 (minor diastereoisomer), 127.6 (major diastereoisomer), 127.4 (minor diastereoisomer), 127.2 (major diastereoisomer), 127.1, 124.7 (minor diastereoisomer), 124.5 (major diastereoisomer), 122.4 (major diastereoisomer), 122.0 (minor diastereoisomer), 66.8 (major diastereoisomer), 66.3 (minor diastereoisomer), 66.0 (major diastereoisomer), 64.8 (minor diastereoisomer), 57.7 (major diastereoisomer), 56.6 (minor diastereoisomer), 48.3 (major diastereoisomer), 48.0 (minor diastereoisomer), 40.0, 35.3 (minor diastereoisomer), 33.0 (major diastereoisomer), 29.4 (major diastereoisomer), 28.8 (minor diastereoisomer), 22.5 (major diastereoisomer), 21.9 (minor diastereoisomer); IR (neat): ν_{max} 2959, 1746, 1479, 1455, 1410, 1389, 1363, 1231, 1091, 1076, 1010, 912, 763, 732, 702 cm^{-1}; ESIHRMS *m/z* calcd. for $C_{21}H_{24}NO_2$ $[M+H]^+$ 322.1802, found 322.1807.

Supporting Information

Copies of ^{1}H, ^{13}C and ^{31}P NMR Spectra

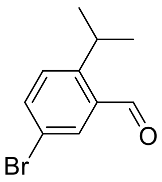
Br
O

S1

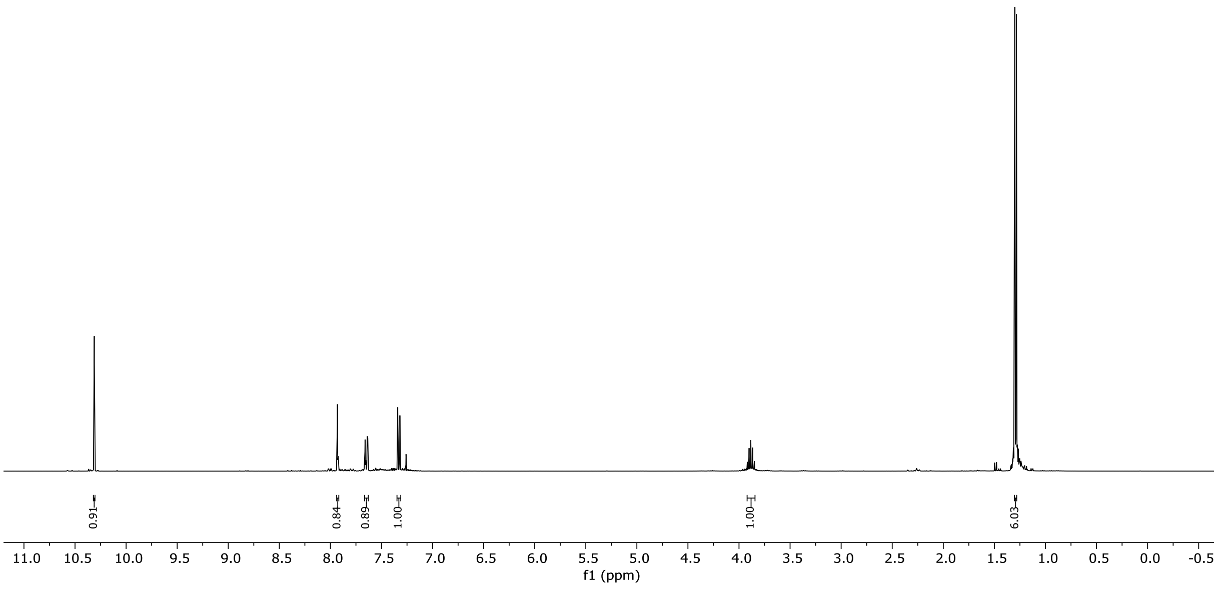
0.91
0.84
0.89
1.00
1.00
6.03
11.0 10.5 10.0 9.5 9.0 8.5 8.0 7.5 7.0 6.5 6.0 5.5 5.0 4.5 4.0 3.5 3.0 2.5 2.0 1.5 1.0 0.5 0.0 -0.5
f1 (ppm)

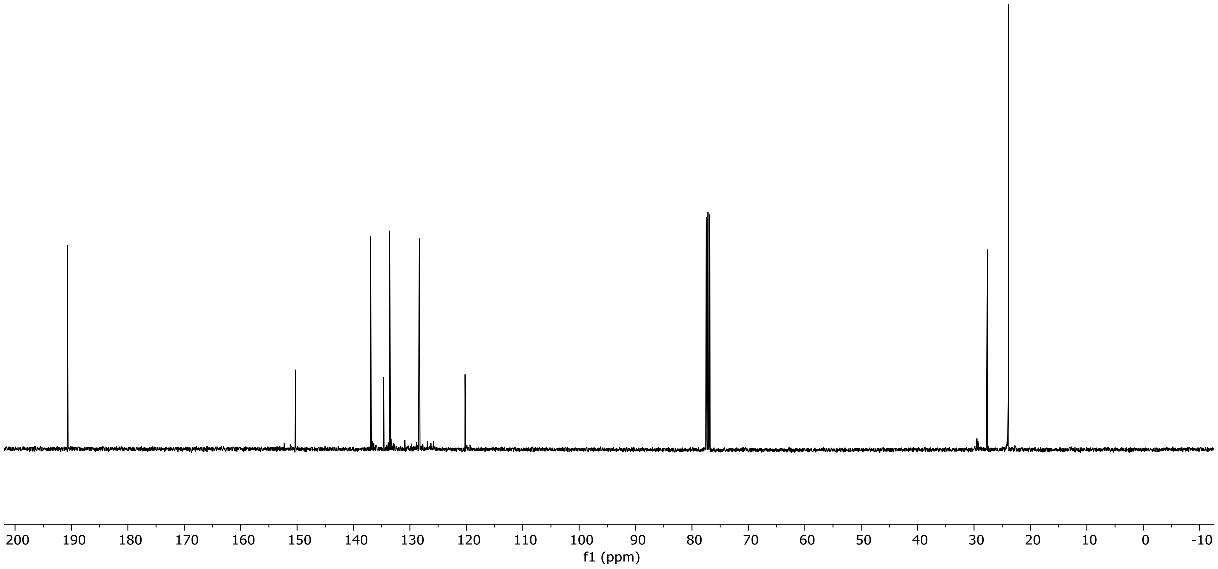
200 190 180 170 160 150 140 130 120 110 100 90 80 70 60 50 40 30 20 10 0 -10
f1 (ppm)

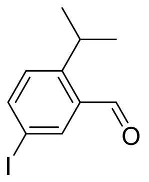
O
I

S2

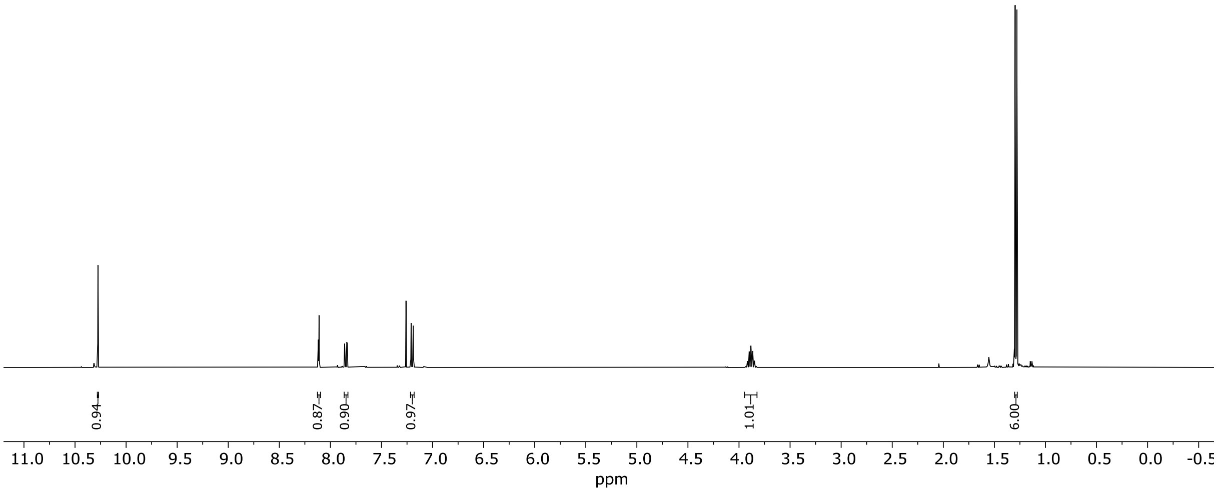
0.94
0.87
0.90
0.97
1.01
6.00
11.0 10.5 10.0 9.5 9.0 8.5 8.0 7.5 7.0 6.5 6.0 5.5 5.0 4.5 4.0 3.5 3.0 2.5 2.0 1.5 1.0 0.5 0.0
ppm

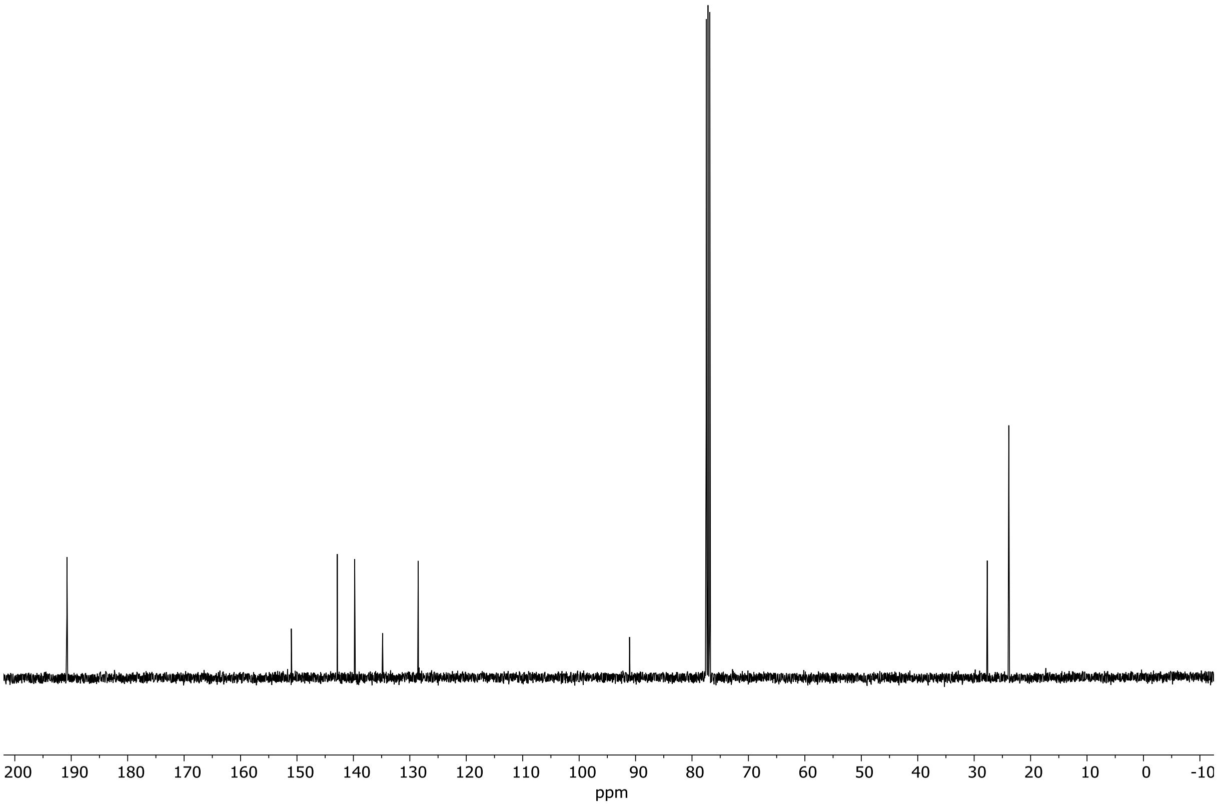
200 190 180 170 160 150 140 130 120 110 100 90 80 70 60 50 40 30 20 10 0 -10
ppm

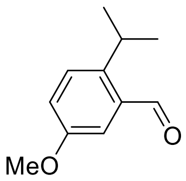
MeO
O

S3

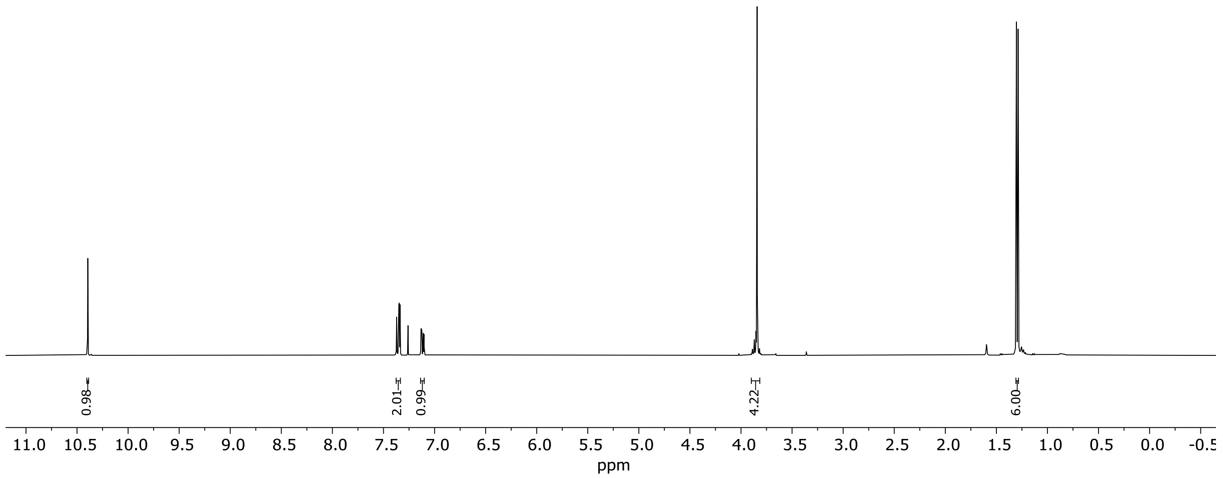
0.98
2.01
0.99
4.22
6.00
11.0 10.5 10.0 9.5 9.0 8.5 8.0 7.5 7.0 6.5 6.0 5.5 5.0 4.5 4.0 3.5 3.0 2.5 2.0 1.5 1.0 0.5 0.0
ppm

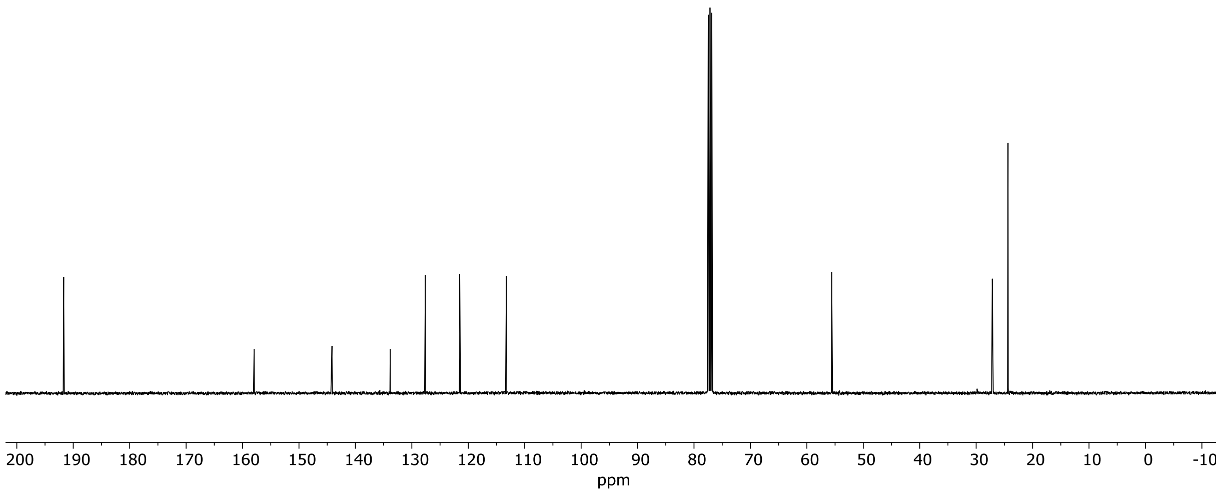
200 190 180 170 160 150 140 130 120 110 100 90 80 70 60 50 40 30 20 10 0 -10
ppm

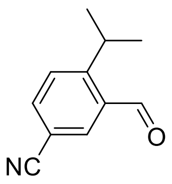
NC
O

S4

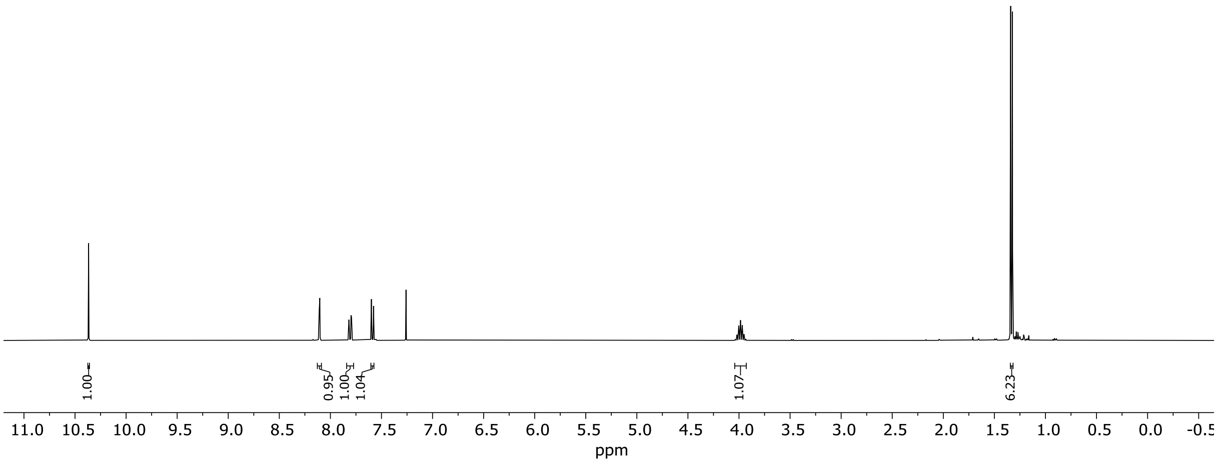
1.00
0.95
1.00
1.04
1.07
6.23
11.0 10.5 10.0 9.5 9.0 8.5 8.0 7.5 7.0 6.5 6.0 5.5 5.0 4.5 4.0 3.5 3.0 2.5 2.0 1.5 1.0 0.5 0.0
ppm

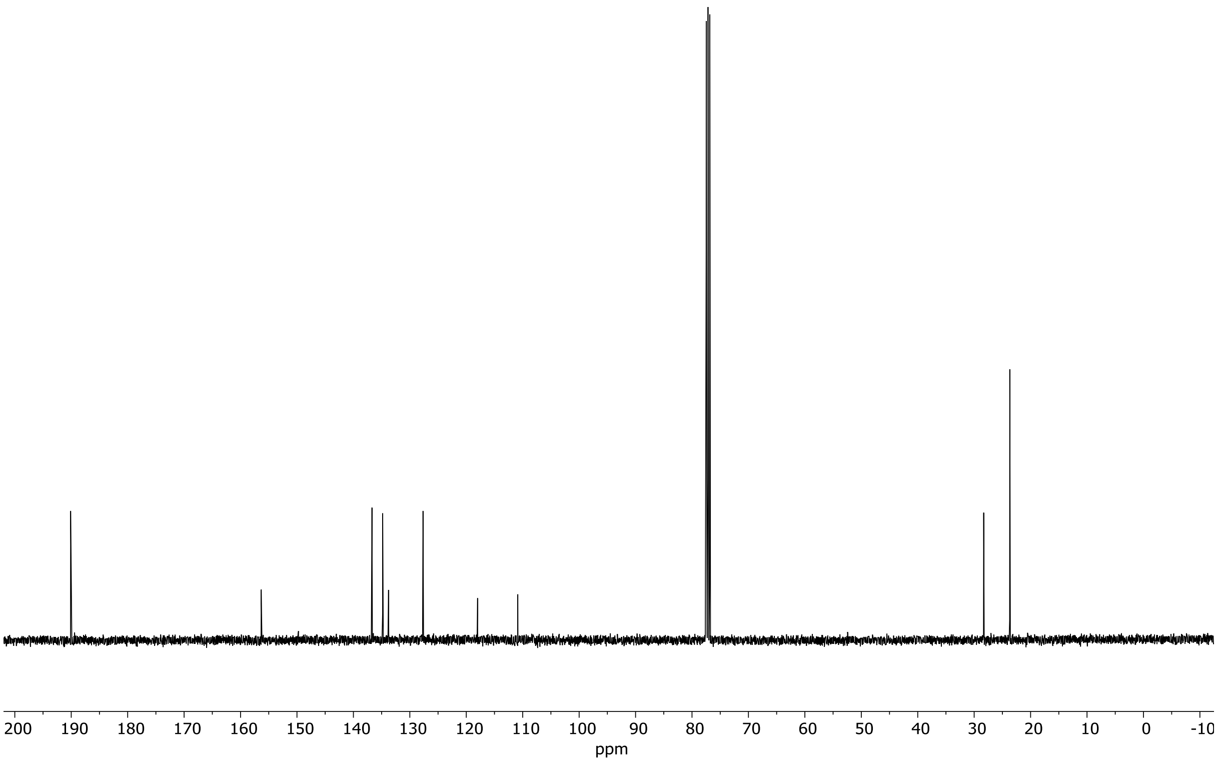
200 190 180 170 160 150 140 130 120 110 100 90 80 70 60 50 40 30 20 10 0 -10
ppm

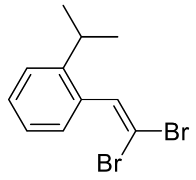
Br
Br

S5

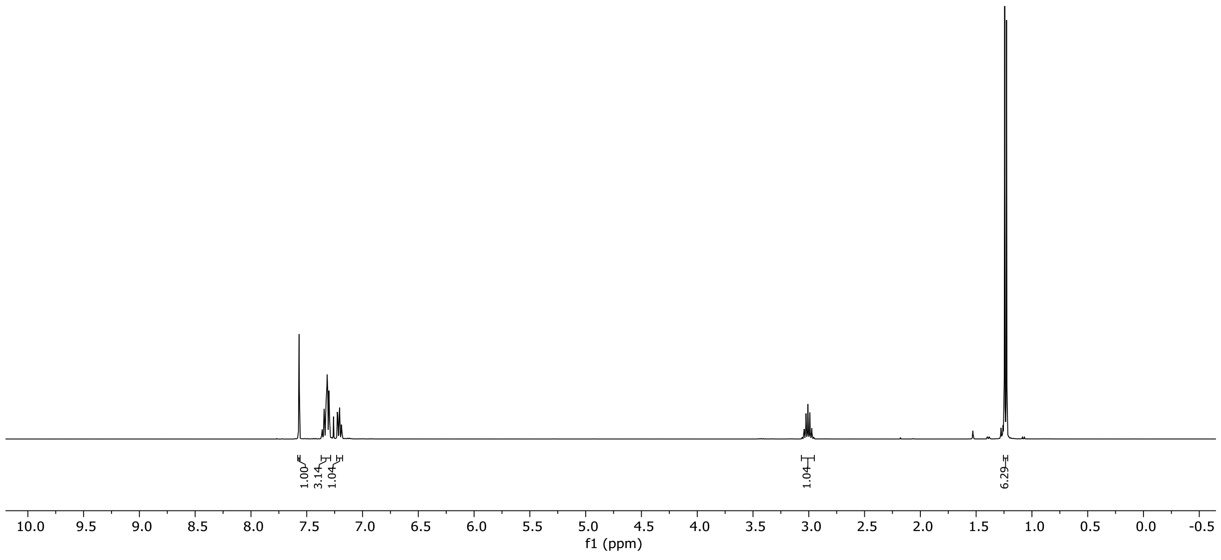
1.00
3.14
1.04
1.04
6.29
10.0 9.5 9.0 8.5 8.0 7.5 7.0 6.5 6.0 5.5 5.0 4.5 4.0 3.5 3.0 2.5 2.0 1.5 1.0 0.5 0.0 -0.5
f1 (ppm)

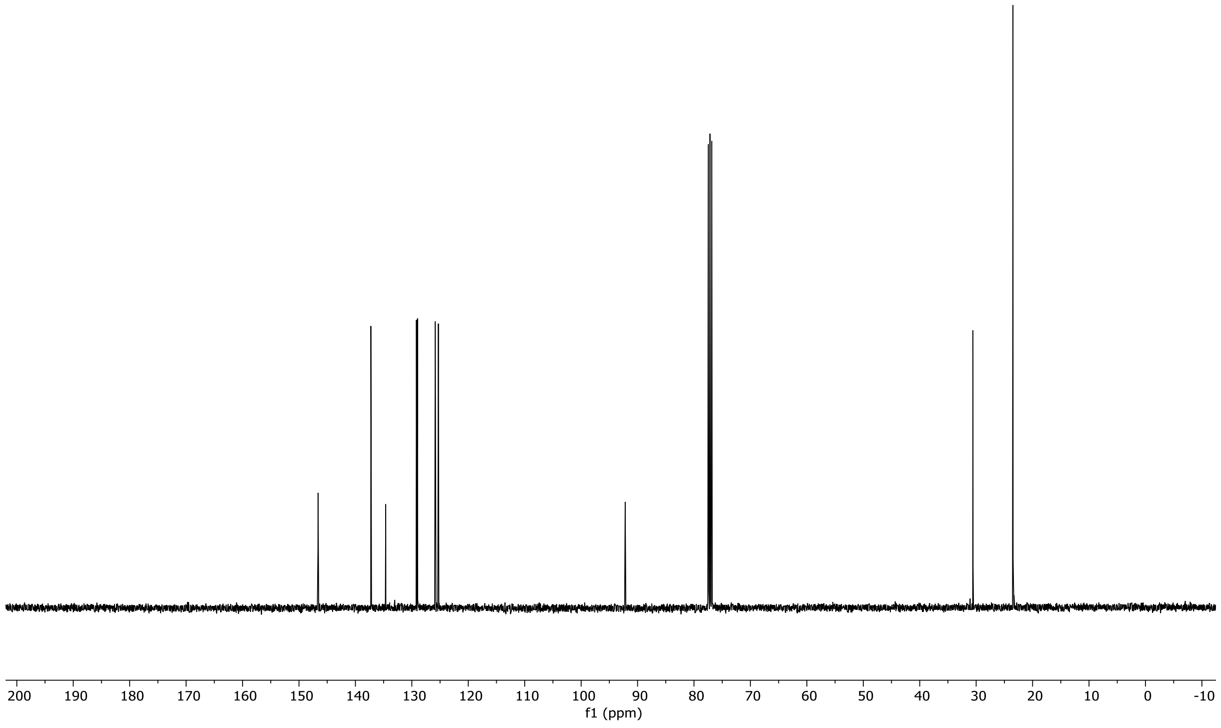
200 190 180 170 160 150 140 130 120 110 100 90 80 70 60 50 40 30 20 10 0 -10
f1 (ppm)

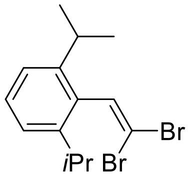
Br
iPr Br

S6

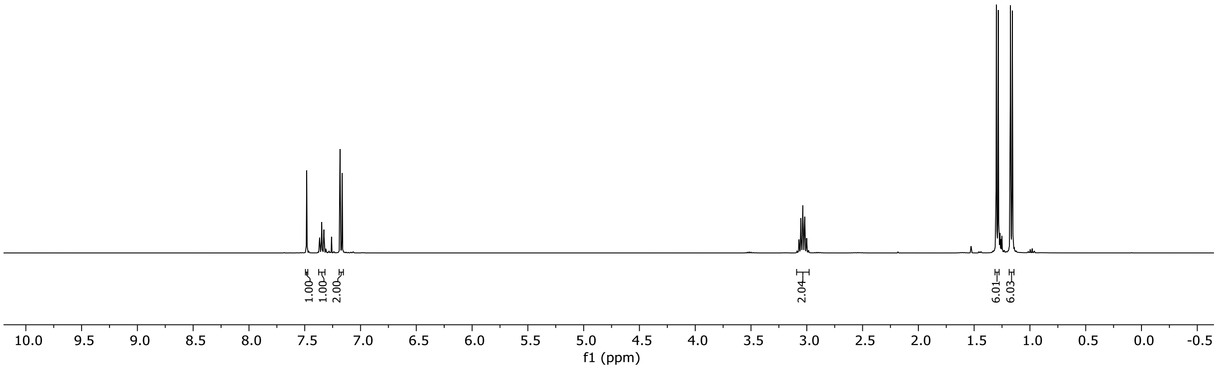
1.00
1.00
2.00
2.04
6.01
6.03
10.0 9.5 9.0 8.5 8.0 7.5 7.0 6.5 6.0 5.5 5.0 4.5 4.0 3.5 3.0 2.5 2.0 1.5 1.0 0.5 0.0 -0.5
f1 (ppm)

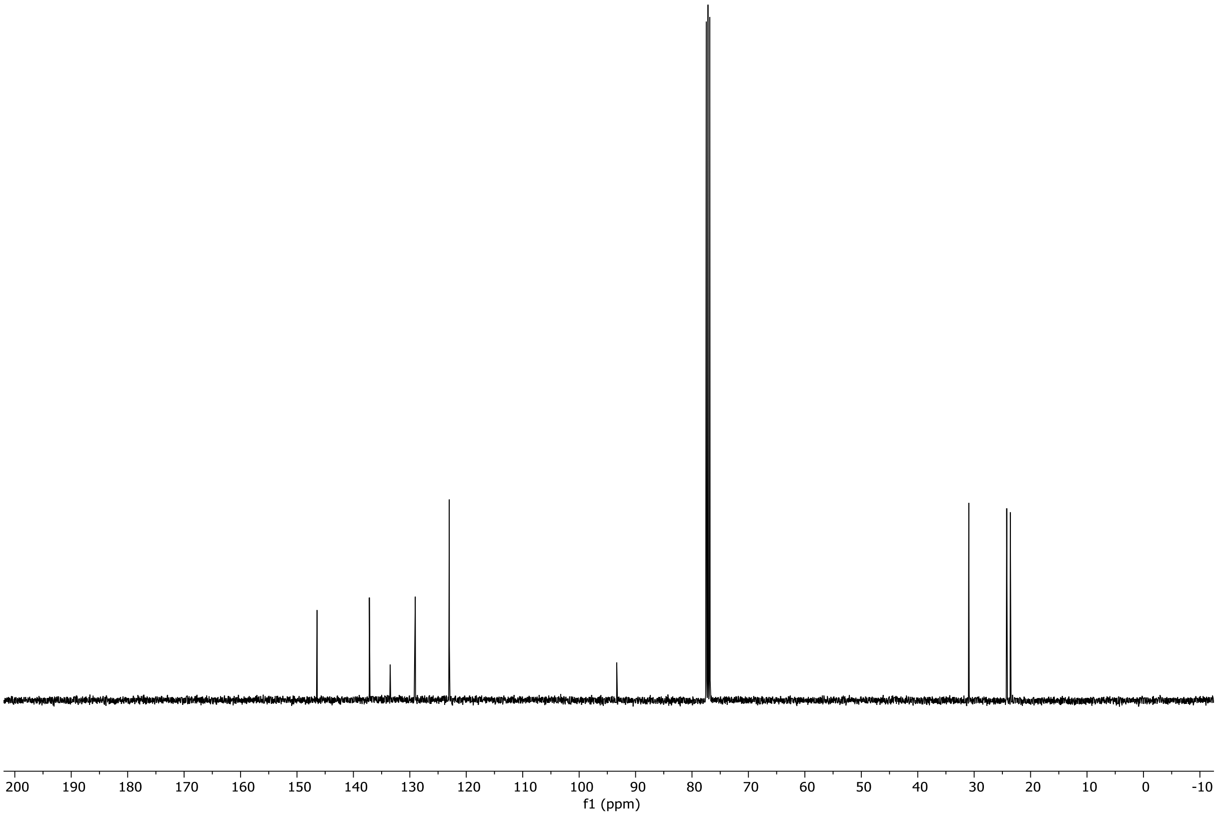
200 190 180 170 160 150 140 130 120 110 100 90 80 70 60 50 40 30 20 10 0 -10
f1 (ppm)

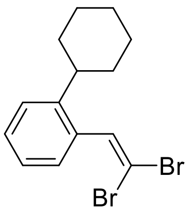
Br
Br

S7

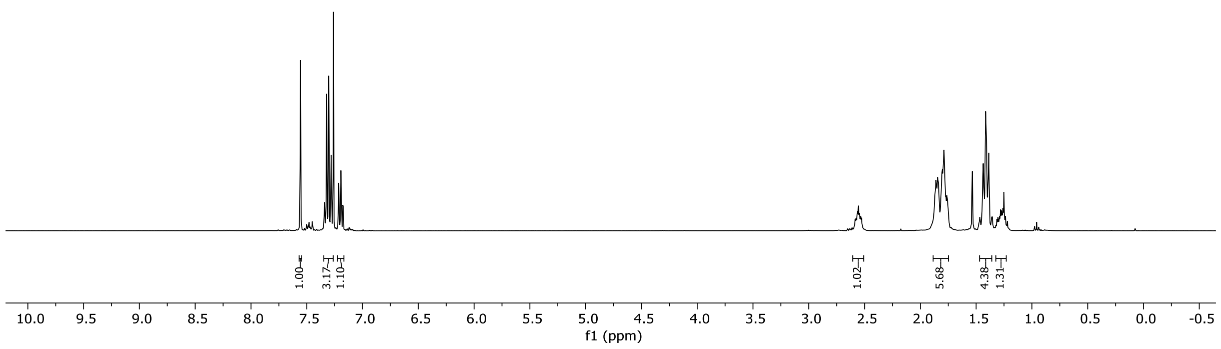
1.00
3.17
1.10
1.02
5.68
4.38
1.31
10.0 9.5 9.0 8.5 8.0 7.5 7.0 6.5 6.0 5.5 5.0 4.5 4.0 3.5 3.0 2.5 2.0 1.5 1.0 0.5 0.0 -0.5
f1 (ppm)

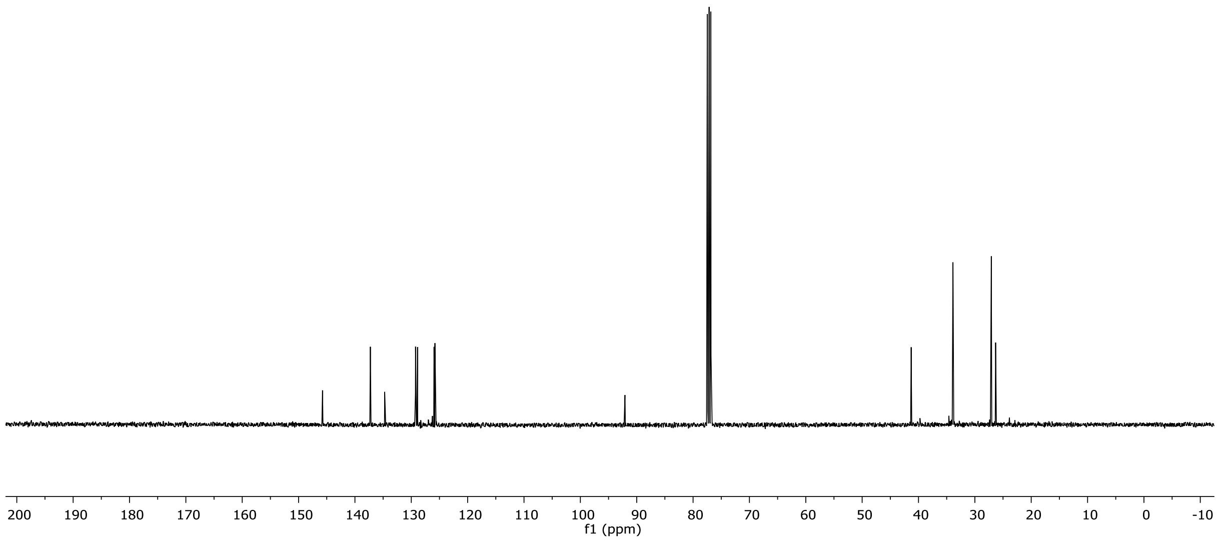
200 190 180 170 160 150 140 130 120 110 100 90 80 70 60 50 40 30 20 10 0 -10
f1 (ppm)

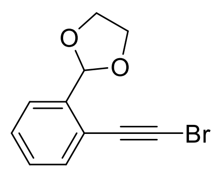
O
O
Br

S8

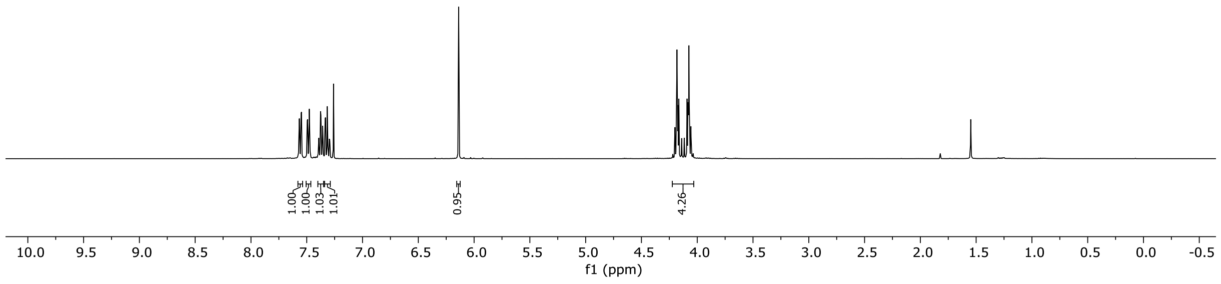
1.00
1.00
1.03
1.01
0.95
4.26
10.0 9.5 9.0 8.5 8.0 7.5 7.0 6.5 6.0 5.5 5.0 4.5 4.0 3.5 3.0 2.5 2.0 1.5 1.0 0.5 0.0 -0.5
f1 (ppm)

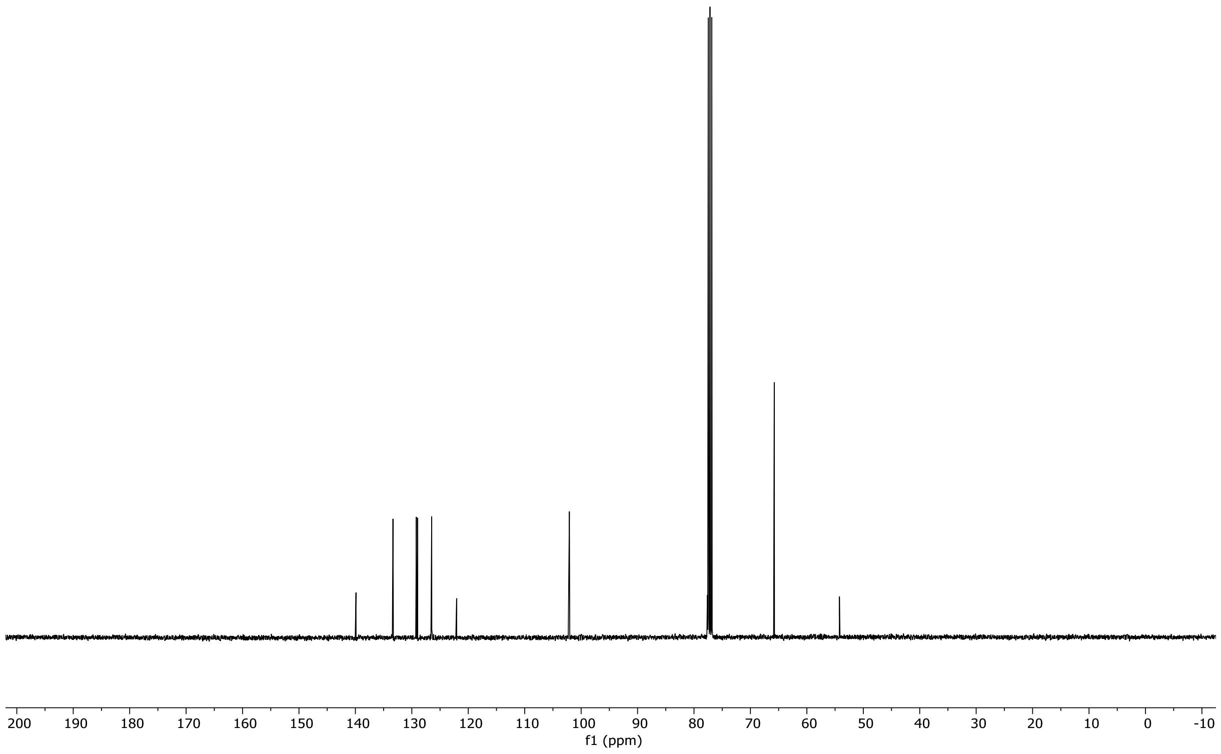
200 190 180 170 160 150 140 130 120 110 100 90 80 70 60 50 40 30 20 10 0 -10
f1 (ppm)

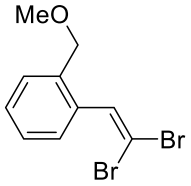
MeO
Br
Br

S9

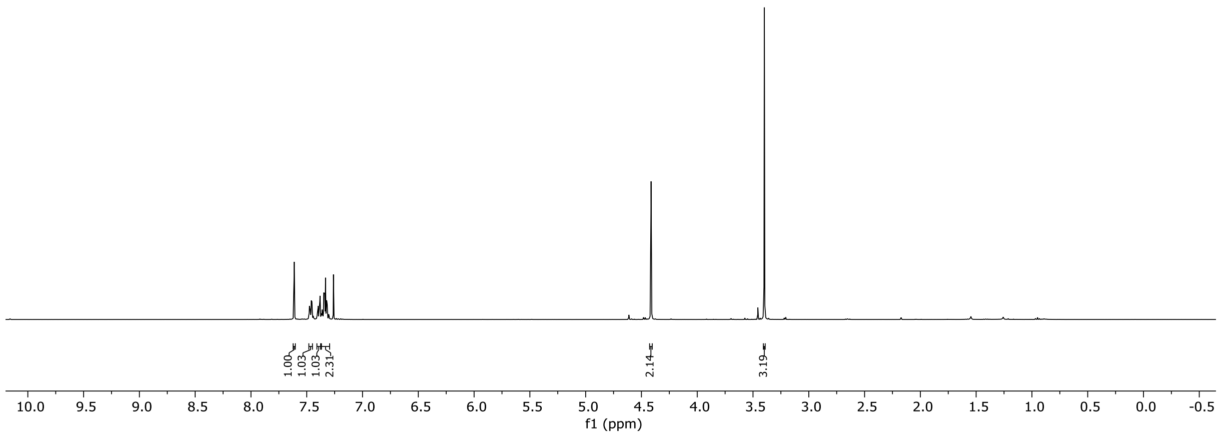
1.00
1.03
1.03
2.31
2.14
3.19
10.0
9.5
9.0
8.5
8.0
7.5
7.0
6.5
6.0
5.5
5.0
4.5
4.0
3.5
3.0
2.5
2.0
1.5
1.0
0.5
0.0
-0.5
f1 (ppm)

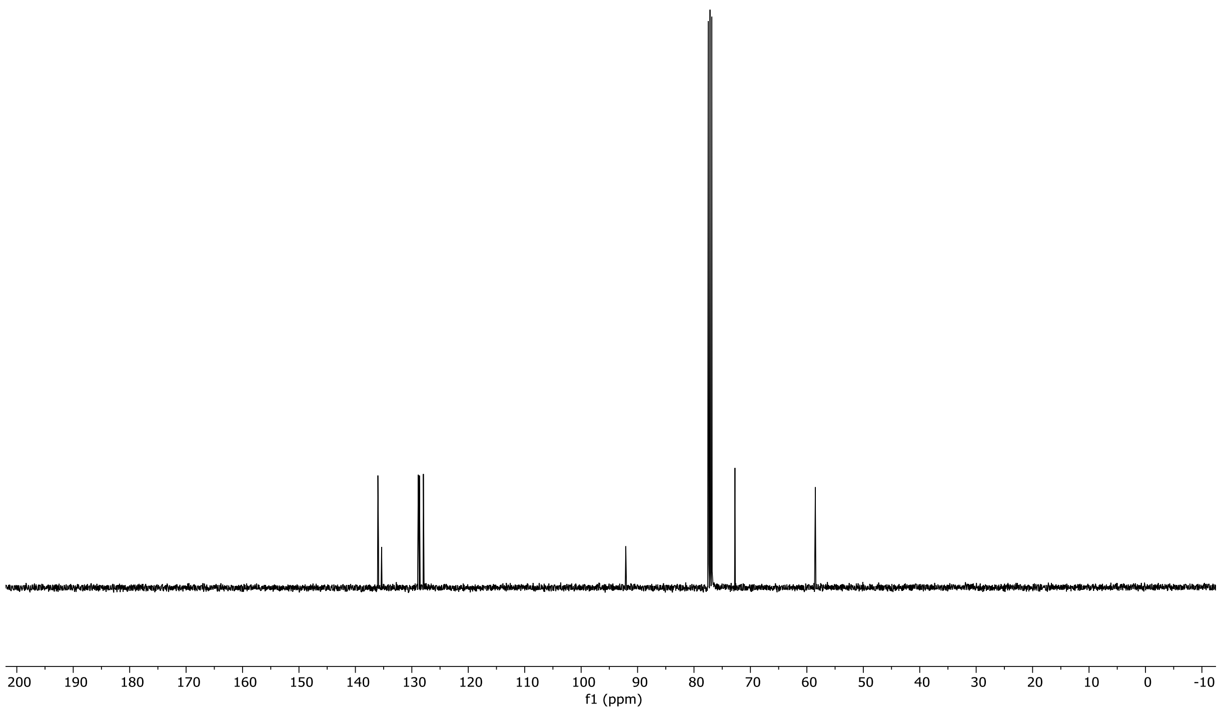
200
190
180
170
160
150
140
130
120
110
100
90
80
70
60
50
40
30
20
10
0
-10
f1 (ppm)

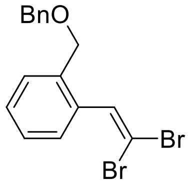
BnO
Br
Br

S10

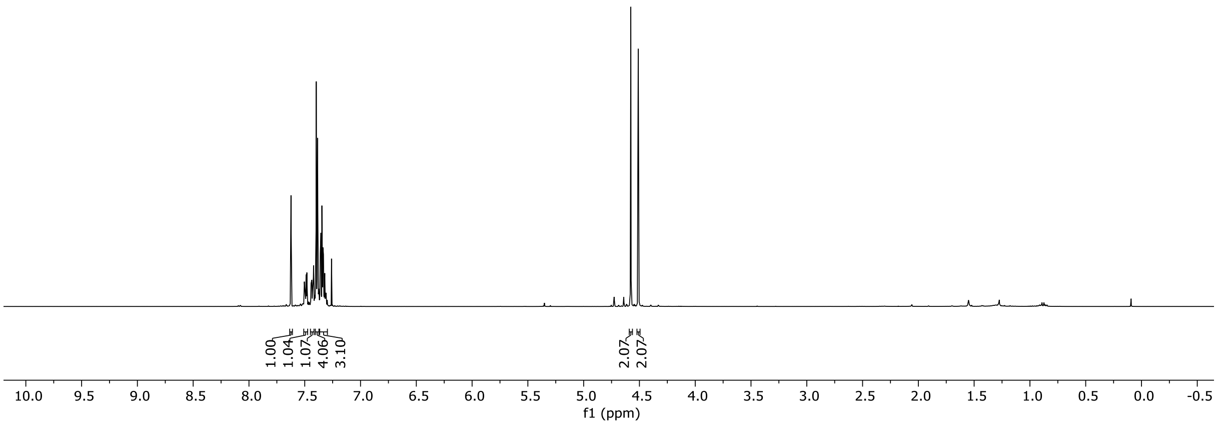
1.00
1.04
1.07
4.06
3.10
2.07
2.07
10.0 9.5 9.0 8.5 8.0 7.5 7.0 6.5 6.0 5.5 5.0 4.5 4.0 3.5 3.0 2.5 2.0 1.5 1.0 0.5 0.0 -0.5
f1 (ppm)

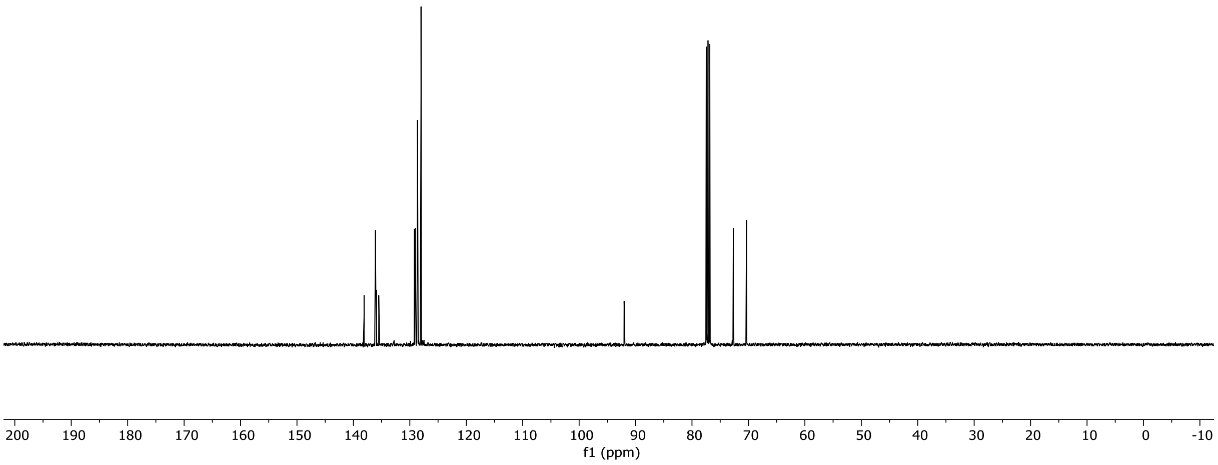
200 190 180 170 160 150 140 130 120 110 100 90 80 70 60 50 40 30 20 10 0 -10
f1 (ppm)

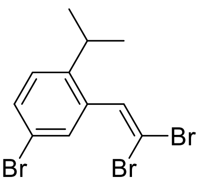
Br
Br
Br

S11

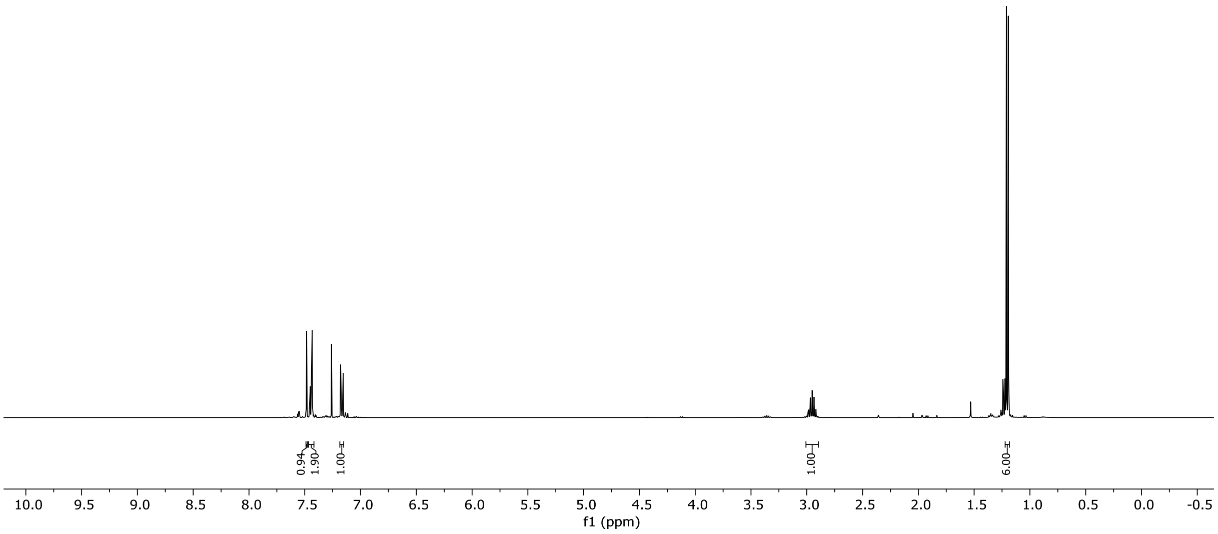
0.94
1.90
1.00
1.00
6.00
10.0 9.5 9.0 8.5 8.0 7.5 7.0 6.5 6.0 5.5 5.0 4.5 4.0 3.5 3.0 2.5 2.0 1.5 1.0 0.5 0.0 -0.5
f1 (ppm)

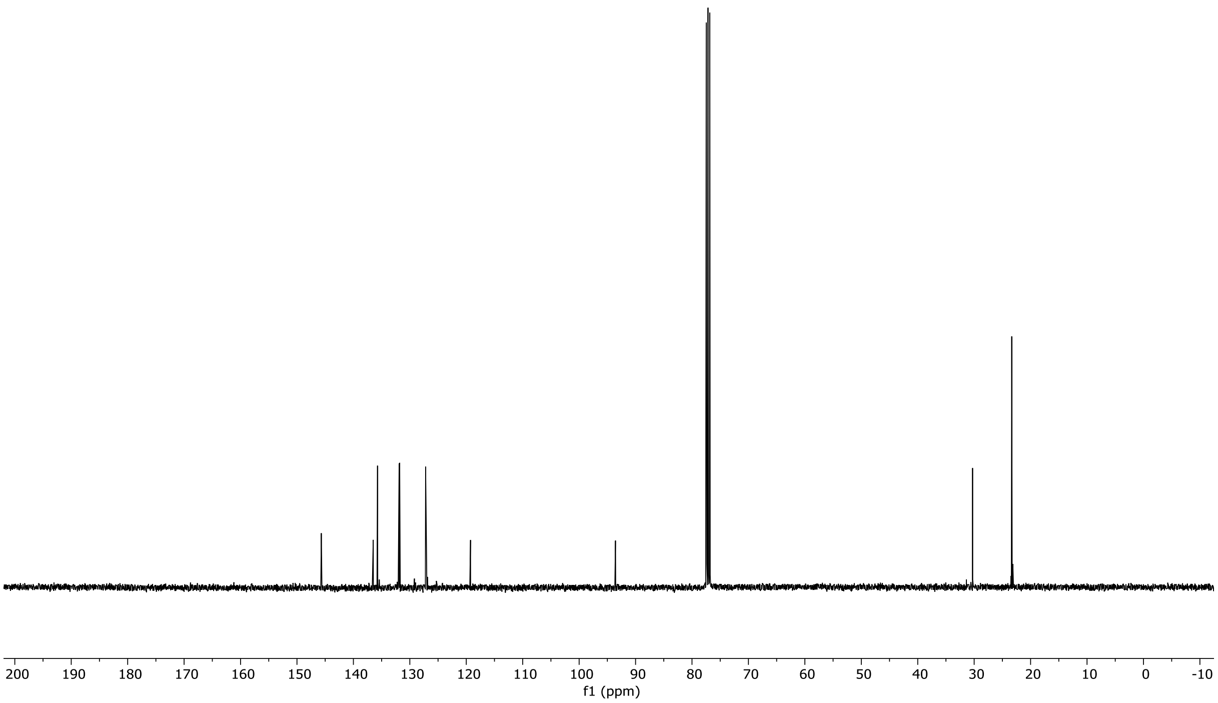
200 190 180 170 160 150 140 130 120 110 100 90 80 70 60 50 40 30 20 10 0 -10
f1 (ppm)

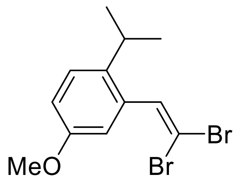

S12

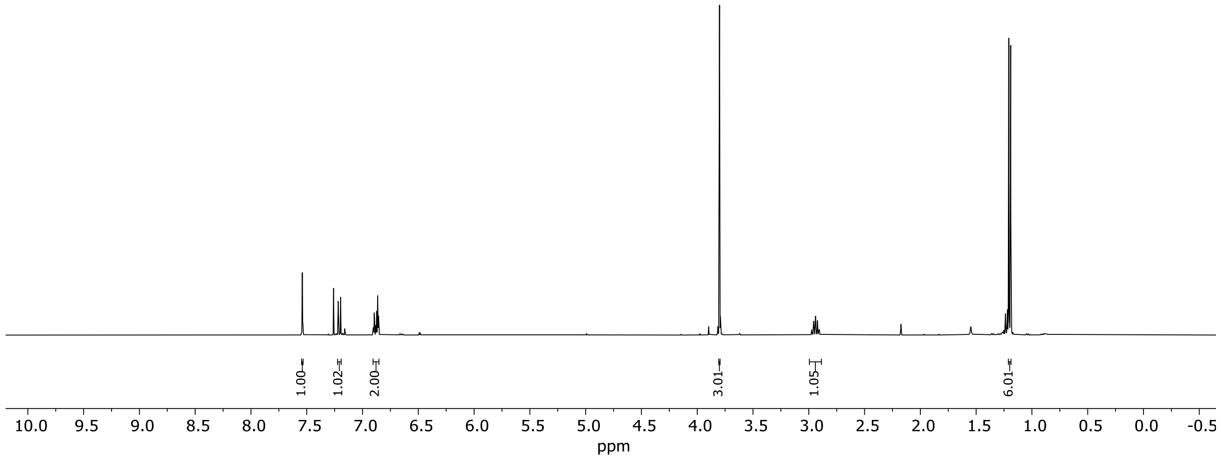

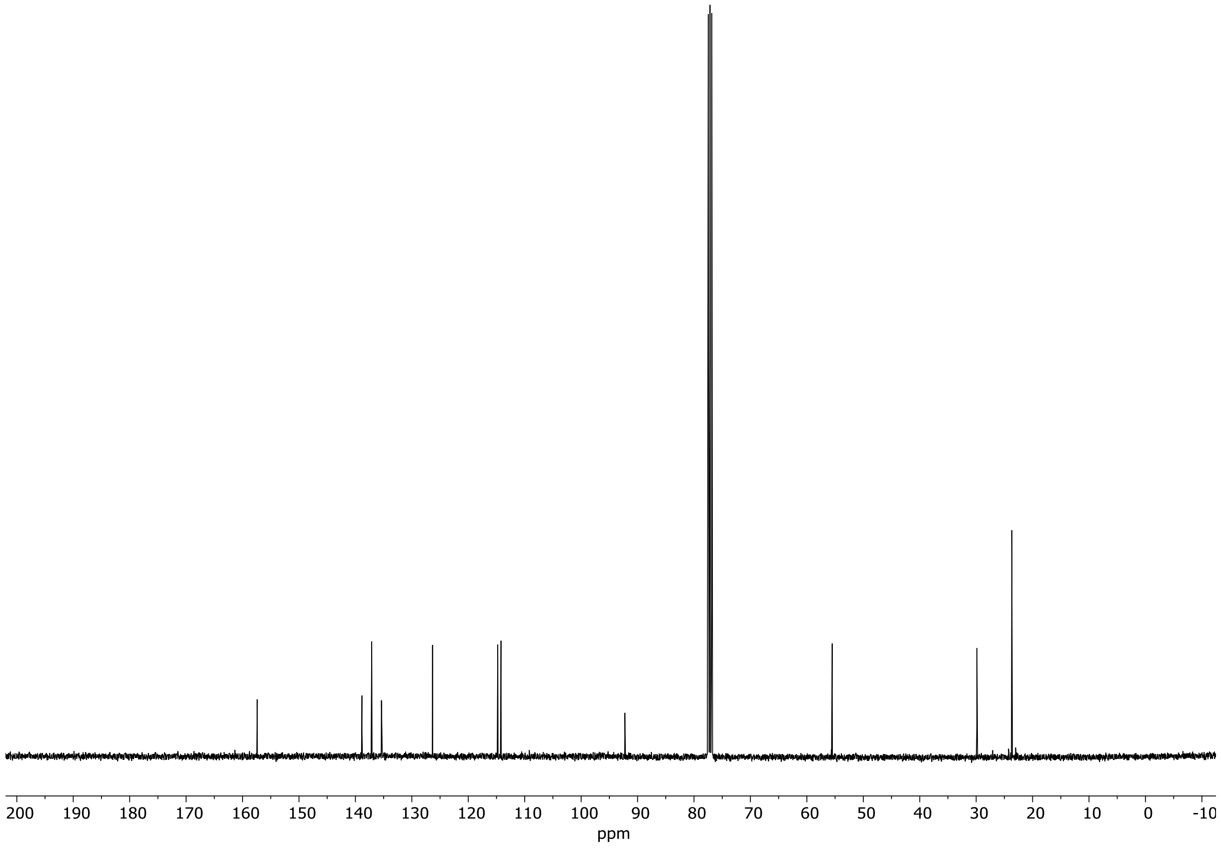

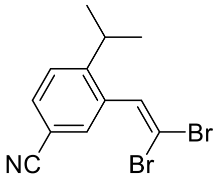
NC
Br
Br

S13

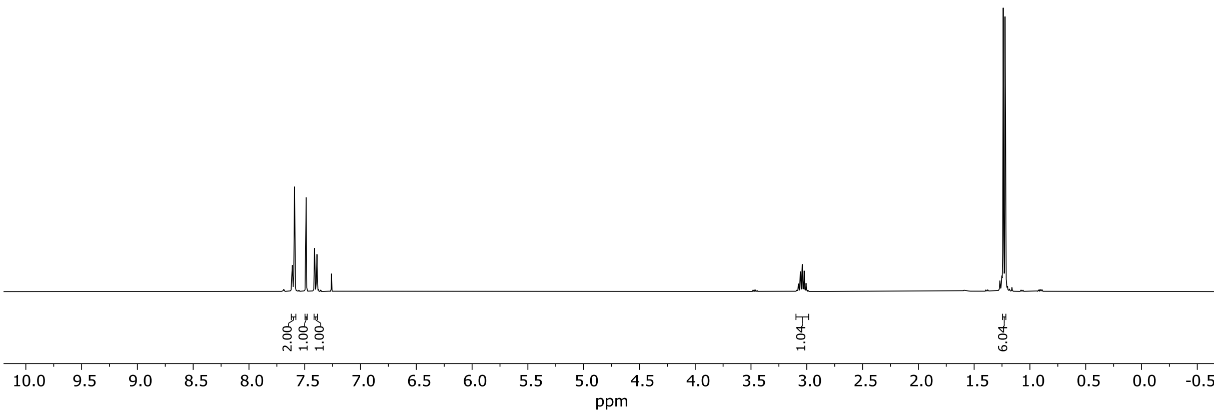
2.00
1.00
1.00
1.04
6.04
10.0
9.5
9.0
8.5
8.0
7.5
7.0
6.5
6.0
5.5
5.0
4.5
4.0
3.5
3.0
2.5
2.0
1.5
1.0
0.5
0.0
-0.5
ppm

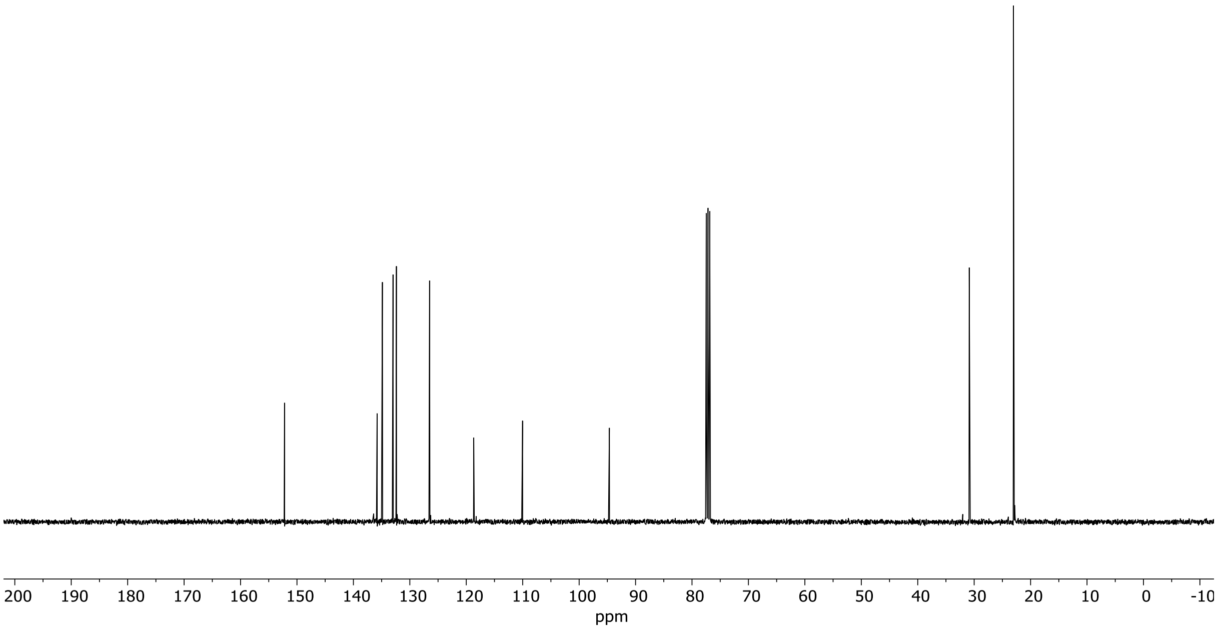
200
190
180
170
160
150
140
130
120
110
100
90
80
70
60
50
40
30
20
10
0
-10
ppm

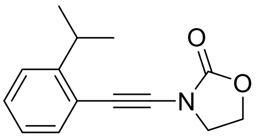

1a

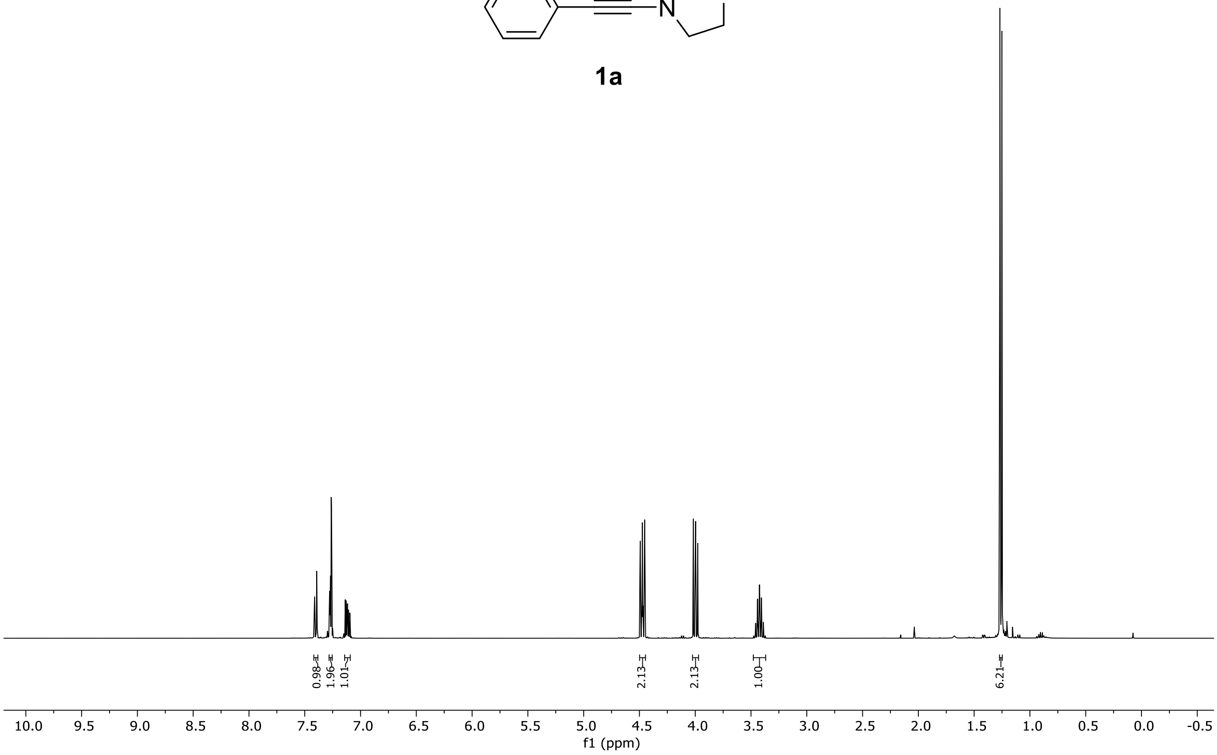
0.98
1.96
1.01
2.13
2.13
1.00
6.21
10.0 9.5 9.0 8.5 8.0 7.5 7.0 6.5 6.0 5.5 5.0 4.5 4.0 3.5 3.0 2.5 2.0 1.5 1.0 0.5 0.0 -0.5
f1 (ppm)

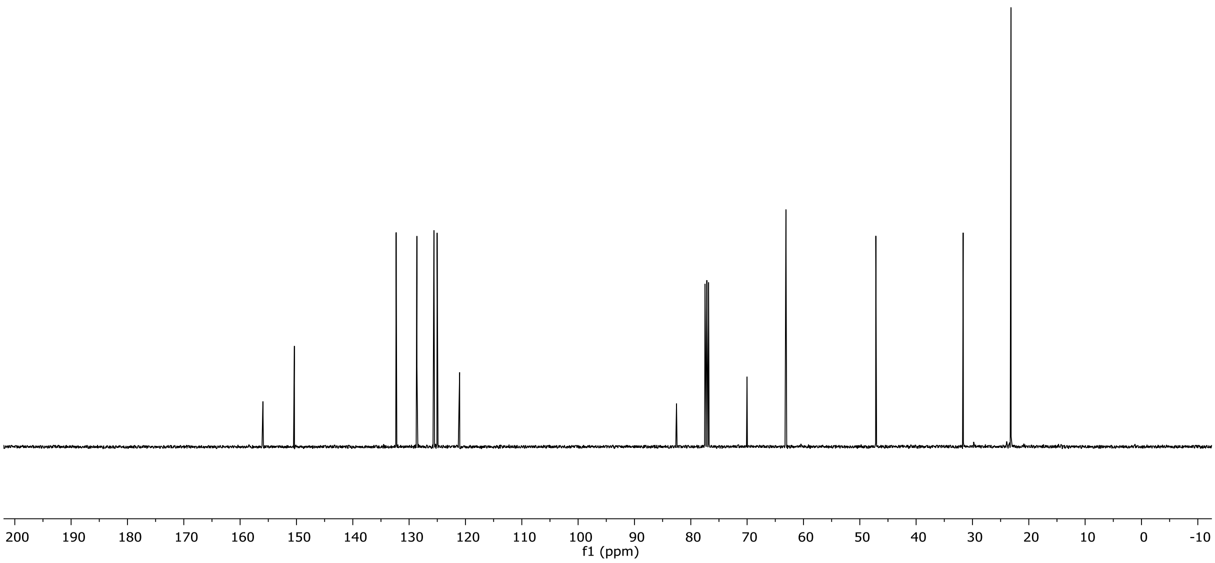
200 190 180 170 160 150 140 130 120 110 100 90 80 70 60 50 40 30 20 10 0 -10
f1 (ppm)

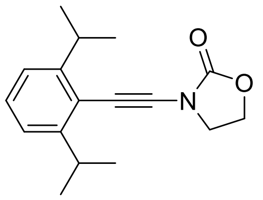
O
N
O

1b

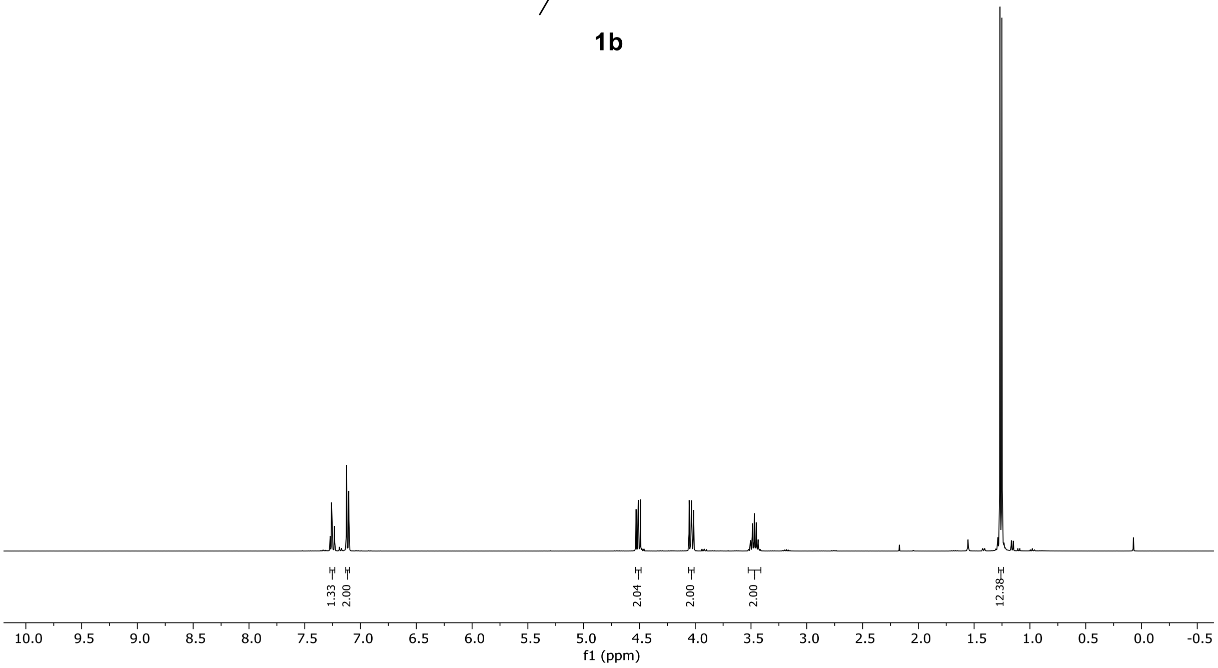
1.33
2.00
2.04
2.00
2.00
12.38
10.0 9.5 9.0 8.5 8.0 7.5 7.0 6.5 6.0 5.5 5.0 4.5 4.0 3.5 3.0 2.5 2.0 1.5 1.0 0.5 0.0 -0.5
f1 (ppm)

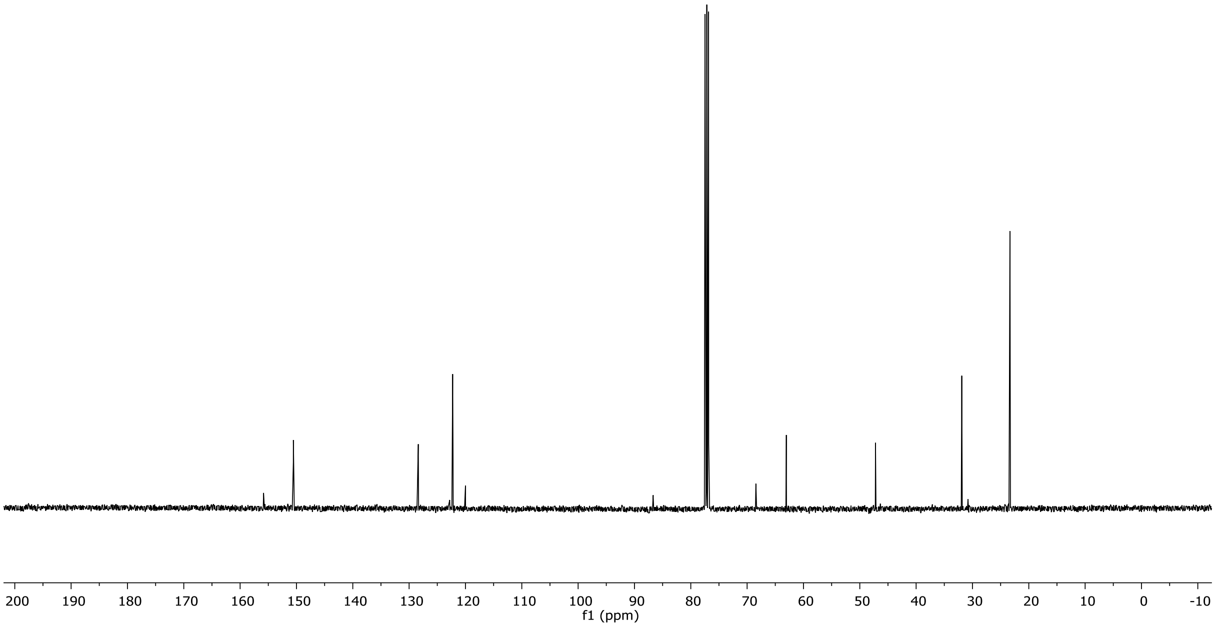
200 190 180 170 160 150 140 130 120 110 100 90 80 70 60 50 40 30 20 10 0 -10
f1 (ppm)

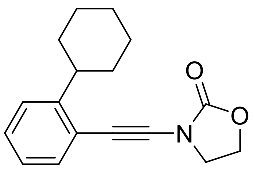
O
N
O

1c

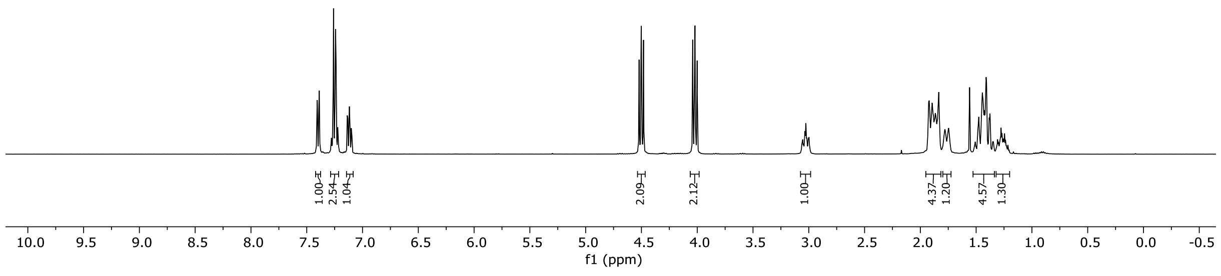
1.00
2.54
1.04
2.09
2.12
1.00
4.37
1.20
4.57
1.30
10.0 9.5 9.0 8.5 8.0 7.5 7.0 6.5 6.0 5.5 5.0 4.5 4.0 3.5 3.0 2.5 2.0 1.5 1.0 0.5 0.0 -0.5
f1 (ppm)

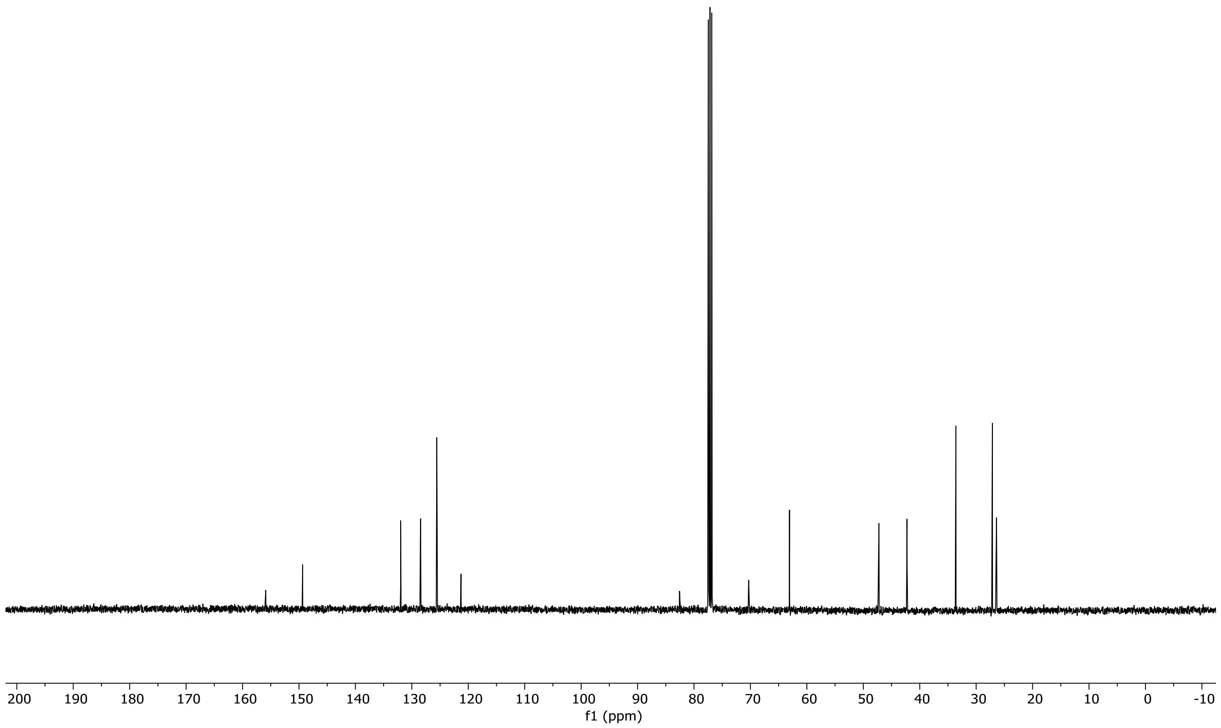
200 190 180 170 160 150 140 130 120 110 100 90 80 70 60 50 40 30 20 10 0 -10
f1 (ppm)

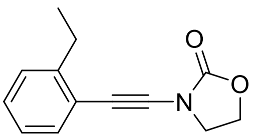
O
O
N

1d

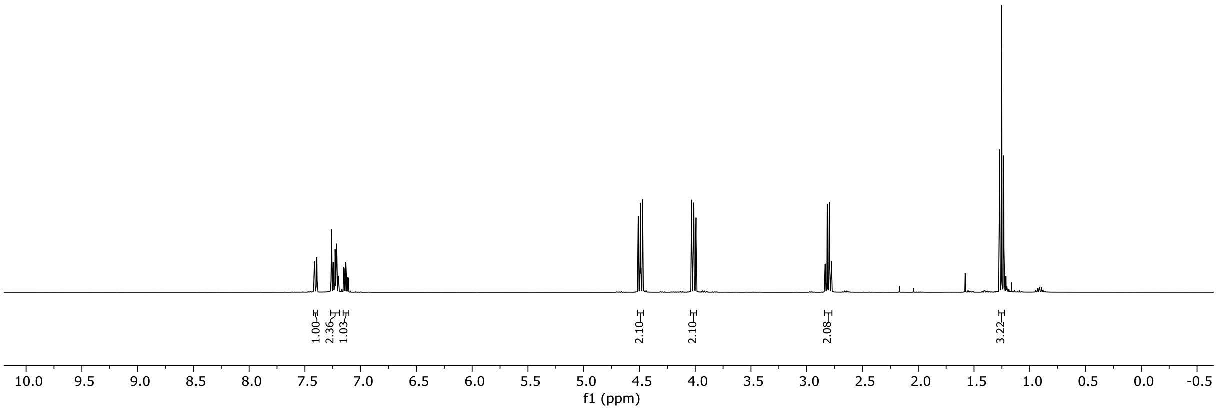
1.00
2.36
1.03
2.10
2.10
2.08
3.22
10.0 9.5 9.0 8.5 8.0 7.5 7.0 6.5 6.0 5.5 5.0 4.5 4.0 3.5 3.0 2.5 2.0 1.5 1.0 0.5 0.0 -0.5
f1 (ppm)

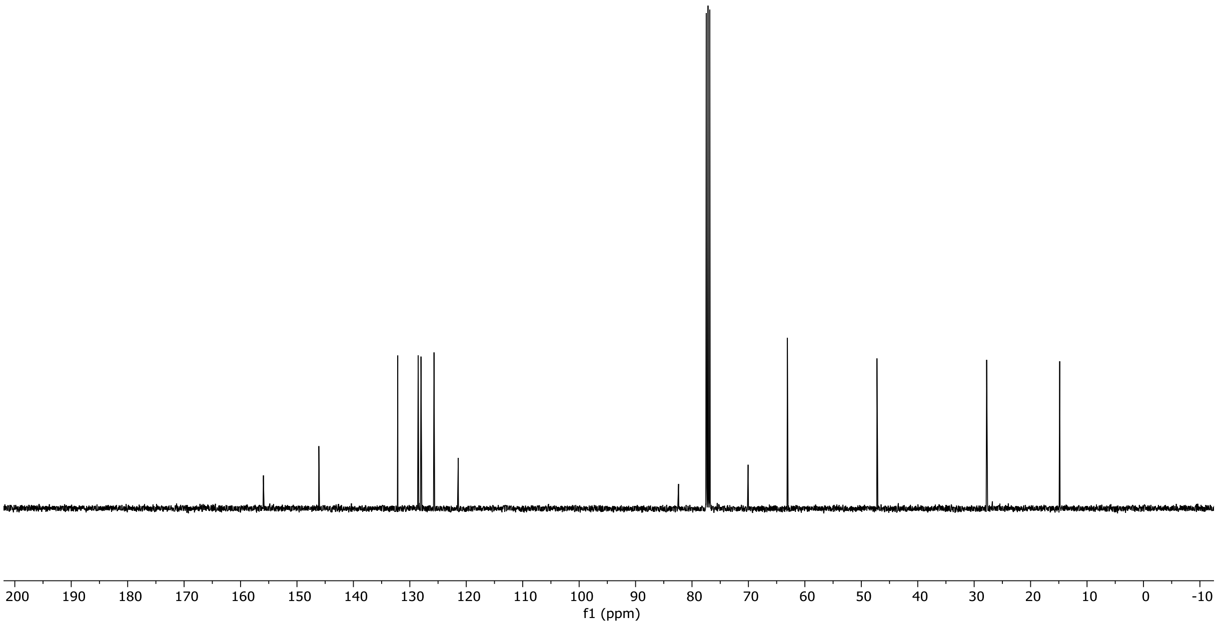
200 190 180 170 160 150 140 130 120 110 100 90 80 70 60 50 40 30 20 10 0 -10
f1 (ppm)

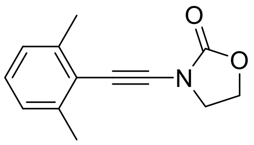
O
N
O

1e

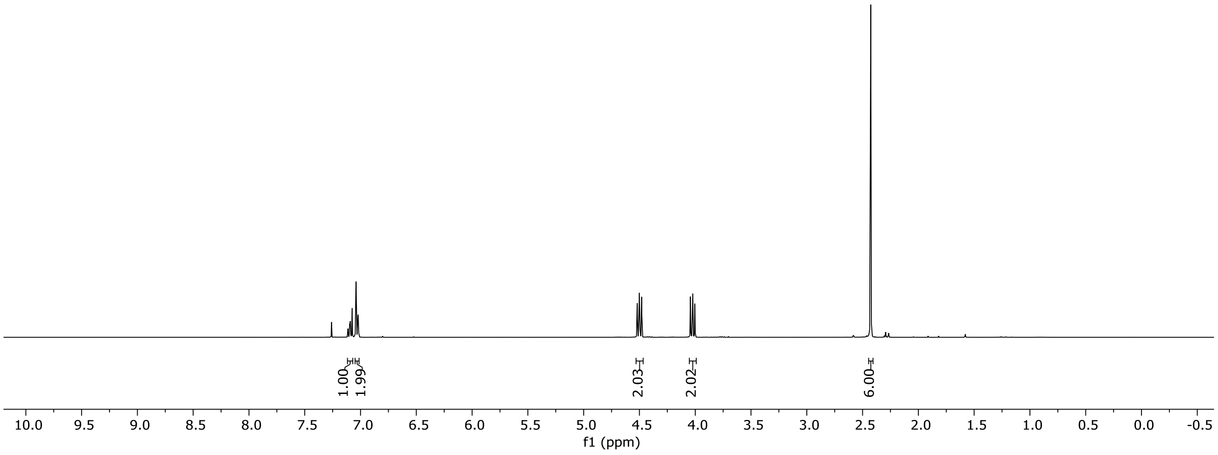
1.00
1.99
2.03
2.02
6.00
10.0 9.5 9.0 8.5 8.0 7.5 7.0 6.5 6.0 5.5 5.0 4.5 4.0 3.5 3.0 2.5 2.0 1.5 1.0 0.5 0.0 -0.5
f1 (ppm)

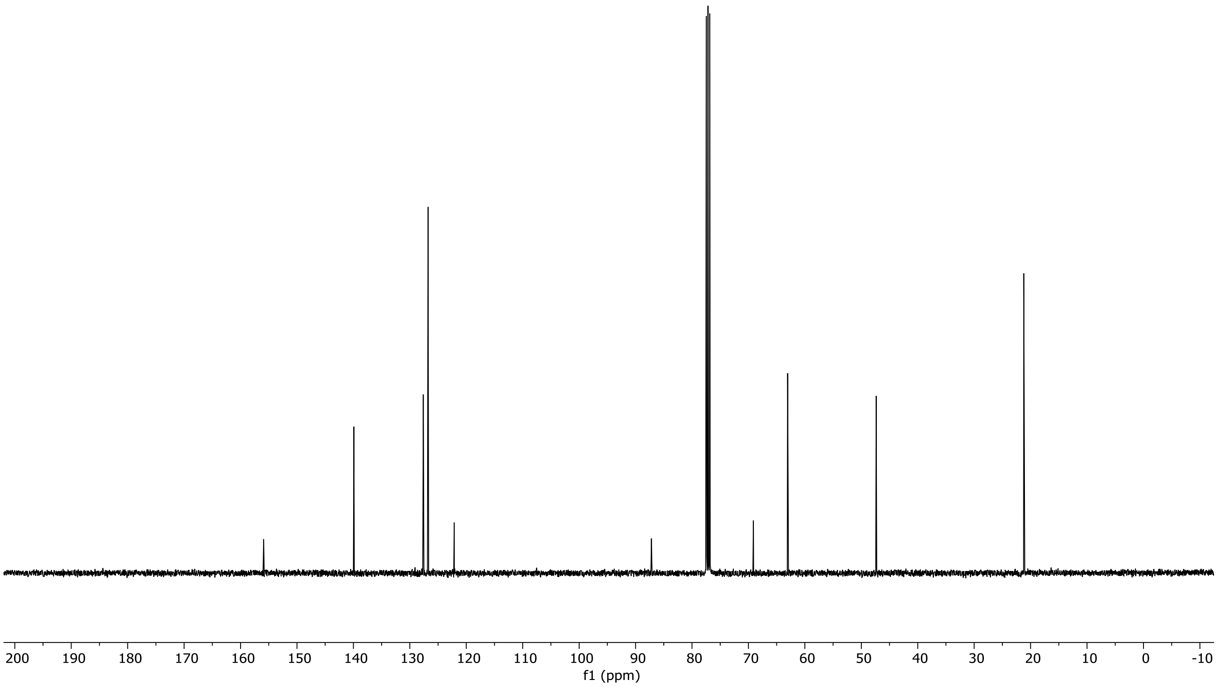
200 190 180 170 160 150 140 130 120 110 100 90 80 70 60 50 40 30 20 10 0 -10
f1 (ppm)

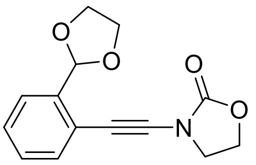
O
O
O
O
N

1f

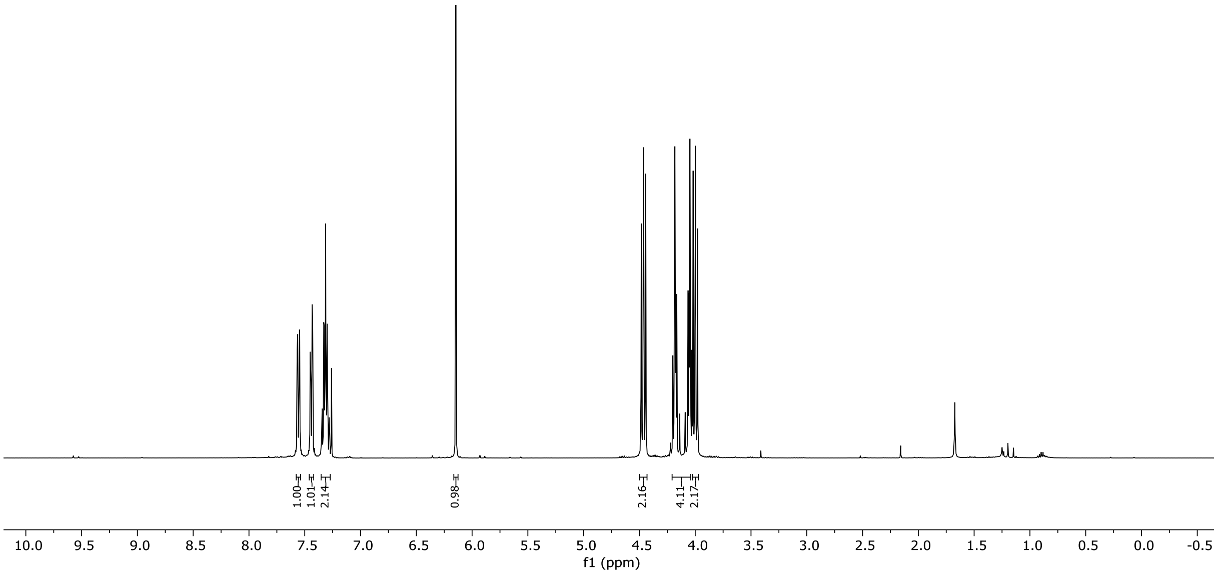
1.00
1.01
2.14
0.98
2.16
4.11
2.17
10.0 9.5 9.0 8.5 8.0 7.5 7.0 6.5 6.0 5.5 5.0 4.5 4.0 3.5 3.0 2.5 2.0 1.5 1.0 0.5 0.0 -0.5
f1 (ppm)

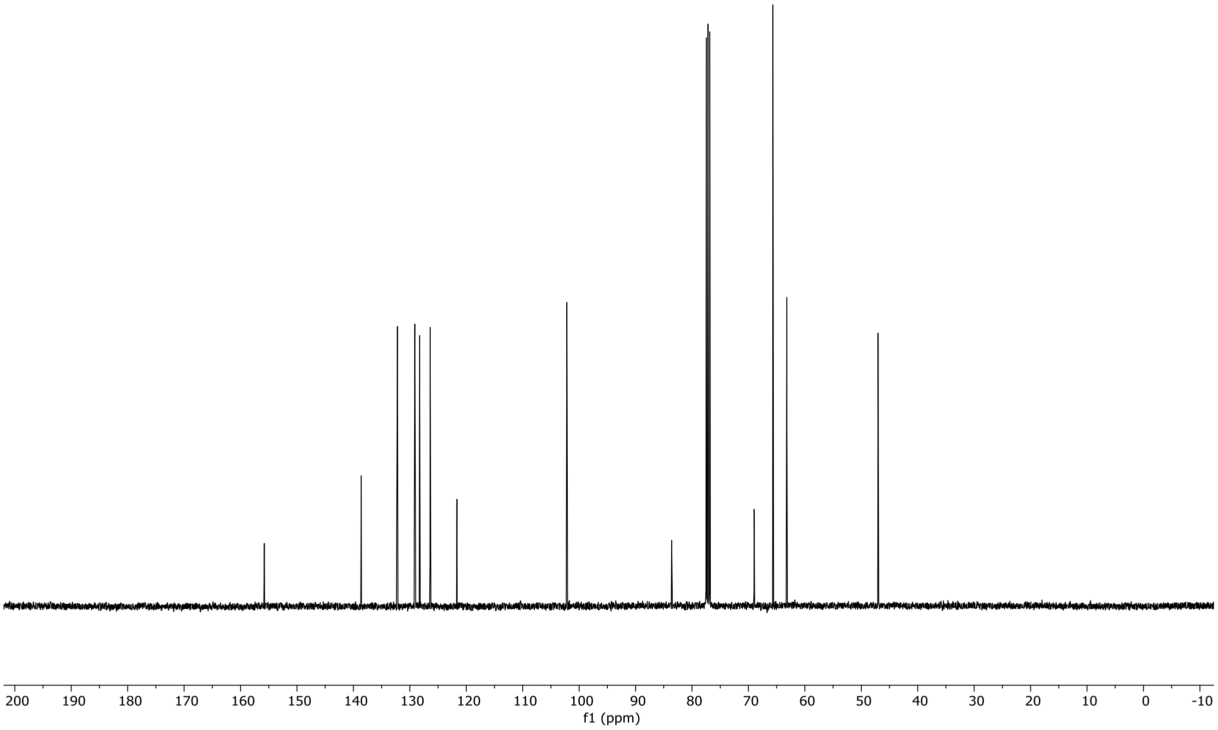
200 190 180 170 160 150 140 130 120 110 100 90 80 70 60 50 40 30 20 10 0 -10
f1 (ppm)

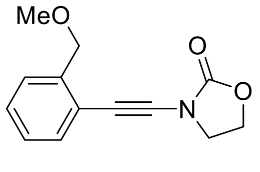
MeO
O
N
O

1g

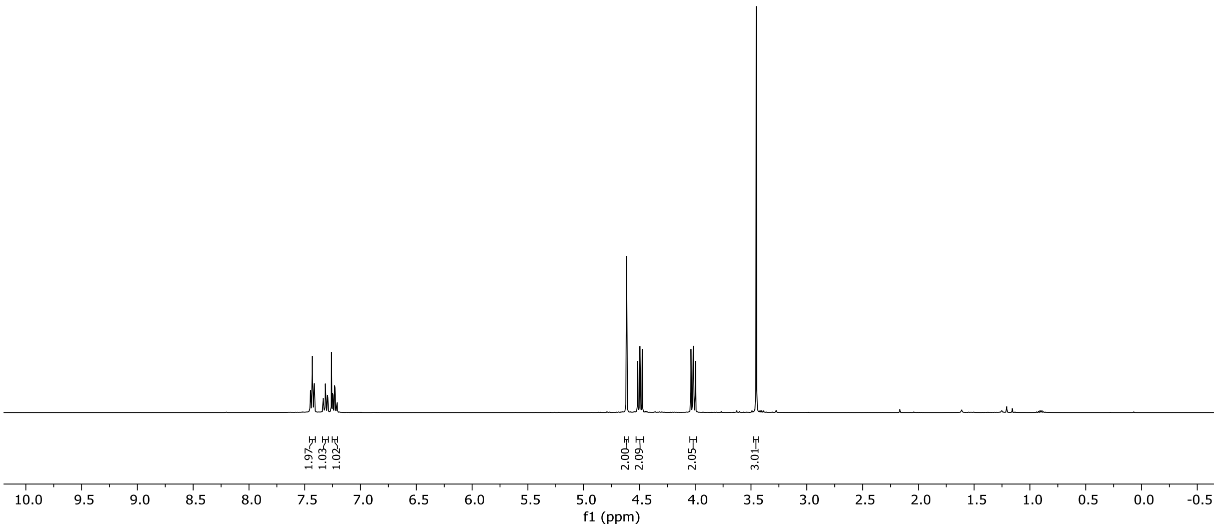
1.97
1.03
1.02
2.00
2.09
2.05
3.01
10.0 9.5 9.0 8.5 8.0 7.5 7.0 6.5 6.0 5.5 5.0 4.5 4.0 3.5 3.0 2.5 2.0 1.5 1.0 0.5 0.0 -0.5
f1 (ppm)

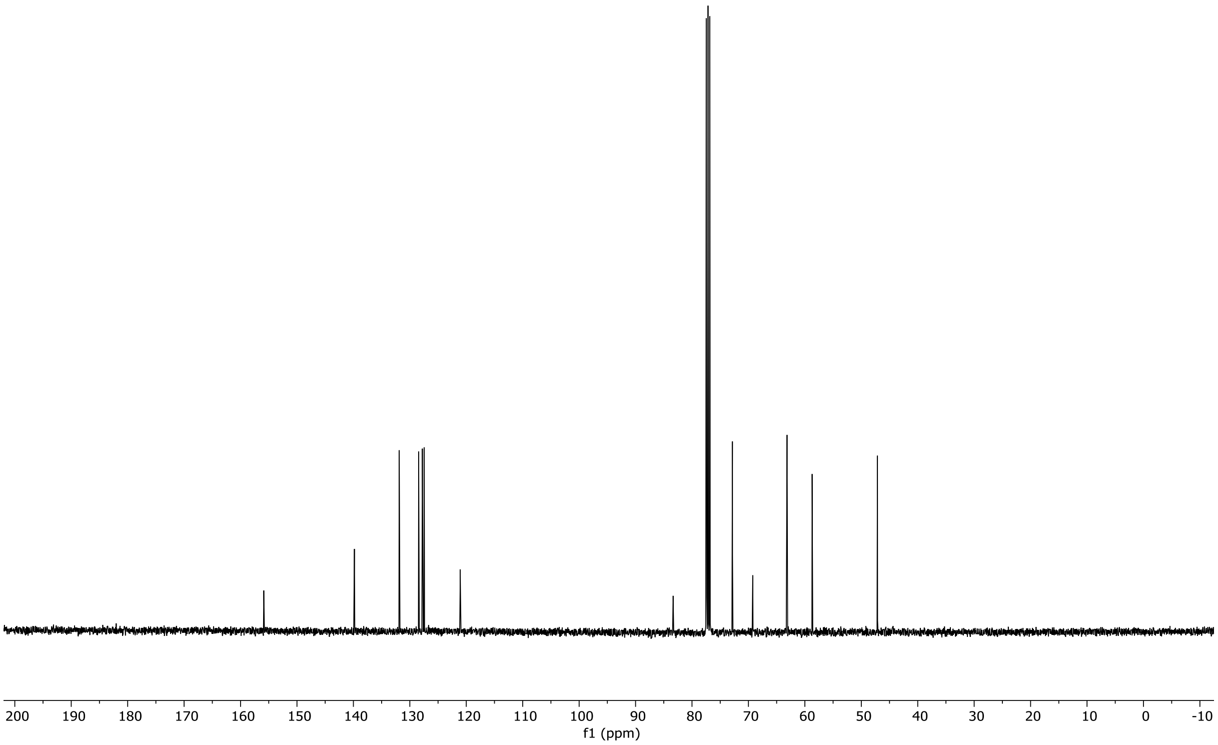
200 190 180 170 160 150 140 130 120 110 100 90 80 70 60 50 40 30 20 10 0 -10
f1 (ppm)

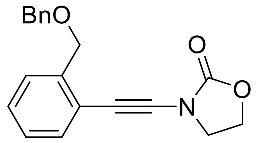
BnO
O
N
O

1h

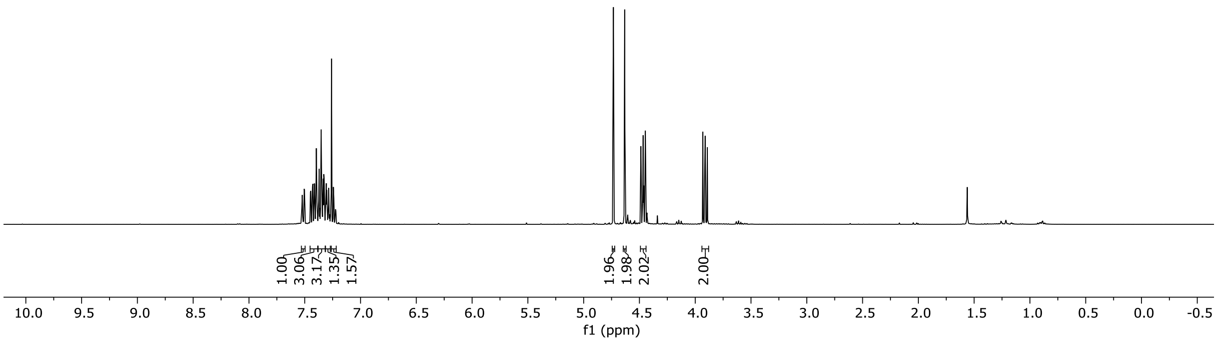
1.00
3.06
3.17
1.35
1.57
1.96
1.98
2.02
2.00
10.0 9.5 9.0 8.5 8.0 7.5 7.0 6.5 6.0 5.5 5.0 4.5 4.0 3.5 3.0 2.5 2.0 1.5 1.0 0.5 0.0 -0.5
f1 (ppm)

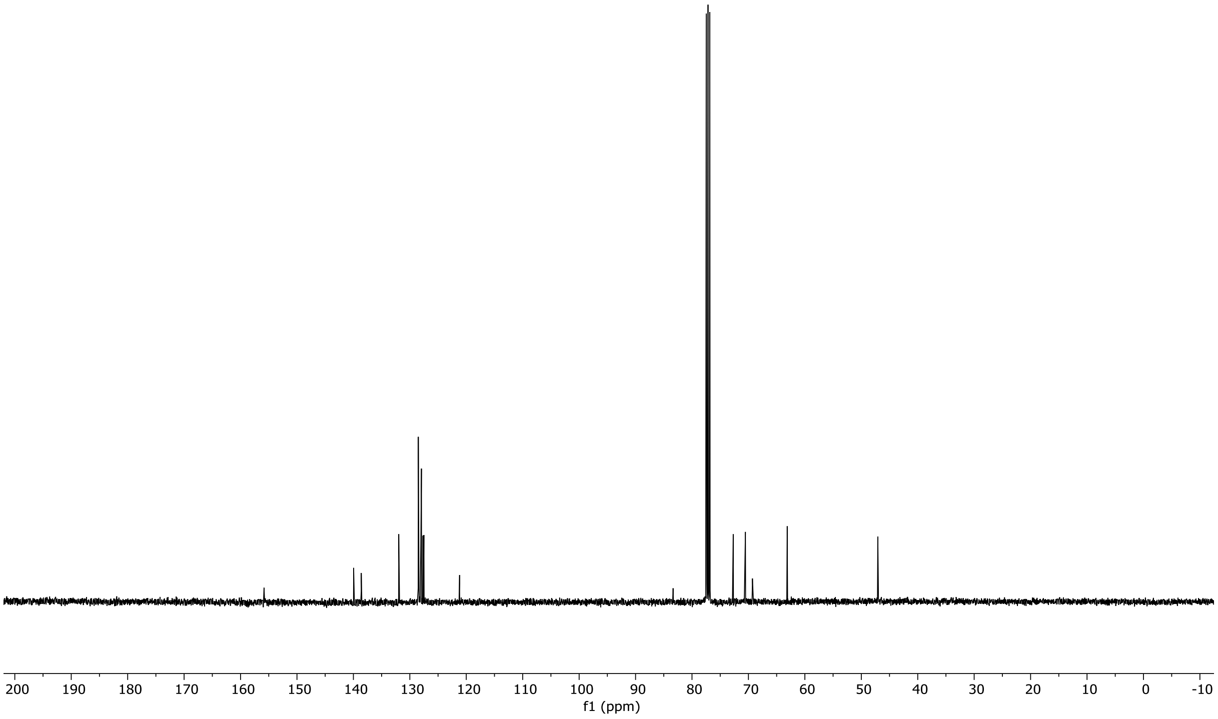
200 190 180 170 160 150 140 130 120 110 100 90 80 70 60 50 40 30 20 10 0 -10
f1 (ppm)

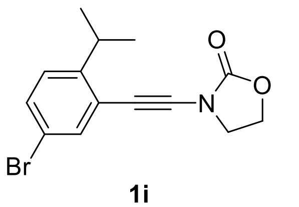
O
O
N
Br

1i

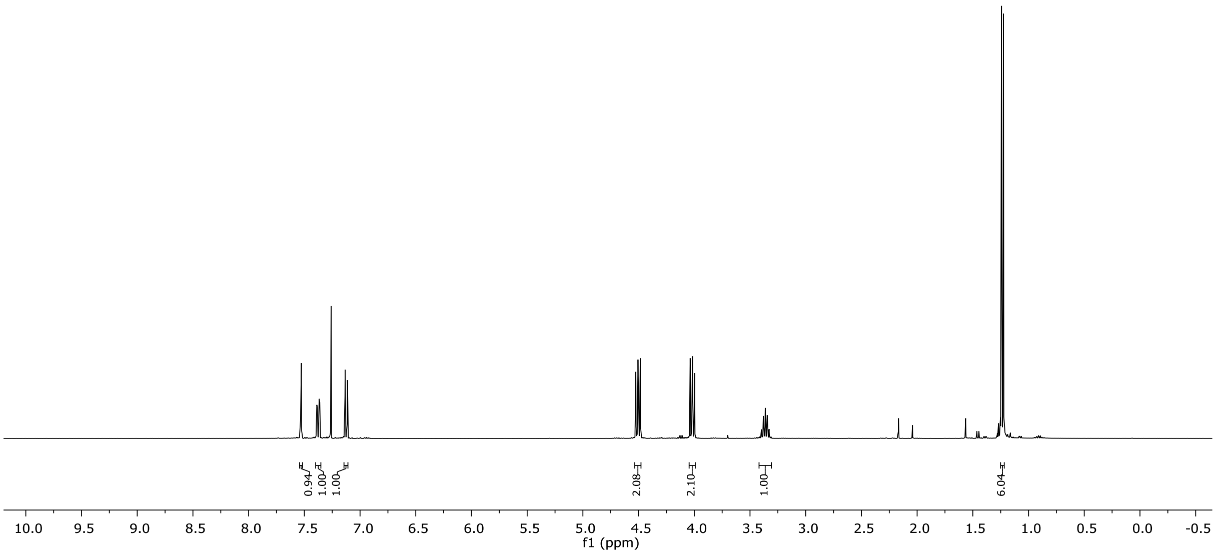
0.94
1.00
1.00
2.08
2.10
1.00
6.04
10.0
9.5
9.0
8.5
8.0
7.5
7.0
6.5
6.0
5.5
5.0
4.5
4.0
3.5
3.0
2.5
2.0
1.5
1.0
0.5
0.0
-0.5
f1 (ppm)

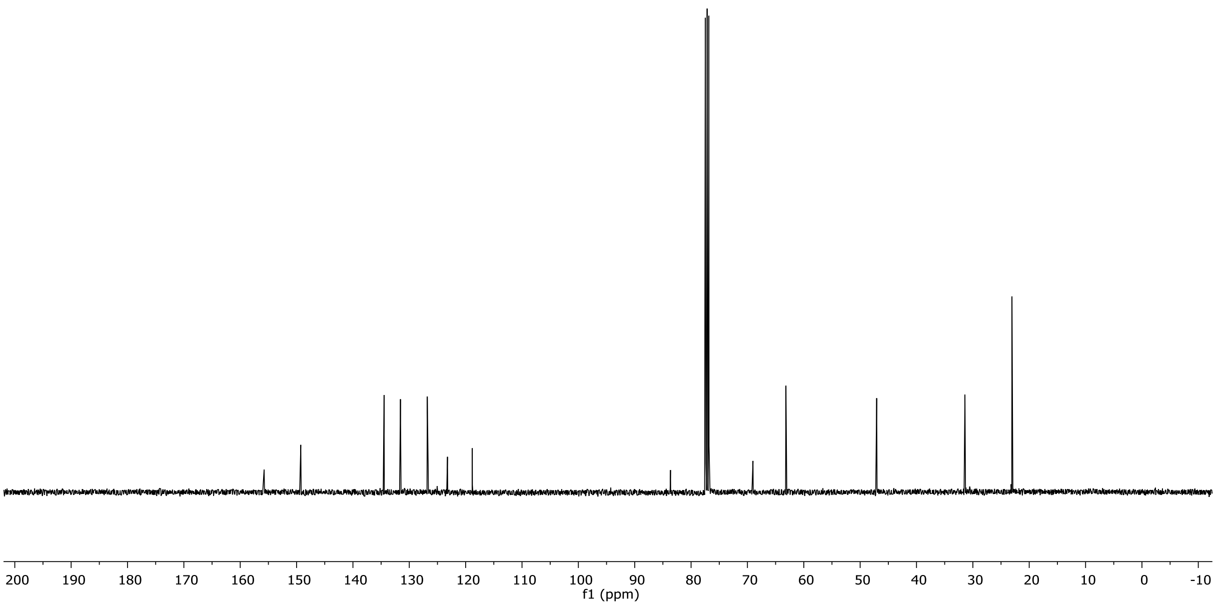
200
190
180
170
160
150
140
130
120
110
100
90
80
70
60
50
40
30
20
10
0
-10
f1 (ppm)

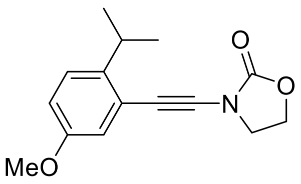
O
O
N
MeO

1j

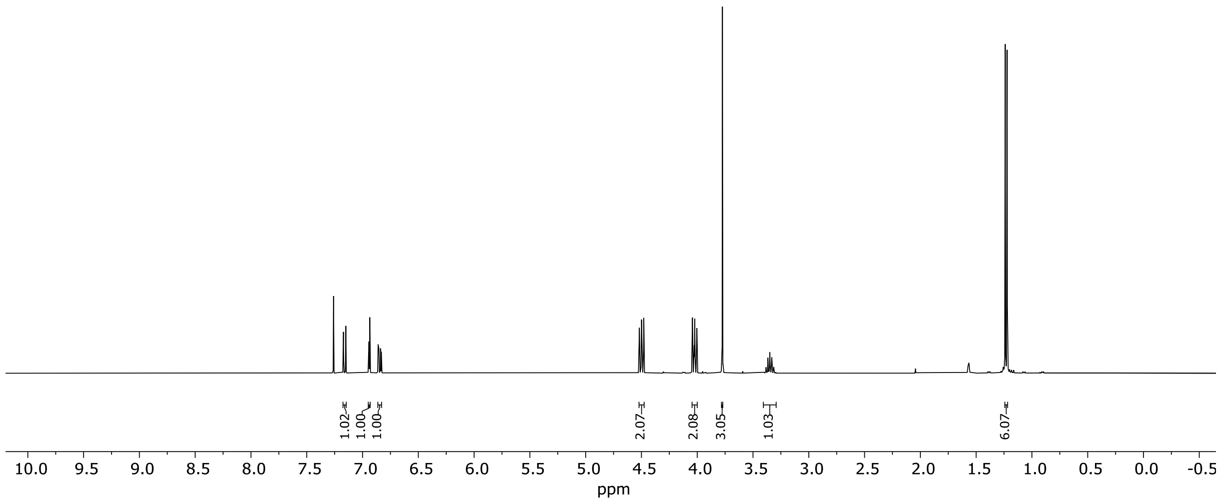
1.02
1.00
1.00
2.07
2.08
3.05
1.03
6.07
10.0 9.5 9.0 8.5 8.0 7.5 7.0 6.5 6.0 5.5 5.0 4.5 4.0 3.5 3.0 2.5 2.0 1.5 1.0 0.5 0.0 -0.5
ppm

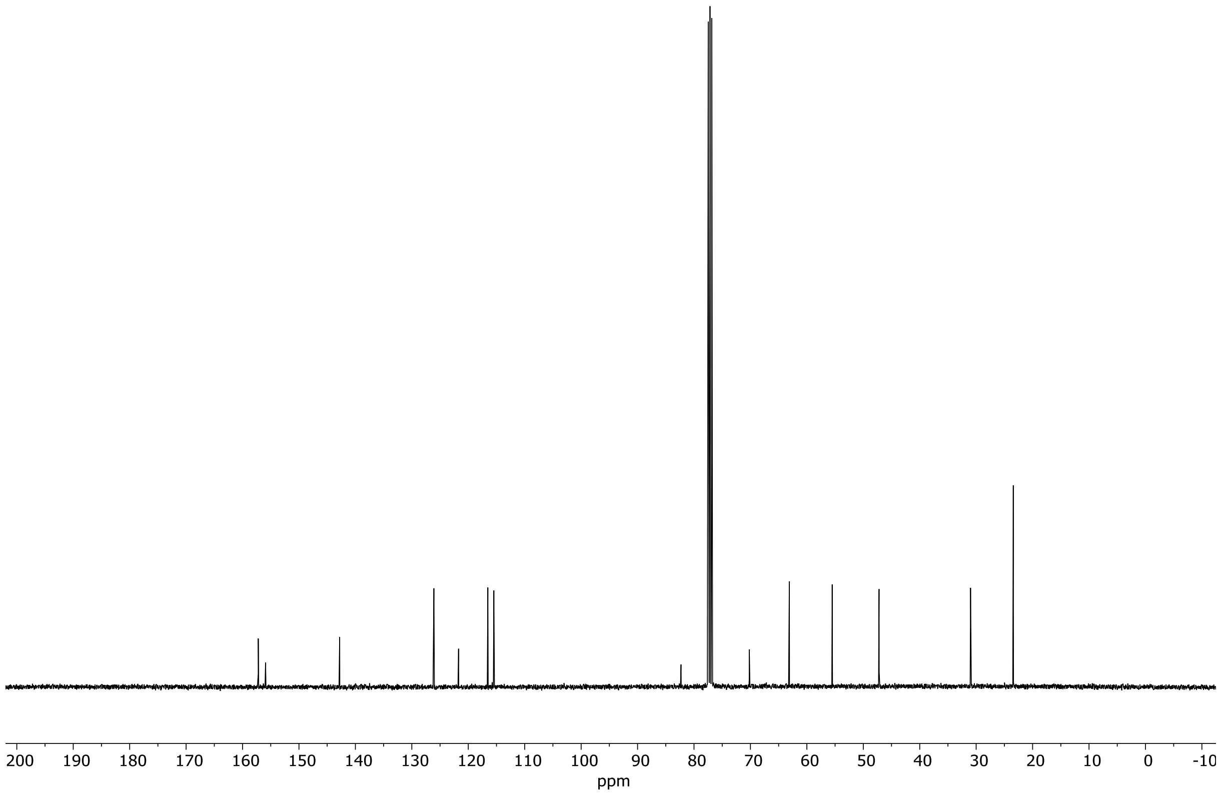
200 190 180 170 160 150 140 130 120 110 100 90 80 70 60 50 40 30 20 10 0 -10
ppm

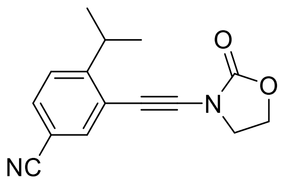
O
O
N
NC

1k

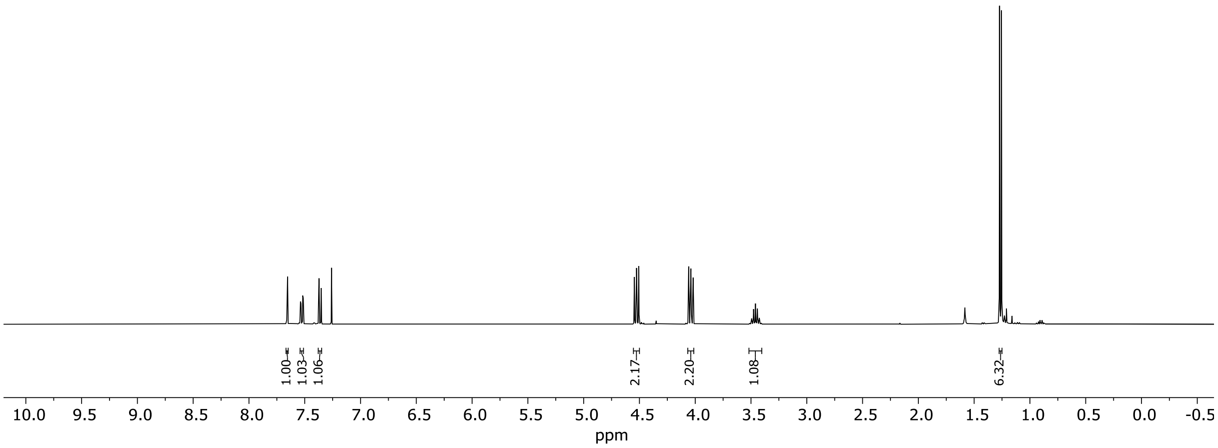
1.00
1.03
1.06
2.17
2.20
1.08
6.32
10.0 9.5 9.0 8.5 8.0 7.5 7.0 6.5 6.0 5.5 5.0 4.5 4.0 3.5 3.0 2.5 2.0 1.5 1.0 0.5 0.0 -0.5
ppm

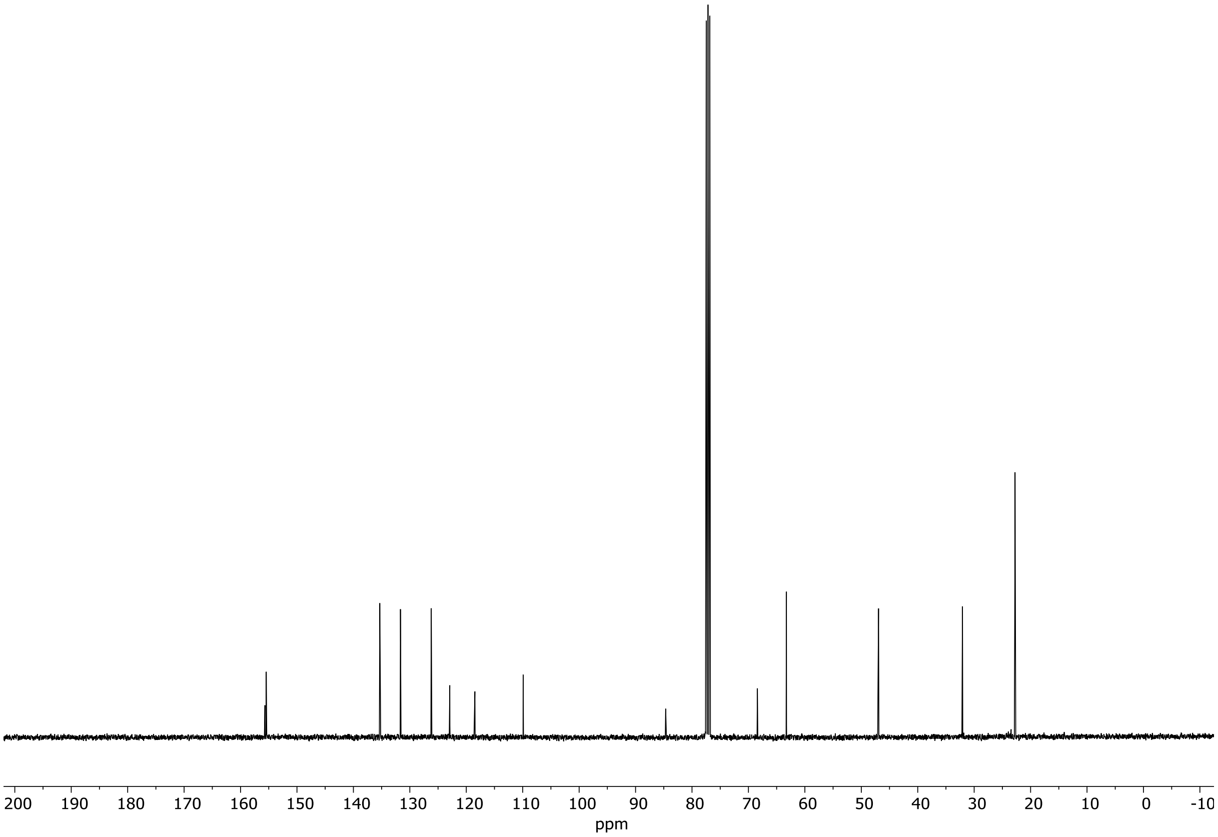
200 190 180 170 160 150 140 130 120 110 100 90 80 70 60 50 40 30 20 10 0 -10
ppm

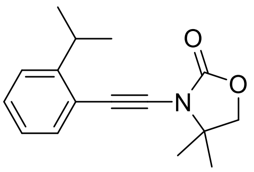
O
O
N

1l

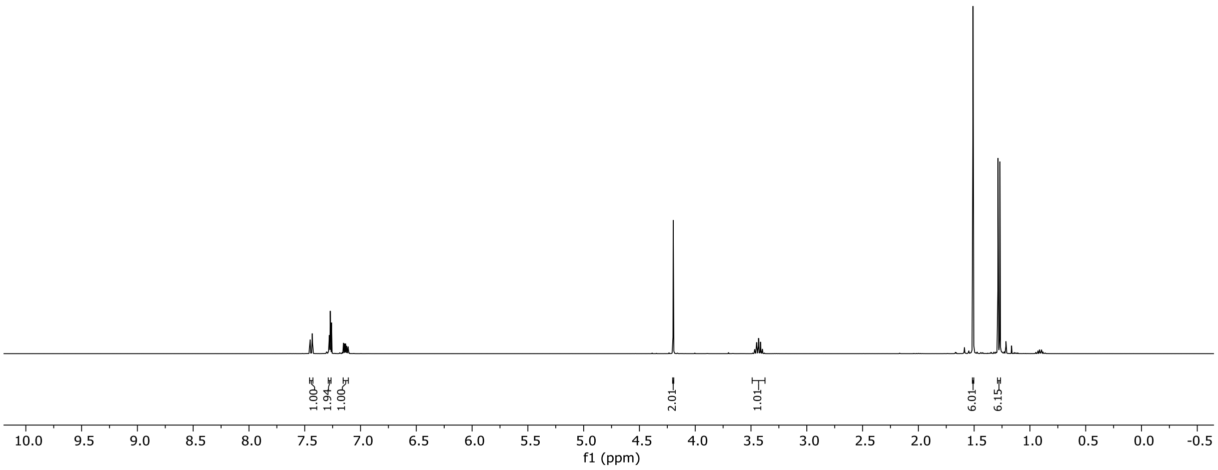
1.00
1.94
1.00
2.01
1.01
6.01
6.15
10.0 9.5 9.0 8.5 8.0 7.5 7.0 6.5 6.0 5.5 5.0 4.5 4.0 3.5 3.0 2.5 2.0 1.5 1.0 0.5 0.0 -0.5
f1 (ppm)

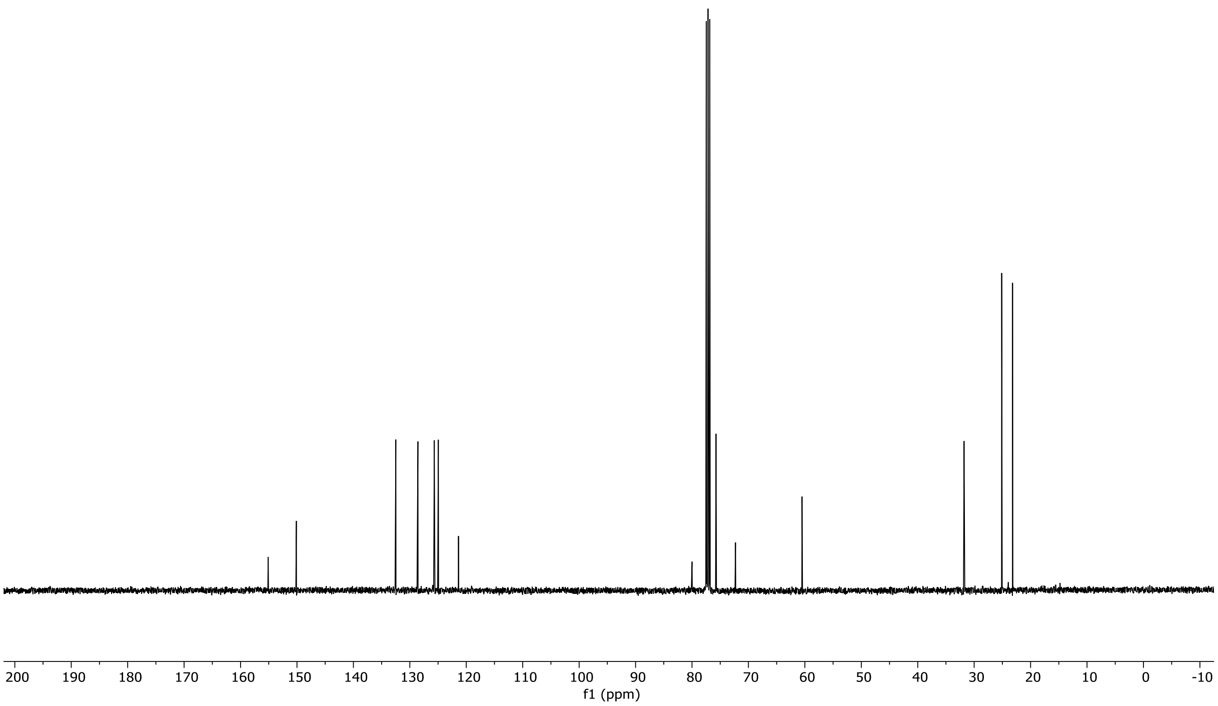
200 190 180 170 160 150 140 130 120 110 100 90 80 70 60 50 40 30 20 10 0 -10
f1 (ppm)

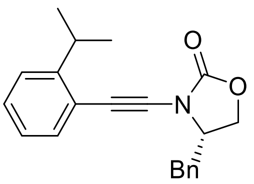
O
N
O
Bn

1m

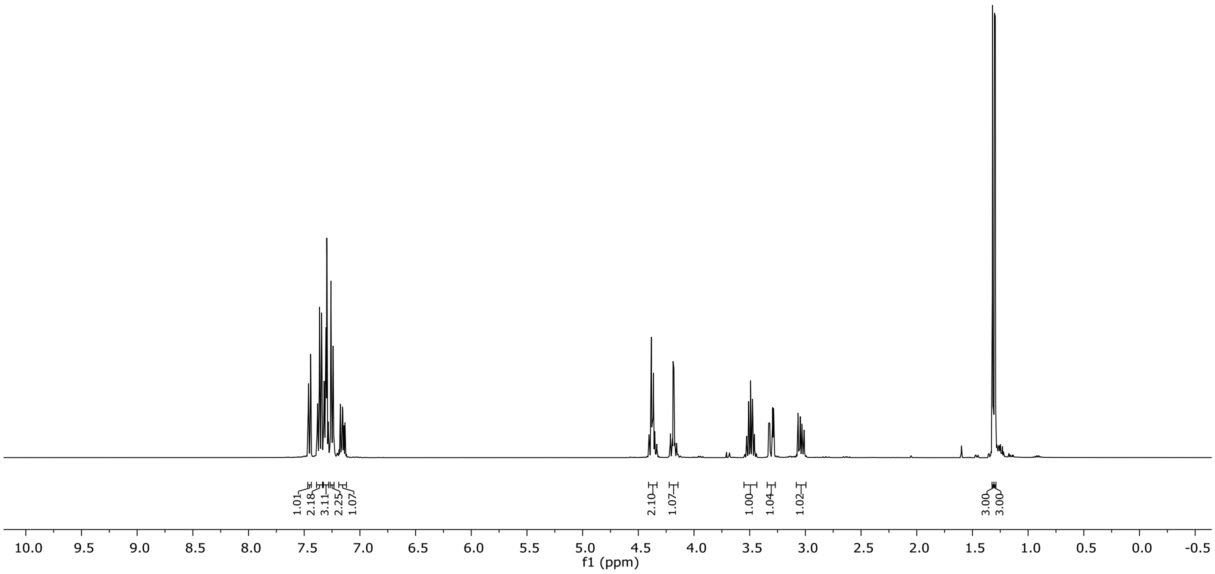
1.01
2.18
3.11
2.25
1.07
2.10
1.07
1.00
1.04
1.02
3.00
3.00
10.0 9.5 9.0 8.5 8.0 7.5 7.0 6.5 6.0 5.5 5.0 4.5 4.0 3.5 3.0 2.5 2.0 1.5 1.0 0.5 0.0 -0.5
f1 (ppm)

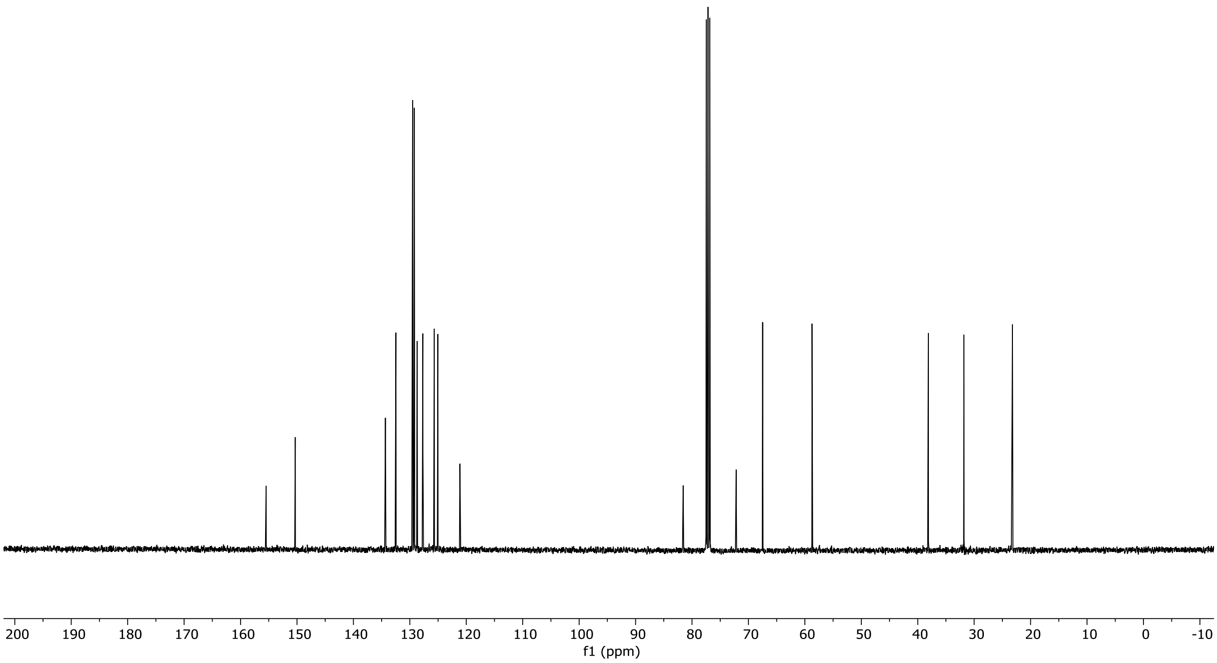
200 190 180 170 160 150 140 130 120 110 100 90 80 70 60 50 40 30 20 10 0 -10
f1 (ppm)

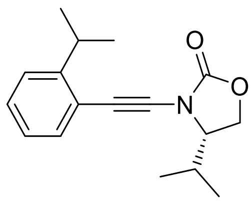
O
N
O

1n

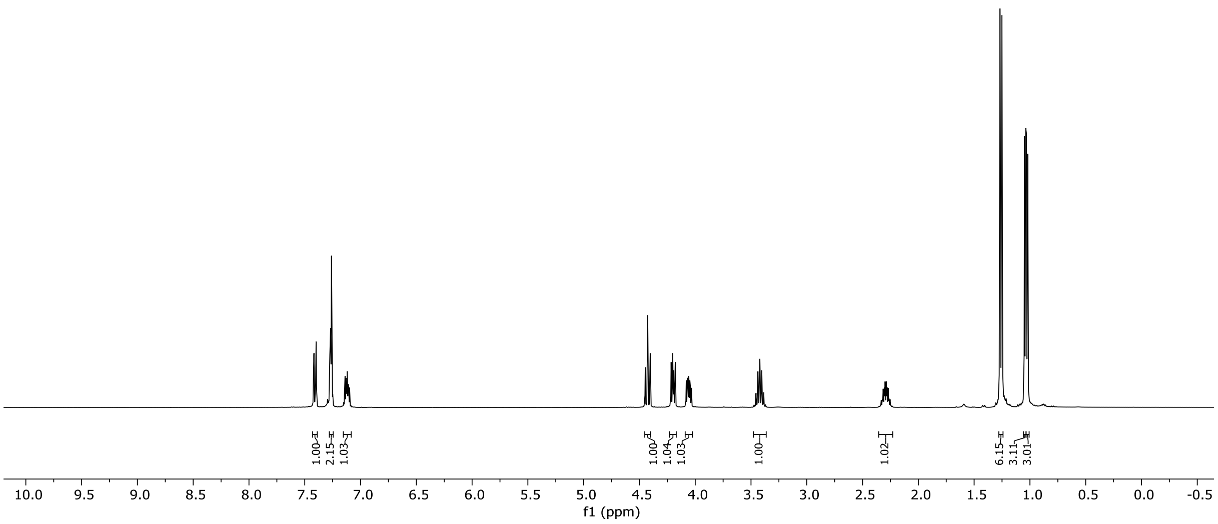
1.00
2.15
1.03
1.00
1.04
1.03
1.00
1.02
6.15
3.11
3.01
10.0
9.5
9.0
8.5
8.0
7.5
7.0
6.5
6.0
5.5
5.0
4.5
4.0
3.5
3.0
2.5
2.0
1.5
1.0
0.5
0.0
-0.5
f1 (ppm)

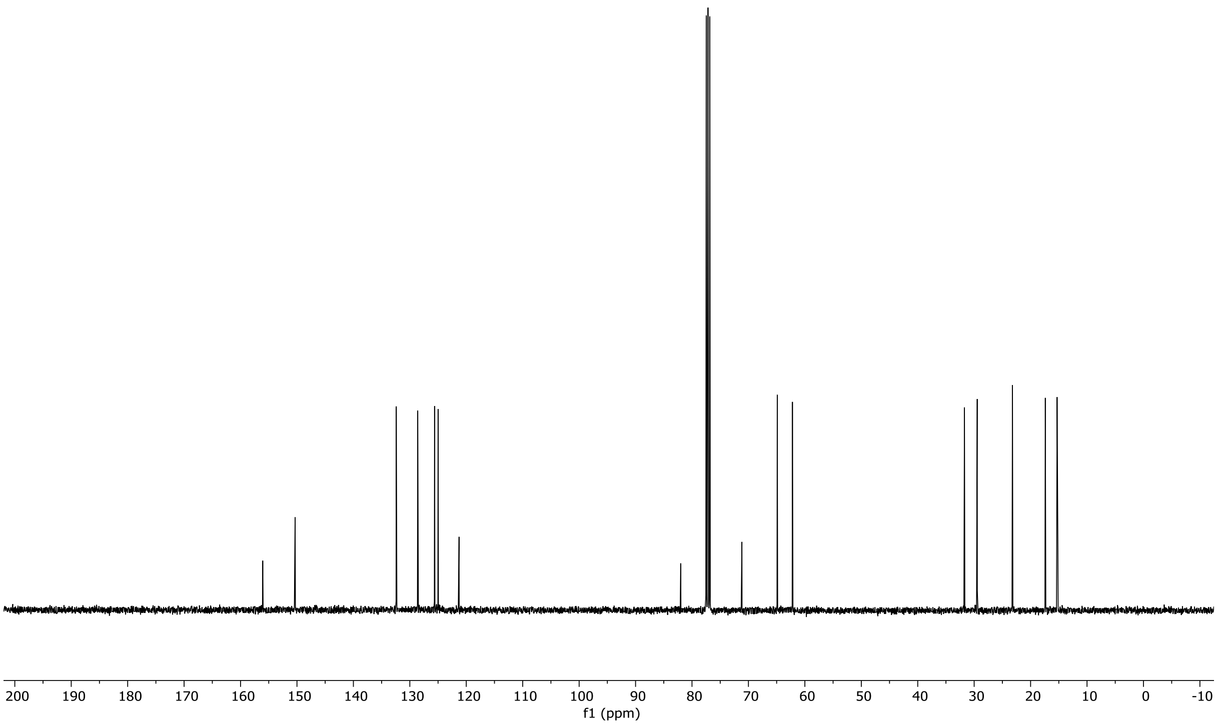
200
190
180
170
160
150
140
130
120
110
100
90
80
70
60
50
40
30
20
10
0
-10
f1 (ppm)

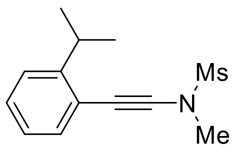
Ms
N
Me

1o

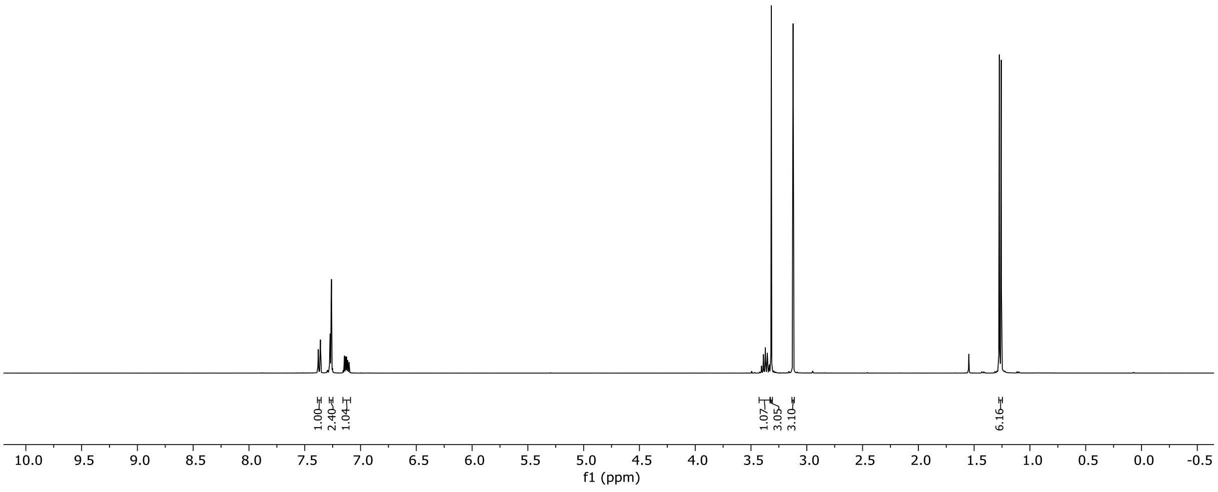
1.00
2.40
1.04
1.07
3.05
3.10
6.16
10.0
9.5
9.0
8.5
8.0
7.5
7.0
6.5
6.0
5.5
5.0
4.5
4.0
3.5
3.0
2.5
2.0
1.5
1.0
0.5
0.0
-0.5
f1 (ppm)

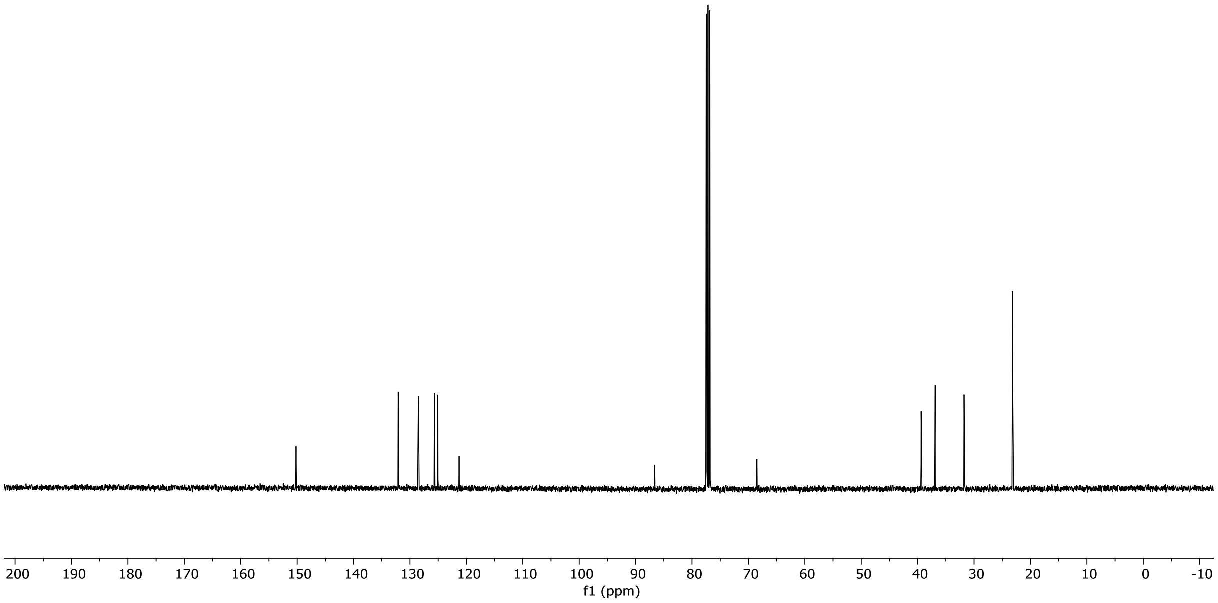
200
190
180
170
160
150
140
130
120
110
100
90
80
70
60
50
40
30
20
10
0
-10
f1 (ppm)

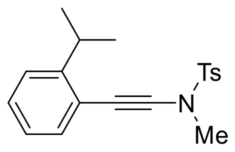
Ts
N
Me

1p

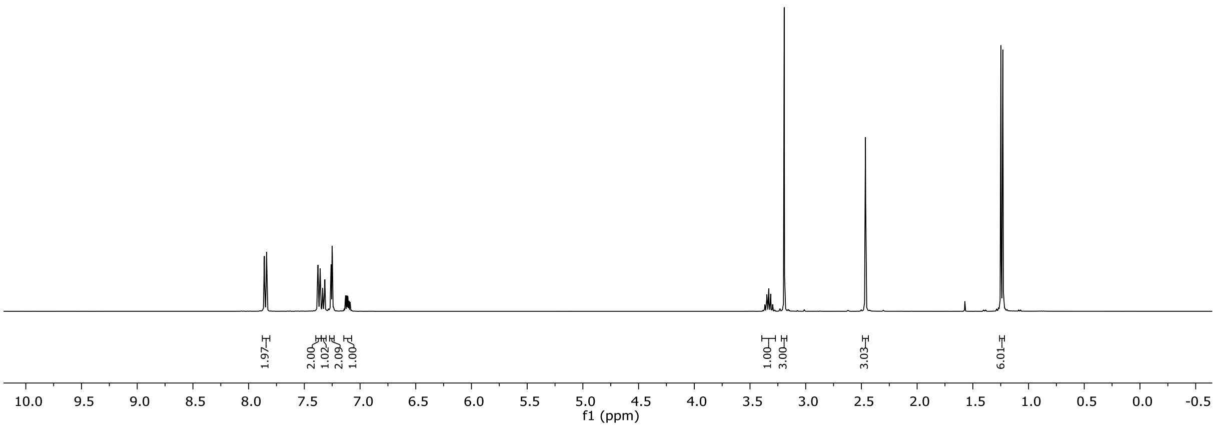
1.97
2.00
1.02
2.09
1.00
1.00
3.00
3.03
6.01
10.0
9.5
9.0
8.5
8.0
7.5
7.0
6.5
6.0
5.5
5.0
4.5
4.0
3.5
3.0
2.5
2.0
1.5
1.0
0.5
0.0
-0.5
f1 (ppm)

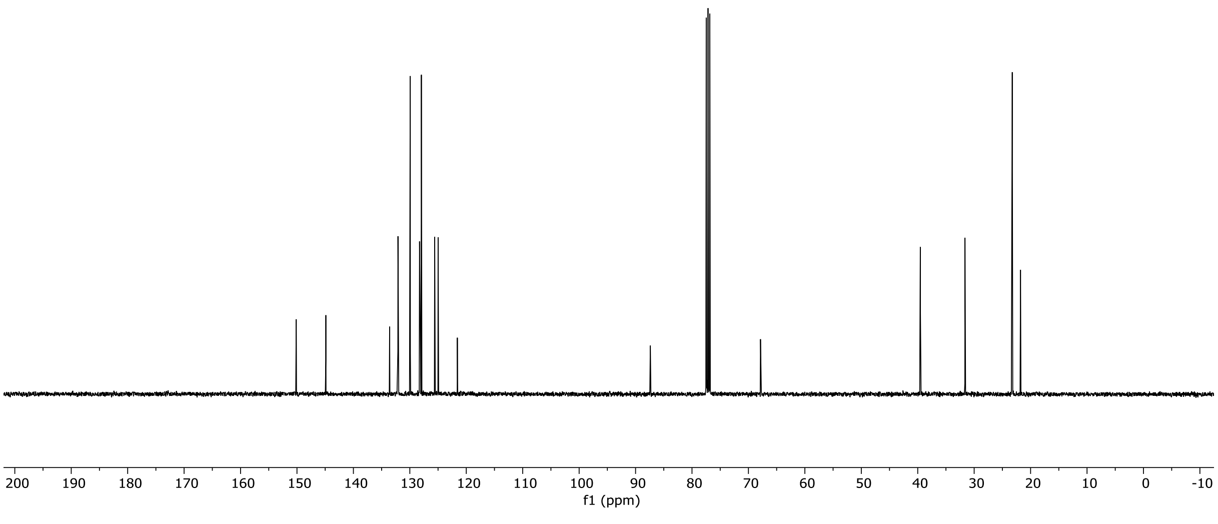
200
190
180
170
160
150
140
130
120
110
100
90
80
70
60
50
40
30
20
10
0
-10
f1 (ppm)

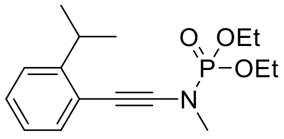
O
OEt
P
OEt
N

1q

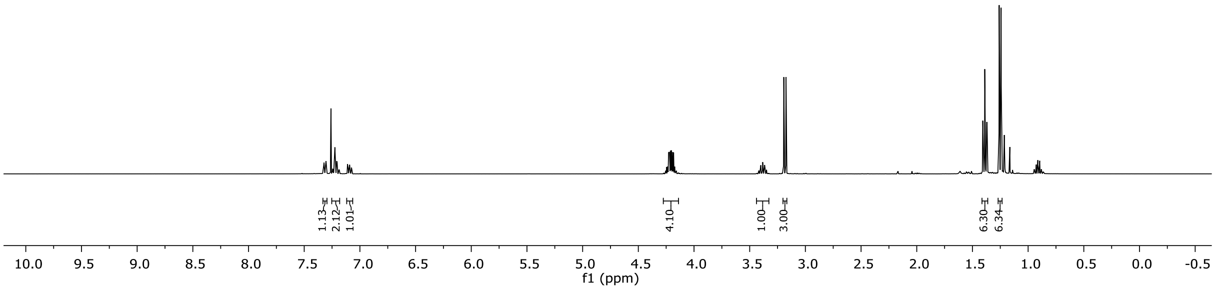
1.13
2.12
1.01
4.10
1.00
3.00
6.30
6.34
10.0 9.5 9.0 8.5 8.0 7.5 7.0 6.5 6.0 5.5 5.0 4.5 4.0 3.5 3.0 2.5 2.0 1.5 1.0 0.5 0.0 -0.5
f1 (ppm)

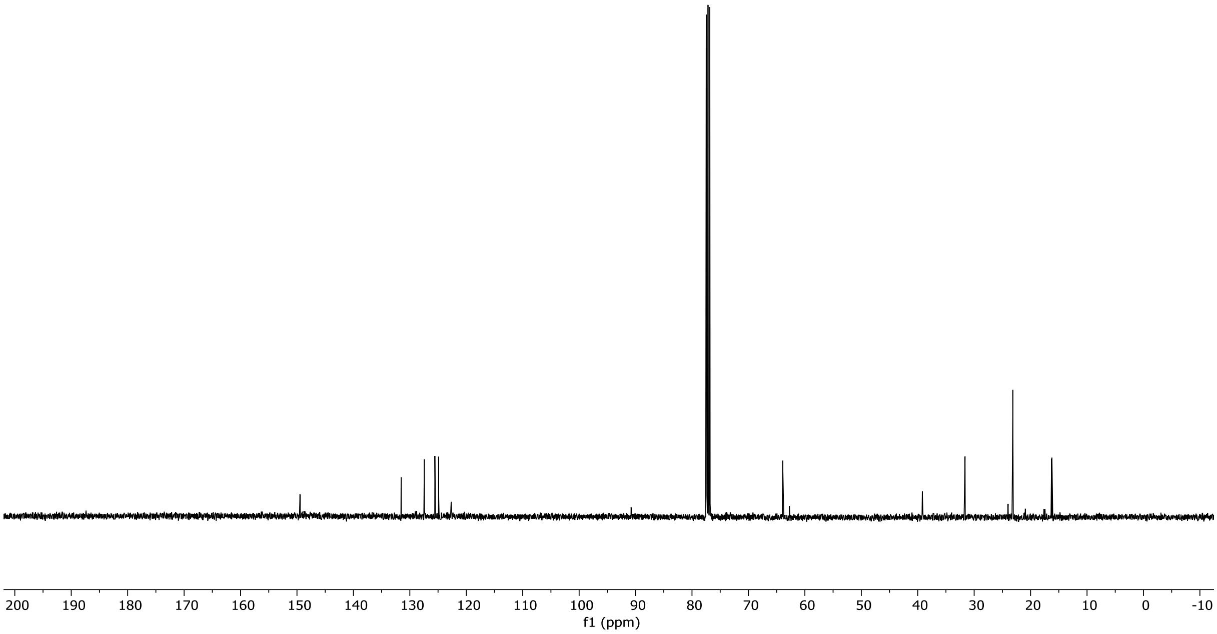
200 190 180 170 160 150 140 130 120 110 100 90 80 70 60 50 40 30 20 10 0 -10
f1 (ppm)

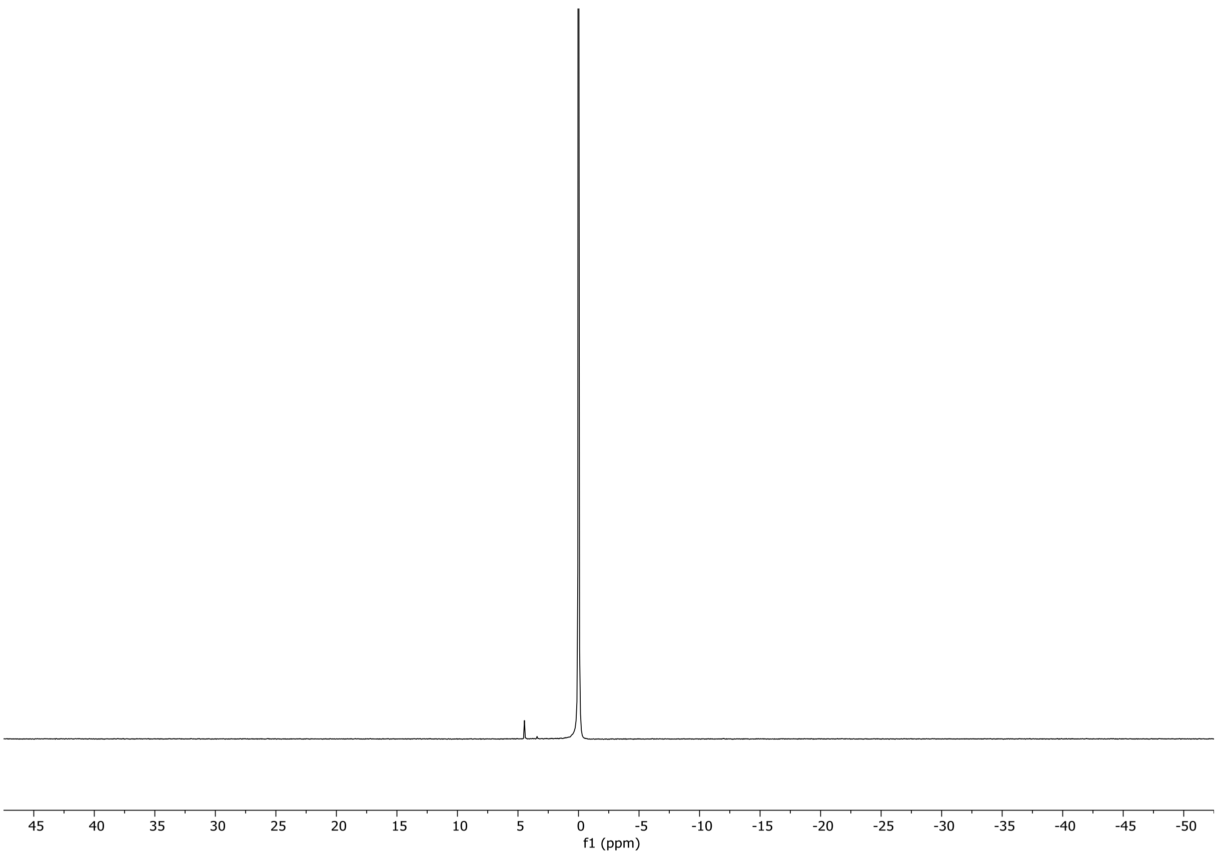
45 40 35 30 25 20 15 10 5 0 -5 -10 -15 -20 -25 -30 -35 -40 -45 -50
f1 (ppm)

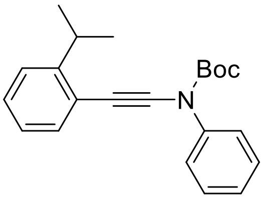
Boc
N

1r

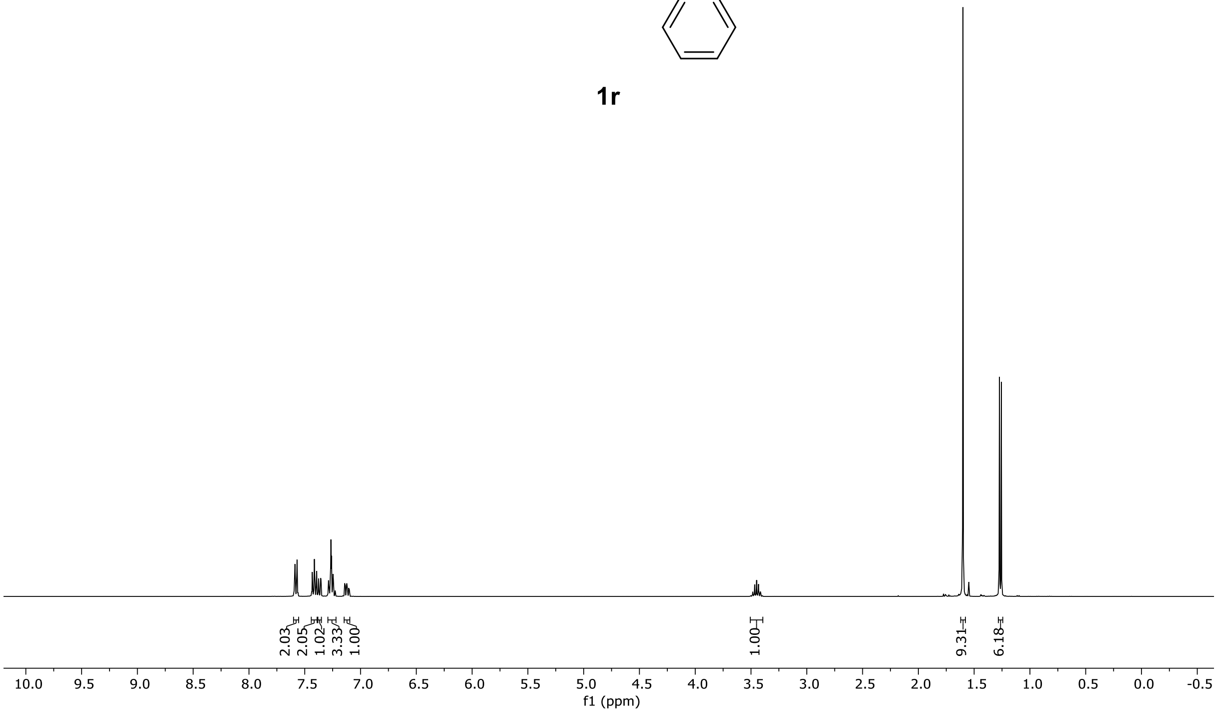
2.03
2.05
1.02
3.33
1.00
1.00
9.31
6.18
10.0 9.5 9.0 8.5 8.0 7.5 7.0 6.5 6.0 5.5 5.0 4.5 4.0 3.5 3.0 2.5 2.0 1.5 1.0 0.5 0.0 -0.5
f1 (ppm)

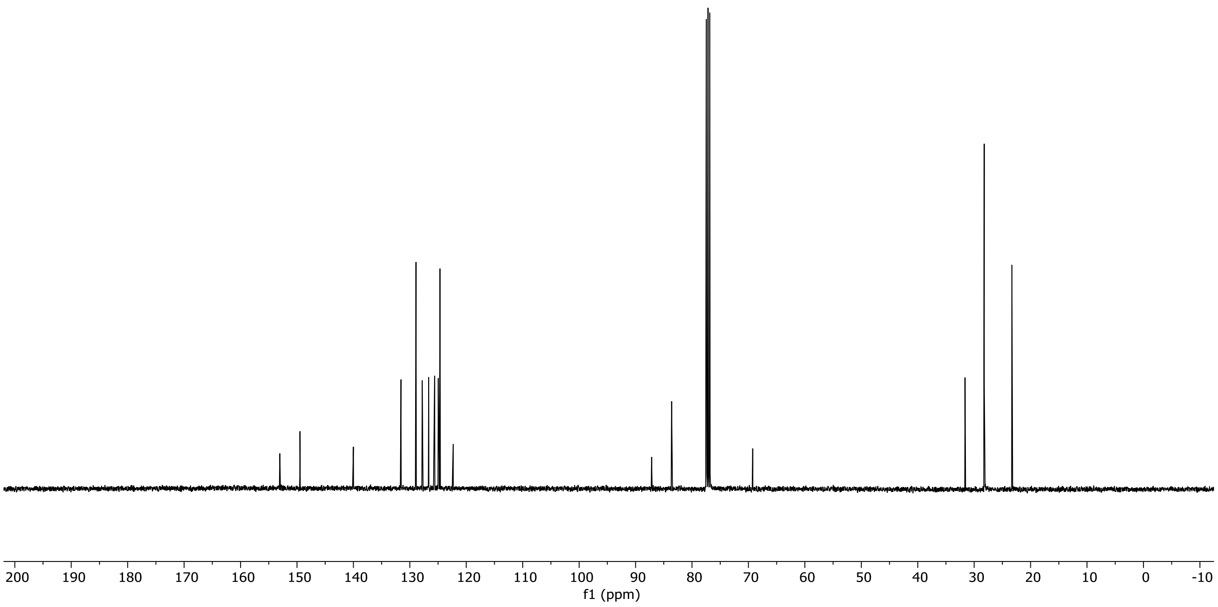
200 190 180 170 160 150 140 130 120 110 100 90 80 70 60 50 40 30 20 10 0 -10
f1 (ppm)

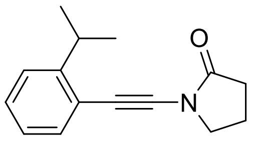
O
N

1s

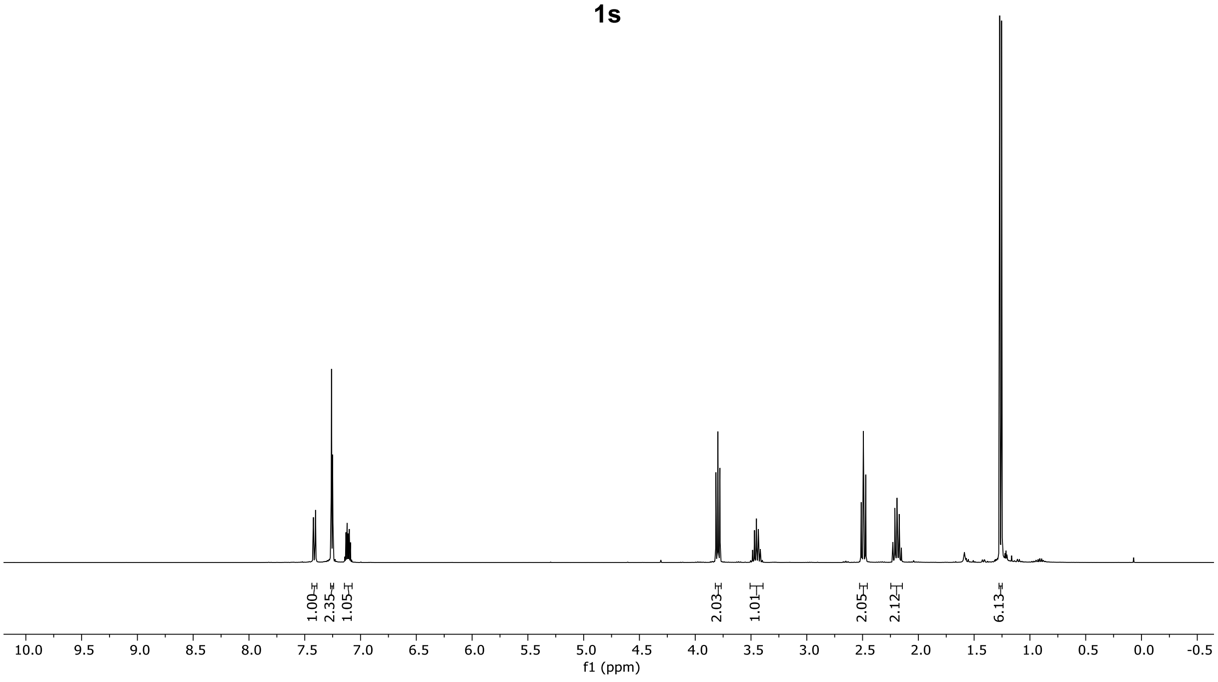
1.00
2.35
1.05
2.03
1.01
2.05
2.12
6.13
10.0 9.5 9.0 8.5 8.0 7.5 7.0 6.5 6.0 5.5 5.0 4.5 4.0 3.5 3.0 2.5 2.0 1.5 1.0 0.5 0.0 -0.5
f1 (ppm)

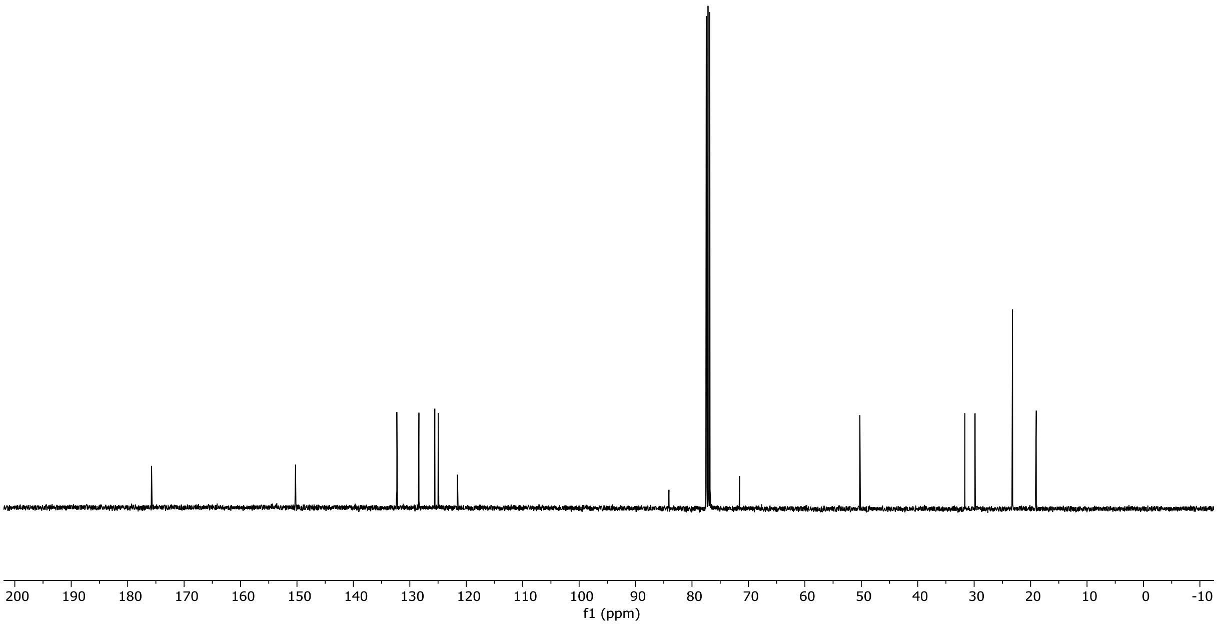
200 190 180 170 160 150 140 130 120 110 100 90 80 70 60 50 40 30 20 10 0 -10
f1 (ppm)

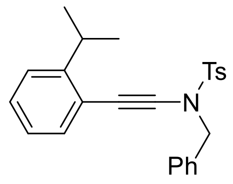
Ts
N
Ph

1t

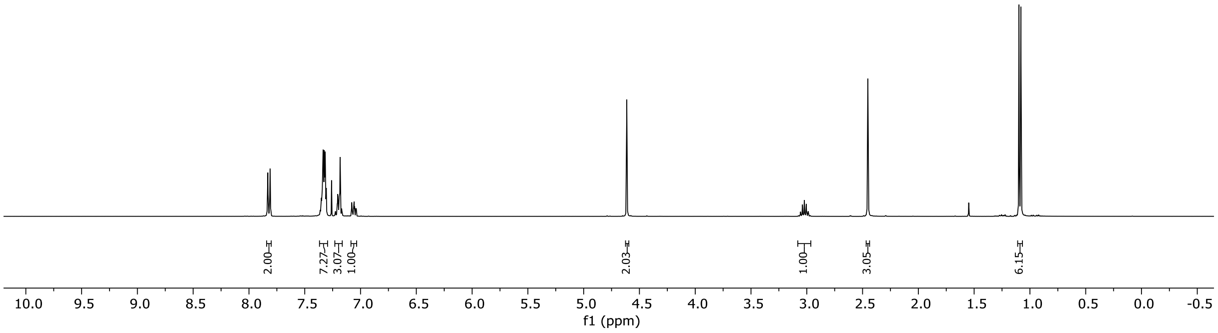
2.00
7.27
3.07
1.00
2.03
1.00
3.05
6.15
10.0 9.5 9.0 8.5 8.0 7.5 7.0 6.5 6.0 5.5 5.0 4.5 4.0 3.5 3.0 2.5 2.0 1.5 1.0 0.5 0.0 -0.5
f1 (ppm)

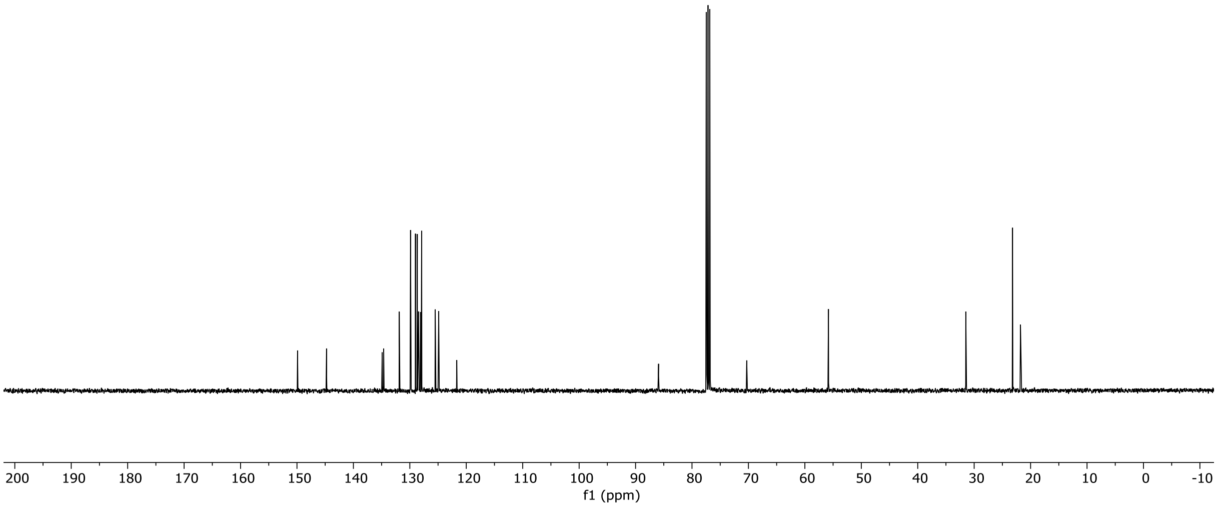
200 190 180 170 160 150 140 130 120 110 100 90 80 70 60 50 40 30 20 10 0 -10
f1 (ppm)

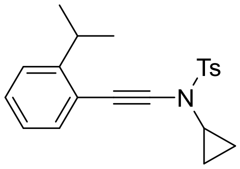
Ts
N

1u

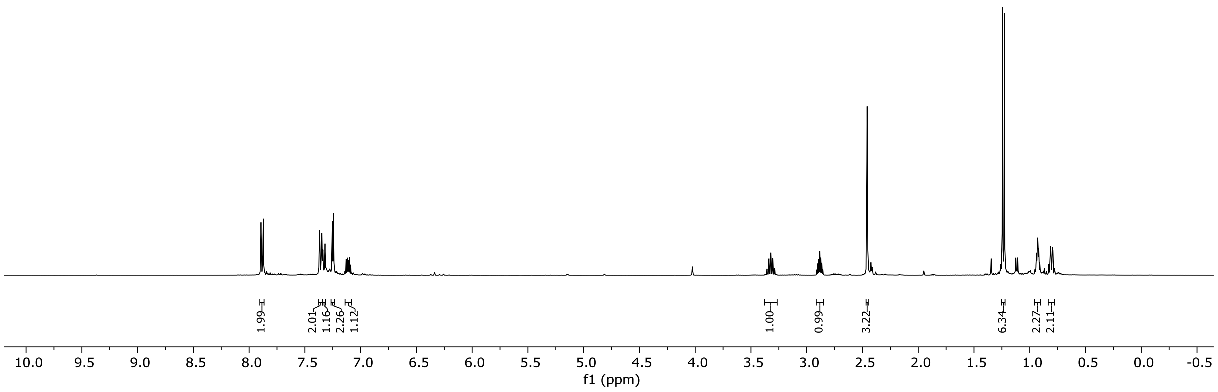
1.99
2.01
1.16
2.26
1.12
1.00
0.99
3.22
6.34
2.27
2.11
10.0 9.5 9.0 8.5 8.0 7.5 7.0 6.5 6.0 5.5 5.0 4.5 4.0 3.5 3.0 2.5 2.0 1.5 1.0 0.5 0.0 -0.5
f1 (ppm)

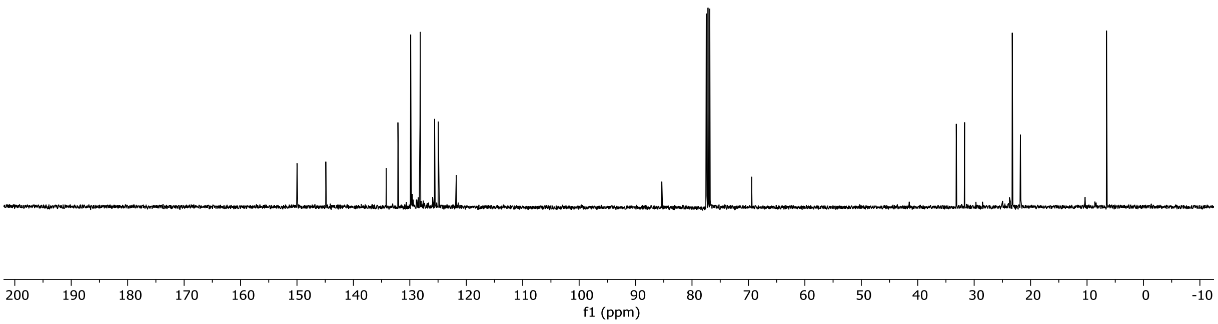
200 190 180 170 160 150 140 130 120 110 100 90 80 70 60 50 40 30 20 10 0 -10
f1 (ppm)

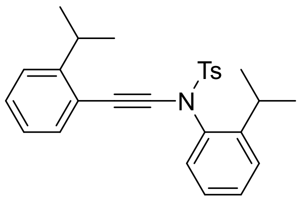

1v

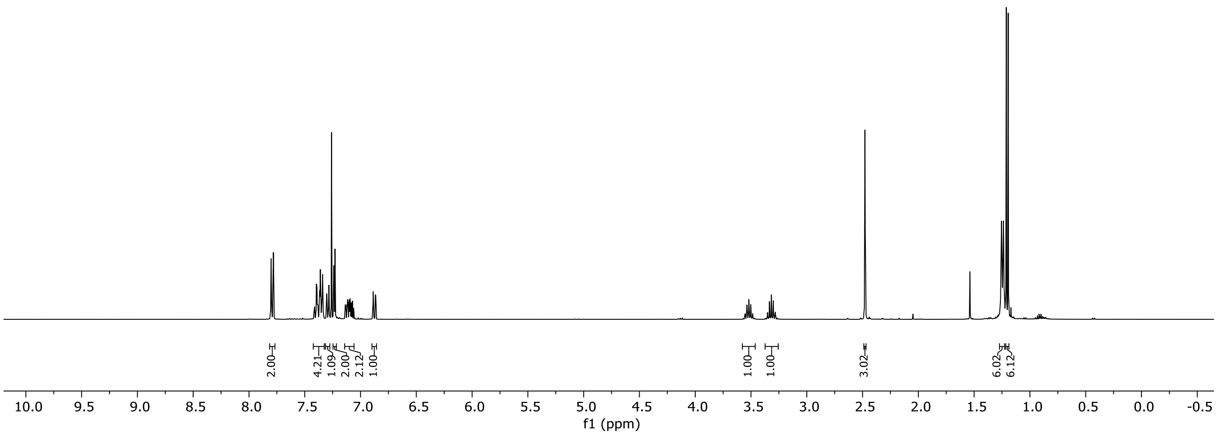

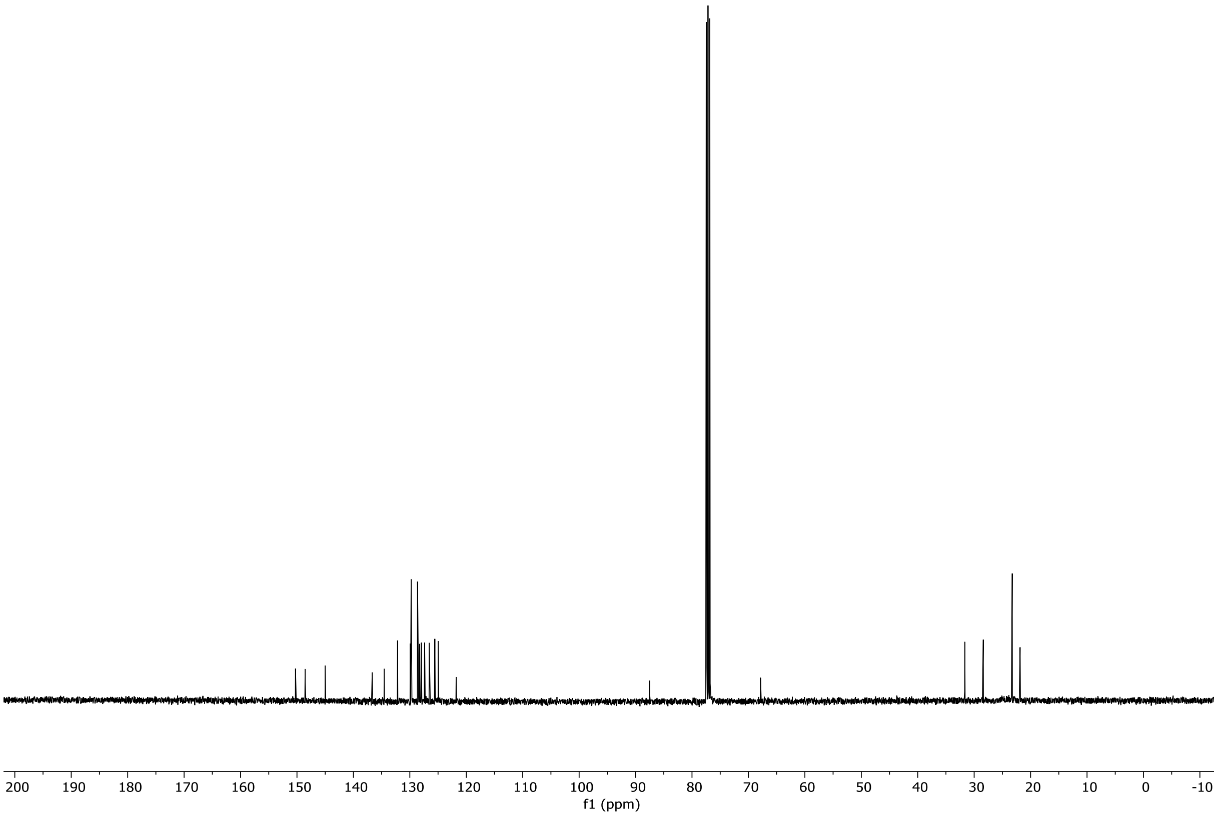

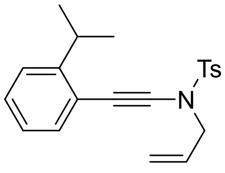
Ts
N

1w

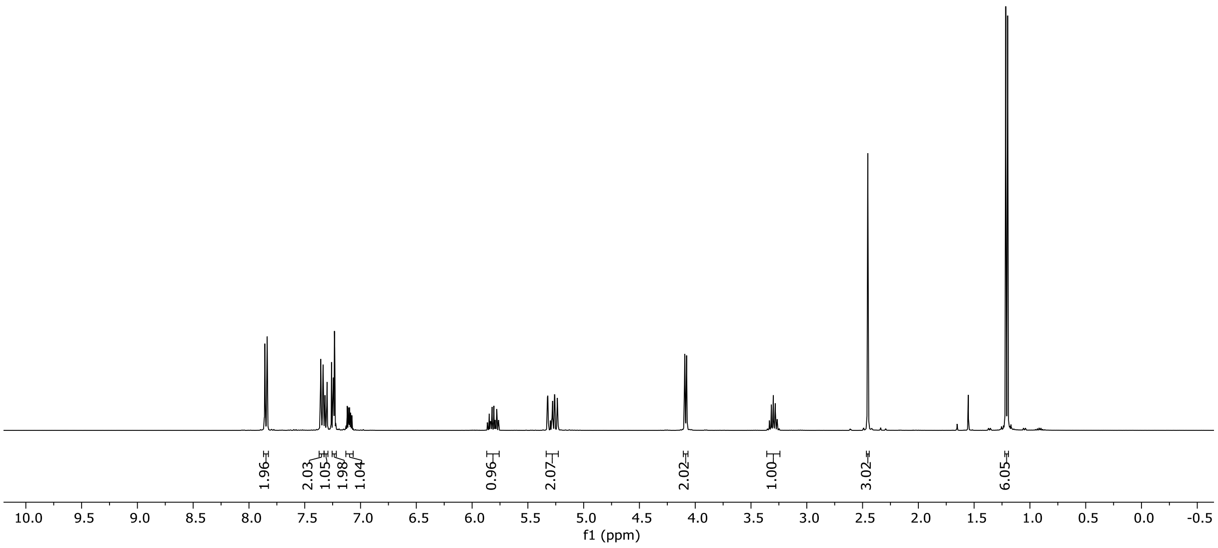
1.96
2.03
1.05
1.98
1.04
0.96
2.07
2.02
1.00
3.02
6.05
10.0 9.5 9.0 8.5 8.0 7.5 7.0 6.5 6.0 5.5 5.0 4.5 4.0 3.5 3.0 2.5 2.0 1.5 1.0 0.5 0.0 -0.5
f1 (ppm)

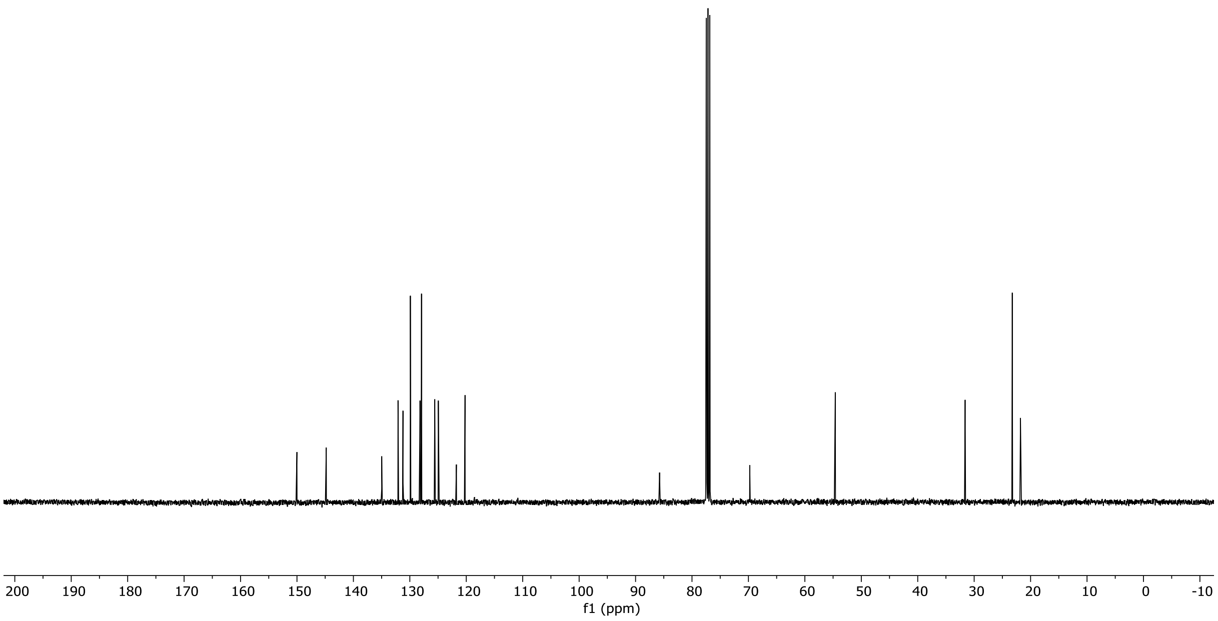
200 190 180 170 160 150 140 130 120 110 100 90 80 70 60 50 40 30 20 10 0 -10
f1 (ppm)

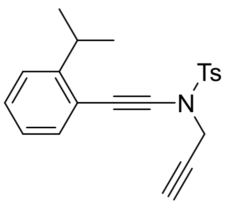
Ts
N

1x

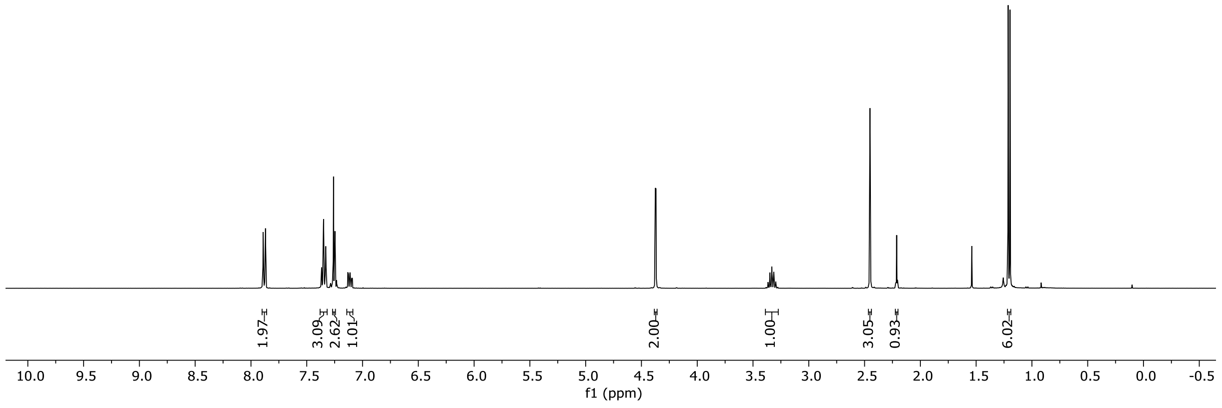
1.97
3.09
2.62
1.01
2.00
1.00
3.05
0.93
6.02
10.0 9.5 9.0 8.5 8.0 7.5 7.0 6.5 6.0 5.5 5.0 4.5 4.0 3.5 3.0 2.5 2.0 1.5 1.0 0.5 0.0 -0.5
f1 (ppm)

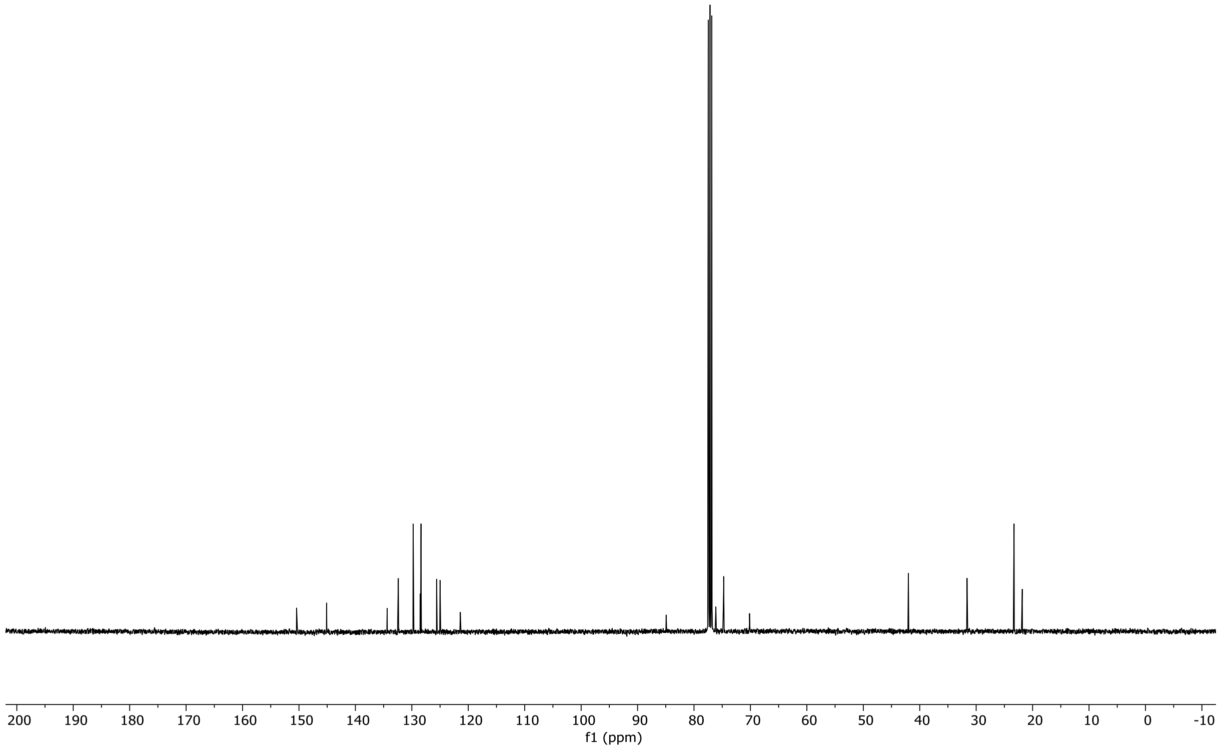
200 190 180 170 160 150 140 130 120 110 100 90 80 70 60 50 40 30 20 10 0 -10
f1 (ppm)

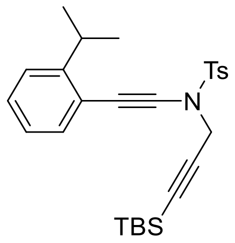
Ts
N
TBS

1y

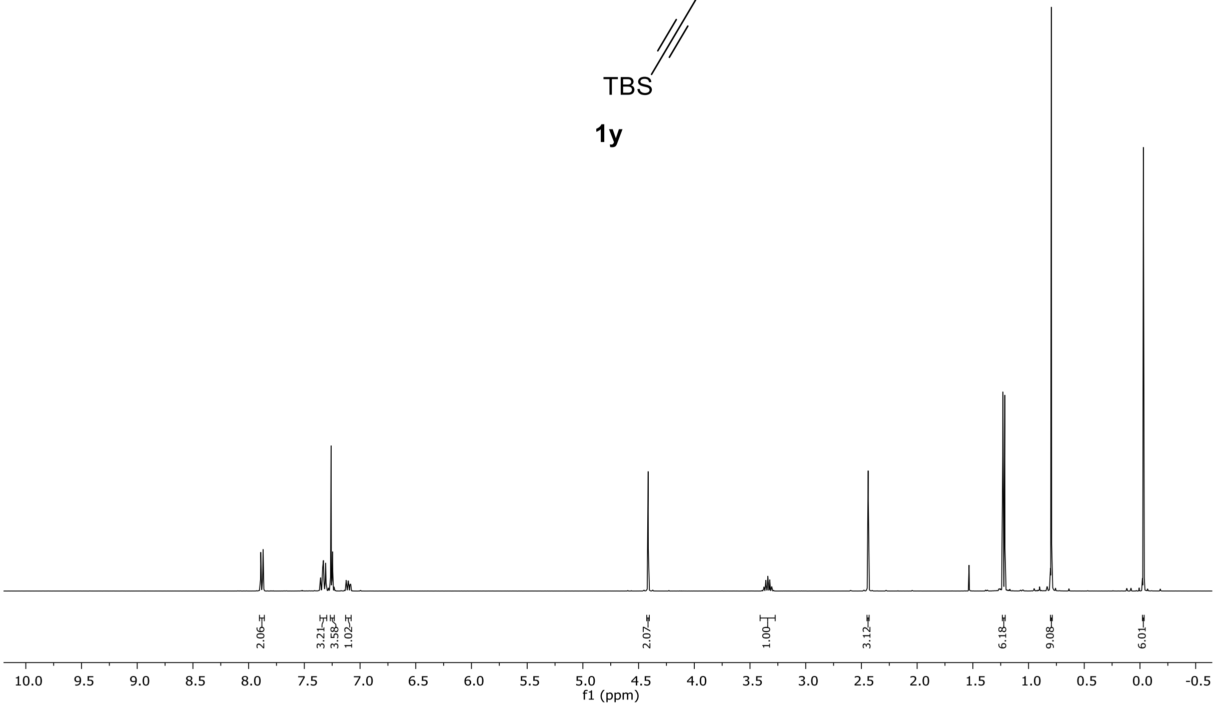
2.06
3.21
3.58
1.02
2.07
1.00
3.12
6.18
9.08
6.01
10.0 9.5 9.0 8.5 8.0 7.5 7.0 6.5 6.0 5.5 5.0 4.5 4.0 3.5 3.0 2.5 2.0 1.5 1.0 0.5 0.0 -0.5
f1 (ppm)

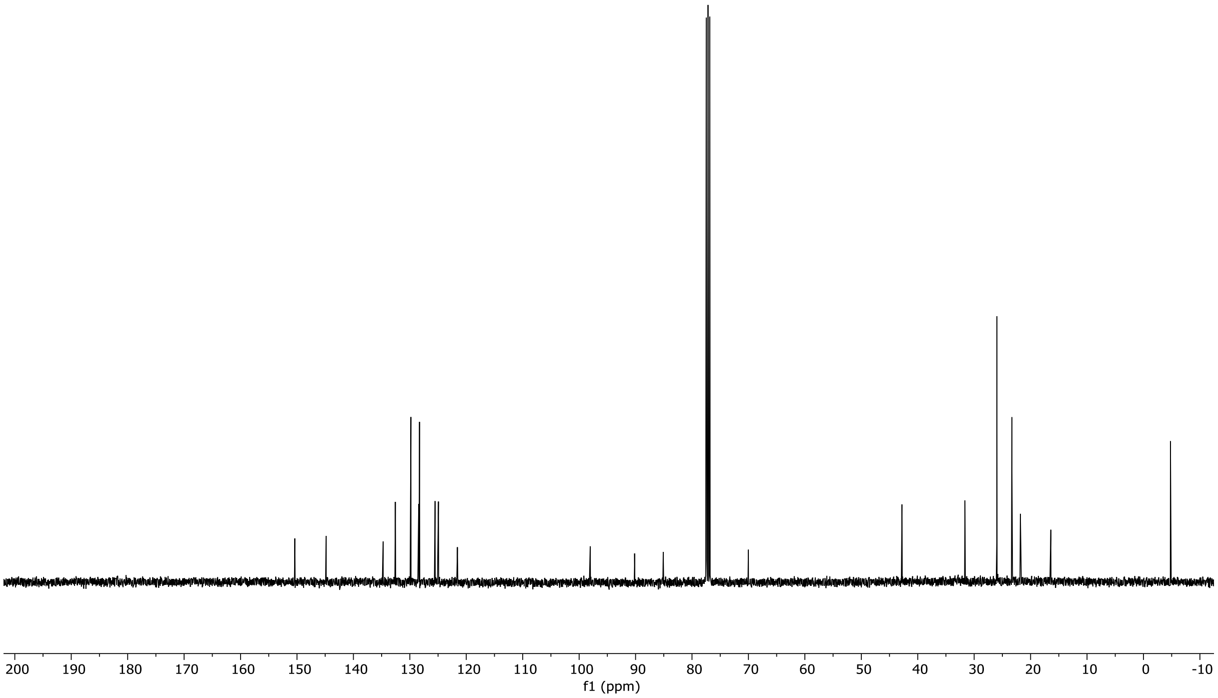
200 190 180 170 160 150 140 130 120 110 100 90 80 70 60 50 40 30 20 10 0 -10
f1 (ppm)

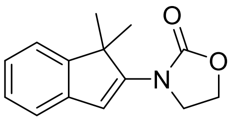
O
N
O

4a

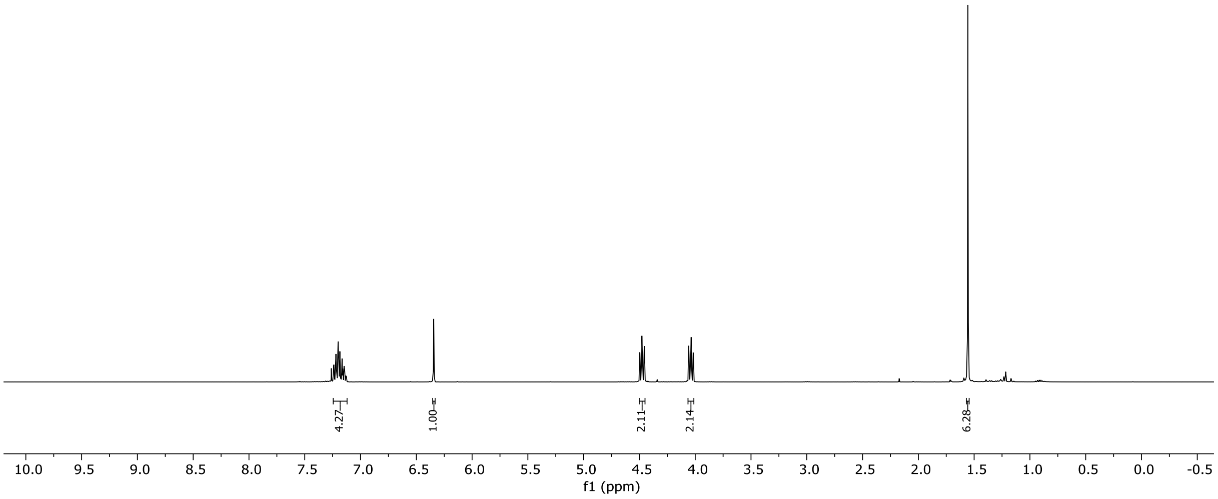
4.27
1.00
2.11
2.14
6.28
10.0 9.5 9.0 8.5 8.0 7.5 7.0 6.5 6.0 5.5 5.0 4.5 4.0 3.5 3.0 2.5 2.0 1.5 1.0 0.5 0.0 -0.5
f1 (ppm)

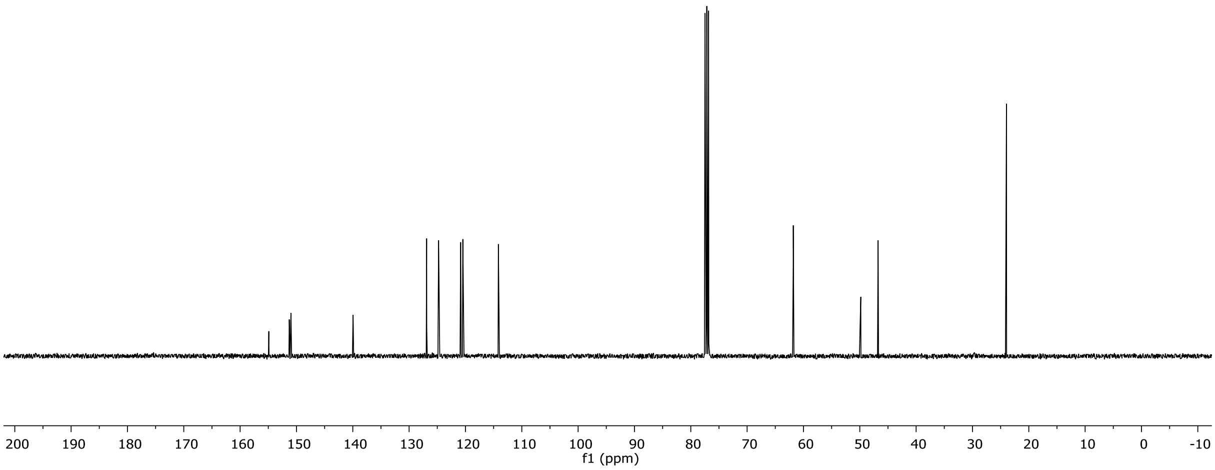
200 190 180 170 160 150 140 130 120 110 100 90 80 70 60 50 40 30 20 10 0 -10
f1 (ppm)

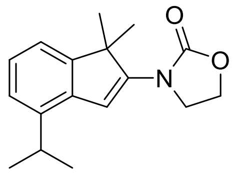
O
N
O

4b

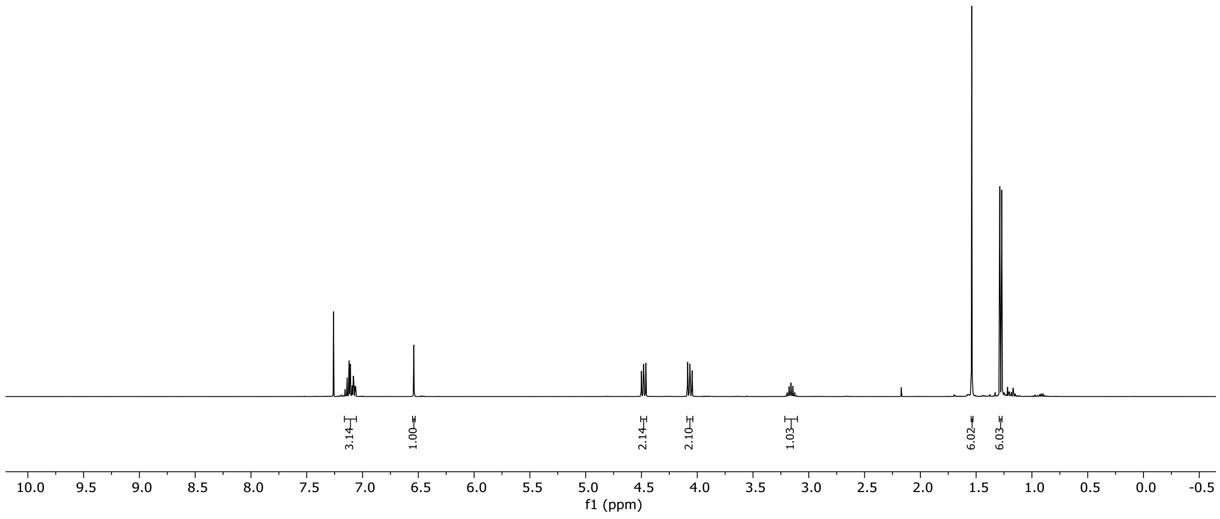
3.14
1.00
2.14
2.10
1.03
6.02
6.03
10.0 9.5 9.0 8.5 8.0 7.5 7.0 6.5 6.0 5.5 5.0 4.5 4.0 3.5 3.0 2.5 2.0 1.5 1.0 0.5 0.0 -0.5
f1 (ppm)

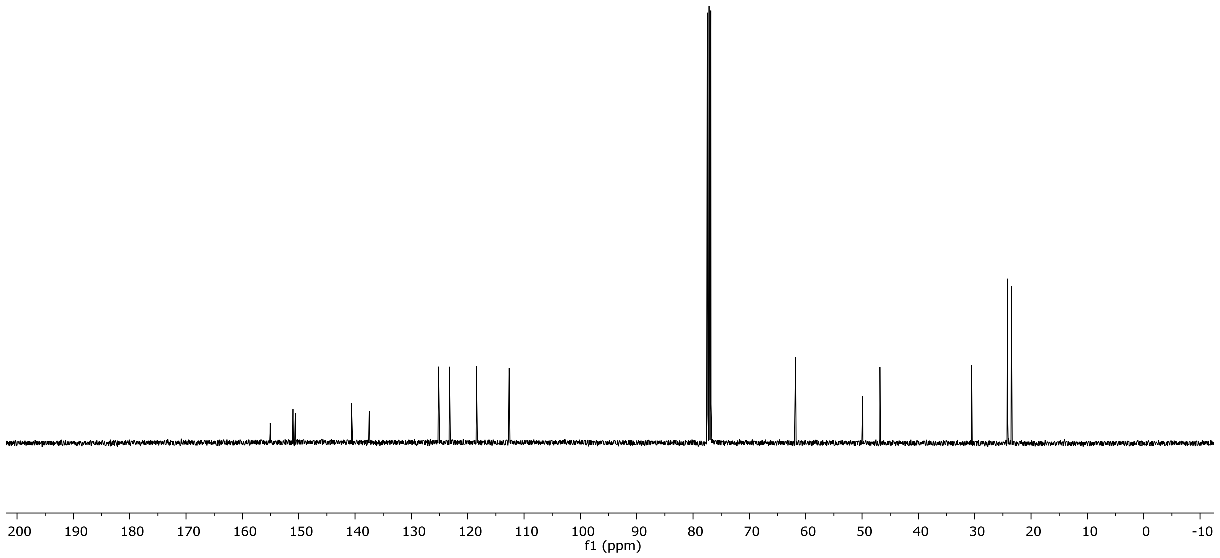
200 190 180 170 160 150 140 130 120 110 100 90 80 70 60 50 40 30 20 10 0 -10
f1 (ppm)

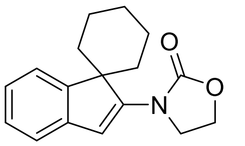
O
N
O

4c

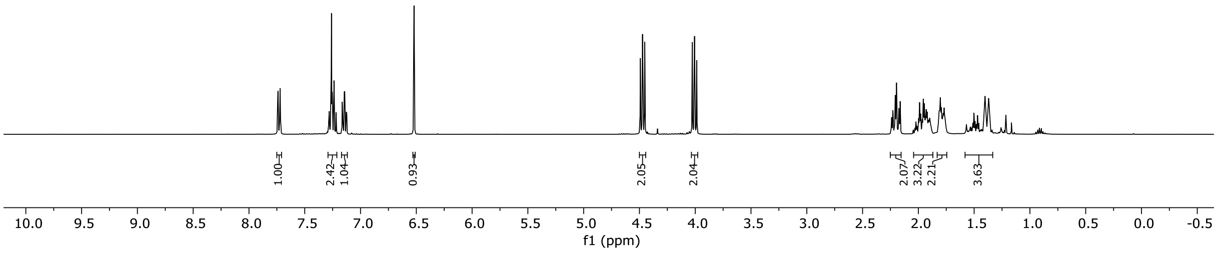
1.00
2.42
1.04
0.93
2.05
2.04
2.07
3.22
2.21
3.63
10.0 9.5 9.0 8.5 8.0 7.5 7.0 6.5 6.0 5.5 5.0 4.5 4.0 3.5 3.0 2.5 2.0 1.5 1.0 0.5 0.0 -0.5
f1 (ppm)

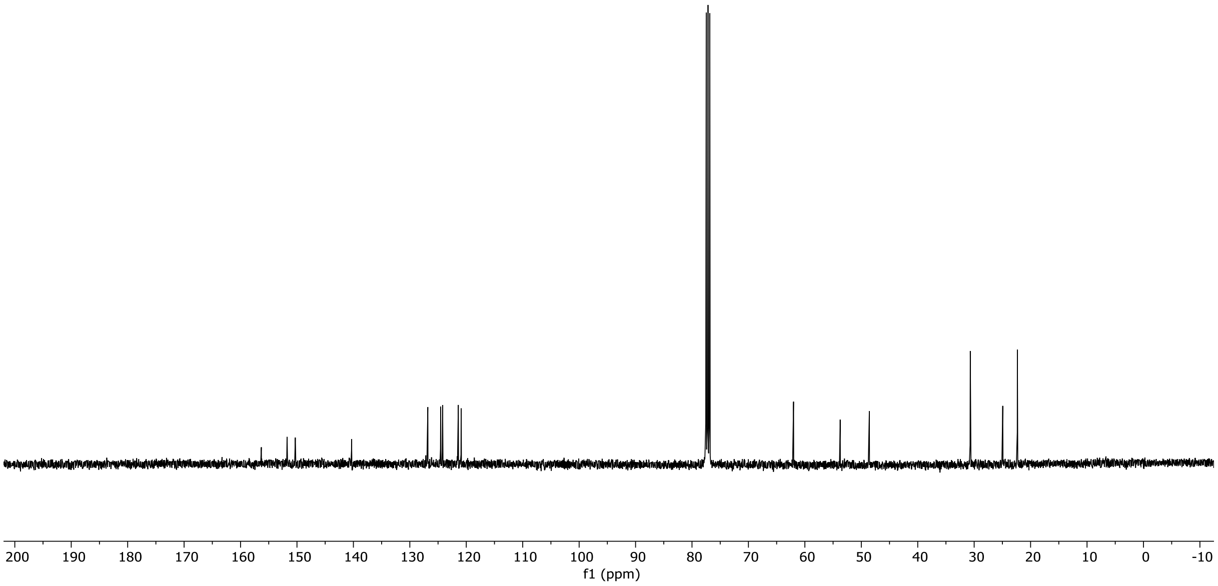
200 190 180 170 160 150 140 130 120 110 100 90 80 70 60 50 40 30 20 10 0 -10
f1 (ppm)

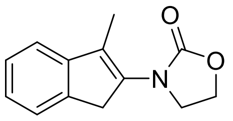
O
N
O

4d

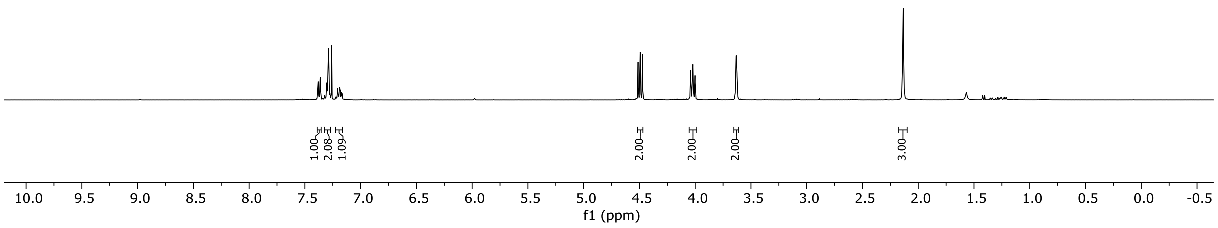
1.00
2.08
1.09
2.00
2.00
2.00
3.00
10.0 9.5 9.0 8.5 8.0 7.5 7.0 6.5 6.0 5.5 5.0 4.5 4.0 3.5 3.0 2.5 2.0 1.5 1.0 0.5 0.0 -0.5
f1 (ppm)

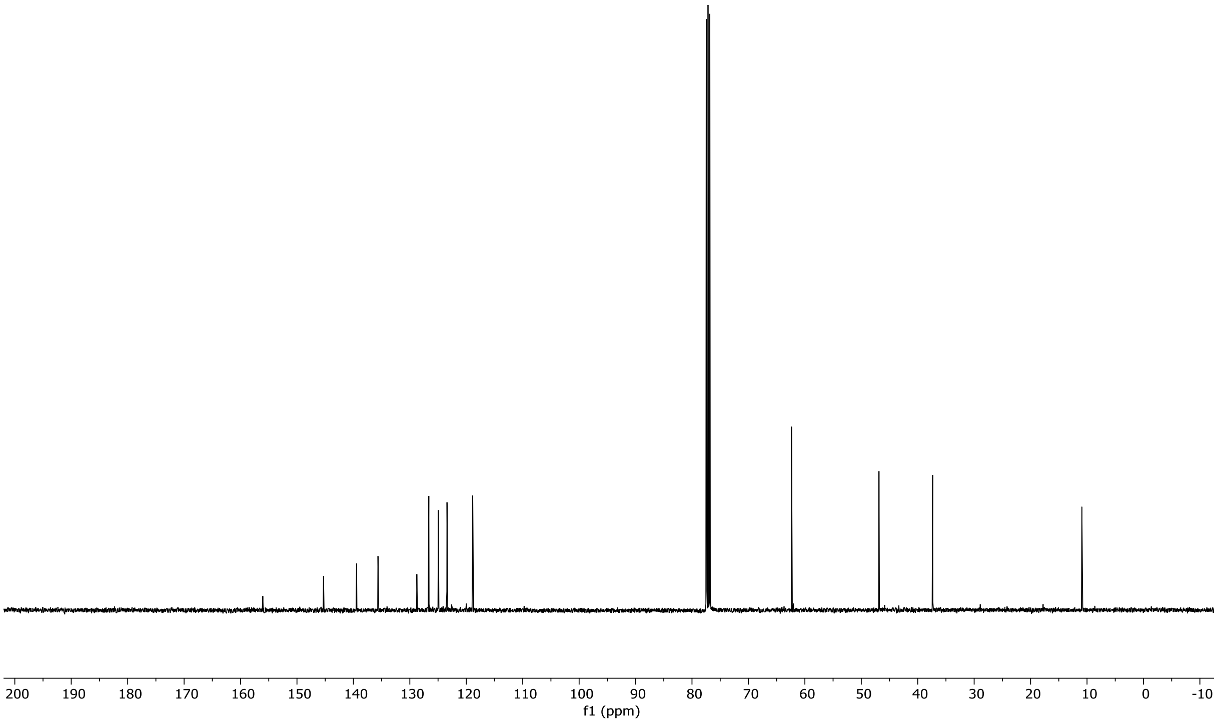
200 190 180 170 160 150 140 130 120 110 100 90 80 70 60 50 40 30 20 10 0 -10
f1 (ppm)

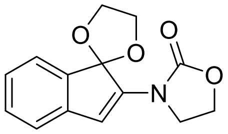
O
O
O
N
O

4f

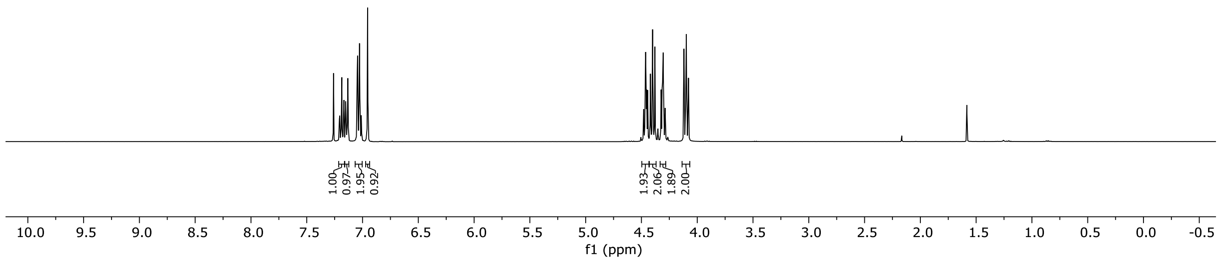
1.00
0.97
1.95
0.92
1.93
2.06
1.89
2.00
10.0 9.5 9.0 8.5 8.0 7.5 7.0 6.5 6.0 5.5 5.0 4.5 4.0 3.5 3.0 2.5 2.0 1.5 1.0 0.5 0.0 -0.5
f1 (ppm)

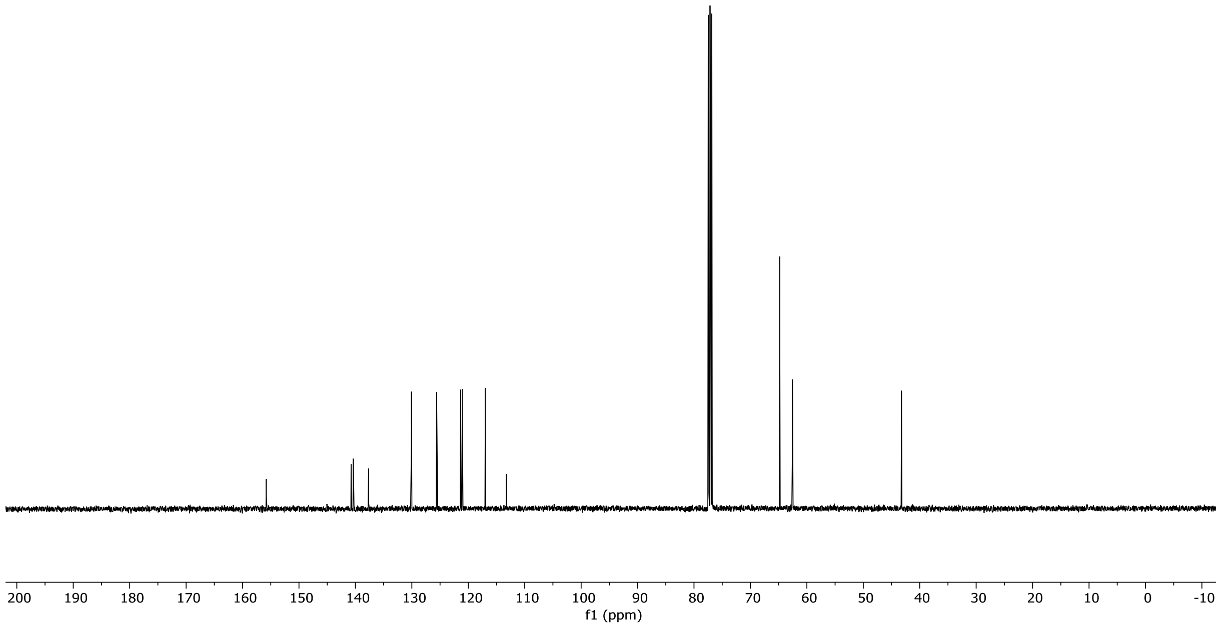
200 190 180 170 160 150 140 130 120 110 100 90 80 70 60 50 40 30 20 10 0 -10
f1 (ppm)

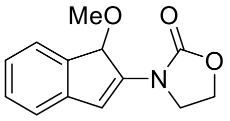
MeO
O
N
O

4g

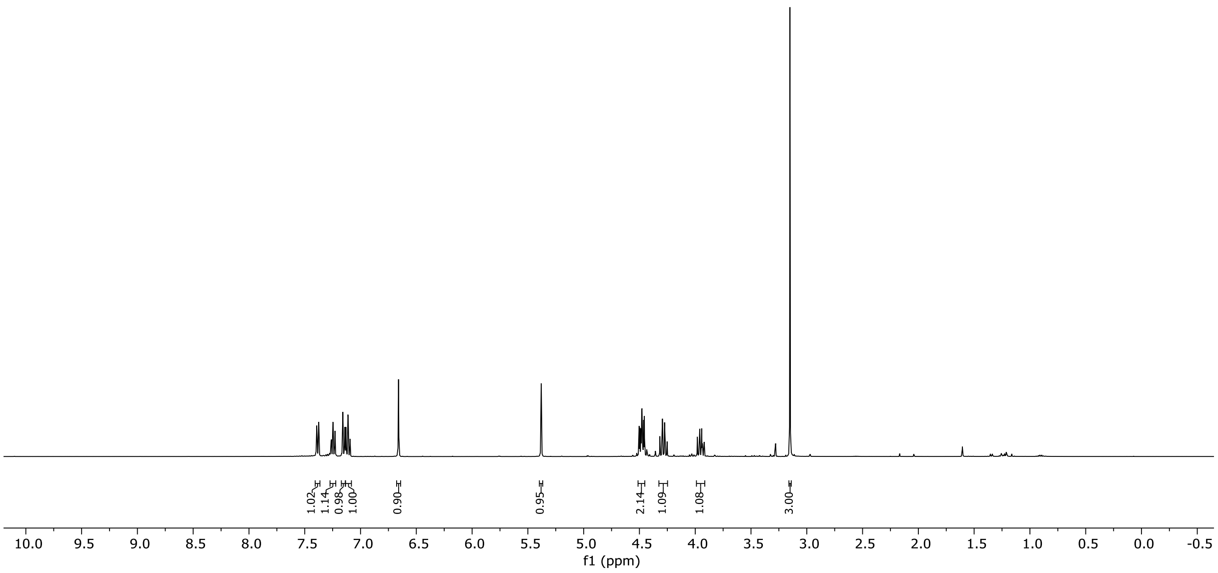
1.02
1.14
0.98
1.00
0.90
0.95
2.14
1.09
1.08
3.00
10.0 9.5 9.0 8.5 8.0 7.5 7.0 6.5 6.0 5.5 5.0 4.5 4.0 3.5 3.0 2.5 2.0 1.5 1.0 0.5 0.0 -0.5
f1 (ppm)

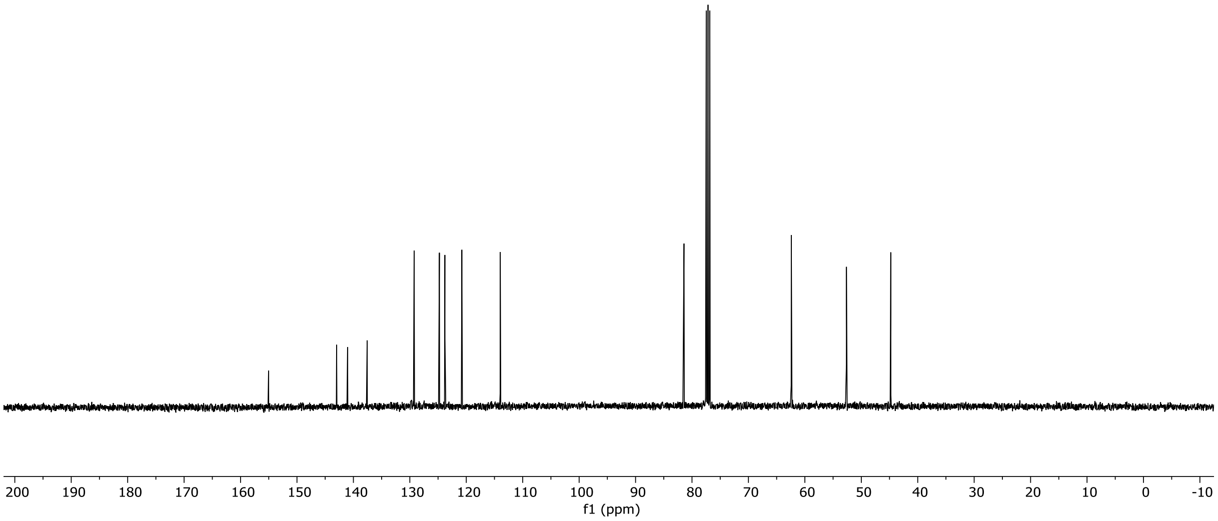
200 190 180 170 160 150 140 130 120 110 100 90 80 70 60 50 40 30 20 10 0 -10
f1 (ppm)

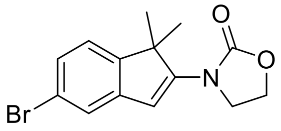

4i

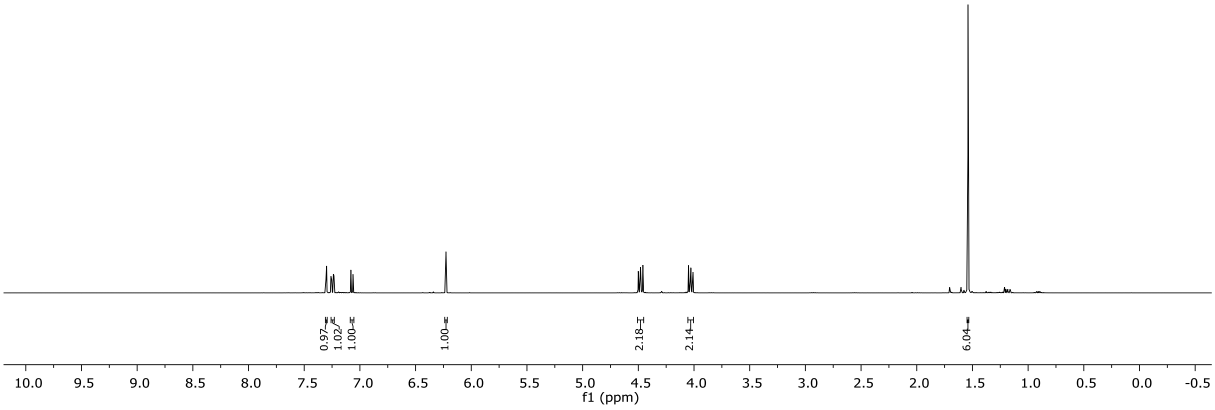

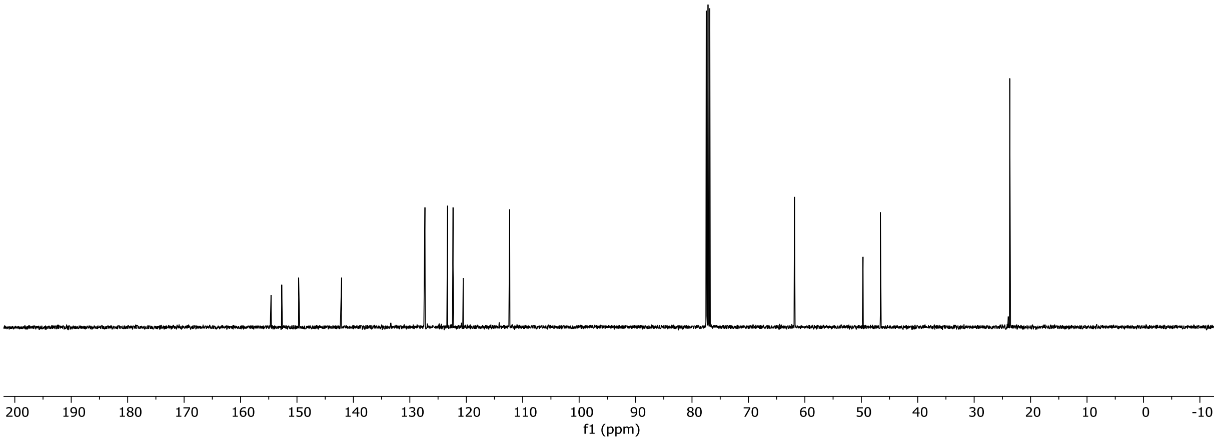

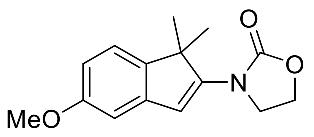
MeO
N
O
O

4j

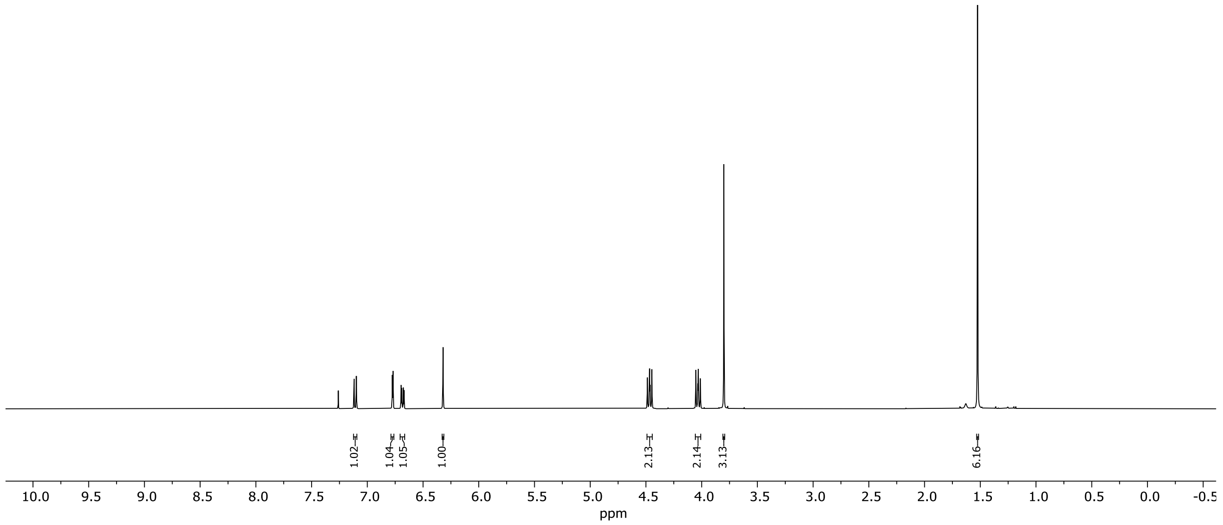
1.02
1.04
1.05
1.00
2.13
2.14
3.13
6.16
10.0 9.5 9.0 8.5 8.0 7.5 7.0 6.5 6.0 5.5 5.0 4.5 4.0 3.5 3.0 2.5 2.0 1.5 1.0 0.5 0.0 -0.5
ppm

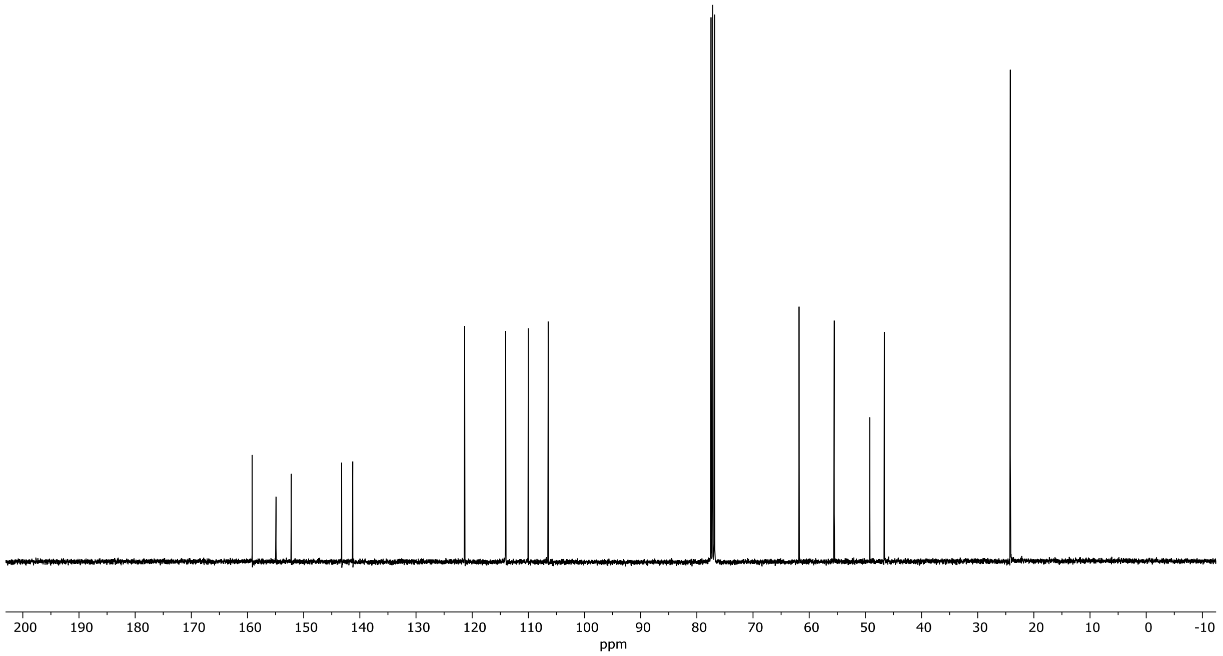
200 190 180 170 160 150 140 130 120 110 100 90 80 70 60 50 40 30 20 10 0 -10
ppm

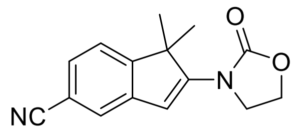

4k

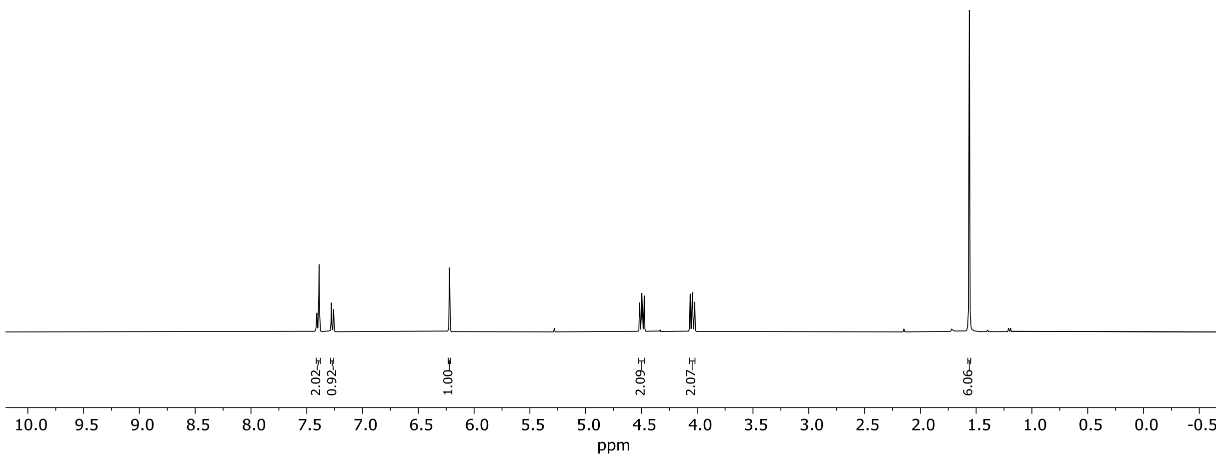

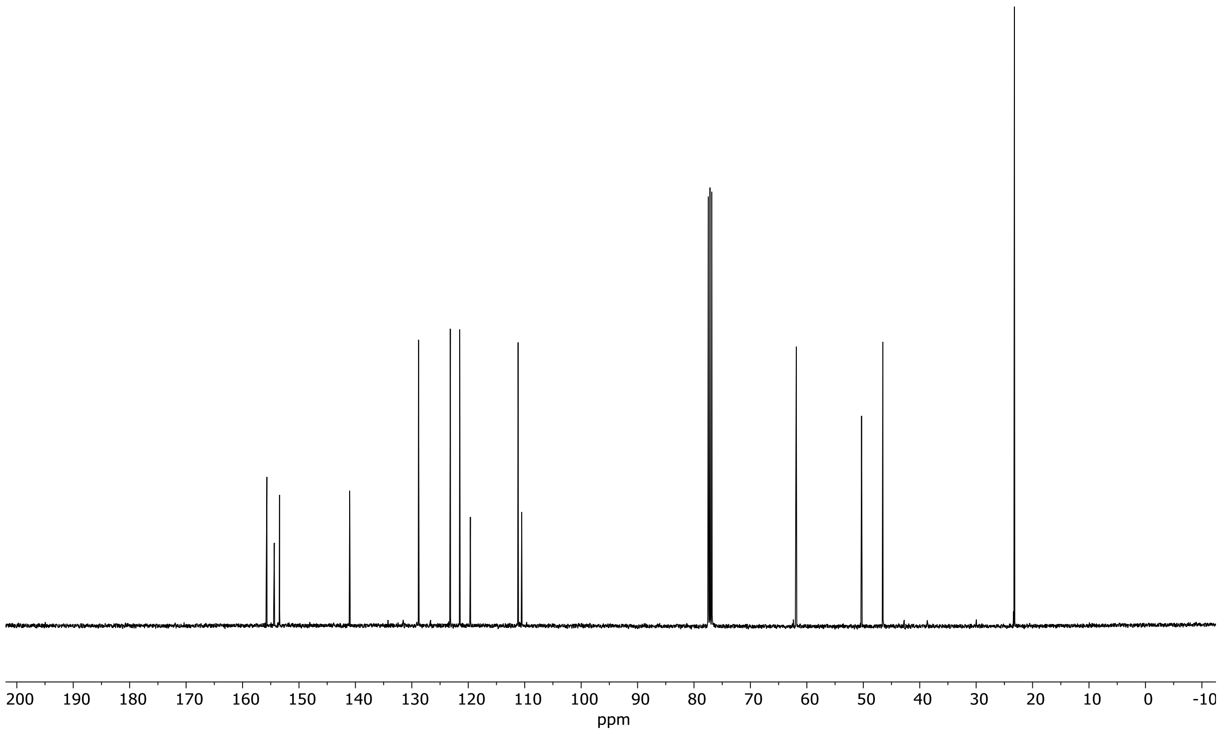

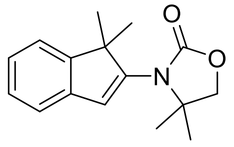

4l

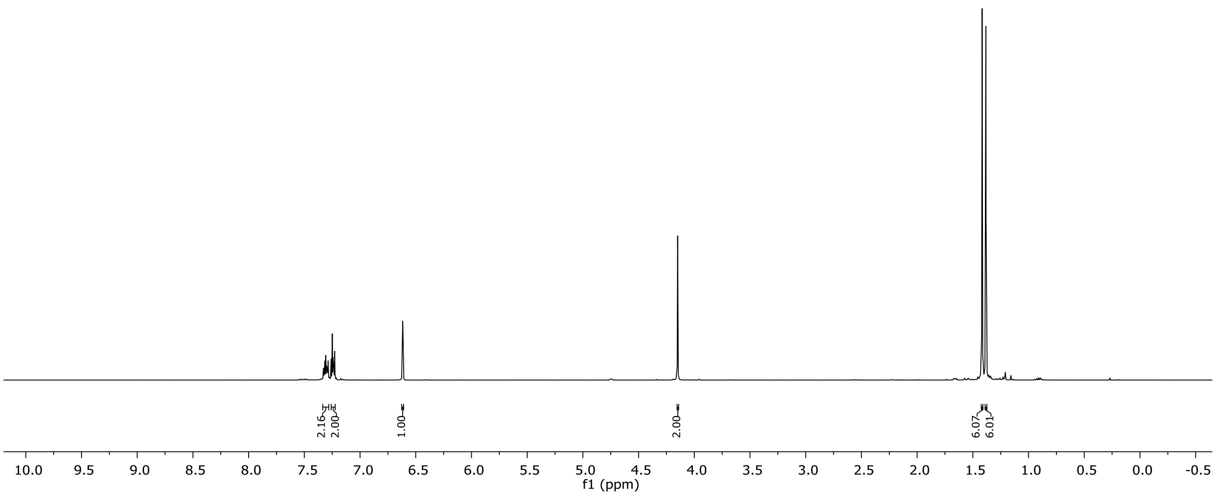
2.16
2.00
1.00
2.00
6.07
6.01
10.0 9.5 9.0 8.5 8.0 7.5 7.0 6.5 6.0 5.5 5.0 4.5 4.0 3.5 3.0 2.5 2.0 1.5 1.0 0.5 0.0 -0.5
f1 (ppm)

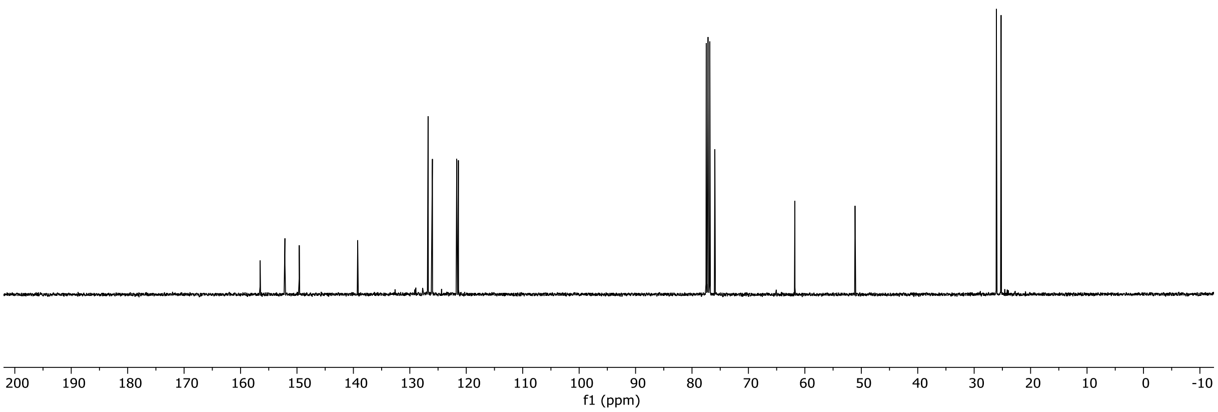
200 190 180 170 160 150 140 130 120 110 100 90 80 70 60 50 40 30 20 10 0 -10
f1 (ppm)

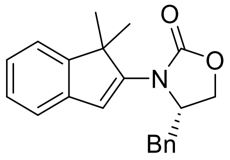
O
N
O
Bn

4m

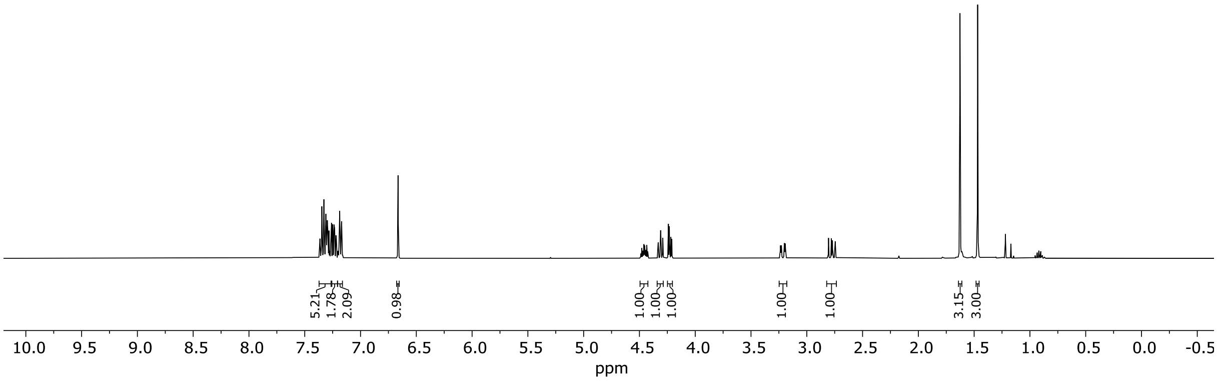
5.21
1.78
2.09
0.98
1.00
1.00
1.00
1.00
1.00
3.15
3.00
10.0 9.5 9.0 8.5 8.0 7.5 7.0 6.5 6.0 5.5 5.0 4.5 4.0 3.5 3.0 2.5 2.0 1.5 1.0 0.5 0.0 -0.5
ppm

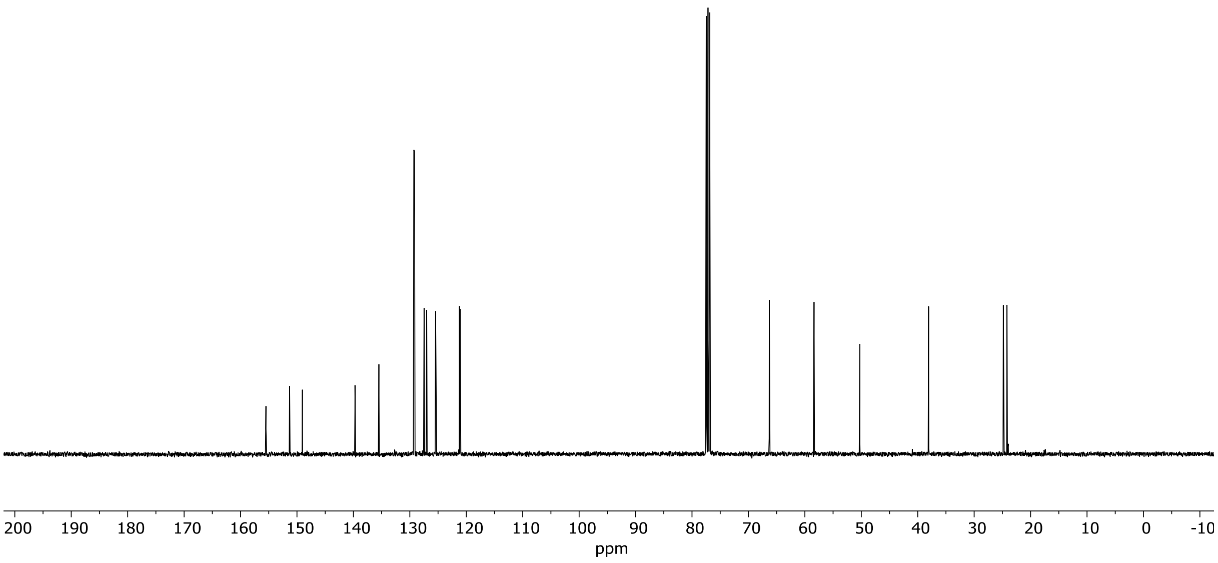
200 190 180 170 160 150 140 130 120 110 100 90 80 70 60 50 40 30 20 10 0 -10
ppm

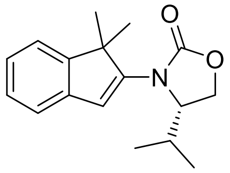
O
N
O

4n

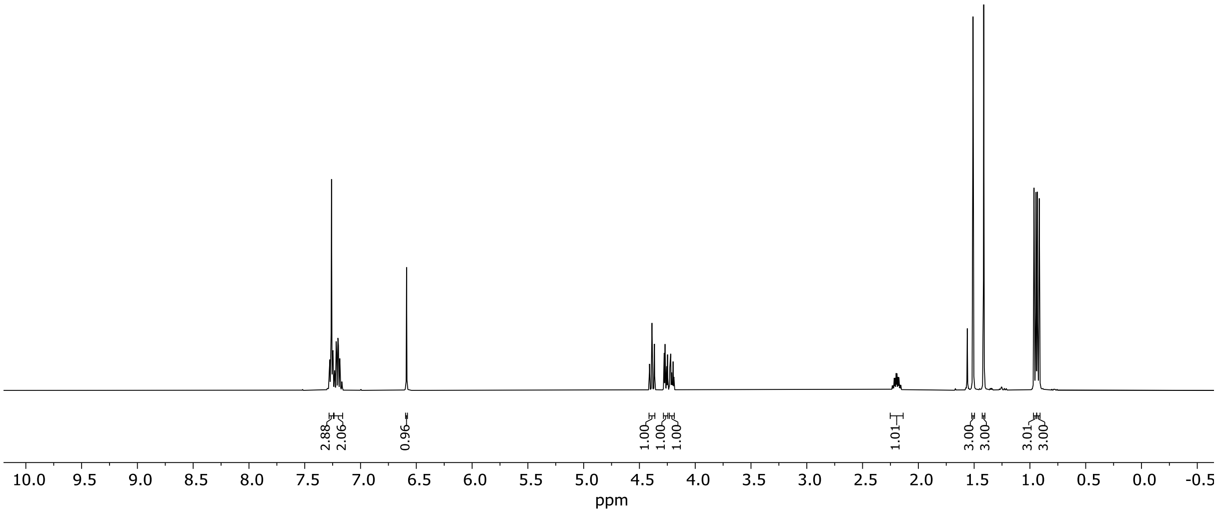
2.88
2.06
0.96
1.00
1.00
1.00
1.01
3.00
3.00
3.01
3.00
10.0 9.5 9.0 8.5 8.0 7.5 7.0 6.5 6.0 5.5 5.0 4.5 4.0 3.5 3.0 2.5 2.0 1.5 1.0 0.5 0.0 -0.5
ppm

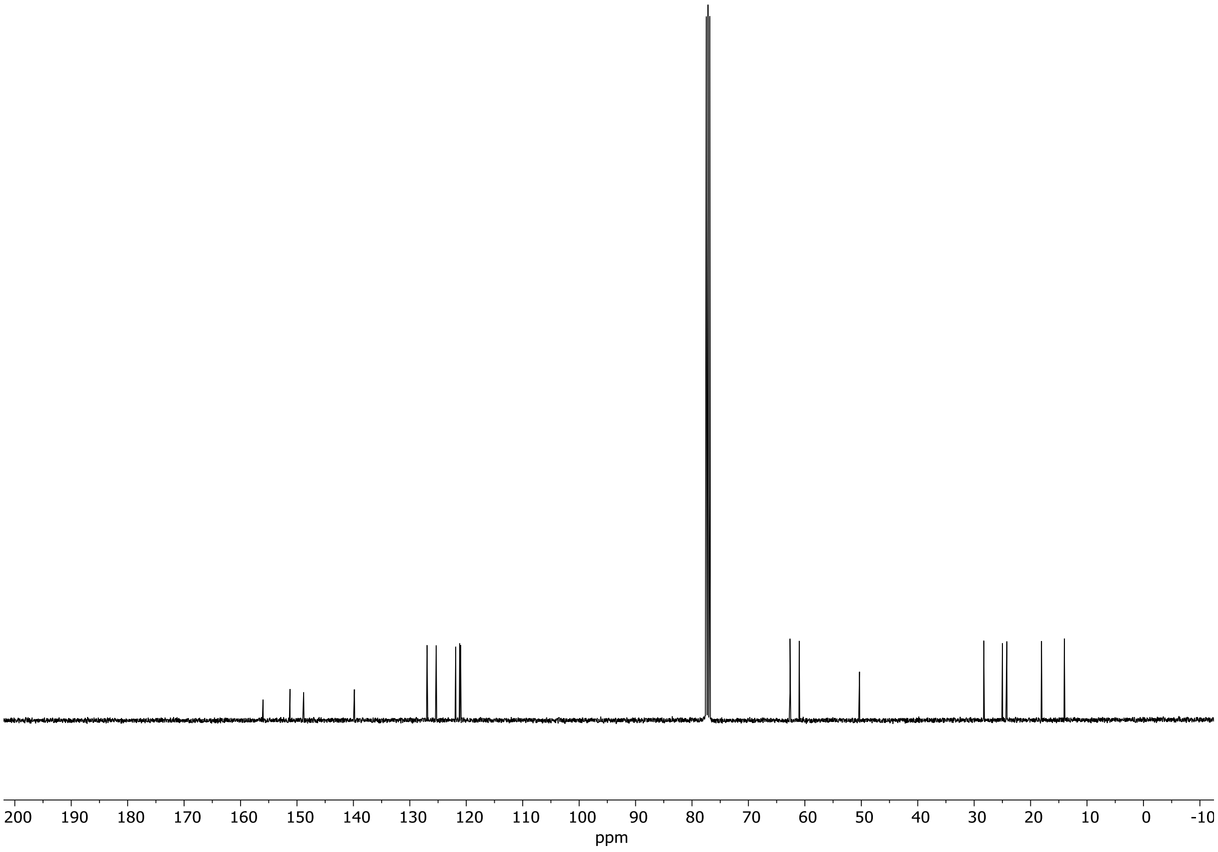
200 190 180 170 160 150 140 130 120 110 100 90 80 70 60 50 40 30 20 10 0 -10
ppm

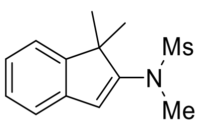
Ms
N
Me

4o

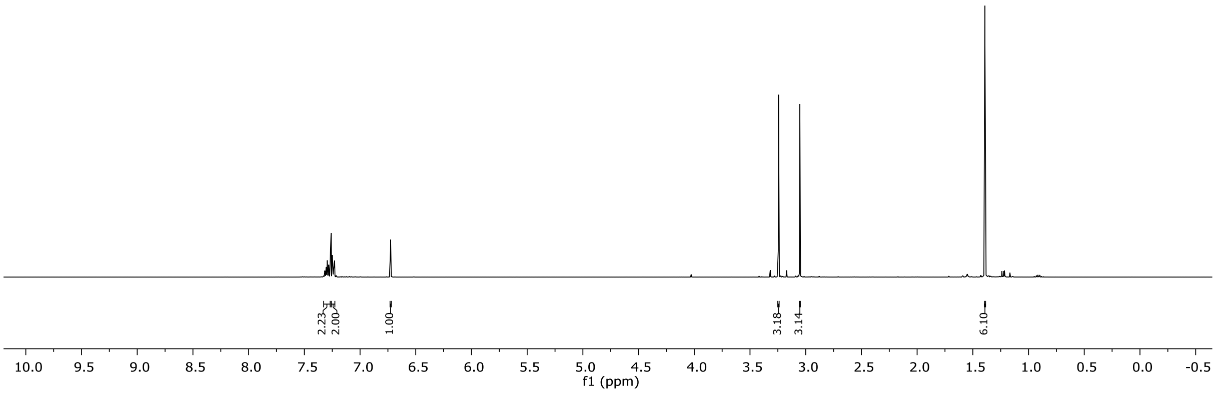
2.23
2.00
1.00
3.18
3.14
6.10
10.0
9.5
9.0
8.5
8.0
7.5
7.0
6.5
6.0
5.5
5.0
4.5
4.0
3.5
3.0
2.5
2.0
1.5
1.0
0.5
0.0
-0.5
f1 (ppm)

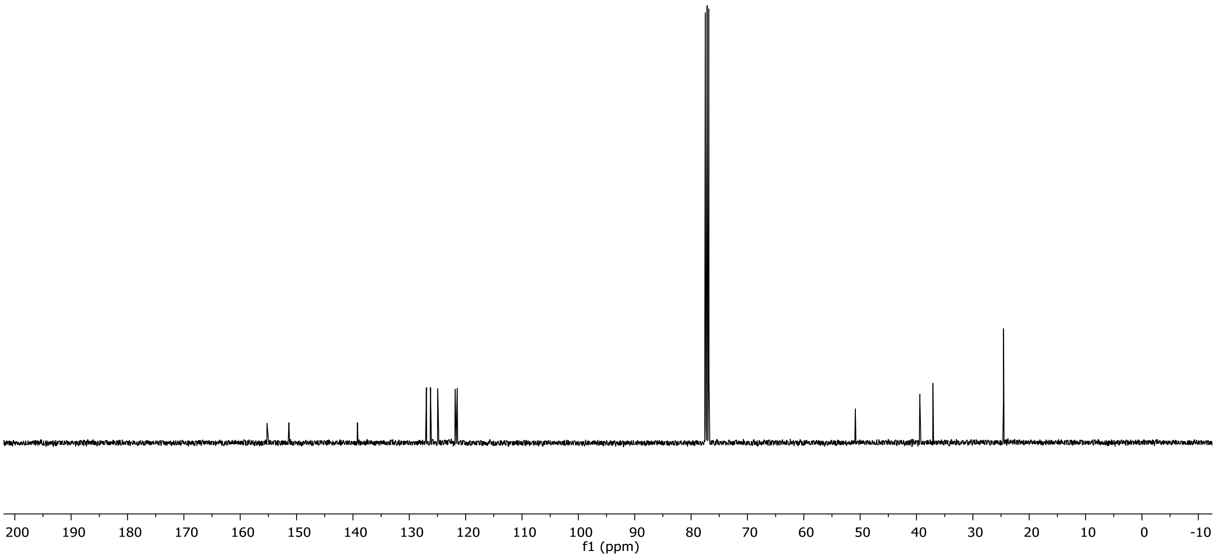
200
190
180
170
160
150
140
130
120
110
100
90
80
70
60
50
40
30
20
10
0
-10
f1 (ppm)

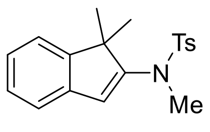
Ts
N
Me

4p

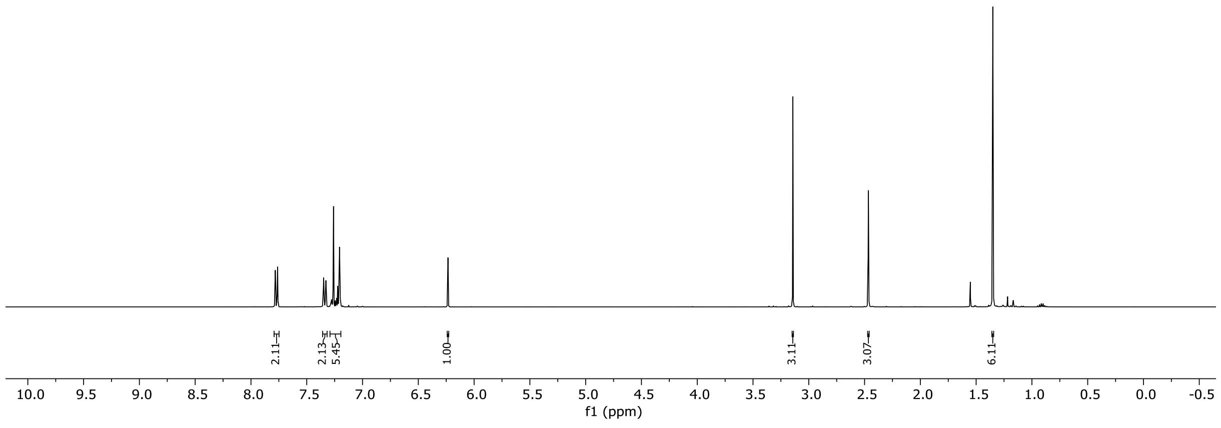
2.11
2.13
5.45
1.00
3.11
3.07
6.11
10.0 9.5 9.0 8.5 8.0 7.5 7.0 6.5 6.0 5.5 5.0 4.5 4.0 3.5 3.0 2.5 2.0 1.5 1.0 0.5 0.0 -0.5
f1 (ppm)

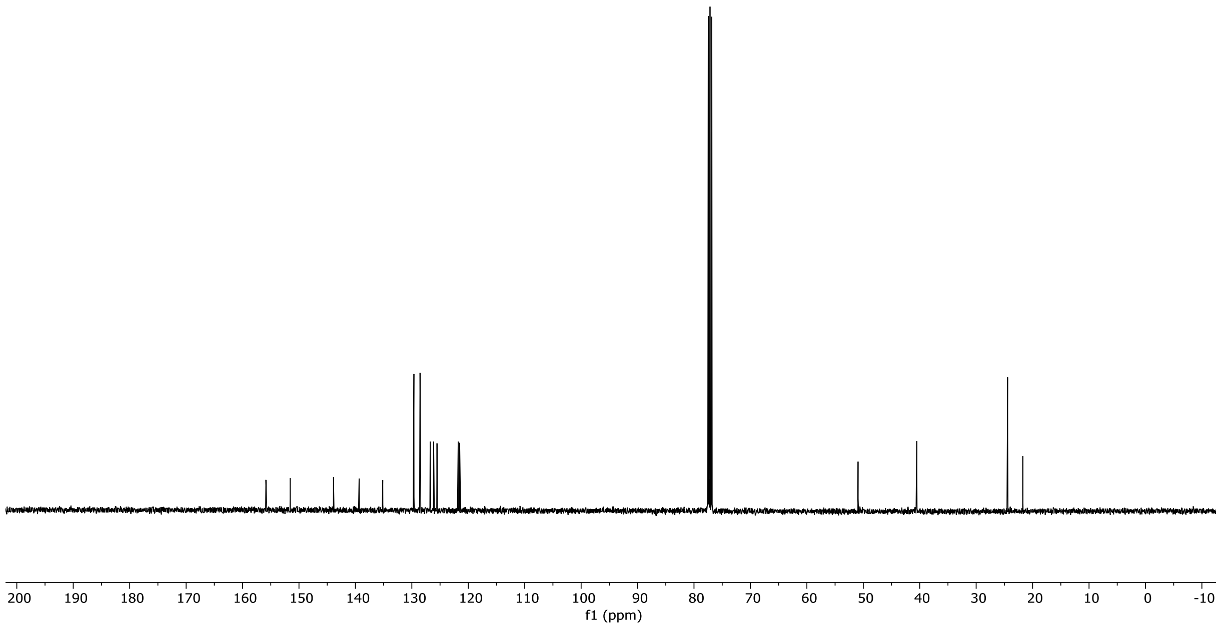
200 190 180 170 160 150 140 130 120 110 100 90 80 70 60 50 40 30 20 10 0 -10
f1 (ppm)

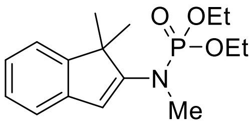
O
OEt
P
OEt
N
Me

4q

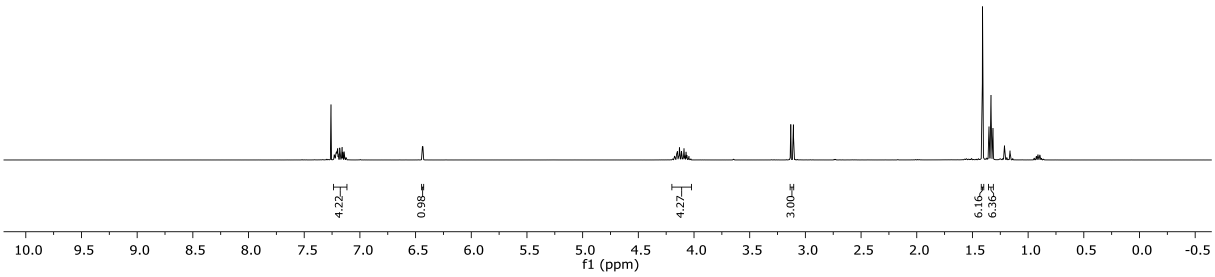
4.22
0.98
4.27
3.00
6.16
6.36
10.0 9.5 9.0 8.5 8.0 7.5 7.0 6.5 6.0 5.5 5.0 4.5 4.0 3.5 3.0 2.5 2.0 1.5 1.0 0.5 0.0 -0.5
f1 (ppm)

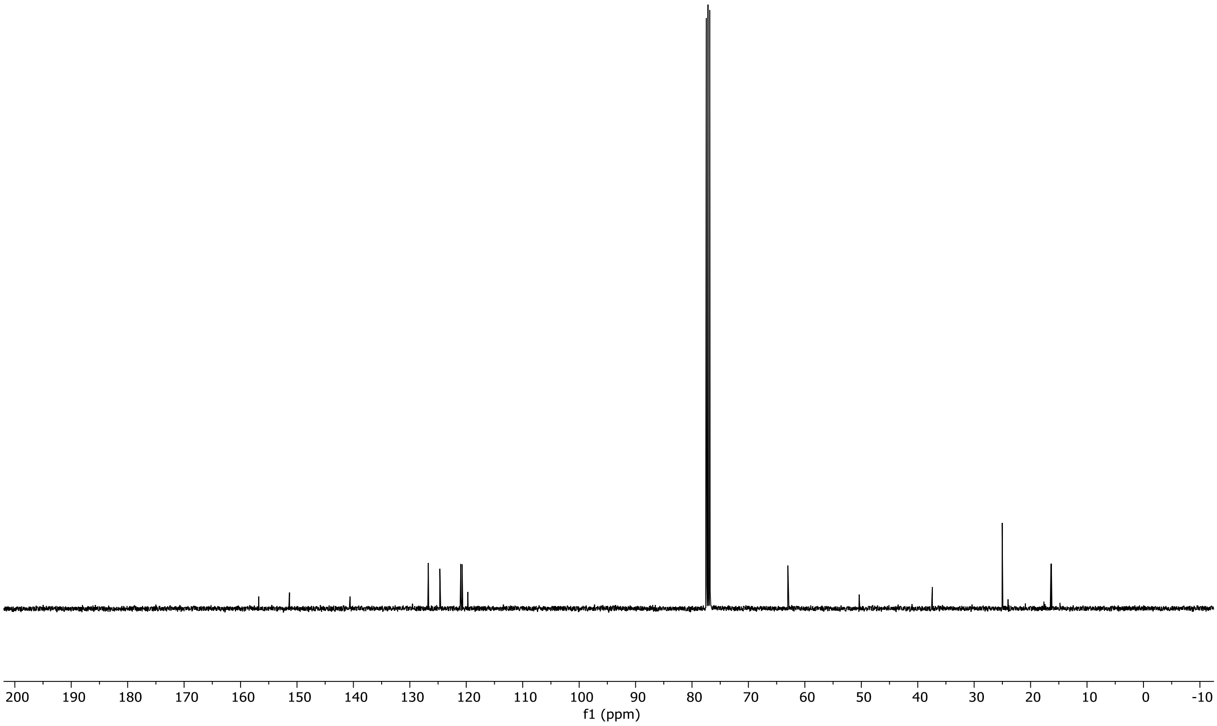
200 190 180 170 160 150 140 130 120 110 100 90 80 70 60 50 40 30 20 10 0 -10
f1 (ppm)

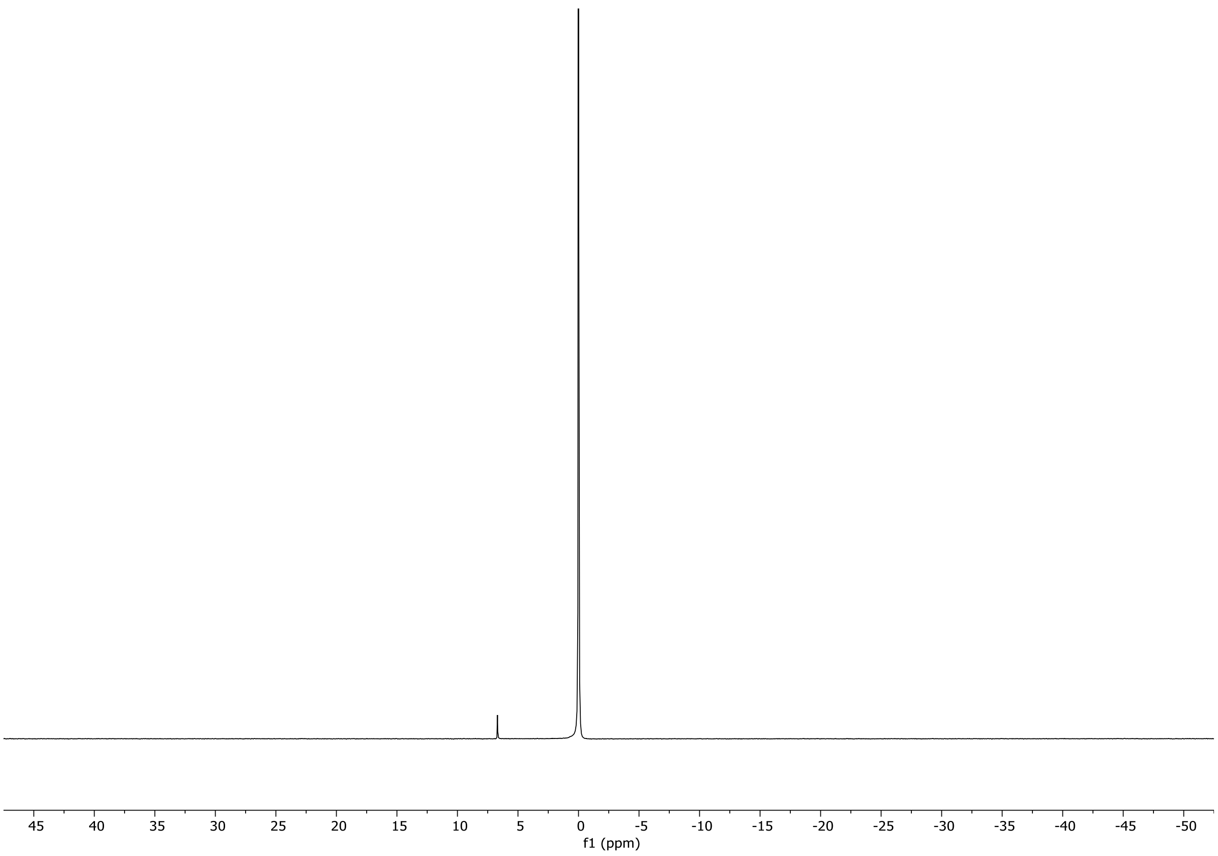
45
40
35
30
25
20
15
10
5
0
-5
-10
-15
-20
-25
-30
-35
-40
-45
-50
f1 (ppm)

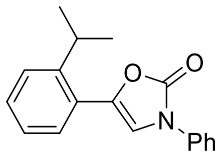
O
O
N
Ph

4'r

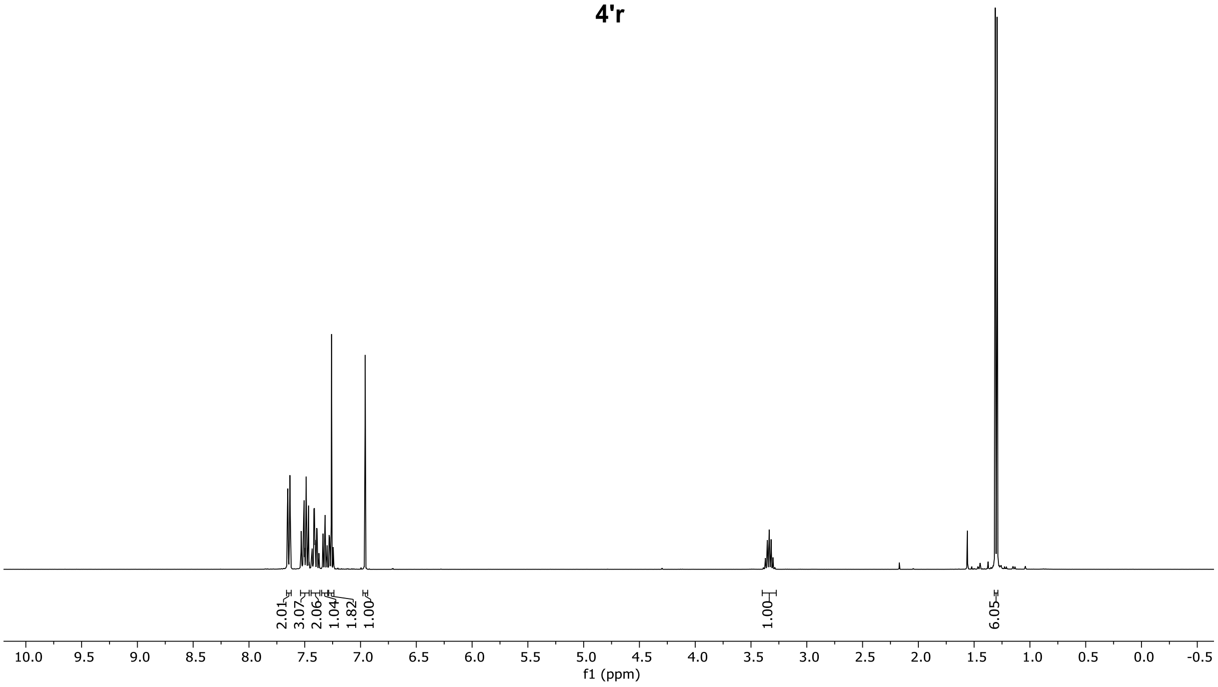
2.01
3.07
2.06
1.04
1.82
1.00
1.00
6.05
10.0 9.5 9.0 8.5 8.0 7.5 7.0 6.5 6.0 5.5 5.0 4.5 4.0 3.5 3.0 2.5 2.0 1.5 1.0 0.5 0.0 -0.5
f1 (ppm)

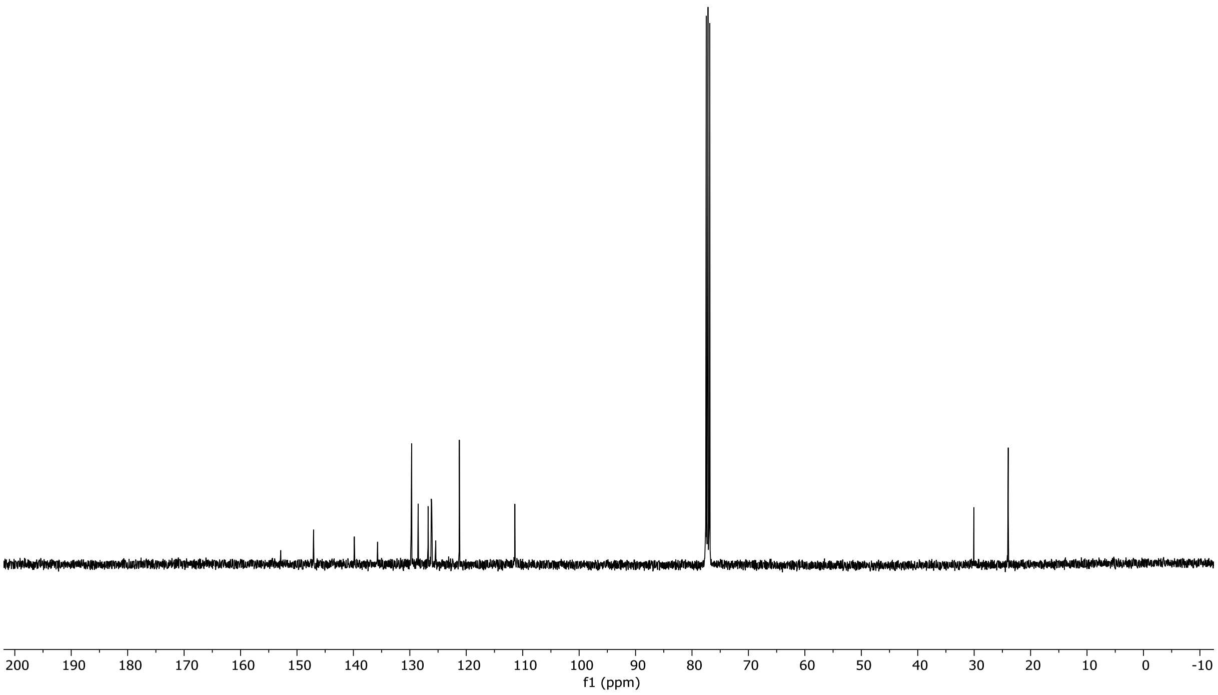
200 190 180 170 160 150 140 130 120 110 100 90 80 70 60 50 40 30 20 10 0 -10
f1 (ppm)

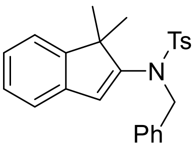
Ts
N
Ph

4t

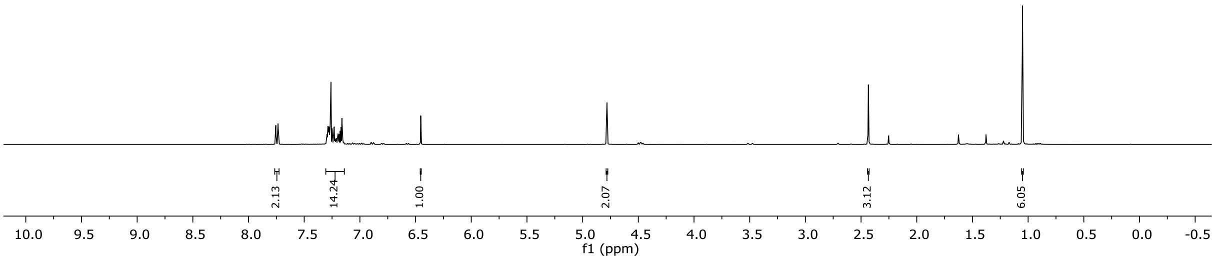
2.13
14.24
1.00
2.07
3.12
6.05
10.0 9.5 9.0 8.5 8.0 7.5 7.0 6.5 6.0 5.5 5.0 4.5 4.0 3.5 3.0 2.5 2.0 1.5 1.0 0.5 0.0 -0.5
f1 (ppm)

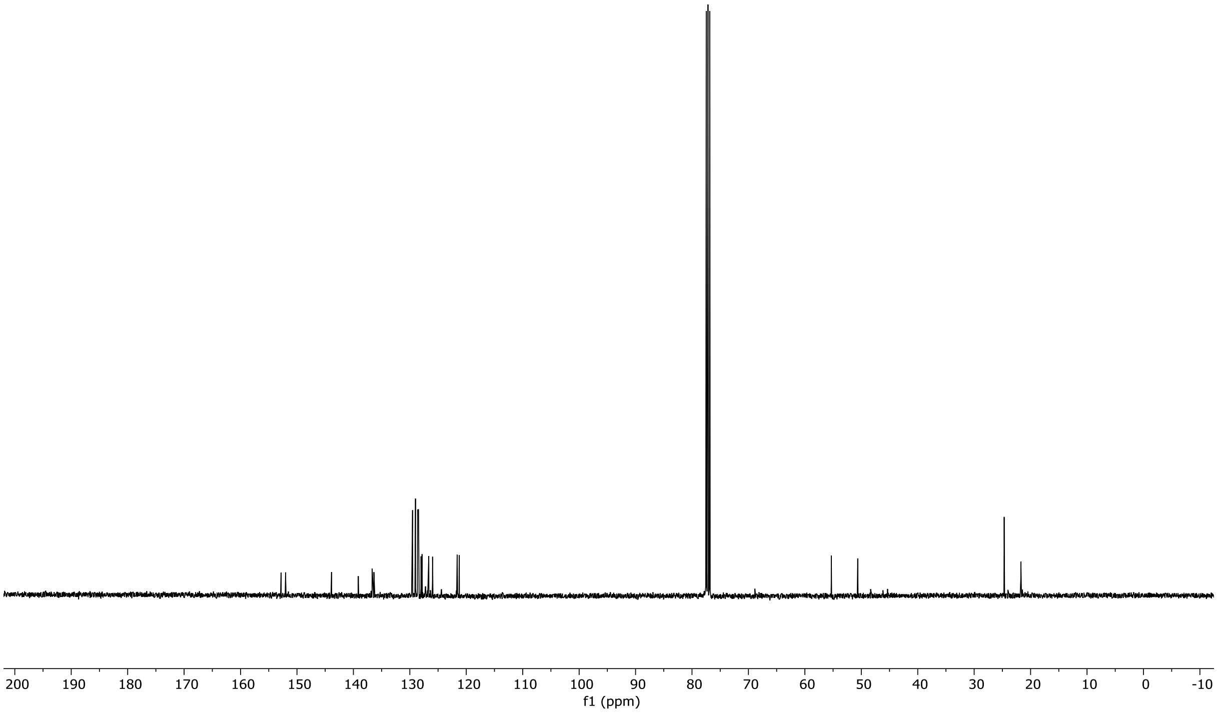
200 190 180 170 160 150 140 130 120 110 100 90 80 70 60 50 40 30 20 10 0 -10
f1 (ppm)

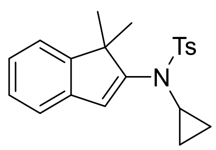
Ts
N

4u

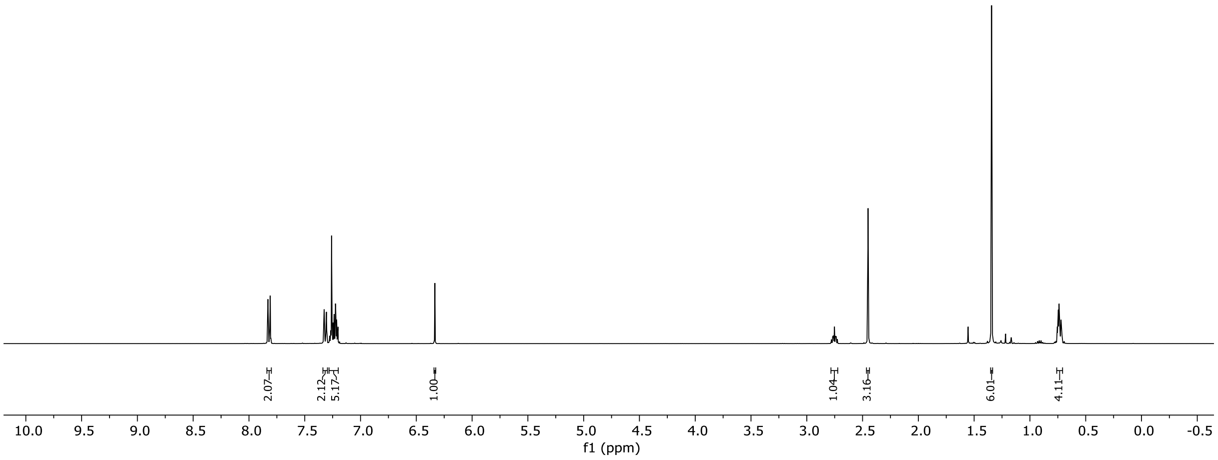
2.07
2.12
5.17
1.00
1.04
3.16
6.01
4.11
10.0
9.5
9.0
8.5
8.0
7.5
7.0
6.5
6.0
5.5
5.0
4.5
4.0
3.5
3.0
2.5
2.0
1.5
1.0
0.5
0.0
-0.5
f1 (ppm)

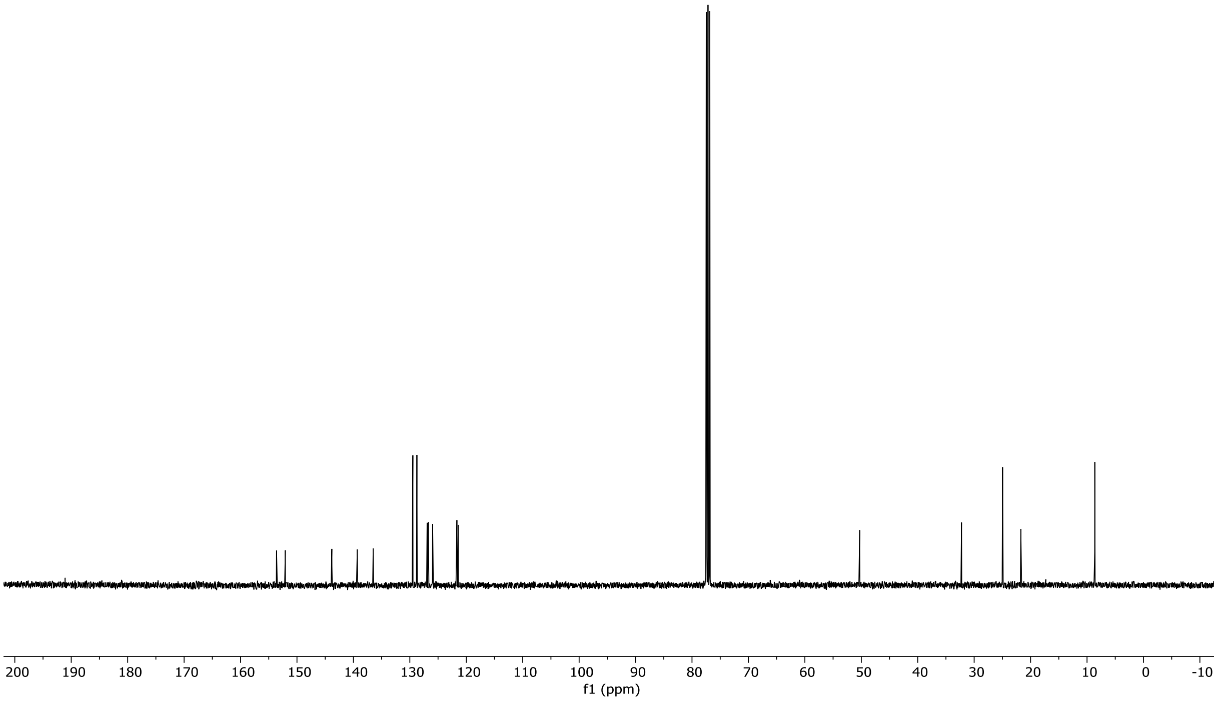
200
190
180
170
160
150
140
130
120
110
100
90
80
70
60
50
40
30
20
10
0
-10
f1 (ppm)

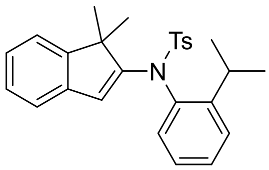
Ts
N

4v

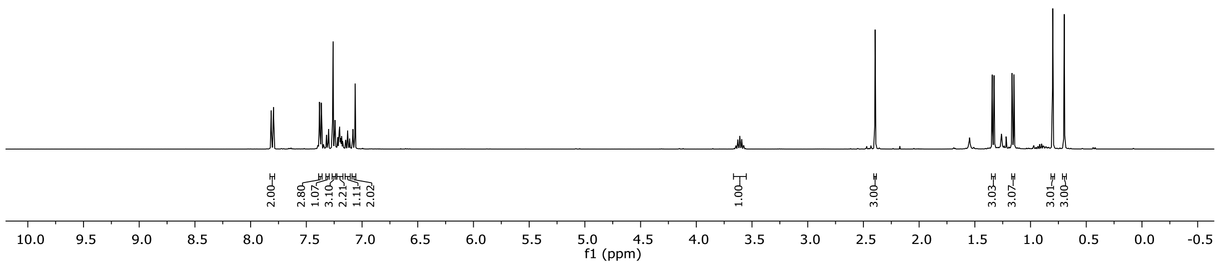
2.00
2.80
1.07
3.10
2.21
1.11
2.02
1.00
3.00
3.03
3.07
3.01
3.00
10.0 9.5 9.0 8.5 8.0 7.5 7.0 6.5 6.0 5.5 5.0 4.5 4.0 3.5 3.0 2.5 2.0 1.5 1.0 0.5 0.0 -0.5
f1 (ppm)

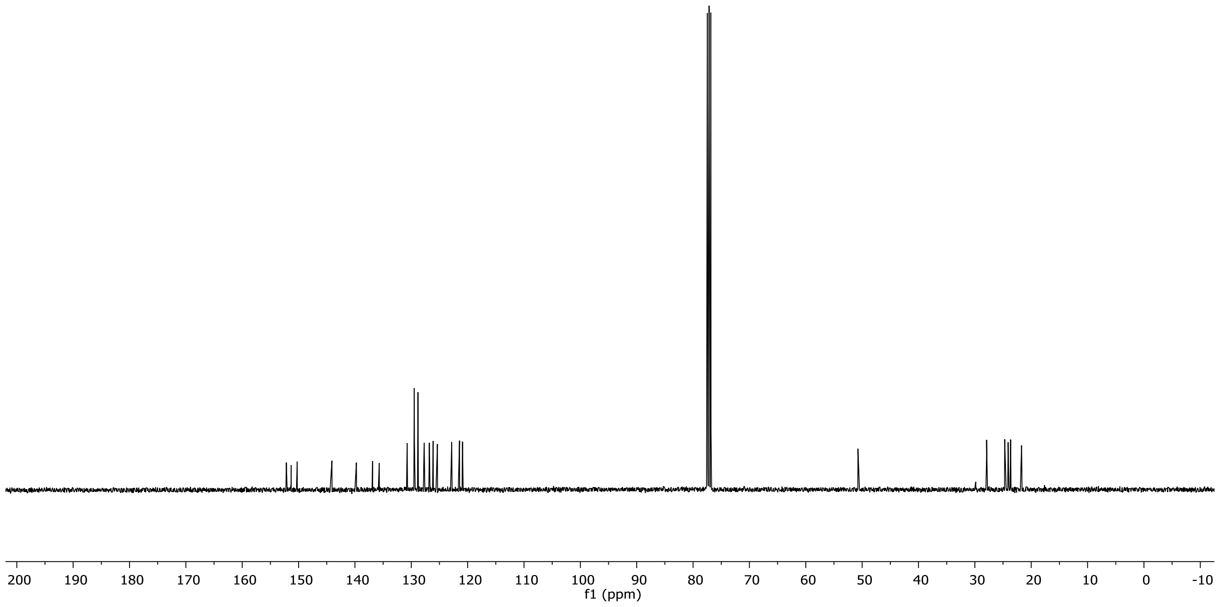
200 190 180 170 160 150 140 130 120 110 100 90 80 70 60 50 40 30 20 10 0 -10
f1 (ppm)

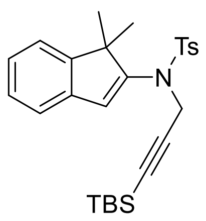
Ts
N
TBS

4y

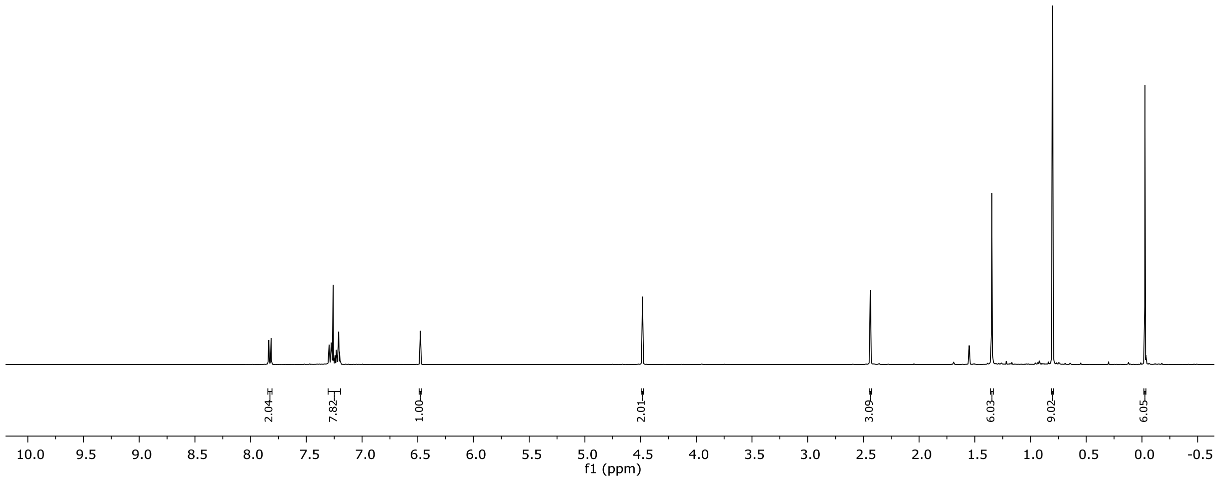
2.04
7.82
1.00
2.01
3.09
6.03
9.02
6.05
10.0 9.5 9.0 8.5 8.0 7.5 7.0 6.5 6.0 5.5 5.0 4.5 4.0 3.5 3.0 2.5 2.0 1.5 1.0 0.5 0.0 -0.5
f1 (ppm)

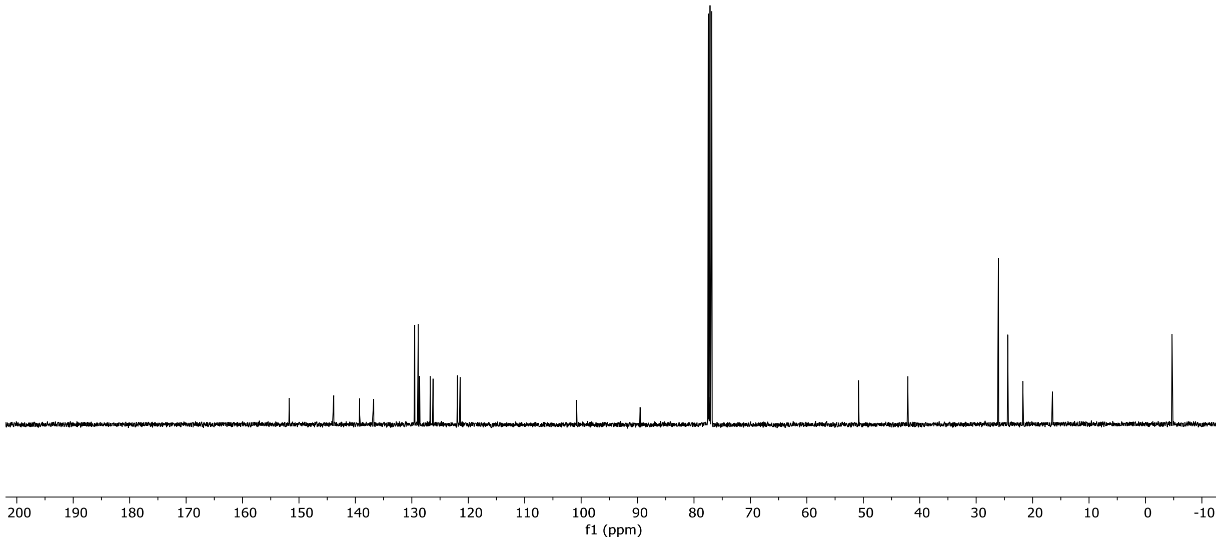
200 190 180 170 160 150 140 130 120 110 100 90 80 70 60 50 40 30 20 10 0 -10
f1 (ppm)

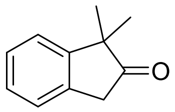
O

5

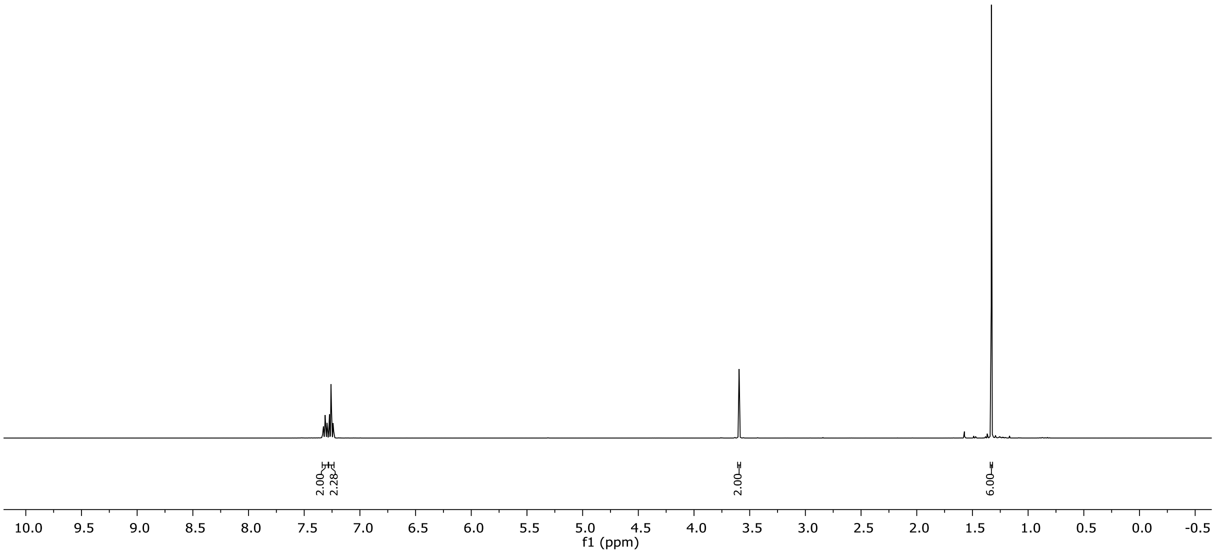
2.00
2.28
2.00
6.00
10.0 9.5 9.0 8.5 8.0 7.5 7.0 6.5 6.0 5.5 5.0 4.5 4.0 3.5 3.0 2.5 2.0 1.5 1.0 0.5 0.0 -0.5
f1 (ppm)

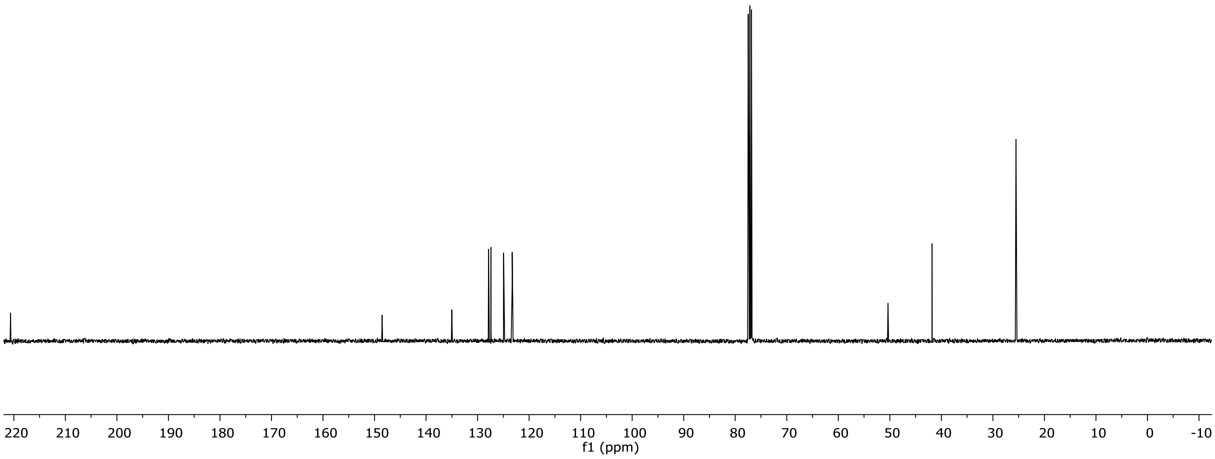
220 210 200 190 180 170 160 150 140 130 120 110 100 90 80 70 60 50 40 30 20 10 0 -10
f1 (ppm)

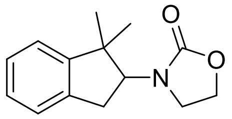
O
N
O

6

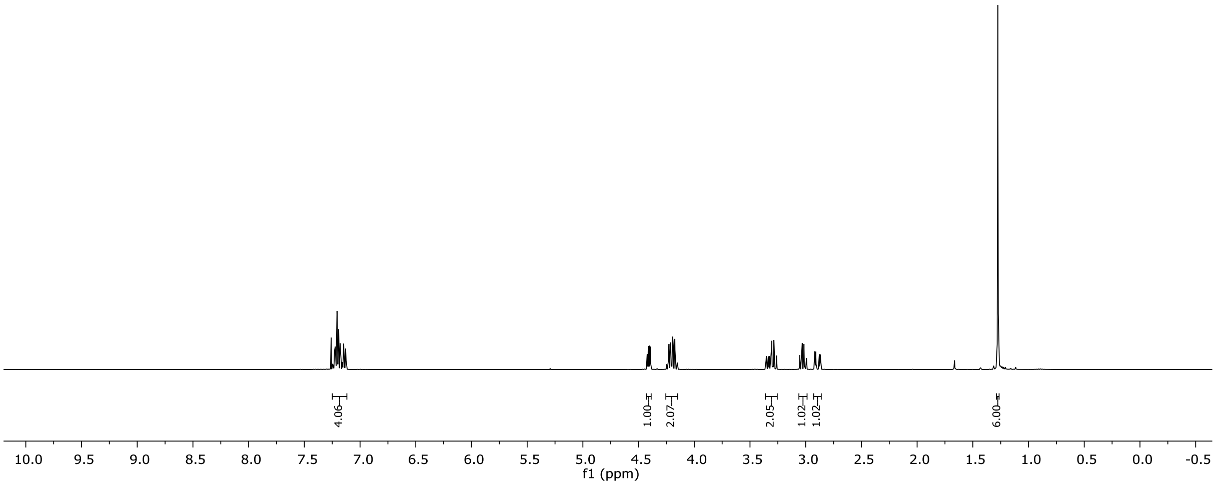
4.06
1.00
2.07
2.05
1.02
1.02
6.00
10.0
9.5
9.0
8.5
8.0
7.5
7.0
6.5
6.0
5.5
5.0
4.5
4.0
3.5
3.0
2.5
2.0
1.5
1.0
0.5
0.0
-0.5
f1 (ppm)

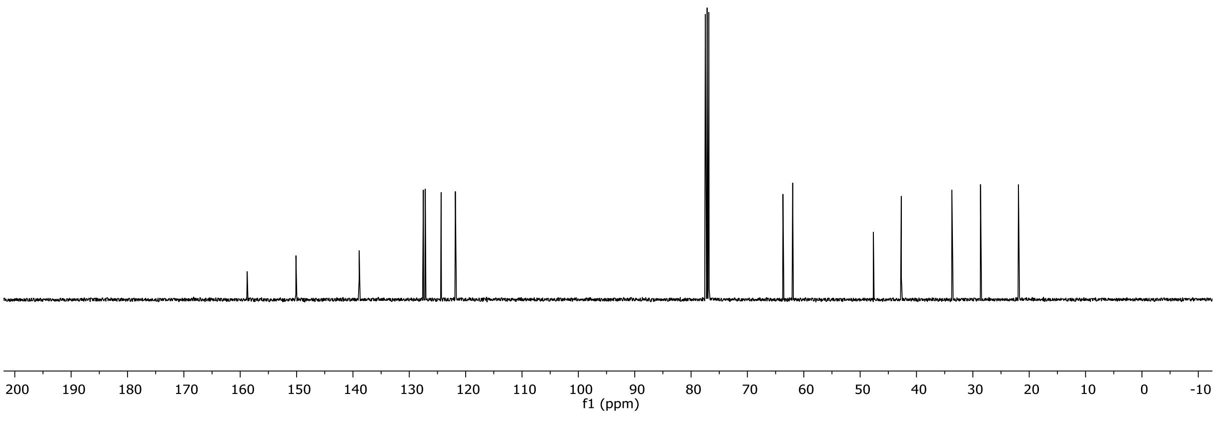
200
190
180
170
160
150
140
130
120
110
100
90
80
70
60
50
40
30
20
10
0
-10
f1 (ppm)

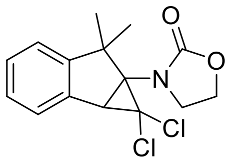
O
N
O
Cl
Cl

7

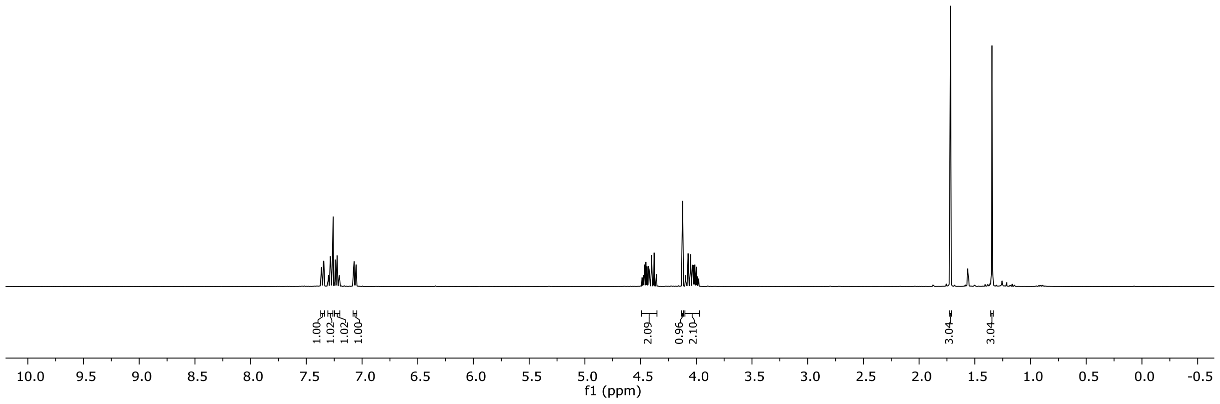
1.00
1.02
1.02
1.00
2.09
0.96
2.10
3.04
3.04
10.0 9.5 9.0 8.5 8.0 7.5 7.0 6.5 6.0 5.5 5.0 4.5 4.0 3.5 3.0 2.5 2.0 1.5 1.0 0.5 0.0 -0.5
f1 (ppm)

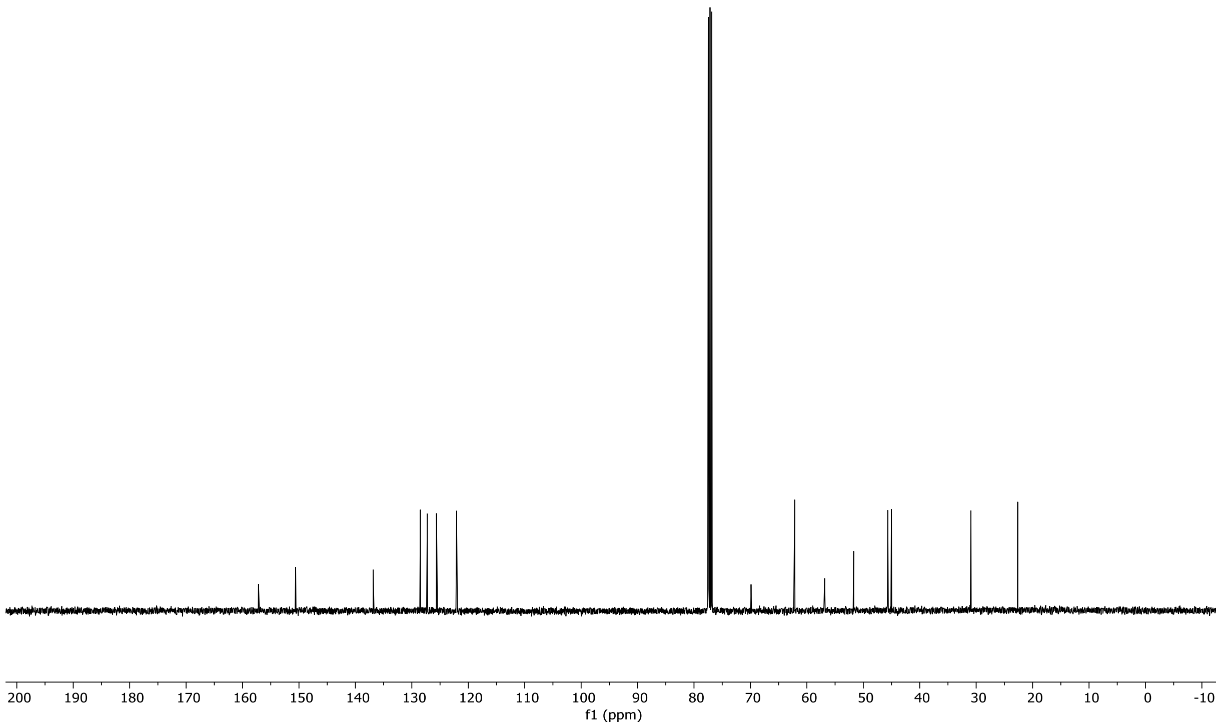
200 190 180 170 160 150 140 130 120 110 100 90 80 70 60 50 40 30 20 10 0 -10
f1 (ppm)

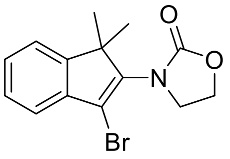
O
N
O
Br

8

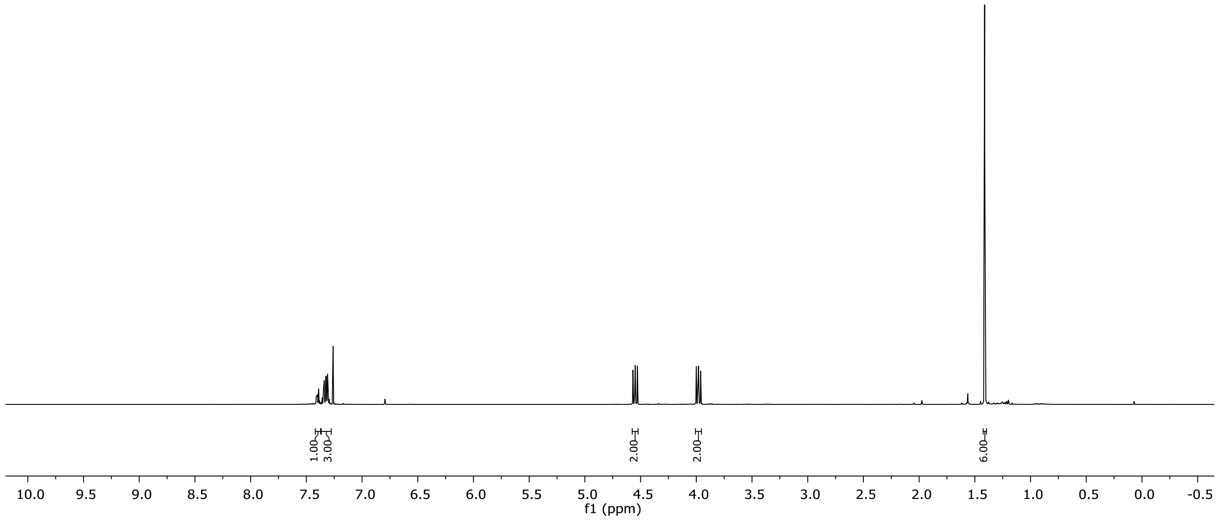
1.00
3.00
2.00
2.00
6.00
10.0 9.5 9.0 8.5 8.0 7.5 7.0 6.5 6.0 5.5 5.0 4.5 4.0 3.5 3.0 2.5 2.0 1.5 1.0 0.5 0.0 -0.5
f1 (ppm)

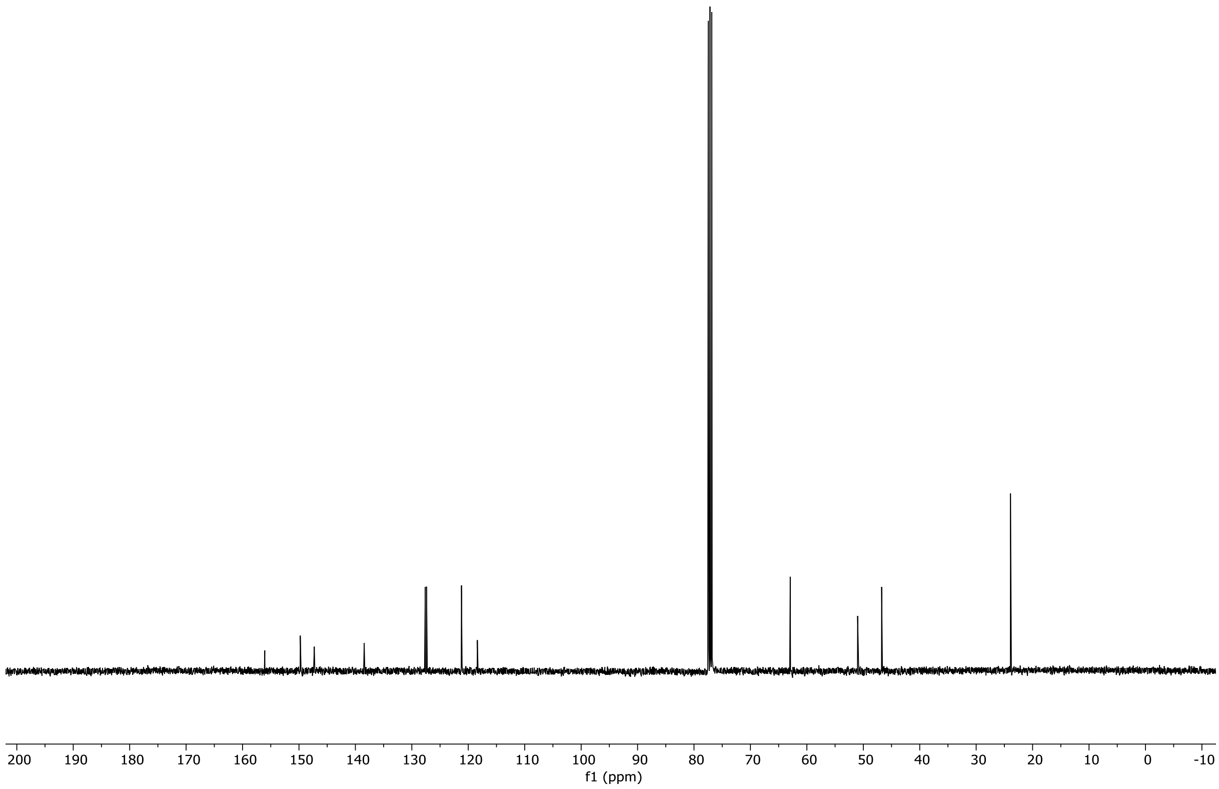
200 190 180 170 160 150 140 130 120 110 100 90 80 70 60 50 40 30 20 10 0 -10
f1 (ppm)

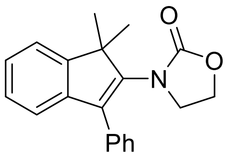
O
N
O
Ph

9

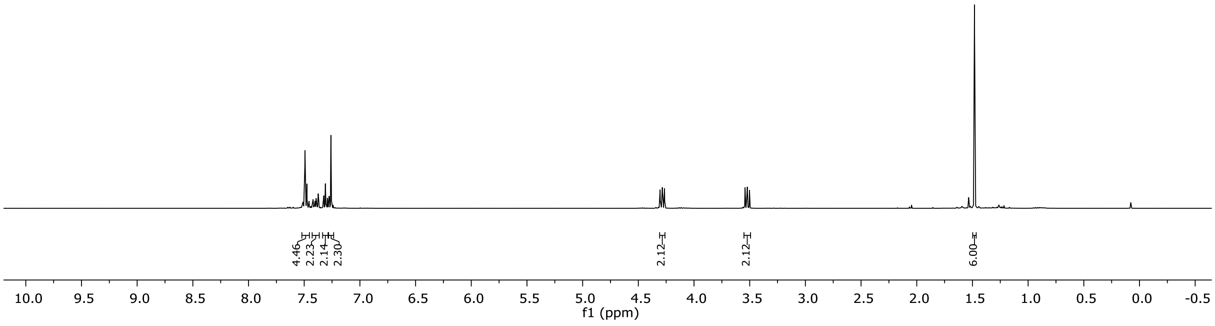
4.46
2.23
2.14
2.30
2.12
2.12
6.00
10.0 9.5 9.0 8.5 8.0 7.5 7.0 6.5 6.0 5.5 5.0 4.5 4.0 3.5 3.0 2.5 2.0 1.5 1.0 0.5 0.0 -0.5
f1 (ppm)

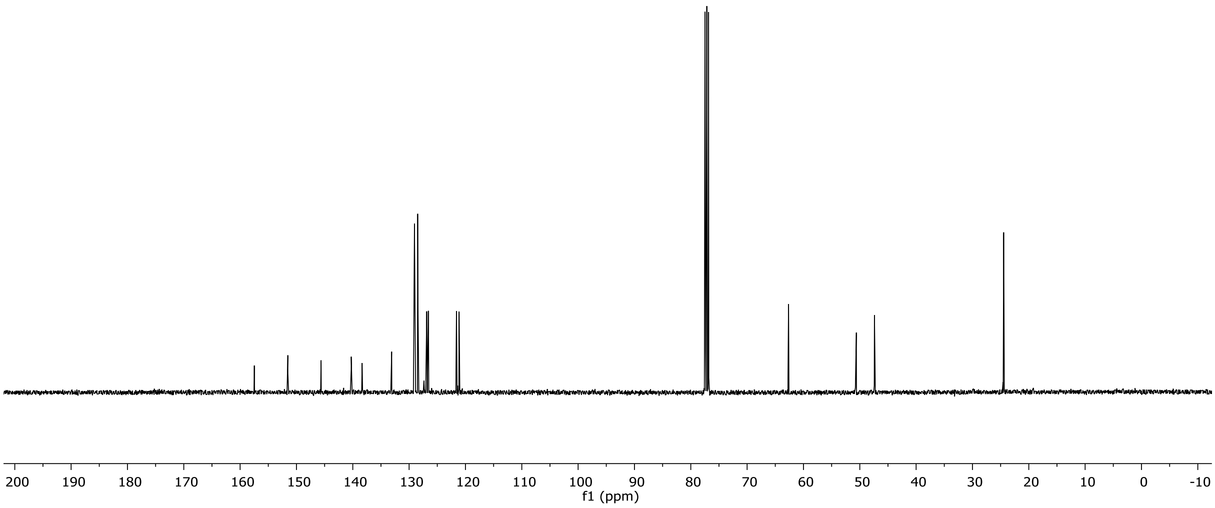
200 190 180 170 160 150 140 130 120 110 100 90 80 70 60 50 40 30 20 10 0 -10
f1 (ppm)

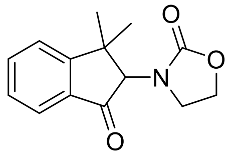
O
N
O
O

10

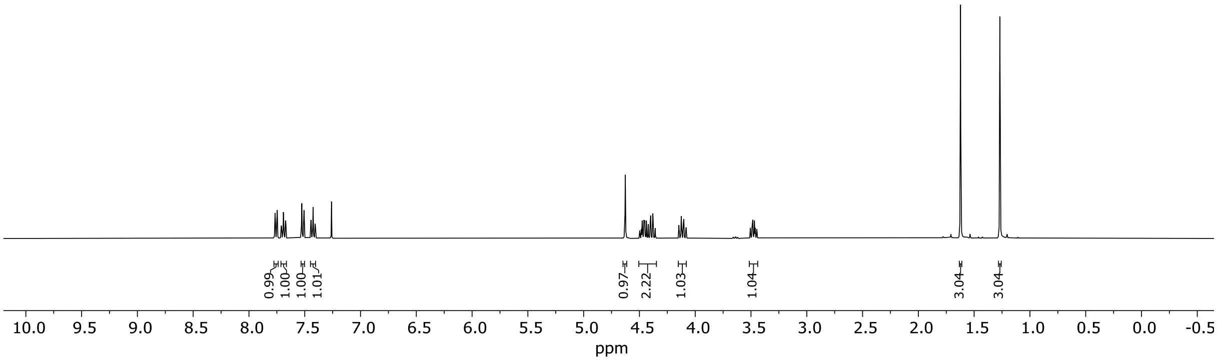
0.99
1.00
1.00
1.01
0.97
2.22
1.03
1.04
3.04
3.04
10.0 9.5 9.0 8.5 8.0 7.5 7.0 6.5 6.0 5.5 5.0 4.5 4.0 3.5 3.0 2.5 2.0 1.5 1.0 0.5 0.0 -0.5
ppm

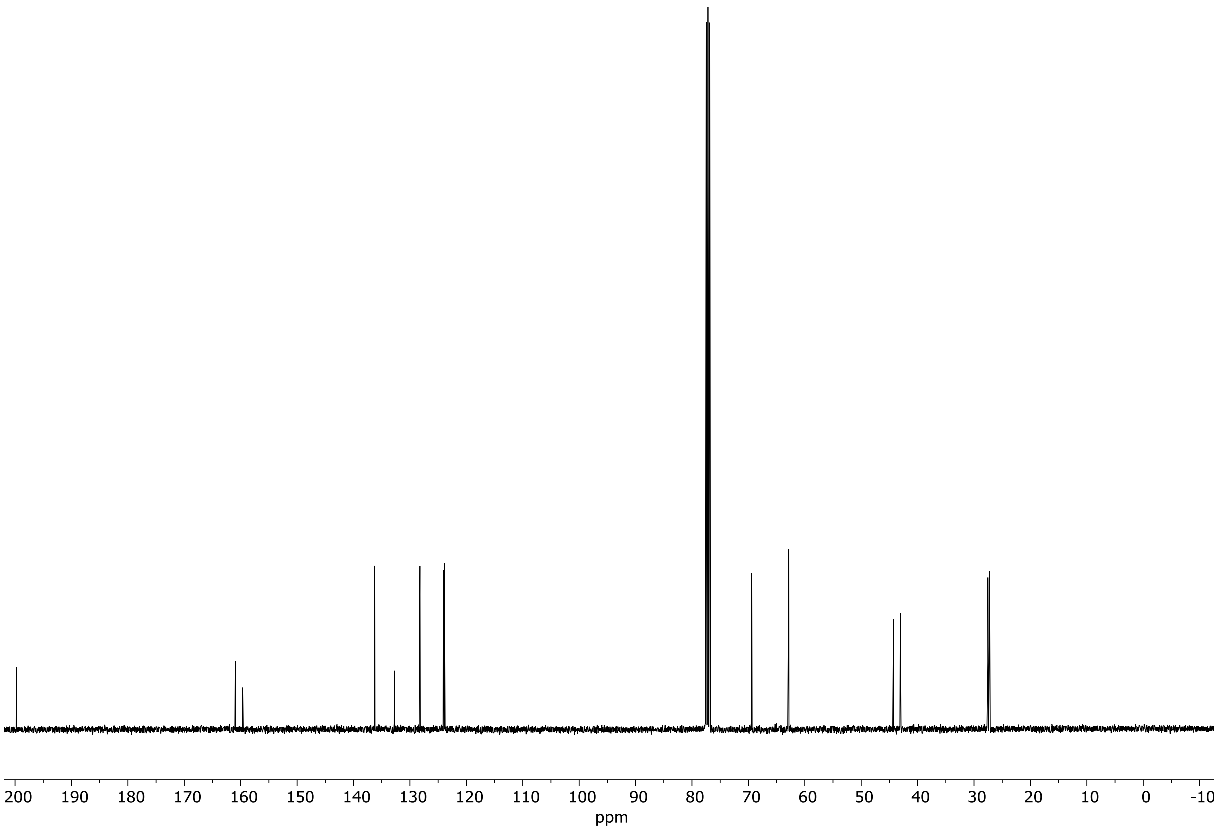
200 190 180 170 160 150 140 130 120 110 100 90 80 70 60 50 40 30 20 10 0 -10
ppm

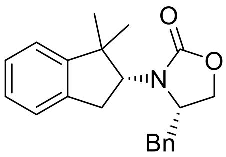
O
N
O
Bn

11

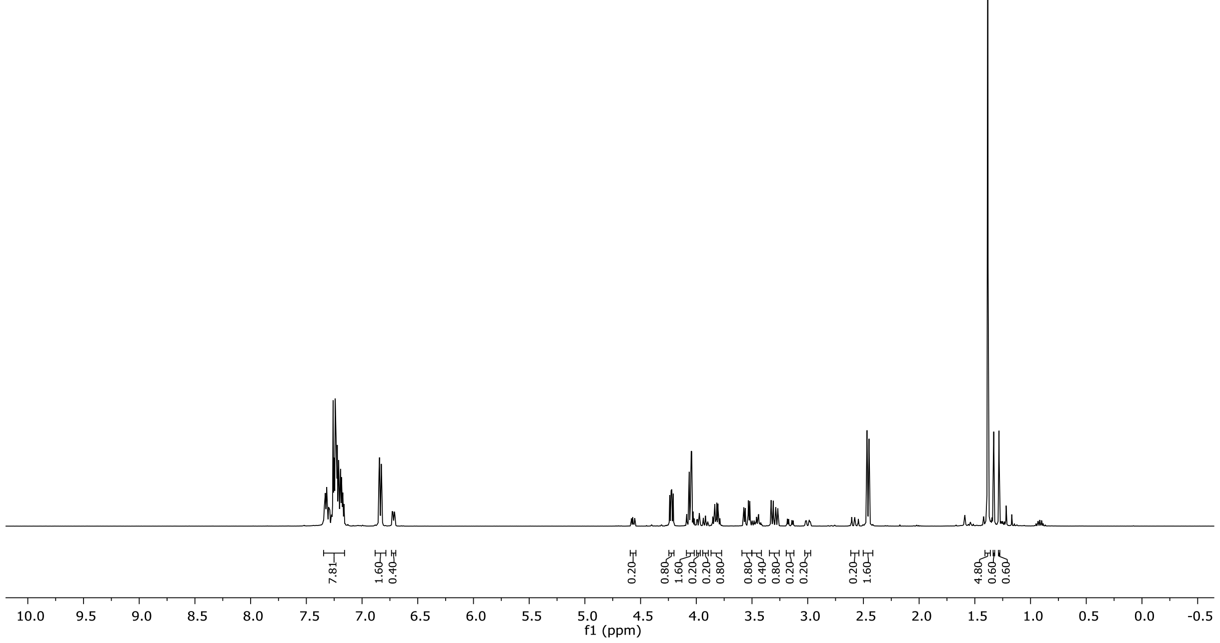
7.81
1.60
0.40
0.20
0.80
1.60
0.20
0.20
0.20
0.80
0.80
0.40
0.80
0.20
0.20
0.20
1.60
4.80
0.60
0.60
10.0 9.5 9.0 8.5 8.0 7.5 7.0 6.5 6.0 5.5 5.0 4.5 4.0 3.5 3.0 2.5 2.0 1.5 1.0 0.5 0.0 -0.5
f1 (ppm)

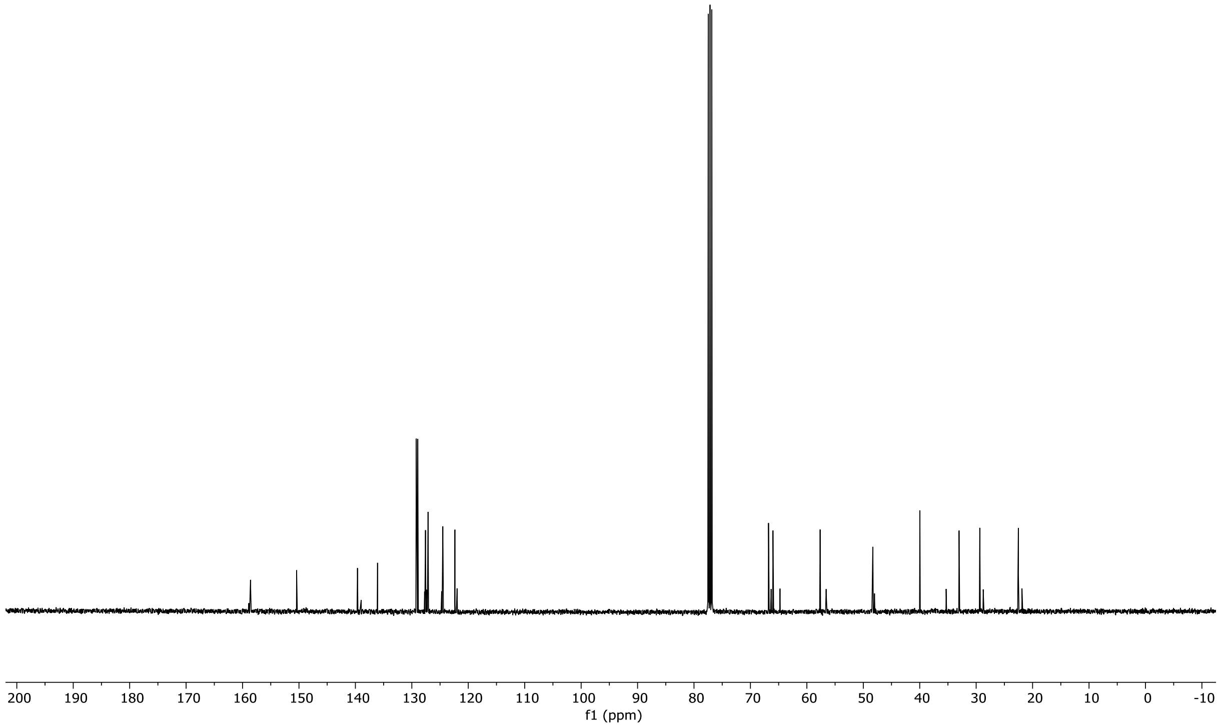
200 190 180 170 160 150 140 130 120 110 100 90 80 70 60 50 40 30 20 10 0 -10
f1 (ppm)
